# Comprehensive Analyses of Cytochrome P450 Monooxygenases and Secondary Metabolite Biosynthetic Gene Clusters in *Cyanobacteria*

**DOI:** 10.3390/ijms21020656

**Published:** 2020-01-19

**Authors:** Makhosazana Jabulile Khumalo, Nomfundo Nzuza, Tiara Padayachee, Wanping Chen, Jae-Hyuk Yu, David R. Nelson, Khajamohiddin Syed

**Affiliations:** 1 Department of Biochemistry and Microbiology, Faculty of Science and Agriculture, University of Zululand, KwaDlangezwa 3886, South Africa; khosietens@gmail.com (M.J.K.); nomfundonzuza11@gmail.com (N.N.); teez07padayachee@gmail.com (T.P.); 2 Department of Molecular Microbiology and Genetics, University of Göttingen, 37077 Göttingen, Germany; chenwanping1@foxmail.com; 3 Department of Bacteriology, University of Wisconsin-Madison, 3155 MSB, 1550 Linden Drive, Madison, WI 53706, USA; jyu1@wisc.edu; 4 Department of Systems Biotechnology, Konkuk University, Seoul 05029, Korea; 5 Department of Microbiology, Immunology and Biochemistry, University of Tennessee Health Science Center, Memphis, TN 38163, USA

**Keywords:** cytochromes P450 monooxygenases, secondary metabolites, *Cyanobacteria*, biosynthetic gene clusters, gene-cluster diversity percentage, mathematical formula, phylogenetic analysis

## Abstract

The prokaryotic phylum *Cyanobacteria* are some of the oldest known photosynthetic organisms responsible for the oxygenation of the earth. Cyanobacterial species have been recognised as a prosperous source of bioactive secondary metabolites with antibacterial, antiviral, antifungal and/or anticancer activities. Cytochrome P450 monooxygenases (CYPs/P450s) contribute to the production and diversity of various secondary metabolites. To better understand the metabolic potential of cyanobacterial species, we have carried out comprehensive analyses of P450s, predicted secondary metabolite biosynthetic gene clusters (BGCs), and P450s located in secondary metabolite BGCs. Analysis of the genomes of 114 cyanobacterial species identified 341 P450s in 88 species, belonging to 36 families and 79 subfamilies. In total, 770 secondary metabolite BGCs were found in 103 cyanobacterial species. Only 8% of P450s were found to be part of BGCs. Comparative analyses with other bacteria *Bacillus*, *Streptomyces* and mycobacterial species have revealed a lower number of P450s and BGCs and a percentage of P450s forming part of BGCs in cyanobacterial species. A mathematical formula presented in this study revealed that cyanobacterial species have the highest gene-cluster diversity percentage compared to *Bacillus* and mycobacterial species, indicating that these diverse gene clusters are destined to produce different types of secondary metabolites. The study provides fundamental knowledge of P450s and those associated with secondary metabolism in cyanobacterial species, which may illuminate their value for the pharmaceutical and cosmetics industries.

## 1. Introduction

*Cyanobacteria* are thought to be some of the oldest known photosynthetic organisms that played a major role in the evolution of life by contributing to the oxygenation of the earth’s atmosphere [1,2,3,4,5]. These Gram-negative photosynthetic prokaryotes also played a significant role in nitrogen and carbon cycles as they are able to fix atmospheric nitrogen and carbon. It is also well known that cyanobacterial species are responsible for the evolution of plants on earth owing to their endosymbiotic lifestyle, which led to the development of light-harvesting organelles in plants [6,7]. Cyanobacterial species can be found in diverse terrestrial habitats, ranging from the oceans to fresh-water bodies, soil to desert rocks, and extreme environments such as Antarctic dry valleys, Arctic and thermophilic lakes [8,9].

To survive in a wide range of environments, cyanobacterial species produce diverse natural products comprising both primary and secondary metabolites belonging to the group’s non-ribosomal proteins, polyketides, and terpenes and alkaloids. These products have varying activities of anticancer, antiviral, and ultraviolet-protective activities, as well as hepatotoxicity and neurotoxicity [10,11]. Many cyanobacterial species are used as model organisms to understand fundamental processes such as photosynthesis, nitrogen fixation, and circadian rhythm [12,13,14]. Owing to their amenability to gene manipulation, these organisms have been genetically modified/engineered for the production of valuable human compounds [15,16]. Despite all their highly beneficial characteristics, cyanobacterial species are also known to cause cyanobacterial blooms in water, resulting in the production of toxins that are harmful to humans, wild and domestic animals [17]. An overview of some of the cyanobacterial species’ beneficial and harmful characteristics is presented in Table 1.

Currently, world-wide research continues to identify novel secondary metabolites with potential biotechnological value from cyanobacterial species. These secondary metabolites are usually produced by a set of genes that are clustered in an organism, and these clusters are known as secondary metabolite biosynthetic gene clusters (BGCs) [51]. Among different genes involved in the production of secondary metabolites, P450s occupy a special place, as these enzymes contribute to the diversity of secondary metabolites due to regio- and stereo-specific oxidation of substrates [52,53]. Recent studies in different bacterial populations belonging to the genera *Bacillus* [54], *Streptomyces* and *Mycobacterium* [55,56] revealed the presence of a large number of P450s in secondary metabolite BGCs. A paper published in 2010 [11] reported some P450s present in cyanobacterial species where the authors performed blast analysis using P450s from *Nostoc* sp. strain PCC 7120 [57] and *Synechocystis* sp. PCC 6803 [58]. However, to date, a comprehensive comparative analysis of P450s and those associated with secondary metabolism in cyanobacterial species has not been reported. The availability of a large number of cyanobacterial species genomes gives us an opportunity to address these issues. In this study, genome data-mining, annotation and phylogenetic analysis of P450s in 114 cyanobacterial species were performed. The study also reports a comparative analysis of secondary metabolite BGCs in cyanobacterial species and identification of P450s that are part of the different BGCs. Furthermore, a comparative analysis of P450s and secondary metabolite key features of cyanobacterial species with other bacterial species belonging to the genera *Bacillus*, *Streptomyces* and *Mycobacterium* is presented.

## 2. Results and Discussion

### 2.1. Cyanobacterial Species Have Lowest Number of P450s

**Figure 1:**
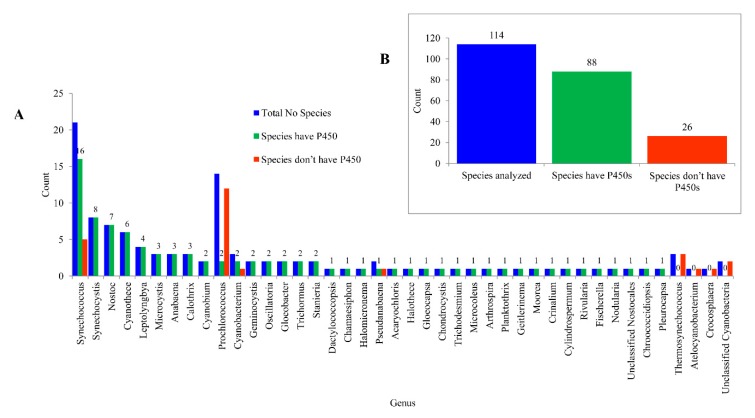
Analysis of P450s in *Cyanobacteria*. P450s were analyzed at both the genus level (**A**) and species level (**B**). The numbers next to the bars indicate the number of species. In Panel A, only species numbers for the species that have P450s are presented. Detailed information is presented in Tables S1 and S2.

Analysis of 41 cyanobacterial genera and species belonging to the unclassified *Cyanobacteria* (Table S1) revealed the presence of P450s in all cyanobacterial genera except in the genera *Thermosynechococcus*, *Atelocyanobacterium* and *Crocosphaera* (Figure 1). This may be due to the lowest number of species genomes being available for analysis in these genera. Furthermore, two species in the unclassified *Cyanobacteria* group did not have P450s (Figure 1). Analysis of P450s at species level revealed that among 114 species, 88 species had P450s and 26 species did not have P450s in their genomes (Figure 1), indicating that most of the species in different genera had P450s.

**Figure 2:**
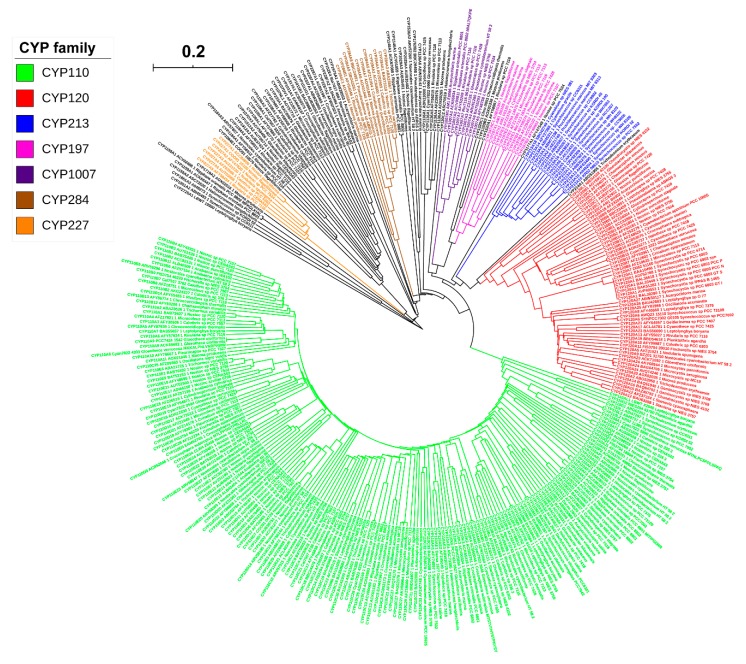
Phylogenetic analysis of cyanobacterial species P450s. Dominant P450 families are indicated in different colours. A high-resolution phylogenetic tree is provided as Supplementary Dataset 2.

**Figure 3:**
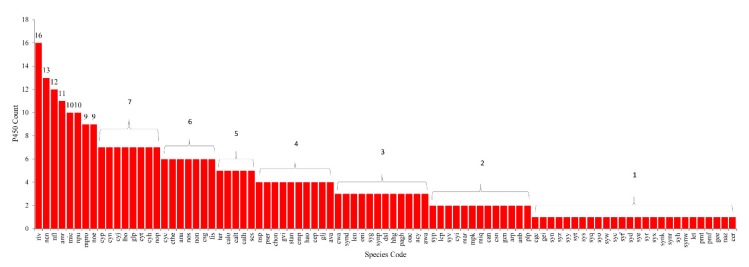
Comparative analysis of P450s in cyanobacterial species. The numbers next to bars indicate the number of P450s in each species. The species names with respect to their codes can be found in Table S2.

In total, 341 P450s were found in 88 cyanobacterial species (Figure 2 and Figure 3, Table S2). The analysis also revealed the presence of 13 P450-fragments and 15 P450 false positives in different cyanobacterial species (Table S2). A list of P450s, P450-fragments, and P450 false positives identified in cyanobacterial species, along with their sequences, is presented in Supplementary Dataset 1. The occurrence of P450-fragments and P450 false positives in organisms is quite common and these sequences were not taken for further analysis. Among cyanobacterial species, *Rivularia* sp. PCC 7116 has the highest number of P450s (16 P450s), followed by *Nostocales cyanobacterium* HT-58-2 (13 P450s), and *Nostoc flagelliforme* (12 P450s) (Figure 3 and Table S2). Most of the cyanobacterial species have a single P450 in their genome (Figure 3). Comparative analysis with other bacterial species revealed that cyanobacterial species have the lowest number of P450s compared to *Bacillus* species, *Streptomyces* and mycobacterial species (Table 2). Cyanobacterial species have an average of three P450s in their genomes compared to an average of four P450s in *Bacillus* species [54], 30 P450s in mycobacterial species [59], and 34 P450s in *Streptomyces* species [55] (Table 2).

### 2.2. CYP110 is the Dominant P450 Family in Cyanobacterial Species

**Figure 4:**
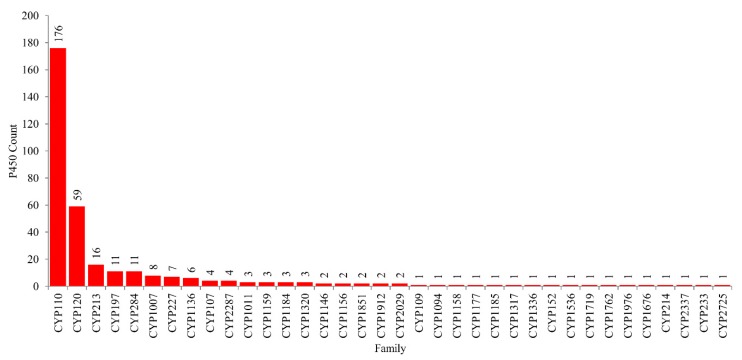
Family level comparative analysis of P450s in the species of *Cyanobacteria*. The numbers next to the family bar indicate the number of P450s. The data on the number of P450 families, along with subfamilies, are presented in Supplementary Table S3.

As per the International P450 Nomenclature Committee rules [60,61,62], 341 P450s found in 88 cyanobacterial species can be grouped into 36 P450 families and 79 P450 subfamilies (Figure 4 and Table S3). Phylogenetic analysis of cyanobacterial species P450s revealed grouping of P450s belonging to the same family together on the tree (Figure 2), indicating the correct assignment of P450 families and subfamilies. Among 36 P450 families, CYP110 has the highest number of P450s (176 P450s), followed by CYP120 (59 P450s), CYP213 (16 P450s) and P450 families, CYP197 and CYP284, which have 11 P450s in each (Figure 4). Comparative analysis of P450 families among different bacterial species revealed that different bacterial species have different dominant P450 families (Table 1). CYP110 is the dominant P450 family in cyanobacterial species, whereas CYP125 is dominant in mycobacterial species and CYP107 in *Bacillus* species and *Streptomyces* species (Table 2). The molecular basis for the blooming (P450 family with many members) [63] of certain P450 families is attributed to the species habitat and lifestyle [55]. In addition to this, P450 subfamily-level blooming was observed in cyanobacterial species, where some subfamilies were found to be dominant in a particular family (Table S3). For example, among 26 subfamilies found in the CYP110 family, subfamilies C (34 P450s), E (29 P450s), D (26 P450s) and A (15 P450s) are dominant; among six subfamilies found in CYP120 family, subfamilies A (35 P450s) and B (16 P450s) are dominant (Table S3), indicating the subfamily-level blooming of P450s in cyanobacterial species.

Apart from five P450 families, the remaining 31 P450 families in cyanobacterial species have a single-digit number of members (Figure 4). In fact, 17 P450 families have a single P450, indicating high P450 family diversity in cyanobacterial species. This was further confirmed when the P450 diversity percentage was compared among different bacterial species (Table 2). The P450 diversity percentage in cyanobacterial species was found to be highest compared to *Bacillus* species and mycobacterial species and almost 50% lower compared to *Streptomyces* species (Table 2). The highest P450 diversity observed for cyanobacterial species indicates that these P450s might have diverse roles, as was observed for *Streptomyces* species [55]. However, future functional analysis of cyanobacterial species P450s will provide more evidence on this observation.

**Figure 5:**
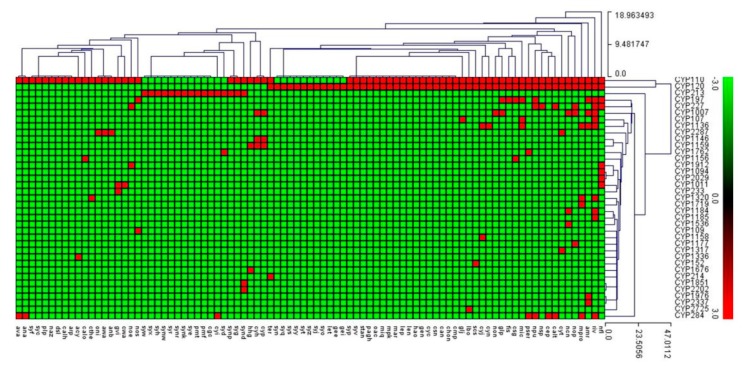
Heat-map of the presence/absence of P450 families in 88 cyanobacterial species. The data is represented as 3 for family presence (**red**) and –3 for family absence (**green**). Eighty-nine cyanobacterial species form the horizontal axis and P450 family numbers form the vertical axis. A detailed table showing P450 family profiles in each of the cyanobacterial species is presented in Supplementary Dataset 3.

P450 family conservation analysis revealed that none of the 36 families were conserved in 88 cyanobacterial species (Figure 5). The P450 profile heat-map revealed that the P450 families CYP110 and CYP120 were found to be a co-presence in most of the cyanobacterial species (Figure 5). Non-conservation of P450 families was also observed in *Bacillus* species [54], but in the *Streptomyces* [55] and mycobacterial species [59] quite a large number of P450 families were found to be conserved.

### 2.3. Cyanobacterial Species Have Lowest Secondary Metabolite Biosynthetic Gene Clusters

**Figure 6:**
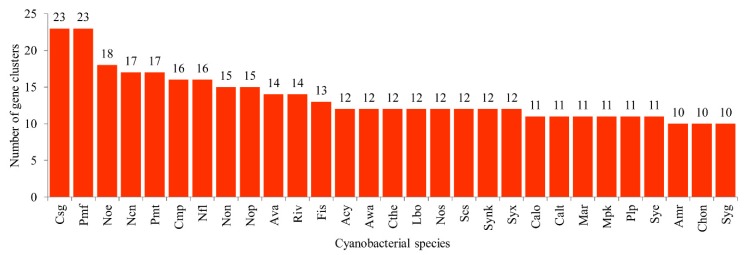
Comparative analysis of secondary metabolite biosynthetic gene clusters (BGCs) in cyanobacterial species. Species with a two-digit number of secondary metabolite BGCs are shown in the figure. The species names with respect to their codes can be found in Table S1. Detailed information on each of the species’ secondary metabolite BGCs is presented in Table S2.

A total of 770 secondary metabolite BGCs were found in 103 cyanobacterial species (Table S2). Species-wise comparative analysis revealed that 29 cyanobacterial species have at least two-digit numbers of secondary metabolites BGCs in their genomes (Figure 6). Among cyanobacterial species, *Cylindrospermum stagnale* (Csg) and *Prochlorococcus marinus* MIT 9303 (Pmf) have the highest number of secondary metabolite BGCs (23 BGCs in each), followed by *Nostoc* sp. CENA543 (Noe) (18 BGCs) and 17 BGCs each in *Nostocales cyanobacterium* HT-58-2 (Ncn) and *P. marinus* MIT 9313 (Pmt) (Figure 6). Comparative analysis of secondary metabolite BGCs revealed that cyanobacterial species have the lowest number of secondary metabolite BGCs in their genomes compared to *Bacillus* species, mycobacterial species and *Streptomyces* species (Table 2). On average, seven secondary metabolite BGCs were found in cyanobacterial species compared to nine in *Bacillus* species, 15 in mycobacterial species and 30 in *Streptomyces* species (Table 2). This indicates that *Streptomyces* species dominate the production of secondary metabolites and this is the reason why more than 80% of the antibiotics that are in use today are in fact sourced from *Streptomyces* species [64].

### 2.4. Cyanobacterial Species Has Highest Gene Cluster Diversity Percentage Compared to Bacillus and Mycobacterial Species

**Figure 7:**
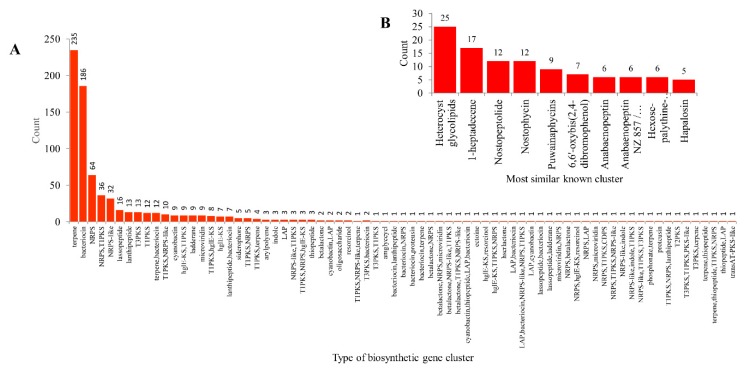
Comparative analysis of types of secondary metabolite biosynthetic gene clusters (BGCs) in 103 cyanobacterial species (**A**) and most similar known clusters (**B**). Standard abbreviations representing secondary metabolite BGCs as indicated in anti-SMASH (antibiotics & Secondary Metabolite Analysis Shell) [65] were used in the figure. Detailed information is presented in Supplementary Table S4.

Analysis of types of gene clusters in 103 cyanobacterial species revealed the presence of 73 different types of secondary metabolite BGCs (Figure 7 and Table S4). Among secondary metabolite BGCs, terpene BGC is dominant (235 clusters), followed by bacteriocin (183 clusters) and non-ribosomal peptides (NRPS) (64 clusters) (Figure 7). Forty types of BGCs have only a single gene cluster, indicating the highest diversity in types of gene clusters in cyanobacterial species (Figure 7). Comparative analysis of types of BGCs among different bacterial species revealed that cyanobacterial species have the highest number of types of BGCs compared to *Bacillus* and mycobacterial species, but the lowest compared to *Streptomyces* species (Table 1).

In order to measure accurate BGC diversity among different bacterial species, we have developed a new equation, similar to the one we developed for P450 diversity percentage calculation [55], with some modification. The formula below will nullify the number of species used and will give an accurate gene cluster diversity percentage comparison between different populations.

$$\mathrm{Gene}\mathrm{cluster}\mathrm{diversity}\mathrm{percentage}=\frac{100\times \mathrm{Total}\mathrm{number}\mathrm{of}\mathrm{types}\mathrm{of}\mathrm{clusters}}{\mathrm{Total}\mathrm{number}\mathrm{of}\mathrm{clusters}\times \mathrm{number}\mathrm{of}\mathrm{species}}$$

Based on the above formula, the gene cluster diversity percentage in cyanobacterial species was found to be four times higher compared to *Bacillus* and mycobacterial species (Table 2). This indicates that despite cyanobacterial species having the lowest number of gene clusters, these clusters are diverse and destined to produce different types of secondary metabolites. This was evident when looking into the most similar known clusters where, among 770 clusters, only 228 clusters showed similarity to the 79 best known clusters (Figure 7 and Table S4). Among the known similar clusters, only four most similar known clusters are dominant, with 25 (heterocyst glycolipids) 17 (1-heptadecene) and 12 (Nostopeptolide and Nostophycin) (Figure 7 and Table S4). A detailed analysis on most similar known clusters is presented in Supplementary Table S4. The remaining 542 BGCs have no similar known clusters, indicating that these BGCs might encode novel secondary metabolites, possibly with potential biotechnological value.

### 2.5. Few Cyanobacterial Species P450s Found to be Part of Secondary Metabolite Biosynthetic Gene Clusters

Analysis of P450s that are part of different secondary metabolite BGCs revealed that only a few P450s were part of secondary metabolite BGCs in cyanobacterial species compared to *Bacillus*, mycobacterial and *Streptomyces* species (Table 2 and Table 3). Only 8% of P450s are part of BGCs in cyanobacterial species compared to other bacterial species, where 22% (*Bacillus* species), 11% (mycobacterial species) and 34% (*Streptomyces* species) of P450s were found to be part of BGCs (Table 2). Among 341 P450s only 27 P450s were found to be part of secondary metabolite BGCs in cyanobacterial species, indicating that cyanobacterial species P450s might play a major role in their primary metabolism. The 27 P450s that are part of BGCs belong to six P450 families (Table 3). P450s belonging to the CYP110 family are dominantly present in BGCs (17 P450s—63%), followed by CYP213 (4 P450s—15%), CYP120 (3 P450s—11%) and a single member found in P450 families CYP1011, CYP1185, and CYP197 (Table 3). A point to be noted is that the CYP110 family is dominantly present in cyanobacterial species, indicating its requirement for the production of secondary metabolites, as the same phenomenon was observed where dominant P450 families were found to be part of BGCs in *Bacillus*, mycobacterial and *Streptomyces* species [54,55]. The 27 P450s were found to be part of 10 types of clusters, where nine P450s were found to be part of an NRPS, Type I PKS (polyketide synthase), followed by five P450s that were part of terpene and three P450s that were part of bacteriocin (Table 2). P450s found in each of the clusters and most similar known clusters were presented in Table 3. Analysis of the most similar known clusters revealed that CYP110AH1 from *Synechococcus* sp. PCC 7502 is certainly involved in the production of anabaenopeptin NZ 857/nostamide, as this P450 NRPS cluster showed 100% similarity to the gene cluster that produces the metabolite (Table 3). Apart from this match, the percentage similarity to most known clusters is very low and thus the metabolites produced by different gene clusters cannot be predicted.

### 2.6. Cyanobacterial Species P450s Functions and Features Resemblance to Eukaryotic P450s

Functional analysis of a few cyanobacterial species P450s revealed that these P450s have some unusual catalytic diversity and resemblance to eukaryotic P450s. Based on our study, it can safely be predicted that the 27 cyanobacterial species P450s listed in Table 3 are involved in the production of different secondary metabolites. Some of the cyanobacterial species P450 functions against different compounds were elucidated. However, the biological relevance of these reactions is not clear. CYP120A1 from *Synechocystis* sp. PCC 6803 was found to be the first non-animal retinoic acid hydroxylase [66]. This P450-catalyzed reaction represents a novel modification of retinoids compared to vertebrate CYP26 family P450s. CYP120A1 hydroxylated all-*trans*-retinoic acid at C16 or C17 positons and converted *cis*-retinoic acids (9-*cis*-retinoic acid and 13-*cis*-retinoic acid), retinal, 3(*R*)-OH-retinal, retinol, β-apo-13-carotenone (C_18_) and β-apo-14′-carotenal (C_22_) resulted in the formation of the corresponding hydroxyl derivatives [66]. CYP120A1 had the highest preference for all-*trans* substrates compared to *cis*- substrates. Among the compounds analysed, CYP120A1 had the highest activity of all-*trans*-retinoic acids, followed by β-apo-13-carotenone (C_18_) [66]. CYP110C1 from *Nostoc* sp. PCC7120 was found to be germacrene A hydroxylase involved in sesquiterpene biosynthesis [57,67]. CYP110C1 converted germacrene A into two different products and the predominant product of this reaction was identified as 1,2,3,5,6,7,8,8aoctahydro-6-isopropenyl-4,8a-dimethylnaphth-1-ol [67]. CYP110E1 from the *Nostoc* sp. strain PCC 7120 was found to be flavone synthase, the first prokaryotic P450 with such activity [68]. CYP110E1 hydroxylated naringenin and (hydroxyl) flavanones into apigenin and (hydroxyl) flavones [68]. CYP110E1 also hydroxylated different compounds such as sesquiterpenes (zerumbone), drugs (ibuprofen and flurbiprofen), and aryl compounds (1-methoxy and 1-ethoxy naphthalene) into novel compounds that are usually difficult to synthesize chemically [68]. CYP110A1 from *Nostoc* sp. PCC 7120 was predicted to be a fatty acid ω-hydroxylase as the purified P450 binds to long-chain saturated and unsaturated fatty acids [69]. Unlike other prokaryotic P450s, CYP110A1 was found to be associated with membrane fraction, indicating its close resemblance to eukarotic P450s [69].

P450s’ role in the synthesis of carotenoids, light-harvesting pigments, in cyanobacterial species will help in addressing an evolutionary link between these species and plants since cyanobacterial species considered as precursors of chloroplasts in plants [6,7]. It is well-known that members of the CYP97 P450 family are conserved across plant taxa [70] and involved in the synthesis of carotenoids in plants [71,72]. Furthermore, research in this direction will also help in identifying the biological relevance of P450s in cyanobacterial species.

## 3. Materials and Methods

### 3.1. Species and Databases

In this study, 114 cyanobacterial species belonging to 41 genera and unclassified *Cyanobacteria* that are available for public use at Kyoto Encyclopaedia of Genes and Genomes (KEGG) [73] were used. Detailed information on different genera and species used in this study is presented in Table S1.

### 3.2. Genome Data Mining and Annotation of P450s

Genome data mining of P450s and their annotation was carried out following the procedure described in the literature [54,55]. Briefly, individual cyanobacterial species proteomes were downloaded from KEGG and subjected to NCBI Batch Web CD-Search Tool analysis [74]. After analysis, the results were analyzed and proteins that belong to the P450 superfamily were selected. The selected proteins were subjected to BLAST analysis at http://bioshell.pl/p450/blast_only.html as part of the P450 page at http://www.p450.unizulu.ac.za/. Based on the percentage identity to the named homolog P450s, the proteins were then annotated (assigning P450 family and subfamily), following the International P450 Nomenclature Committee rule, i.e., sequences with >40% identity were assigned to the same family as named homolog P450 and sequences with >55% identity to the same subfamily as named homolog P450 [60,61,62]. Proteins with less than 40% identity to a named homolog P450 were assigned to a new P450 family.

### 3.3. Phylogenetic Tree Construction of Cyanobacterial Species P450s

The phylogenetic tree of cyanobacterial species P450s was built using the methodology described elsewhere [54,55,56]. Firstly, the alignment of cyanobacterial species P450s protein sequences was performed by the MAFFT v6.864 program available at the Trex web server [75] (http://www.trex.uqam.ca/index.php?action=mafft). Then, the alignments were automatically subjected to tree inferring and optimisation by the Trex web server [76]. Briefly, the server inferred the trees with different algorithms, including maximum likelihood, maximum parsimony, neighbor joining, in the library, and searched out the best phylogenetic tree in the least-squares sense. Finally, the best-inferred tree was envisioned and coloured using iTOL [77] (https://itol.embl.de/).

### 3.4. Generation of P450 Profile Heat-Maps

P450 profile heat-maps were generated following the method reported in the literature [56,78,79]. The presence or absence of P450s in cyanobacterial species was shown with heat-maps generated using P450 family data. The data were represented as 3 for family presence (red) and −3 for family absence (green). A tab-delimited file was imported into Mev 4.9 (multi-experiment viewer) [80]. Hierarchical clustering using a Euclidean distance metric was used to cluster the data. Eight-nine cyanobacterial species formed the horizontal axis and P450 families formed the vertical axis.

### 3.5. Secondary Metabolite Biosynthetic Gene Clusters Analysis

Secondary metabolite BGCs analysis in cyanobacterial species was carried out following the method as mentioned previously [54,55]. Briefly, individual cyanobacterial species genome IDs (Table S1) were submitted to anti-SMASH (antibiotics & Secondary Metabolite Analysis Shell) [65] for identification of secondary metabolite BGCs. The gene cluster information generated by anti-SMASH is analyzed for the presence of P450s by manually mining the cluster sequences. Information on the type of cluster, most similar known cluster and percentage similarity to a known cluster is also noted and presented in table format. Among 114 cyanobacterial species, 11 species’ genome IDs did not pass through anti-SMASH analysis. Thus, in this study, secondary metabolite BGCs data for 103 species is presented. Lists of species that are not part of the secondary metabolite cluster analysis are presented in Table S5.

### 3.6. Data Analysis

P450 diversity percentage analysis in cyanobacterial species was carried out following the method described elsewhere [55]. P450 diversity percentage is calculated using the formula: P450 diversity percentage = 100 × Total number of P450 families/Total number of P450s × Number of species. The average number of P450s was calculated using the formula: Average number of P450s = Number of P450s/Number of species. The average number of BGCs was calculated using the formula: Average number of BGCs = Total number of BGCs/Number of species. A new formula was developed in order to calculate gene cluster diversity percentage and is described in Section 2.4.

## 4. Conclusions

Research on harnessing the biotechnological potential of cyanobacterial species is gaining momentum. In this direction, this study is an attempt to provide a complete picture of P450 enzymes in different cyanobacterial species as these enzymes are the key players in primary and secondary metabolism of organisms, including the production of different secondary metabolites. Furthermore, providing names for P450s as per International P450 Nomenclature Committee rules enables researchers to make use of the cyanobacterial species P450 names presented in the study. A limited amount of cyanobacterial species P450s functional analysis revealed that cyanobacterial species P450s are unique in terms of their catalytic activity and they show high resemblance to eukaryotic P450s. Unravelling the role of P450s in carotenoid synthesis is necessary to understand their biological relevance in cyanobacterial species and also to address the evolutionary link between these species and plants since cyanobacterial species are considered as precursors of chloroplasts in plants. The mathematical formula presented in this study will enable researchers to conduct accurate comparison of secondary metabolite biosynthetic gene cluster diversity among different organisms. The highest gene cluster diversity observed for cyanobacterial species compared to species belonging to the genera *Bacillus* and *Mycobacterium* and the fact that a large number of biosynthetic gene clusters have no similar known clusters indicate that these gene clusters might encode novel secondary metabolites with new biological properties whose potential needs to be explored for the food, cosmetic and pharmaceutical industries.

## Figures and Tables

**Table 1:** Some of the cyanobacterial species well-known characteristics.

Species Name	Well Known for	Reference(s)
*Acaryochloris marina*	Species were isolated from the marine environment and can produce chlorophyll d as primary photosynthetic pigment that is able to use far-red light for photosynthesis.	[18]
*Anabaena* sp. WA102	Filamentous nitrogen-fixing cyanobacterium that often form blooms in eutrophic water bodies and able to produce a range of neurotoxic secondary metabolites.	[19]
*Synechocystis* sp. PCC 6803	Species found in fresh water and capable of both phototrophic and heterotrophic growth; owing to this ability, it was one of the most highly studied cyanobacterium for these characteristics. This species lost its nitrogen-fixing ability.	[20]
*Synechococcus elongatus* PCC 6301	Unicellular, rod-shaped, fresh-water living, obligate photoautotrophic organism that has long been used as a model organism for photosynthesis research.	[21]
*Synechococcus* sp. WH8102	Widely found in marine water across the world. It is well known for its oligotrophic nature, as it can utilize nitrogen and phosphorus sources. It also developed strategies to conserve limited iron stores by using nickel and cobalt in some enzymes. Species belonging to the genus *Synchococcus* are considered generalist compared to *Prochlorococcus* species, as they are nutritionally versatile and adapted to different ecological niches. These species developed a unique type of swimming motility, as they propel in the absence of any demonstrable external organelle.	[22]
*Thermosynechococcus elongates*	This species is unique among cyanobacterial species, as it grows in hot springs and has an optimal growth temperature of 55 °C.	[23]
*Cyanobium* sp. NIES-981	This species is used for standard inhibition tests for toxicants in water, as it fulfills the criteria provided by the Organization for Economic Co-operation and Development test guidelines.	[24]
*Dactylococcopsis salina*	Gas-vacuolate cyanobacterium isolated from Solar Lake, a stratified heliothermal saline pool in Sinai.	[25]
*Chamaesiphon minutus*	It is an epiphyte of fresh water red alga *Paralemanea catenata* (Rhodophyta).	[26]
*Leptolyngbya* sp. NIES-3755	Species belonging to this genus are found in various environments ranging from soil and fresh water to hypogean sites. This species was isolated from the soil at the Toyohashi University of Technology, Japan.	[27,28,29]
*Halomicronema hongdechloris*	It is the first cyanobacterium to be identified that produces chlorophyll f and is isolated from a stromatolite in the World Heritage site of Shark Bay, Western Australia.	[30,31]
*Pseudanabaena* sp. ABRG5-3	It is a semifilamentous, non-heterocystous cyanobacterium isolated from a pond in Japan.	[32]
*Prochlorococcus marinus* subsp. *marinus* CCMP1375	Among species of the *Prochlorococcus* genus, this cyanobacterium is extreme as it can grow at very low light levels in the ocean. Species belonging to this genus are the smallest known oxygen-evolving autotrophs and dominate the tropical and subtropical oceanic phytoplankton community. Species in this genus are adapted to different light levels in the ocean.	[33]
*Geminocystis* sp. NIES-3709	Fresh water living cyanobacterium capable of accumulating large amounts of phycoerythrin, light-harvesting antenna proteins, compared to *Geminocystis* sp. NIES-3708.	[34]
*Microcystis aeruginosa*	Species belonging to this genus are the most representative of toxic bloom-forming cyanobacteria in eutrophic waters. *M. aeruginosa* is well-known for its toxicity by producing various toxic small polypeptides, including microcystin and cyanopeptolin.	[35]
*Cyanobacterium* sp. Strain HL-69	It is isolated from the magnesium sulfate-dominated hypersaline Hot Lake in northern Washington.	[36]
*Crocosphaera watsonii*	Nitrogen-fixing cyanobacterium found in oligotrophic oceans adapted to iron and phosphorus limitation.	[37]
*Crocosphaera subtropica*	Unicellular cyanobacteria capable of fixing atmospheric dinitrogen (diazotroph) in marine environments, like filamentous cyanobacterial species.	[38]
*Trichodesmium erythraeum*	Filamentous cyanobacterium known as the primary producer and supplier of new nitrogen through its ability to fix atmospheric dinitrogen (diazotroph) in tropical and subtropical oceans.	[39]
*Arthrospira* (*Spirulina*) *platensis*	Economically important cyanobacterium, an important source of nutrition and medicinal value. This species is consumed as a source of protein around the world.	[40]
*Planktothrix agardhii*	Cyanobacterium forming bloom in eutrophic water and capable of producing toxins.	[41]
*Moorea producens*	Prolific secondary metabolite producing filamentous tropical marine cyanobacterium. One-fifth of its genome is devoted to the production of secondary metabolites.	[42]
*Gloeobacter violaceus*	Ancient cyanobacterium that lacks thylakoid membranes.	[43]
*Nostoc* sp. PCC 7120	Filamentous cyanobacterium capable of fixing atmospheric dinitrogen (diazotroph).	[44]
*Nostoc punctiforme*	A facultative heterotroph symbiotic cyanobacterium capable of establishing symbiosis with *Anthoceros punctatus*.	[45]
*Nostoc azollae* 0708	A nitrogen-fixing endosymbiont of water fern *Azolla filiculoides* Lam.	[46]
*Anabaena* sp. strain 90	Hepatotoxic bloom-forming cyanobacterium with 5% of its genome devoted to synthesis of small peptides that are toxic to animals.	[47]
*Calothrix* strain 336/3	Industrially relevant cyanobacterium capable of producing higher levels of hydrogen (biofuel) compared to *N. punctiforme* PCC 73102 and *Nostoc* (*Anabaena*) sp. strain PCC 7120.	[48]
*Fischerella* sp. NIES-3754	Cyanobacterium isolated from hot spring in Japan with potential to have thermoresistant optogenetic tools.	[49]
*Nodularia spumigena* UHCC 0039	Cyanobacterium responsible for Baltic sea brackish water cyanobacterial blooms producing toxins.	[50]

**Table 2:** Comparative analysis of key features of P450s in different bacterial species.

	Cyanobacterial Species	*Bacillus* Species	Mycobacterial Species	*Streptomyces* Species
Total No. of Species Analyzed	114	128	60	48
No. of P450s	341	507	1784	1625
No. of Families	36	13	77	144
No. of Subfamilies	79	28	132	377
Dominant P450 family	CYP110	CYP107	CYP125	CYP107
No. of BGCs *	770	1098	898	1461
Types of BGCs	73	33	18	159
No. of P450s Part of BGCs	27	112	204	554
Average No. of P450s	3	4	30	34
P450 Diversity Percentage	0.09	0.02	0.07	0.18
Average No. of BGCs	7	9	15	30
Gene Cluster Diversity Percentage	0.08	0.02	0.03	0.23
Percentage of P450s Part of BGCs	8	22	11	34
Reference	This work	[54]	[55,59]	[55]

Note: * 103 cyanobacterial species gave results with anti-SMASH (antibiotics & Secondary Metabolite Analysis Shell). Eleven species genomes did not give results. Detailed information on gene clusters is presented in Table S2.

**Table 3:** Comparative analysis of P450s that are associated with secondary metabolites biosynthetic gene clusters (BGCs). Types of clusters, most similar known cluster and similarity were obtained by submitting individual P450 clusters to anti-SMASH (antibiotics & Secondary Metabolite Analysis Shell) [65]. Standard abbreviations representing type of clusters as indicated in anti-SMASH [65] were used in the table.

P450 Names	Type of Clusters	Most Similar Known Cluster	Similarity
CYP213A8	T3PKS	Xenocyloins	25%
CYP213A5	bacteriocin		
CYP213A6	T3PKS	Colicin V	2%
CYP110AH1	NRPS	Anabaenopeptin NZ 857/nostamide A	100%
CYP213A3	bacteriocin		
CYP120C2	T2PKS	Ambiguine	6%
CYP110K6	NRPS		
CYP120A21	bacteriocin		
CYP110Q3	NRPS, T1PKS	Hapalosin	40%
CYP110C17	terpene		
CYP110C29	NRPS, T1PKS	Nostophycin	27%
CYP1011G1	NRPS, T1PKS	Crocacin	23%
CYP110AP1	terpene		
CYP110AT1	NRPS, T1PKS	Hapalosin	40%
CYP110Q4	NRPS, T1PKS	Hapalosin	40%
CYP110C21	NRPS-like	Anacyclamide	14%
CYP197E3	NRPS, T1PKS	Cryptophycin	37%
CYP110AG1	terpene	Hectochlorin	25%
CYP110E29	terpene, thiopeptide, T1PKS, NRPS	Nostophycin	27%
CYP110E18	terpene, thiopeptide, T1PKS, NRPS	Nostophycin	27%
CYP110C21	terpene		
CYP110Q4	NRPS, T1PKS	Puwainaphycins	40%
CYP110AT1	NRPS, T1PKS	Puwainaphycins	40%
CYP120A13	ladderane		
CYP1185A1	lassopeptide, bacteriocin		
CYP110Q2	NRPS, T1PKS	Hapalosin	40%
CYP110C14	terpene	6,6′-oxybis(2,4-dibromophenol)	14%

## Supplementary Materials

Supplementary materials can be found at https://www.mdpi.com/1422-0067/21/2/656/s1.
