# Supplementary material for: Comprehensive Analyses of Cytochrome P450 Monooxygenases and Secondary Metabolite Biosynthetic Gene Clusters in Cyanobacteria
Source: Int J Mol Sci. 2020 Jan 19;21(2):656. doi: 10.3390/ijms21020656 (PMC7014017; doi:10.3390/ijms21020656)
Supplement: Supplementary file 1 [file ijms-21-00656-s001.zip › Khumalo et al. 2019 supplementary files/Supplementary Dataset 1.docx]

Article

Comprehensive analyses of cytochrome P450 monooxygenases and secondary metabolite biosynthetic gene clusters in *Cyanobacteria*

Makhosazana Jabulile Khumalo ^1^, Nomfundo Nzuza ^1^, Tiara Padayachee ^1^, Wanping Chen ^2^, Jae-Hyuk Yu ^3,4^, David R. Nelson ^5^*, and Khajamohiddin Syed ^1^*

^1^ Department of Biochemistry and Microbiology, Faculty of Science and Agriculture, University of Zululand, KwaDlangezwa 3886, South Africa; khosietens@gmail.com (M.J.K.); nomfundonzuza11@gmail.com (N.Z.); teez07padayachee@gmail.com (T.P.); khajamohiddinsyed@gmail.com (K.S.)

^2^ Department of Molecular Microbiology and Genetics, University of Göttingen, Göttingen 37077, Germany; chenwanping1@foxmail.com (W.C.)

^3^ Department of Bacteriology, University of Wisconsin-Madison, 3155 MSB, 1550 Linden Drive, Madison, WI 53706, USA; jyu1@wisc.edu (J-H.Y.)

^4^ Department of Systems Biotechnology, Konkuk University, Seoul 05029, Korea

^5^ Department of Microbiology, Immunology and Biochemistry, University of Tennessee Health

Science Center, Memphis, TN 38163, USA; dnelson@uthsc.edu (D.R.N.)

***** Correspondence: dnelson@uthsc.edu (D.R.N.) and khajamohiddinsyed@gmail.com (K.S.)

**Supplementary Dataset 1.** List of P450s, P450-fragments and P450 false positive identified in different cyanobacterial species. Each P450 is presented with its given name followed by protein ID in parenthesis and species name.

>CYP110K1(ABW25656.1)Acaryochloris marina

MTATAPTLAPTPSAPIPRIPVPTWLQTIRAIADPIPYLENAYAQYGDIFEGRSFGFPPFV

ILSHPDAIEQVFTTDPSLFDSVSGNQIISPITGDQGLLLLEGEAHKKRRKLVMPPFHGER

MRAYGDAMCAIATQVSQEWPQNQPFVMRSATQEITLRVILRTIFGIDNNERFQQLSQLLD

EMLHTFDTPLGSSHLFLTGLQKDLGPWSLWGKFLRRRQAINELLLAEIQERRQQPLGQDI

LSLLLAAQDEQGEPMSDAELRDELMTLLFAGHETTASALAWAFYWIHKVPEVRTKLLAEL

RDITPDTDPSEIAKLPYLSAVCSETLRIYPLVLFTFSRTLKQPLRVMGYDLPPGAMLTPC

IYLVHHREDLYPNPNAFRPERFLERQFSPYEYFPFGGSNRRCLGYAFALFEMKLVLATIL

RQTSLQLVSSKPIQPVRRGITFTPAGGVPMLATRLGG

>CYP2337A1(ABW25859.1)Acaryochloris marina

MDVISYSIVTPQEFLGKRQQLFGQNFAALGRIIVGEYNLAAKIIAEPQQREPYIGRFRCI

PDRFSKNFLLFLSDKEVKGDTTHKKARSAVLEVVFKPAFDNTESQEAKDLLNQLATIANQ

HGNPPALDDISDDIQRTVIQYIMLVLLEVKLSPQQLSSLKTLFFSSKPKESLLISRVKPL

APSANKLQEVKRLEGIALNLIENSPVMSRYVPTTENDLSRTELSTLLLEAIAIAGCLGSE

SLLRSLIAKVPRDLEIDVDNRHEVLRVVLETARRHSPVNNINTIAQTETEVTINGRKRKF

PKGTLFSANIGLANLDSTVFENPLDFNPHRDNLLAALSFNSVGEAKRRECPGRSIAEKMG

SDLLVALQCKTVKS

>CYP110B17(ABW25868.1) Acaryochloris marina

MQTIAAQPSTHKLPEGPSSSGLWQMLQWIGRPLGFMEACRQRYGDLFTLQLGQLGQIVFC

SNPVGIEEILTADPKKMDAGCNNSVLLPLLGNASVILLDGESHQRQRQLLMPPFHGERMR

AYGSSITQITKDIASPWQVNHPFDVREAMQAISLNVIVKTVFGVSNPERYQALQALLPQF

LDLTGSPAGASLLFLRGLQKDWGAWSPWGRFLRVRSQLDHLLYQQMDEHRSQSEMGSDIL

SLMLAATDEVGNPMTDIELRDELVTLLLAGHETTASALTWALYWIHFLPDVKEKLIAELA

TADLNDAKSIQRLPYLNAVCSETLRIYPIAPITSPRTTRMPLSLMGHNLPVGTTVAPCIY

LTHRSPELYPNPQAFQPERFLERQFSPYEYLPFGGSNRRCLGMTFALFEMKLVLSTLLTG

YQFQLEQPQPLKPVRRGVTLAPPSHFRMILTQRR

>CYP1976A1(ABW28553.1)Acaryochloris marina

MTGLSAGSLTAGLHNTLKQLEAKPRQWVESKLQSYSQTLFQRIFSKQTQAFSQIPGPQPL

FPIGNVWALQQGKPIWQVLGEQGQQYGGLSLFWILARPSLVLNDPDLIQQVLLTAQQQTN

PHSHEHRANQADFYKDIPRKALRPILTDSHPFVAKTAGSKWQHLVKNNPFNMTYFREWME

TQIQPLQVFIESRAEALVKQSETEMLPAYEVIQKLAFDGFSLATVGQVFPDHVFEQFNTL

CATGTDRMNRSSIANWMIPEEPKGKHYKEASRQWFQRFSEVLETTQADPAGGSLLDWVLK

RGGSAFTPEQMRNFCAGVYPGGAVSAPSGITSTLHLLFQHPQALSALQHDLDGLFKEPLT

LERLENCIPVEQVLRESLRLWPPVPFFTRNTVGATHLAGHEIPANTQIFINNWFLHRLSP

HWHDSDSFQPERWDTKTLAENDYGSDYFFPFGRGERACIGQDFAKFFMRLTLATLLAKVE

IEFGDQPHDQEFYFGVSVPRQLKARFILRS

>CYP1136A6(ABW29142.1)Acaryochloris marina

MSDVLVLETEQAPPTGTQLPPRQPKWTDTLAYITNPDQFCRHNLKEHGPIFRTSVFGGTT

IFVGSSRAVQMALNGDLRYTEIGLPQTTMTMFGEYSLFQRPDLHRQRKSALRAGLAGQAI

QGYIPKINDVIIQRLQTWPAQGEIALFPAVEQICFDVLVPLLLGVELDDGCFQGLPIPSK

AGLKQVYKTFFDGFYGLLPWRSSFTTFGRGLQARARLIKFMQAVIHRRQSEINLDPTADF

LSMMLVSQQTDPESVFSNTLIENQCLLQLWASHYEISGLVSSWIYQLGRHPEHFAHLRGE

QATIQAQSAQPLTSEQLKTTSFLDATLQETLRTLPPSSTANRRLTQSVVLDGVLYEKGWT

LIAEPRIAHILPEHFPQPDAFQPNRFLENANARDKYAYIPFGGGVHACLGAQLAMTIAKI

FGSHLLRQFEWQVQGTAPFVQFPLKKIKDSYPMQIARLQQP

>CYP120A27(ABW30117.1)Acaryochloris marina

MAASNPSKPLPLPPGSLGLPVIGESLQFFQDSDFVSKRQERHGAIFKTHILGRPTVMIYG

AEANQTILGNESKNFENSWPASTKALLGPLSLALQTGELHRNRRKLLAQAFKPRTLATYV

EIMGDIAQQYTQKWQQAETLTWYPELRSFTLDVACKLFIGQDNGCQTPLGQHFETWCAGL

FSLPISLPWTKFGKAKRCRDLLLQELGQTIQARQSMANPGNDTLGLLLQARDEQGQGLSQ

DELKDQILLMLFAGHETLTSAITSFCLLTAQHPEITARLRQEQQQWKSKEALTVDDLKQM

TYLEQVLTEVMRLIPPVAGVFRTVLQTCEMDGYQIPQGWSVQCSIGSTHQDQALYSNPKQ

FDPERFSPEHLAQQSTEQQRYGYVPFGGGIRECLGKEFARLEMKIFAAHLLQNYEWQLLP

NQALDLVVIPSPRPRDGLKVTFKALSE

>CYP110AE3(ABW30725.1)Acaryochloris marina

MQTLPSPKTPTPVQSTQWIFDPVGYMKTNFKRYGDLFEACVLWGNSAPLIMVNDPKAVQY

ILTHDTRKEFTSPGDVNKLLEPLVGKRNMMLLSGNQHQNRRQLVLPPFHGERLTVYGAII

QQISNDLITQWPLHESRNTREIMQKITMQVILKAVFGLHQGERYQQLEQLLRIRLDMTGS

PLASAIVFLPWLQKDYGSWSPGGRLLKIAAQTDELLFAEIAERRANPDPDRVDILSLLLA

ARDEDGNGLTDQELRDEMMGLLVAGHETTATALTWAMYWIHSLPEVKGKLMAELDAVTNP

NDPSEFLKLPYLTAVCNETLRIHPVAMLTFPRRVEMPMELCGYQLDPGMLIMGCIYLIHQ

REDLYPNPQQFRPERFLEKKFSPYEFLPFGGGVRRCVGSALAQYEMKIVLGTLLTQCDFA

LLNKQPVRPGRRGVTLGQKQSVQIQKKGLRSQQSLQTV

>CYP197X1(AM1_B0109)Acaryochloris marina

MLGTWKTGEIHDIHQDMSQLTVKVITKVLFGVDITQMALEIGDALESILLQYYHKAQTSF

LLPSWVPTPGNLRANRAIEYLNKLVIDIIEQRRESPQDDLLSSLLLAQDEDGSQLSMKEL

RDEVMTLLLAGHETTATALTWTLMLLAQNPEVAQKLASETQSVLEGRAPGITDLPRLPYT

EMVLKESMRLYPPAWALSREVAQDCMIGPYFLKRGTSVFFSQWVVHRDTRFFDNPEQFLP

ERWQDNLELNLPRCAYFPFGAGPRGCIGQAFSIMEATLILAMILKQNYFKLVPDQAIELL

PSITLRPKYGIKMILESSNL

>CYP120C4(AM1_B0302)Acaryochloris marina

MTLETLEYTEASLPPGSLGLPIVGESLEFIKNKNFISDHQKKYGNIFITSLFGTPIVFVG

GGDEVNFILSNENKFFQAFPVGNVKSLLGEYSLSLQTGETHLKRRKLILKAFSPRRLASY

QETIHAITQSYINKWAKTDSFKWYDELRNYTFDIASKYLISLDNGSETKLCEYFKSWSNG

LFAIAPPLPFTKTRKSLVDRKKLIKLVDEIIQDRLSSSQTYDDALFHLMDAELEDGEKLS

VEEVKHQILLLLFAGHETLTSSLCSLCRNLSLHPDILSRCKEEQSNIPHNPDINQSSMPY

LDKVLLESMRIVPSVVSGFRKVLKECEIGGYKVEKGWLVFYQIDFTHYNKETYPNPHSFN

PENFNPAEHKELVKSSNYIPFGGGVRECIGKDFAMLEMKIFASSLISQCDWELLPNQNLE

YDLVPLPRPKDGLITKLRIN

>CYP1007M1(AM1_E0096)Acaryochloris marina

MAWTVPGPKSTPIVGTTGNTFRFTQDPIGYSNQLFQDYGPMVSLCEQGGTNLFTPFEKCP

GTVIVHGPDLIRQARTPHSQWNLTPLSMTLYPMEAVSNRKQPLKHFLVGLWGVNGDEHRL

QRRLMMPAFHTRHIQSYWQDMVNITQSSLSHLRPGTTVNLVEETRLLAMRIVTKTLFGED

SGKAGQGVGHLLEQALAVLSQPLTTLMPYDIGGLPYRRFLNLIACFDREMRGIISAKRAR

QSKDNDVLSMLIQARDEETGISLSETELLGHVGSLFAAGHETTSLALGWTVLLLSQHPDI

AADLMDELGAVLQGDPPRLDQLDQLPLLERVIKESLRLIPPAPFTWRYNNQIVDLGGYSL

PVGTEVYCSIYATHHMPDLYENPNCFDPSRWERINPDMWEYMPFSAGSRLCMGAAFAMME

LKIVLSIMLQTFRMEFVPSVSVDRYGIITIAPKNGLPMHLHHPDRNFTAGVGGVKGNIRE

MVQLPA

>CYP197X2(AM1_F0120)Acaryochloris marina

MTANASQLPLHDEAHRISGLAALRELFTFARNPIEFVTKSAQEYGDTAILSLGSIEIFLF

HHPDLIAEVLNTQYQSFIKDVSYRSLSKVLGNGLVLSDGELWRRHRQLMTPAFSQERISA

YASIVVEETSHLLKHWKKGGILDIYQEMRQLTVKVIAKALFGIDVTQTALEIGDALEAIS

LQIYHRAQTNFLLPDWMPTKSNLRANRAIQYLNKIVISIIEQRYQSPKDDLLSTLLSVKD

EDGNQLSFEELRDEVMTLLLAGHDTTANALTWTIMLLAQHPTVANQLRKETQTELDGKIP

NITFLPRLAYSQKVIRESMRLYSPAWILTREAIQDCQIGPYRLKKGAGVVVSQWVVHRDP

RFFEEPEKFLPERWQDNFEQKLPRCTYFPFGAGPRVCIGKAFSMMEATLILAMIANQFHF

KLVPDQSIELLPSITLRPKQGIKMILD

>CYP110E16(AFZ57188.1)Anabaena cylindrica

MQLPGESQTSRFIQRLQWVFNPLQLMETSVKTHGEFFTLRLSGEQPIVFITNPQAIQEIF

TTPPEYFDSGRGNGLLKPLVGENSLLLIDGKTHQRQRRLLTPPFHGERIKSYAQIITNIT

KQVINDWEIGKPFSVRDSMQEISLKVILQAVFGLCDGERFRELEELVSSLLNLSGSPLRS

AVTFFPSLQVDLGAWSPWGMFLRQRKKIDQLLQTEIEERRNNFDSSRSDILSLMISARDE

NGEAMTDSELRDELMTLLFAGHETTASALTWALYWIHHLPTVREKLINELNSLAENADLN

EVSRLPYLTAVCQETLRIYPIAMITLPRIVKSPTTIMGHEFAPGILLAPCIYLTHRRPDL

YPEPEKFKPERFLERQFSQYEYIPFGGGNRTCIGMAFAMFEMKLVLATVLSQLDLALVDN

IPVKPMRRGVTLAPSGGKWLIPSGKKERVKSVVEV

>CYP110B6(AFZ57194.1)Anabaena cylindrica

MPLPISPKTPATLQMLRWVFTPMPYMEECAKTYGDIFALKLQKKLPPLVFIHSPEAMQQV

LSNDTKELEAPGDLNSIFEYLLGKRSVISLSGAEHQRQRQLMMPPLHGERMRNYADLMGD

ITETTMNKQQLNQPFNVRTVTQDITLSVMMQAVFGLYEGERAEKLQNLLCEILEVGSAVW

RVAVLYFPALKDVIGLSQLWEKQQRQQDKADQLIYEEIQERRDNPDQSRTDILSLLMAAR

DEAGQPMTDVELRDELITLLVAGHETTATAIAWALYWIHKLPEVRQKLLSEIDGLGGNLD

SNAIFKLPYLTAVCNETLRIYPIGMLTFPRRVKTPISVCGYELEAGTVIMGSIYLTHNRE

DIYPNPKKFNPERFLEKQFSPYEFLPFGAGARRCIGLAFAQFEMKLVLAKILSGWQLELA

>CYP1336A1(AFZ58759.1)Anabaena cylindrica

MLTEILQQGEKYDLLNPLFFANPDATLHQMRTEDPIYWHSQLESWILTRYVDIYNVIRDP

RFSVDRGGKIGKSKSLAVKTKLDFCNQYFTQWMVFSDPPRHTRLRNLVGKAFTPQLIESL

HPLIQNFADELIDAIQDSGKMEVIHDFAVPLPALVTAKLLGVPQEDIPLLKRWSSNMFML

FGAGWASEEVVETTYQSLVDSIQYFDALIAEYRQSPSDNLINQLIAAEDHGSVLSDEELT

ATCITFMAGAYETTTYLISNGLLALLQNPEQLQMLQENPLLIDSTVEEILRYCGPAFSVV

RRAIADIEINGQLITNGQKIYCVLHAANHDPAKFPNPDKFEITRKENRHLGLGQGIHVCL

GAALTRLETKIAINTILQRLQNIRLDTEQLTWIPNLAMRGLEELPIVFSLKGIPNKKIFN

>CYP2287A2(AFW97023.1)Anabaena sp. 90

MQPTLLDKALNHFQAWAIENPQNPVSRTFLPIISFVEGTIANNPPYLDRKRQVLGDAFCC

AGEVVLGDFKTLETALTSPQARDWRLGTSMLSSSHGPGVDQGGRNVLLITLSDAAPGGEN

HHAFRKCVDNYFFNEGMVMRQNDQVAQRLFNKLAADYVDRITNNTLEEFFNNDQRDWMGF

LVRYLHYVIFGINPDDQATIRIITTFHYTKRGTLYYFAGSSSLEKLNIWGFRQVPELVEQ

VATIYENSPALANFQENDPKYHKMTRRELAKLMVSLMSIAGLQGPLHLGKTALGYQSLPA

YQGRQTDKIDIKSYWDRLDLNDRPSIQLFLLECARLFMPVSASHRVATEPFRVTLAGKAR

TFPKGTKILIPMLLGMLSEDFWGKTAYEFNADRENLCPFHMGFNSVGSRHAGRICPGKDL

ALEMLTNVIISVGKARRSYLSQIS

>CYP110E14(ANA_C11544)Anabaena sp. 90

MSNLKLPDGPKTHPWIQTYQWLKDPLGYMEECAKNYGDIFTLRVGPVFTPQIFISNPQGI

QQIFTTDTKQLDSGEPAGVQAPLLGRQSLLALEGKPHQRQRKLLTPPLHGERMLAYGDLI

SEITQQVTNKWQIGQTFNILYSMQEISFEVILKAVFGLQNGSHYEQLKTFLLKILNPKQP

FLRAMMLVFPFLQKDLGAWSPWHKFLQLKAEIDEVIYAEIKERQEKPDSSRSDILSLMMA

ARDENGQPMSDVELRDELITLLIAGHETTATSLTWALYWINHVPQVRAKLLQELDNLGEN

PDANEIFKLPYLNAVCSETLRIYPVAMLALNRLVKSPFELMGYNLEAGTIVIPCIYLTHH

REDLYPDSQQFKPERFLERQFSPTEFVPFGGGNRRCIGMAFALFEMKLVLANVLSNWNLE

LADTQPVQPARKGALLGPCGGVKMIVKGKREKNQPILQKAR

>CYP110B11(ALB40509.1)Anabaena sp. WA102

MQLLPGPKVSATIQVLNWIFRPMSYLTECAKTYGDLFTLKLESNLPPILFVHSPEAIQQI

LSNDHKDLEAPGELNSIFEYLLGKKSVISLSGKEHQRQRQLIMPPFHGERMRSYAELIEN

ITTKTINKQPKNQPFNIRNVFQEITLQIMMEAVFGIYEGEQAENLRHLLCEIIEQSSVPW

RVAFLYFPKLKEVIGISELWKQQIKKQEKADQLIYQEIQQRRENFDPQRTDILTLLMSSR

DENGEPMTDEELRDELMTLLFAGHETTATAISWAFYWIHKLPEVREKLLVELDSLGENPD

SNTIFKLPYLTAVCNETLRIYPIAMLTFPRKVKTPISLCGYQLEAGTIIMGSIYLTHQRE

DIYPQPEKFNPERFLEKQFSPYEFLPFGGGARRCIGLAFAQMEMKLILAKVLKTWSMKLV

NTDEIKPQRRGLVTGPNGPINLEIQNLRQPKYVNLETVTV

>CYP110E13(ALB42404.1)Anabaena sp. WA102

MSNLKLPNGPNTHPWIQTFQWVKNPLGYLEECAKNYGDTFTLRIGPLFKPQVFISNPQGI

QQIFTTDPKQLDSGTPAGSGVPLLGPNSLLSLEGKTHQRQRKLLTPQFHGERMLNYGELI

NNITQQVTSKWQIGEVFPILSSMQEISFQVILKAVFGLKDGERYDKLKQNLIAILNPKRP

LFRVAMIMFPFLRRDLGAWSPWGRFLRLRQELDDIIYAEIHERQLQPDPSRTDILSLMMA

ARDEVGEAMTDIELRDELMTLLVAGHETTASSLAFALYWIHSQSGVREKLLAELDSLGET

PDANAIFKLPYLNAVCSETLRLYPVAMLGLNRLVKSPLEIGGEKFEPGTIVVPCIYLTHH

RQDLYPDSQQFKPERFLERQFSPSEYLPFGGGNRRCIGMAFALFEMKIVLAKVLSNWHLK

LAETEPVKPVRKGLLLGPGGGVKMILKEKRRQNQPVI

>CYP2287A2(ALB42484.1)Anabaena sp. WA102

MQPTTRPETNLQPSLLDKALNHFQAWAIENPQNPVSRTFLPIISFVEGTIANNPPYLDRK

RQVLGEAFCCAGEVVLGDFKTLETALTSPQARDWRLGTSMLSSSHGPGVDQGGRNVLLIT

LSDAAAGGENHDAFRKCVDNYFFNEEMVIRQNDQVAQRLLNKLAADYVDRITNNSLEEFF

NNDQRDWMGFLVRYLHYVIFGINPDDQAIIRIITTLHYTKRGTLYYFAGSSSLEKLNIWG

FRQVPELVEQVATIYENSPALANFQENDPKYHKMTRRELAKLMVSLMSIAGLQGPLHLGK

TALGYQSLPAYQGRQTDKIDVKNYWDRLDLNDRPSIQLFLLECARLFMPVSASHRVATEP

FTVTLAGKARTFPKGTKILIPMLLGMLSEDFWGKTAYEFNADRENLCPFHMGFNSVGSRH

AGRICPGKDLALEMLTNVIITVGKARRSYLSQIS

>CYP110M3(BAI88901.1)Arthrospira platensis

MKLPDGPRLMSPLQILQLVIDPPKYLENCADKYGDRFTLRVLGNNSPPVVFFSHPQAIEA

IFAGSEGLCPADQLELGRITHVFEPLTGPQSLIAKDGSEHQRLRQLLMPPLHGKQLPRYG

EAIANIADSVTENWSVGDCISIRKYTARMSLQTILQVVLGLGPGYLMNQLQQTLETYLEW

VNSPLNSLQFFWPALQKDLGAASPWGKFLRQRQQIDDLIYTAIGDRRREATGEDVLSLLI

SARDENGEGMSDQELRDQIITLLLLGYDTTASATAWVFYWIHKFPHIRDQLVAELQTLGS

DSDPAEAVQLPYLTAVCREALRLHPIALISQPRVVRESVTIGGDRFQPGTVLVPCIHLAH

RRSQTYPEPEKFRPERFLERRFSPYEYFPFGGGSRSCIGMALSLFEIKIIVATVLSRCQF

SLADLGPVRPVRRGITIVPSDNFKLIVENIRPSLISSVV

>CYP110C22(BAI92008.1)Arthrospira platensis

MKLIDGPQNLPTLQKIQWITNPLGYMENCQRKYGDIFVTEIGVAGHPIVFVSHPQAIQEI

LTRDKEFTAPGSSNRILAPVVGDKSVILLDGKDHLRQRKLLMPPFHGERMFNYGKLICNL

TTEIGDTLPIGKTFLARDYVQKISLAVILKAVFGVYEGPRYDRLKKLTTELVAVTDSPIR

ASILFFPILQQDLGRWSPWGRFQGLRRDIDKLLLEEIEERRQNPDPNRTDILNLMMSARD

ENGEPMTYEELRDELLTLLFAGHETTATAMAWSLYWVHKLPTVRDRLLSELATLPPNPDP

MTIYRLPYLTAVCQETLRIYPVAMVTFPRVTTQAVELMGYHIEPNIEVIGSIYLTHNDPT

IYPEPKQFKPERFLDKKYSPYQYLPFGGGVRQCIGMALAQYELKLVLATVMLNYQLTLAN

SQPAKAARRGVTLSATGGIPMVLTGRRSLSPNSVGVAPKETPLLG

>CYP110AT1(AKG21609.1)Calothrix sp. 336/3

MNLPQGPKLPAWWLRMQFTADPLRFLDQMNQRYGDTFTIMSESTPLIFVGNPQGMKQVFT

NTTEIIASGELNAESAPLVGQNGLLLLDGIRHRSRRKLLMSPLHGNKIRAYGQQICDVTE

KVMNQLPLGKSFLAYPTMQKITLEVILNTLFGLYTGDRYEQLKHLLNNLMNYARSPFVEI

PLSFPFLQQDLGRWSPWGYFRYLWRQFDKLIYAEISDRRQQPNPDANDVLSELIFARNET

GELLTNEEIRDLFPSLLFGGRDASATAITWALYWLHREPQVREKLRQEINNLGESPNPMS

IVELPYLNAVCNEILRIYPTQFVTFPRIVESATNFMGYNLSPETIIIGCIYLTHQNPELY

PQPQQFRPERFLEKQYSPYEFMPFGGGARRCPGEALAMFEMKLVLAKIISRYQLSLTHTQ

REKPQTQGANYPPASKLQMVLSNIQQHQQSPQEAVHF

>CYP110Q4(AKG21610.1)Calothrix sp. 336/3

MKIPEVNIPLWLNIIKIANNPLNHLDAIAKRYGNIFIINFGETPIVFVSEPQAIKQIFTN

TKDIKTPGELNAEAALITGNQGILQLDGLRHKHRRKLMMPAFHGVKMQAQGKLICNLTKQ

VMDEYLASKTFIAYPTIEEITLQISMKVVLGLQEGERYEKLKYLLPTILKSMRSPIMQTT

SFLPFLQKDLGKWSPWGYLLHLRQEIFELLYAEVQERRQQADTSRSDILSELIFARDETG

ELMTDEEVRDLLLSPLFAAQDASAVAITWLLYWVYRQPQVREQLLQELATLGDSPDPMNI

AQLPYLSAVCNEVLRIYPAQLFTFPRRVESPIELMGYDLAPGTLIMGCIYLTHQHPDLYP

NPQQFQPERFLEKQYTPYEFFPFGGGARSCIGAALAIFEMKLILATILSNYELALVKKQP

EQPKFEGLMCYPASGVKMHISTKTPRPSAETSAPLCV

>CYP110B16(AKG21668.1)Calothrix sp. 336/3

MALPNPAKIPTFIQIVRWFVSPLSLLEECAKNYGDIFTLSISPQLTPVVIVSNPIALQQI

LTGDSSGNFTAPGYQDATFGCLFGPFSLMSIDSTEHQRQRQLLTPAFHGDRMRSYAAEIN

NITEATMSSLETGASFSIRDVTQAISLRIIIKLVFGFYEDESAKQMENLLNQMLNNISSP

GGAAMLYLRFLQKDWGNWSPWGKHIKFQQEIDNLLYSEIQERRKQADTSRTDILSLMMSA

KDENGESMSDQELRDELFTLLIAGQETTATALAWSLYWIHKLPEVREKLLLEIDSLYENS

SENLDINTINKLPYLNAVCCETLRIYPVGMQTFIRRVEKPSQICGYDLQPGTLLYGNIYL

THQRQDLYPEPQKFRPERFLEKQFSPYEFLPFGGGVRRCIGAAFALFEMKVVLVKILSTY

SLVLVDDVDVKPKRRVLVTAPSSPINMLITDKRNR

>CYP110E28(AKG24605.1)Calothrix sp. 336/3

MKGILRPIYYLETTRKSLGDIFIAEFASFPDQVIISDPEAIQKIFTADAKLFLSGSGNEV

IQPLVGTNSLILLDGDRHIQQRKLLMPPFHGERMRAYGQLFRQITQQVMDKLTPGETFIA

REKMQEISLQVILRTVFGLQEGERYQQIQTLLTEMLDSFESPISAVFLLLKSLQIDLGSL

SPWGKFIRKRAILDKLLYQEIKERRQQNQSLGEDILSLLLSVQDENGQFMSDTELRDELM

TMLFAGHETTATAIAWALYWLHYLPEVKEKLLNELHSVDLENIDPMELAKLPYLNAVCSE

TLRIYPVVFFAFPRILQAPMQLLDYQFPQGTMFSICIYLTHHRSDIYPEPEKFLPERFLE

KQFSPYEFLPFGGGNRRCLGMAFASLEMKIVLATILSRYKLTLQESQPLKPVRRGLTFTP

AGGVHLALQEKSSL

>CYP110B2(AKG24327.1)Calothrix sp. 336/3

MKLPQGPQTPAIVQMLQWIARPMPYMEECAKKYGDIFTLHLSFPILFVSHPQAIQQILTS

DNKDFTAPSRANQLFAPLLGKNSVITLEGEPHFRQRQLITPPFHGERMKTYTEVITKITE

SVMGQWRIGQSVDARAAMQAITMRVIMQAVFGLHEGERAEELERLLAIMLEEGGSSPLRA

ALLYIPILQQDLGTWYPWGRFLRRRQRVNELIYAEIRERRAQADTSRTDILSLLMAAQDE

NGVGMTDEELRDELMTLLTAGHETTATALAWALYWIHKFPEVKEKLLAELDTVPDKSDLN

TLLKLPYLNAVCNETLRIYPVAMLTFPRSVKNTLTLSGHELQPGTLVMGAIYLTHQREDL

YPNPKQFRPERFLERQYSPYEFVPFGGGARRCVGTAFAQLEMKLVLATILSSLNLDLATK

GDILPRRRGLVTGPDRVIELVVQGQRQVKTSLLEKVSS

>CYP120A10(AFY99987.1)Calothrix sp. PCC 6303

MSSTKNLPLPPGKLGLPLLGETIDFLRDRSFSQKRQNWYGNLYKTHLFGRPTIVVIGADA

NRFLLTNENTYFTSSFPKTTKELLGAASLAIQKGGEHLQRRKLLSQAFQPRALSGYVGGM

EAITHGYLQKWEKIGDLTWYPELKKYTFDVACKLLIGTDTASDDEIGQFFEEFSEGLFSL

PINLPWTKFGKALHAREQLLMKIEQIILQSQQQTASSQDALGLLLQATDEDGSKLSIKEL

KDQVLLLLFAGHETLTSAIASFCLLLAQHPDVMAKVRTEQQNFDFSQPLTLESLKEMTYL

EQVLKEVMRVIPPVGGGFREVIQTCEFNGYQIPQGWAVLYQVNRTHRDESIYTEPDSFEP

ERFNPDRAEDKTKPFSYMTFGGGVRECLGKEFAKLEMKIFAALLIRKYQWELVNGDKPEM

AMVPTPRPRDNLQVRFGCLSK

>CYP110C12(AFZ00287.1)Calothrix sp. PCC 6303

MKPLNPVKSHPFLQKIKWVIDPVGYMENAVAENADIFSTQIVGFGDGLIFVNNPQAIQEI

LTNDRKKYNAPGKLNGILRPLLGDYSTIMIDGDRHRQRRQLVMPSFHGDRMKSYGELIVN

LTKKVMDELPINQPFLAREKMQEISMQVIIESVFGLHEGERCQEIKGLMEEMSQLFRSPL

SSSALFFHSLQKDLGAWSPWGKFVRDRAKLDELLYAEIAQRRANPDSSRIDILELLMSAK

DENGEGLNDQELRDELMTLLFAGHETTATAMSWGLYWSHHLPETRQKILQELSQLSNFGD

TMSIFRLPYLTAFCNETLRIYPVAMLTFPRVAQEDTEILGHQIQKGATVIGCMYLIHQRE

DLYPQPKQFKPERFLERQFSPYEFIPFGGGSRRCIGEALALFEMKLVLATILSNYQLELV

DSQPEKPRRRGVTLSPAKGVKMMLKGKNASINLSKQAVTV

>CYP227A4(AFZ02566.1)Calothrix sp. PCC 6303

MALKQQVIKGHQRQIWLAKILAKVGNNSPIGKLLRLGFYYADGIVILRHWRDFLYIRKDN

IGDTYLAVDQAIMHSSHTQVKELIHTEPQLRGNDLGIIRILAPSYLLNNPLSLGMNGNQH

QGARALFSQALPNPSEQADILGELVHQYLADAAKQKQLHIGNDLPAIMLQILHRVVFGIS

LSDEEINGSRNFIKGLPLASLPNFISEKVIASKTAPSIKHRKNLIKRYQQSPKWADYLET

GSRYQLNPHQIANTLFDMIHIAGTAGTSALLGSVIGVLCQDNALRNDVVAEINTVWDGSE

ALKEDNIEQLILIRNTILETARLYPPVRFVSQLARESGEIEITASKCPFQKGTRLLGSIF

TANRDPRRYDNPDNFDTNRDFSDLLSWNGKGHERTCPGKDLSIALIQIFCLYLFKKYQWN

STTAVKWDFEKVTSVTPNDLVLQGFSSKL

>CYP284A4(AFZ02903.1)Calothrix sp. PCC 6303

MFQEITGQIAYSSSFAYLATTLSITSIAGTIGWRWWKQKKTYKSLQSLPSPPQHWLLGNI

PQMLAAVKQKKLFRLFFDWSQELGPMYVIWNGSHPTVVLSKPKVIEDTIVNGMRDGSLIR

TQRLRQAWNDIIGPIMIGETGTEWQWRRKAWNPEFSSSSLSKYAEFISQACEQVIETLKE

ASPPKEVQVDPLFVELTMRVISSLVLGIPVDKNSTSNEGPPLEVMKMYEAMSVVGYRFLR

QATGEKRWMKYLPTKNSQDYWAARRYLEEFITPRVDLALRISQQNKTDLPQVSSLFQESM

LVKIAAKEAKYDRQTLIAESVELLIAGTDTTAHSLSFAVAELSLNPRVFQQAQDIVDQTW

NNFGGINAESLKELAYIRAIIKETLRLYSVASGSTSMQTQRDTVIDGKEIPRGTGVSWSM

LAAGRDPEVYANPEEFLPERWLDKSKETSPLPMIDFGSGLHRCLGEHLSMLESTTMLALL

LRYFDWELVNGRSSVEQLQQNLLIYPSDKMPVRFRLRNWE

>CYP110B2(AFZ03925.1)Calothrix sp. PCC 6303

MALPNPVKIPTFVQILRWFVSPLSLLEECTKNYGDIFTLSVSPQLTPVVIVSNPVALQQI

LTGDSSGNFSAPGYKNSTFGSIFGPSSLISISSTEHQRQRQLLTPAFHGDRMRSYAGTIR

DITEGVMGTLETDKSFSTRDITQAISLRTIVKLVFGFYEGESAKQMENLLNEMLNNISSP

GGAAMLYLRFLQRDWGSWSPWNKHIKLQQEIDNLLYSEIKQRRKQADTSKSDILSLMMSA

RDEAGESMSDQELRDELLTLLIAGQETTATALAWALYWIHKLPAVREKLLAEIDSLYENS

SDNSDINAINKLPYLNAVCCETLRIYPVAMQTFIRRVEKPLQISGYDLQPGTLLYGSVYL

THQRQDLYPEPKQFRPERFLEKQFSPYEFLPFGGGVRRCIGAAFALFEMKVVLVKILSTY

SLNLVDNIEVQPKRRGLVTAPASPINMVITGRRNSSNINSLSQDSLSARS

>CYP110A3(AFY30606.1)Calothrix sp. PCC 7507

MLNQLPNCITSPPWWQLINWIADPIAFQHKYSQKHGDIFSMHLSGVGSYVIIGNPQAIQE

IFNQDSKFDIGRGNELAKPLLGQNSLMLMDGDRHRRERKLLMPPFHGERLQTYAQQICLI

TNQITSQWQIGQPFIARSTMQKVSLEVILQIVFGLSAGERYQQLKRLLTAWLDMTDSPLR

SSMLFFQFLQKDWGSWTPWGQMKQRQGQIHRLLQAEIAEKSTKNDEKGADVLSLMMAARD

ENGQAMSDAELTDELLTILFAGHETTATTLAWAFYQIYQHPDVLEKLQQELDSLGDNPHP

MEIAQLPYLTAVSQETLRMYPVIPGLFPRITKSSTKIAGYEFDAETTLMPSIYLVHYRED

LYPNAEQFKPERFLGRQYSPSEYLPFGGGSRRCLGYALAQLEIKLVLATVLSKYQLALAE

DKPVKLQRRGFALAPAGGVRMVMIGKR

>CYP110D12(AFY32065.1)Calothrix sp. PCC 7507

MTANNNLPDGPRMQRWLRLVKFITQPVKYVEDFAKIYGDAFTVWGSRGTHMVYFSQPQAL

EQIFSADPSHFEIGRGNGGLRFLLGDNSLILLDGDRHKRQRQLLSPPFHGDRMRAYGQDI

SEITQQISDEWNIGKPFNIRRSMQEITLRVILRVVFGLNEGERFQELQQLLTSVLDQIGS

PLTTMTLFFGFLQKDLGVWSPWGKIMYLLQQIDQLIYTLIAERRLESNQNRQDILSLMMS

ARYDDGQPMSDVELRDELMTLLVAGHETTASALTWAFYWIDQLPEVREKLLKELESLGVN

PDPSNIAKLPYLTAVCQETLRIYPIVMNGVARIVKSPIEIMGYNLPTGTIIIPSIYLAHH

REEVYPNSQQFRPERFLERQFSPYEYLPFGGGNRRCIGLAFAQYEMKIVLAKILSHFQIS

LANQHPVRPVRRGFTLATPAGMQMIPKLKVKRATQPVAV

>CYP1156A1(Cal7507_3320)Calothrix sp. PCC 7507

MHTPITPLIPPEHLGLNGEDNQSGLAKRHQSLTYDLFAPEVIANPYPLYKRIREEDPVHY

EASLGYWILTRYQDVEAALRDERLSANRRTLFIQQLGNLDVSLIQNFLQLTGNSMIEKDP

PDHSRMRKLANQGFTTRALESWRSIIQKTTDTLLDQVQHQGYMDIVSDLSVPLPSLIIAK

IFGVPESDRANLIQWATDTSSFWGAPSGKNIEELAQKADTSAAQFTAFINQLVAERRQNP

GTDMISLLTMAYTEQNLDFAELPSLCILILNAGRSTTTDLIPNGVSALLNHPQQLQKLKD

NPALISSAIEEIIRYDSSVSFVFRIAKEDIEIGGQKIPAGSVIALGLAAANHDPAKFPLP

DNFDIERSPNEHLGFGPGIHFCLGAVLARMELNICFSTLLKRFPNLQFDSTRPPLPRRST

LVFKGFDALPVRF

>CYP110B7(Cal7507_3762)Calothrix sp. PCC 7507

MKLPKGPQSPALVQMLQWIFRPMPFMAECAERYGDIFTMQLNGPVVFTCNPQALQQMLTS

DTKEFTAPRDWNMVFEPMLGKNSLITVDGEVHRRQRQLLMPPLHGDRMRTYGQVINDVTE

QVISQWQIDKPFCVRSAMQAITMRVIMQAVFGLYEGPRAEELETVLATMLNESSPFRVIE

LYFPALRQDFGPRSSWGRFIRRRQRVHQLLNEEIQERRDNPDESRTDILNLLMAARDEDG

QPMTDEELRDELMTLLTAGHETTATALTWALYWIHKSPSVQEKLLAELDNLSDRSDSTSI

LKLPYLNAVYSETLRIYPVGMLTFPRVVKTPVSLCGYELAPGTVVLGSIYLTHQREDLYP

QHQQFKPERFLERQFSPYEFLPFGGGVRRCIGMAFAQFEMKVVLAKILSSWDLALVDHRD

VQPKRRGLVTGPDRSIQMVVKSQHQPKSRTLVHPQGDSHNN

>CYP110C21(Cal7507_4736)Calothrix sp. PCC 7507

MQLPNRLKIPSFLQKLYWVVDPVGYMEKAVQQHPDSFTAEIIGFGGNVVFVNHPQAIQEI

LTNDRKKFAAIGELSRILEPLIGEYSVLLLNGNHHKRQRQLMMPSFHGERLDIYGQLIRN

MTEEVCNQLPFDKPLSALDVTQNISMEVILKVVFGLSEGERYRKLKDLVPKFVSEIFSSP

LKSSFLFFPFLQQNLGSWSPWGRFIRQRQQIDELLYSEIAERREKNDPERVDILSLLMSA

KDEEGQPMTDPELRDELMTLLIAGHETTATAMAWGLYWIHHVPKVREKLLQELDTIGDLK

DPMNIFRLPYLGAVCNETLRISPVTIFTLPRAVQEPVTLLGHPLEAGTVVQACIYLTHQR

EDLYPQPKQFKPERFLEKQFSPYEFLPFGGGVRRCIGDALAQFEMKMVLATILSSYQLSL

AGRQPERLQRRGIVLGPANGVKILVKGRRARQESLVTAANALSS

>CYP110B12(AFY93206.1)Chamaesiphon minutus

MYWESIMQLPLGPQTPAFVQLTRWIFSPMSFMDECVRRFGDMYTVNLGKNNPNLVFVSNP

QALQQMLTQDTRADFHAPGDRNGLLKTLLGDNSVICLSGSQHQRQRQLMMPPFHSDRMRT

YSQVINQVTTETLARVQIGQTFDVRKTTQAITLRVIMQAVFGLNRGERAERLAHLLSQLL

DRSGSPLSVSMLYFPILQQEWGGISPWAIQMRVQRAVDALIYAEIAERRTHPDSSRTDIL

SLLMAAQDEAGESMTDVELRDELMTLLVAGHETTATALAWALYWLHKLPAVRDKLVAELD

GLGAEPDVNAIIKAPYLEAVCNETLRIYPVGMLTLPRVSLKSVTLAGREIPPETTILGSI

YSTHQRADLYPQPQEFRPERFLERKFSTFEFLPFGSGARRCIGSAFAMMELKLALAKILS

RWELELVDKRDIHPKRRGLVTAPERPIQLLVKSQRSASDLHPAQAIDRGAFA

>CYP120B12(AFY93961.1)Chamaesiphon minutus

MSIARQAATLPLPPGRTGLPWIGETLSFLLDPDFATKRRQEHGAIFRTHIIGRPTVVMSG

AAANKFILSTHFDKFSWRDGWPDNFKELLGASLFLQEGVEHQRNRRLLMPAFHGKALTNY

VSTMNEITDRYLAKWSQTGNLTWFPELKNLTFEIASVLLIGSEPGAETIELSRLFTELTN

GLFTIPLNWSYTTYGRAVAARDVLLAHIEKVVLERQQAPTQDALGLLVQSQDEEGNRLSV

AELKVQALLLLFAGHETTTSLVSSLCLALARHPEILATTRAEQVQIGLDAPITIDSLKQM

TYLDRVLREVERMYPPVGGGFRGVTEEFEFNGYRVPKGWQVLYRIPEAHYDAEIYPEPDT

FDPDRFAPERYEYKPSDYNFATYGGGSRICIGMAFAQMELKIIAAKLLRHYSWELLPNQN

LTLDPIPTLHPRDGLKVKFEKLDMSA

>CYP120C4(AFY94075.1)Chamaesiphon minutus

MSAEQKFMVMNNNLKSADLMPGSYGLPIIGELISFLTKREEFYWQKHQANGNIFKSSSLI

FGKTACLAGPDANRLVFKDAADKLSSRLGNKLLEPISADSVLLKDGEAHRTNRKLILPVF

HQQAIAGYFDTIQSVVTEAIADWGERGTIDLSAELHKLTLTVVVKIFLGSESTAEVQQVS

EWFNTLVDSVSGVIRWDSPLTSYGRGQAARRKIADYILQVIAERAARGDLDRSTDVLSLL

VNTIDEDGNKFTETQIIDQVMAFLFAGHDTTSTLMSWLLFELGNSPAWRQRLRDEQQQVM

GTEPISMTHLRQLPDMTNVIKEGERLYPPAFLIARVALADIEYAGYLIPAGWFIFISPML

THRLPEIYQNPDTFDPDRFAPPREEDKKYPYSLIGFGGGAHICIGVELAQMEMKIILSTL

LQKYDWTVTPTTAEIAPVRRPFTMQKRLKATLVPLRSP

>CYP120C3(AFY94077.1)Chamaesiphon minutus

MTDRLKSAEMMPGSFGLPIFGEIINIISKQELFYWQQHQQHGNVFKTAALGFGKAACLVG

PDANRLVLKEQADRLSSRLGNQYLAPIVSTDAVLLQDGEQHRTSRKMILPVFHQQALAKP

LQGRIAAYFDTVQSVVTAAVADWGERGTIDLDAELRELTLAVVVKTFLGSEKTAEIQQVS

EWFTTLTGGLYGLFKWDTPLTLHGRGQAARRKIVKYIRQVIRERVDRNDPANALDVLGLL

MNTVDEDGNKFTETQIVNQAILFLFAGHETTSSLMDWVLFELGNRPEWRQKLRAEHQQVV

GNEPIAMSHLRQLPQMSNVLKEGERLYPPLFLISRMAIADIEYAGYLIPAGWYILIFPLL

THRLPEIYQDPDLFDPDRFAPPREEDKKQPYSLIGFGGGVHSCIGADFAQMEMKIILSTL

LQKYDWTVTPTTAEISPVRQPFMKQKKLTATFVSIEPIQS

>CYP110C30(BAZ43300.1)Chondrocystis sp. NIES-4102

MKSIPKLKQSSIQQRLQWVATPDRYMDMASLQAPDIFAADIASTGREYIFVNHPEAMRQI

VTSDRQKYFASSQDNQLLKPLVGENSLLLIEGDSHRQRRKLLLPPFHGERMEYYSQLICD

LTHNIIQQLQPNQPFIARRVSQTISLQVILEAVYGLQDSERSQFLKDRITKLANIFESTL

TSALLFFPLFQKDLGAWSPWGNFLRQRQAIDEAIYQELASRRIEGNNERQDILSLLMSAQ

DDTGEKLKDSQLRDELMTLMLAGHETTATAIAWSLYWVHRDPEIKAKLQEEIASLGENPH

PMELTKLPYLDAVCKETLRIYPVAMLTFPREVIESTELMGYKLEVGQVLMGCIYLLHQRQ

DVYPSPRQFKPERFLEREFTPYEFFPFGGGKRRCIGEALAMLEMKLVLATIISHYDLELM

SNRPELPARRGVTLAPKTGVRMIFKGKK

>CYP120A15(BAZ45792.1)Chondrocystis sp. NIES-4102

MNTTSNLNDLPQPPGNKGLPLIGETISFLNDPDFNQKRIAQYGKIYKTSVFGRPTVMMIG

SEANTFLFRNENRYVVATWPKSTRVLLGKASLAVNHGSLHTNRRKLLYEAFQPRALASYI

PTMAKITEEYLEKWAEMKTLTWYPQLRDYTFDIASNLFVSADGGANSSVGHYFETWCAGL

FTLPISLPWTKFGKAKAAREKLLTSLEEIILKRKQKSDPGEDALGLLIKAKDEDGNSLSL

EELKDQVLLLLFAGHETLTSAIASFCLLTAQHPDVLHKLQVEQAELNISGTPTLEDLKNM

TYLEQVLKEVMRIIPPVGGGFREVVETFEFNGYRIPKGWMVQYQIAQTHQQPELYPESDR

FLPDRFAPPVSVDKQASFGYIPFGGGLRECLGKEFARLEMRLFASKLLQNYRWELLPEQN

LELITVPTPHPRDGLKVNFYRLEH

>CYP110AM3(BAZ45271.1)Chondrocystis sp. NIES-4102

MTTVTSPANKNKLPPTVKTPGIITIIQALLDQFGTLEKYQQKYGELFYSPKSSLFPPNVI

FSNPKAIEQVFTADPNLFEVGTQSSAPVRVLLGDNSLVLLDGIKHKRHRKLLMPPFHGER

MKSYGETMVEITKEVISQWQVGQTICIRDYTQEISLQIILKTIFGLDQGKRCYRLKEILV

DWLEIFNSPLKSFFLFFPALQQDLGAITPWGRFLRLKRQIRDILQAECDRRRQDPDTMRE

DILSLLLEAKYEDGKGMSDEEIKDELMTMLFAGHETTASSLAWSFYWLHRRTEVGYRCQT

ELNSLRENIDFNEIIKLPYLDAVVSETLRLNPVVVFVNRQLKQPMELMGYHFEAGTSLLP

SIYLTHQREDLYPQPKEFKPERFLTRQFSPYEFLPFGGGNRRCLGYAFALYEMKLVLATI

LSHVELELLDNRPLPFGRRGFTFTPAGGVKMRVKAIK

>CYP120L1(NIES4102_42100) Chondrocystis sp. NIES-4102

MKIYGMLREKNNDNEDKVSFLPPGARKLETIKETIELIKDSQQFTELRSRKYGSVFKTCI

FGQPMIYVTGQAGCRFVLENEDIYFQNKMLPNMESLIGKFAVTTQIDRIHHNRRKILAKA

FTPKYLEEKIPIITQITAKYIERWDQHRYINWYDELQNYSLDIACKLFVGIDNGSQSELG

SLYKIWSEGLFSFALPLPWTKLGRALDSRDRILAIINNLIEDRKNKYWQNDLLGILLNAR

DEEGNCLTEHEVKDQILNMLSAGHGTLASALCSICLLLTQNPGVLERCRQEQKVFINCEP

MTINALEKMVYLEAVIKEVLRIFPPVGGGFREIIKDCEFENYYFPKGWRVIYNSFMSHKD

ATCFISPEKFNPDRFLELGENNYRGYFPFGGGKRRCLGENLARLEMKIFAAMIISRCEWQ

ILHGQDLTMQQLPFPHPKDNLKVSFSCQKY

>CYP110F4(AFY86012.1)Chroococcidiopsis thermalis

MKLTEVNKTPALLQTLQLIANPIEFLNTCANKYSDPFAVRVLGLNSPPVVFFSHPQAIKE

IFAIPSEYFDYKKATHVFQPLMGEQSLILQEGKSHQRQRQLMLPPFHGDRMKTYGQIICQ

ITQAVTQQWQVGKQISINHFLPDITLQIILQVVFGISPGERYEQLKVKLSSLLEDVTTPW

YSSLFFFPPLQKDLGAWSPWGHFVRRRQQIDKLIYAEISQRRRENNLTRTDILSMLMAAR

DENGQQMNDEELRDQLMSLLLLGYETTAAALAWAFYLLHSHPQVRDRLQQELNNADNTKP

DAIAQLPYLTAICQESLRVHPIALICTPRMVRDSVQIAGDKFNAGTIIVPCIYLTHRREE

IYPQPEKFQPERFLHQKFSPFEYLPFGGGSRGCIGAAFSLYEMKLVLATVLSQYELELAN

SRPVSPVRRGITIVPSENGMQMSVISG

>CYP110D22(AFY86608.1)Chroococcidiopsis thermalis

MRLPDGPSGPPWLRRLRFINWILRPFEVMEARAKKYGDIFAIAKNASPSMVYLSNPAAIE

QVLGANPEFFDTNSGNDVLLPLLGANSLILLGGMKHQRQRKLLMPPFHGDRLRTYGQTIW

DITTQVTSQWRSGQSITVRAATQEISMRVILSAVFGLDGGERYDRLRKLLTSLLETVSSP

VSSMVLFFPSLQKDWGKWSPWGLFLQMKQQIDDLLVAEIQQRRAEMLQRVETLHVTSPPR

DDILSLLLAARDEAGQPMTDEELRDELLTLLFAGHETTASALAWALYWLDRLPEVKEKLQ

AEIDSLGDNPDPSAIAKLPYLNAVCCETLRIYPIAISPFPRILKVPMEIGGYQLESGAIV

VISIYLTHQREDLYPQPKQFIPERFLERQFSPYEYLPFGGSNRRCIGAAFALFEMKLVLA

NMLSNYDLKLASNRPVKPTRRGLTVAPPANMKMVVQQKSKVESQNSKVLLTSDR

>CYP110B13(AFY86774.1)Chroococcidiopsis thermalis

MFLPQGPKTPPLVQLLQWIANPFALMESCAQRYGDWFTLKVGLNYRPLVYVSSPQLLQEI

LTNDHYKQFDAPGEINGIFAPLLGDFGVIMQSGDRHRRQRQLMVPPFHGDRMKAYGEIIT

EIAKQVTSQWNPNQPFSVRDSMQAISFNVILQAVFGLREGERYRQIEKLLYNMLELTNSP

LKASLLFFPFLQRDLGAWSPWGNFLRQKQQLDRLLHAEIEARRANPDPGRTDILSLLMAA

RDEAGEPMKDEELRDELMTLLVAGHETTATALTWALYWIHKLPEVRQKLLAELDILGDTP

DQNAIVRLPYLNAICSETLRIYPVGMLTFPRVVRSPVTLLGQQLEPGTVLVGSIYLAHQR

QDLYPEPKQFKPERFLERQFSPYEYLPFGGGVRRCIGAAFALFEMKLVLATILSNLELSL

ANNRPVHPVRRGLVSAPTKVEMVMTGRRIEGDKRDKGEGGDKGDKEAIPATSY

>CYP110A5(AFY87939.1)Chroococcidiopsis thermalis

MVGDRAIVSERFIRWRTIVLAQLPNPITSPAWWQLMNWIADPIGFQDKCSQKYGDIFTMH

LSGIGSYVVIGNPQAIGEIFNQDAKFDVGRGNAIAEPLVGRNSLMLVDGDRHRRERKLLM

PPFHGERLQTYAKQICLITKQIASSWQVGQSFVARTTMQKISLEVILQVVFGLSEGERYQ

QLKPLLTEWLDMTDSPLKSSMLFLQFLQKDWGAWSPWGQMKQRQHHVRALLQAEIEERRA

KDIKGRDVLSLMMAARDENGQAMTDEELKDELLTILFAGHETTATTLAWALYQIHQHPDV

RDRLLHELDSLGEFAPMKIAQLPYLSAVCQETLRMYPVIPVIFPRITKSPTKIAGYLFEA

QTALMPSIYLVHYREDLYPNAKQFQPERFLERQYSPSEYLPFGGGSRRCLGYALAGLEMK

LVLATILSKYQLALVEDKPVQIQRRGFTLAPQGGVSMVVSAKSI

>CYP110AJ1(AFY89291.1)Chroococcidiopsis thermalis

MSLPHGPRVIPFLHNLKWRGRFLEFLEYCVGRYGDIFTFRVIGSRPFVVVSHPQAIEEIF

TAPAGYFDSGKANQAFRALLGNNSLLLLDGIQHQQQRKLLLPPFHGEHLSKYGQIICGIT

QQEIQQWAVDRPFAIRPQVQAITLRIILQIIFGSQQESSVESLNQCLSELVDSFISPFWW

QRFSFPQKKQQLDRLVYAQIRQRRQETACSTPDILSLLLSARDEAGAALTDEEIRDELIT

LIMAGYETTTTAVLWALYWVAKLPQIREKLDKELCSLGSHPAPSEIARLSYLGAVCSETL

RIYSITGFTFDRVVKVPLSIMGYQFEPGTVLSPCTYLTHQREDLYPLPKQFQPERFLVRQ

FSPYEYYPFGGGNRRCIGMAFAQMEIELVLATILSHWHLSLVDDRPVRPVGRGVTLTPPL

SLEMVANRPN

>CYP1320C1(Chro_0853)Chroococcidiopsis thermalis

MALATTPPGPSSYIAFSKAYRDDPLRAFGQAWKTYGDSIRFKALPGVDVYFVVHPDAAAH

VLTSHGQAYRKAPSVHQPLSLLLGNGILISEGESWLRQRRLMNPAFHRQSIVKLASVMTR

FAQERVRRWEGYPTGSAIDVAEEMQQLTLEIVGEALFSTGLEAQLDSFSTAFRRAAEFIN

DRINAPFKMPMWVPTKPHRQFIENRDRLQQIAMHLIGLRRHQQNVPLDLLSMLMAAQDAD

TGAQMSDSELLDEVMTLLIAGHETVSVTLSWAFHLLGSHPEVLHQLQDELETVLKGNPPG

AEDYMHLPYTRMVIEETLRLYPPVWGLSRETIRADEIQGYSIPPKSFVIVGTYFTHRHPE

FWTAPEQFNPERFTEAEASKRHKFAYYPFGGGPRICIGNQFALMEATLILATLVQRFHLE

PASGQPVEIDPTFTLRPKNGLSMRLVRR

>CYP284A5(AFZ11054.1)Crinalium epipsammum

MLQKIAAQIAVASFPNLATVLGITSIVGILSFYWLKQKNTYKPLQSLPSPPKHWLLGNIP

QVLSAVKQKKFFQLVFEWSKELGSMYVYWADKPVVVLSKPKVIEETIINGMRDGSLVRPE

LANKAWNDLAGPILIGQNGSEWQWRRKAWNPEFSSSSLSKYIDIINQACEQIIEQIKETT

PSQAVKVDPLFVELTMRVISCLVLGIPVDSKIPSQEGPPLDVPKVYDAMSILGYRLIRVF

TGEKRWKKYLPTETSRSYWGSRRYIEELITPRVNLALQMREQNNIDLTQISLLFQESMLV

KIAAKEPKYNRETLIAEAMELLLAGTDTTAHTLSFAVGELILNQKVFQQAQAVVDRAWQG

TGSINAETLKELSYIRAIVKETLRLYSVASGSTFLEAQRDTIIDGQVIPRGTRVSWSMLA

AGRDPDIYHQPDEFLPERWLDKSKENSLLPMIDFGSGSHRCLGEHLSMLEATVMLALLLR

YFDWELVNGRSSLEQLQQNLLIYPSDGMPVRFRLRKAI

>CYP110C28(AFZ12224.1)Crinalium epipsammum

MKLPPSPKTHPFAQLLQWIATPLDYLEGTQKVYGDCFTARFGKLPPLVMVSNPQGIQQMM

TADSKQFDSGRGNQIFRPLVGDNSMLLLDGDRHQRDRQLLTPPFHGERMRTYGNLICDIS

DKVMSKRMIGEPFFVRAAMQEISLLAILNAVFGVNQGERFEKLKELLTSMLDSVSSPVSS

SVLFFNSLQQDFGAWSPWGRFLRQQKQIDQLIYAELEERRSHPDSSRSDILTLLMSARDE

QGQPMTDQELRDELLTLLIAGHETTASALTWALYWIHSSPEVHQKLLTEISSLSDPSDPT

VLAKLPYLNAVCQETLRIYPVAMLTLPRIVKSPINIMGYEFEPETTIMGCIYLTHQREDI

YPEAKLFKPERFLEKQFSPYQYLPFGGSNRRCIGMAFALFEMKLALATVVSRYQLSLTDK

HQPKPVRRGVTLSPSSSFKMVLNSQL

>CYP120B7(AFZ12802.1)Crinalium epipsammum

MAINPDAMSTTGCAYASKSLPPGSFGLPLIGETISFLRDPDFVTKRRKQYGSIFKTHIIG

RPTVIMSGAEANRFILSSDMHRFSWREGWPNTFKELLGGSLFVQEGAEHQRNRKLLMPAF

HGKALSNYVGTMERLTINYLEKWEQLGTFTWFSEFKQLTFEIASALLIGSEPGAQTAYLS

ELFHYLTNGLLAIPLRWRWTSYGKALQARDQILAHIEQAVRHRQQQPAQDALGLLVQSRD

EDGNSLSMEELKAQALLMLFAGHETTTSMLTSLCMALAQHPEVLAKARSEQQQLATNEEL

TLEQVRQMTYLEQVLKEVERMYPPVGGGFRGVVEEFEFQGYYVPKGWQVLYRITDTHLDS

GIYTKPDCFDPERFSPSRAEHKKQDFILVGFGGGSRNCLGVAFAQLEMKIVAAHLIRNYT

WELLPKQNLTLDAVPTLHPVDGLKVKFQRLSISN

>CYP110D21(AFZ14176.1)Crinalium epipsammum

MKLLHRHSTPRLIRRLQWFAYPLEYLEMYAKQYGDTFKVGGKNSPAAVYLSHPQAIQQVF

NAPPEIFDSGRGNGALNFLLGDNSLILLDGDRHQRQRKLLTPPFYSERMRAYSQLICDLT

QQVTNEWQIGKSFNVRTYMQEITLRVILQAVFGLHQGERYDQLQQMLTALLETIGSPLGS

AVLFFPVLQKDWGAWSPWGRFLRLRQQVDELIYTEIRERRQIDHPNNDILTLLISAQDQN

GQPMTDEELHDELMTLLVAGHETTASALTWALYWSHYHPEVKEKLHYELQSLGDNLDPNL

IAKFPYLTAVCQETLRIYPITATTFIRILKSPLEIMGEQLEAGTALIPCVYLAHHREELY

PESKHFRPERFLERQFSPYEYFPFGGGNRRCIGMGLAQLEMKLALATILSRFELALTSNH

PVKPVRRGLTMAPPGNLRMVVKNKLENVDKKLTKNISDSRKD

>CYP120B14(AFZ52279.1)Cyanobacterium aponinum

MTINREKKQEYPLPPGSFGLPLMGETLSFIKDPNFGNKKEAKYGSIFKTNIIGKPTVFMV

GAEANHFILQTHFDHFSWREGWPENFRTLLGESLFLQDGEIHQKNRRLLMPAFHGVALTK

YFNTMKEIIDRTLKKWAEMGKLTLFPEMKEMTFEIASVLLLGSEMDNREEIKLLSQKFGE

LTKGLFAFPFNLPFTNYGKALKARDFLLQHIEKEIEKRKNNLKEDTISLLLQSQDEEGNR

FSEAQIKVQALLMLFAGHETTTSMLTSFCMALAQNPEVREKAILEQKTLMDESDFTMEQI

KKMTYLDQVLKEVERLYPPVAGGFRGVVKPFVYNGYYVPKGWQVLYRIERTHKDPNIYTE

PKKFDPERFNSQRMEHKKTDFSLVGFGGGARFCLGYAFAQLEMKIFASLLLRNYHWQLEP

NQDLSLDRIPSLHPRSGLKVALYSGR

>CYP110C32(AFZ53578.1)Cyanobacterium aponinum

MVYVNQLSTPVWLQTVKWILNPPEYMSQAVKEYQDLFTAQITGFGNNILFVNHPDAIQQI

LTNDRQTFFADGELNTVLTPIVGYSSLLSLDGKRHKRERKLMMPPFHGDRMIYYGNSVQE

IVDNLFSKFKTNDIFITRETMQEVSLQVILKVVFGLTEGDRFLQMAQLIKDILDRFNQPI

NISFLFYEWLRKDFGKWSPWGSFLRIKKRLDDLIYREIEERRKEKNSERVDMLSLLLEAK

DENGEGLTNQELRDELMLILFAGHETTAIAMSWALYWIHYYPEVKERLLQELNTLPPDSD

GMTIYKQPYLTAVCNETLRIYPVAMLTFPRVATEDVQLLGQTVKKGTIVTGCIYLTHQRE

DLYPNPNQFQPERFLHRQYSPYEFLPFGGGVRRCLGEALATYEMRLVVAKILREYDLELV

TKKAVKPRRRGVVLSPKGGIPMKFLGHK

>CYP110C33(AFZ47748.1)Cyanobacterium stanieri

MKEIKTIFTGWGKKILWIVDPIEYMRQAVIECPDIFMGKPAGFGEPLVFINHPQGIQQLL

TNDRKTFFAGGDLNGLLVPIVGNSSLLSLDGSRHRRERKLMMPPFHGDRMVYYGDLVHDI

VDDLFKDFQVGDKFIAQNLMQNVSLQVILKVVFGLREGQRYDRIANLIKRILSLFNQPVN

VSFLFYKFLRQDLGKWSPWGNFLRVQKELDQLIYEEIKQRRIEKNPDRVDMLSLLLQATD

EQGEGLSDQELRDELMLILFAGHETTAIAMTWALYWSHYYPEVGAKIRAELEQLPHDADG

MTICKQPYLNAVCNETLRIHPVAVLTFPRLATEDIEMMGYHIPKNTVVAGCIYTVHHRED

LYPSHDEFIPERFLEKQYSPYEFLPFGGGVRRCLGEALASYEMRLVVAQVLSKYQLDLVE

KKPLKARRRGVVLRPEGGVPMIYRGKIDQ

>CYP120B11(AFZ48529.1)Cyanobacterium stanieri

MSSQNLDQNIPLPPGKFGLPLIGETINFLTDQDFALKRYQKYGSIFKTNILGKPTAVMIG

SEANKFILQSHFDHFSWAKGWPATFQELLGKSLFLQDGEEHKKNRKLLMPSFHGQALINY

FETMESIIKSYLNKWEKQQNFTWFSELKQMTFEIASVLLLGSERGDKIDYLSNLFTDLSN

GLFTIPLKIPGTKYSKAIRGRDLLLQHLEQEIIKRKENPSNDALSLLVQTQDEDGNSLSI

NEIKVQALLMLFAGHETTTSMLTSLCLVLAQHPDILDQAKKEQEKLQNTDIITLEQIKNM

TYLDQVLKEVERLYPPVAGGFRGVVKPFTFNGYYVPQGWQILYSIQLTHQNSEIFTNPKT

FDPQRFNKEREEHRKTEYSLVGFGGGSRFCLGYAFAQMEMKIFAALLIRNYHWNLEQGQD

LSLNRIPTLHPNSGLKVKEFQPLPK

>CYP213A14(AFY27388.1)Cyanobium gracile

MVSGVLETLAFFRDPDFARSRFERYGDVYETSLLGQRTVFIRGGQAIADLLAQGEAVEGW

WPDSVRQLLGPLSLANRNGADHKARRRVVGQLFAAAALRRYSPAIVALVEGLNQELLAAP

APVALVPRLRRFAFTVIATTVLGLDGADRDALFEDFEVWCRGLFSFPLALPGSPFARARQ

ARQRLLRRLGSVLQKAQAASASGAPLVAGGLDLLAGGLDEAGLPLADDDVAEQLLLLLFA

GYETTASALSCLLLTLLQHPAELAWLREELDGLSWPPAEADAVSAYDAVRAPRLDAVVKE

VMRLTPPVGGFFRRTREPIALAGVLVPAERVVQVSITASHRNGTDPEDLAAFRPQRHLGG

AEPVTLLPYGGGERVCLGKALAELDIRLLAVGLLKQVSLALEPDQDLTLLVIPSPSPKDG

LLVLPRRRTNAAGL

>CYP213A18(SBO42749.1)Cyanobium sp. NIES-981

MPDGLTPVAASAADGPRPLPRTGALSGVLEALAFFRDPGFAERRFDRLGNVFETVLLGQP

LVFIRGERALVDLFAQPDALEGWWPASVKQLLGSRSLANRNGADHRARRRVVGQLFSASA

LQRYTPAITALVEELRDELLVSGHPVPLAARLRRFAFAVIAGVVLGLEGEDREALFQDFE

IWTRGLFSFPVALPASPFARALAARERLLGRLTLVLRRARDPGRPASGGLDLLAGGVDEA

GVPLSDPDLVEQLLLLLFAGYETTASSLSCLMLALLQHPAQRAWLLEEIDTLPWPPEDGS

GSRAVGGPRLDAVVREVMRLMPPVGGFFRRARQPLVLDGVAVPAGRVIQVALASSNRYGA

CGQESAPEAFRPERHCDGSWTSTLWPFGGGERVCLGKALAELEIRLLTTGLLRGVEFDLL

PDQDLGLAVIPSPMPRDGLLVRARLRSAQGAPEHGGGPDQHQSGGP

>CYP284A9(SBO43261.1)Cyanobium sp. NIES-981

MLTQAPWLLPALPVAVAGLLAWGAWRRRRHFAPLRQLPSPPGVPLLGHIPAVLAAVRQKR

FFQLLHVWSETYGPAYVYWAGRPVLVLSRPALIETTIVQGMRSGCLIRSPGTRRAWNDMR

GPILIGEDGPAWQWRRRAWNPEFTAASIAAHLPLLQQASAQVLERLAQAPAAQAIPLDPL

FVEMAMRVIAALLLGIPLRPGAESPEGPPLDVARTYQAMGVLGYRFLRLATGESRWMKFV

PTRASRRYWDARRQLEDLLAPRVALALRLRDGNAGDDAGLTPGFRQCLLVRMAAKEPRYD

QDALTAEAIEFLIAGTDTTAHTLSFAAGCLALQPDVLARVRQEVDRAWERHGGLTPACIG

ALDLVRGVIKETLRLFSVASGSTSLQVVKAINLDGLGRVPVGTTLLWSMLGAGRDPQAYP

RPLEFLPERWQQGGADTPAPPMIDFGSGAHRCIGEHLAMLEATVMLAQLLRHYTWELVNG

AASLENQRQNLLIYPADGMPVRIRRRGAAEGSAAWS

>CYP110K4(ACB50046.1) Crocosphaera subtropica MTSTVTHTSTPNYSYPSGFKTYGIKNSLRLIFQPLQVLEENRNDYGDIYFSPEFGGFPPF

IIIGNSQGVEALFNINPDLLDTSSSNALLKPLLGDRSLIQLDGDQHQKRRKLLMPSFHGQ

RLQSYGNIITDITQNILNHWSKSQTFPMRKITQEISLKVILRAVFGLNLGERYQDLGRSF

TDFLDLFNSPLHTMTLFYPSLQKDWGKWTPWGQFQAYKRDVYHRLEQEIRQHQQGSDSED

ILSLLLSTVDEEGNHLSQEEIMDELVTLLFAGHETTASTLAWAFYWIHSQPEVYQNLMAE

LDSININSDPMMIAKLPYLSAVVSESLRIYPSVLFTFGRKLKTDLHFLDYYLPKETAIIP

CIYLLHHHLDIYPNSKQFQPERFLERQFSPYEFIPFGGSNRRCLGYALALYEMKLVLATV

LKQVKLALVNNKEILPIRRGFTMSPAGGVKMRVIQHY

>CYP1317A1(ACB50375.1) Crocosphaera subtropica

MIPTQQLERNKVDQSYQFNPFDAEFKANPYPYYDYLRTHDPIHWGMLGGWVITRYADAKA

ILKDPRVDEAPMPDSFRKKSQYLAKKERDLEALILASQHWLFFLNPPDHTRMRGLVAKAF

KGQSLQKIASEIQAIANQLLLPLKAERTLDIVTDFANFIPIKVMMKLMGLPKQDEHFVRG

WVRDIFSIFDPLNSLHKCEEMNQISLEFRDYLQHQINQKRKQPQPDLITALLEVEDGGEK

LTDAEIISSCMMLGAAGEGTTASIIGNSILALLNHRDQLELLKQQPHLINKAVEELLRYD

SPTQLVLRTAREPLEIAGKIINQGDFIILCLGSANRDPQQFVDPDQLDLLRSDNQHIAFA

TGIHFCLGAALARVEVPIAINTLVQQLPNLQLAINRVEYHDNVVTRFLKSLPVTFDVA

>CYP2287A1(ACB51997.1) Crocosphaera subtropica

MNSTNQSSVSLKPNLLDRILNRFQAWAIENPQNFVSLTLLPIIGFLEGFLANFLFSYPVY

LDMKKRKLGSTFCCAGQVVLADFKTIETNLTSPQARTWYLGISPLSARHCPQIDIGGRNV

FLISLSDTGTEGNDHDAFRHCVEDYFFTDEMKVRQHDPVGRELLETLGADYRELASENRL

EGFFIDENRGLMSFLIRYLHYVIFAIDPHDDSSIAALTNFHYTRRGTLHYFAASRILERF

NLLGWSEIPGMIEAVGTIYEKSPVLANFQENNPKYNNMTRRELAKLMVALMSIAALQGPL

HLGRTAMGYQSLPPYKGLKTSEIDVTAYWDRLDLDNPSSIKLFLLECGRLFTPVSASHRV

ATDSFTTTIAGKTRTFPPGTIILIPMILGMLTETVWGATPYEFQPQRENLCPYHMGFHSV

GDRHAGRICPGKDLALETLTDVIRIVGKVRRSQLGLTSH

>CYP110D9(ACB52046.1) Crocosphaera subtropica MTLPPSISTPRLLRLFKLIFYPLDYLEDYYKKYGDIFSFGQSESPFVYISNPQGIQEILT

KDKTDFRSAAGGSFLTTLLGENSLLFLQGKRHQRERKLLTPPFHGERLQSYTQLIYSITD

EVSENLQINQAFNVRKIMQEITLKVILKAVFGITEGDRYQQLKDLLKAWLSFFDSPANAT

IIFFPFLQKDWGNWSPWGRFLRIKGKIDDLIYTEIKERRQQENYTGKDILTLLMLTKDEA

GNPMSDQELHDELITLLIAGHETTASSLTWALYWIHYCPDVEEKLRLHFSILEKNTDLLN

IVKLPYLDSVCSETLRIYPVIVTTFLRILETPLELMGYQFKPGTVFAPAIYLVHHREDIY

PNSKEFRPERFLERQFSPYEYFPFGGGSRRCIGLELAKMEIKIVLFTLLSKYQLKLSSSR

PLKPVRRGLTIAPPNSFKMIITQKLTPN

>CYP120B13(ACB53076.1) Crocosphaera subtropica

MKAPSHPLPPGRFGLPIVGETLQFLLDPNFADKREKQYGSIYKTQILGRKTVFMSGSKAN

QFILSSHMDHFSWGKGWPNNFQELLGRSLFVQDGQEHQRNRKLLMPAFHGQALAKYLTTM

EQIISQYLSKWEKQKYLTWFPEMKKMTFEIASVLLLGTSPGENIEVLSQWFAELTNGLFA

IFPLRTGWSKYGKALAARDRLLEYIDQQIEERISNPGDDALGLMIQTRDENEGHLSKEEI

KVQALLLLFAGHETTTSMLVSFCMALAQHRDILEKAKAEQKALNIDRDLTPEKLKQMTYL

EQILKEVERLYPPVGGGFRGVVKPFVFNGYYVPEHWIVSYRINASHQDRNVFPNPEKFDP

DRFSRQREEHKKTEYSLVGFGGGPRFCLGYGFAQMEMKIFASLLLRHYFWEILPDQNLTL

DAIPTLHPRSGLKVKLVSSLN

>CYP110C8(ACB53173.1) Crocosphaera subtropica

MIVEINYHKEIRYLTTIIISRMKTIPTAKSPKFIQQLQWILNPTGYLQTNHRRYPDIFKA

KVIGLGDNVIFTSDPEIMQYVLTHDRKQFTSPSSLNALLRPLIGDYSVIMLDSDRHRQRR

QLVMPSFHGERLKVYGDLTCRITKEVMEKVPQNQPFLAREIMQDISLKVIMEAVFGVTQG

ERYEELQDRLKKMLDLFNSPVTSTFLFFPFLQKDWGNWSPWGRFLRQRKAIDDLIYAEIS

DRRANPDSDRTDILSLLMFAKDEQGQGMRDQELRDELMTLLTAGHETTASAMAWALYWLH

DTPEVKDKLIEELDTLSPNAEGMDIFRLPYLTAVCNETLRLSPSAMLTFTRVVQEKVEVA

GYTFESGDMIQGCMYLTHRREDLYSNPEQFNPQRFIDRQYTPYEFIPFGGGSRRCVGEAL

AQFEMKLVIATIMSQYCLKLADTQPEKQQRRGLTLSPARGVKMILEGKRQPQPVRELELS

RR

>CYP110K2(ACB53761.1) Crocosphaera subtropica

MRKTRMTATIKAKKSNDLAFPQGIKRYGLKGILNLIFRPLPTLESNRNRYGDIYYSPAFS

SFPPFIVVGNPEGIEALFTADPDLFNSGTSNAAFQPLLGSQSILQLDGEPHKKRRKLLMP

SFHGQRLQTYGEIITNITQAILNGWQKKDIFSMREVTQEITLSVILQAVFGITQGDNYQS

LRQQLSCYLDLFNSPLYTSTLFLPILQKNWGTWTPWGNFIDQRDKVYELLSQEIDKRATT

KDGEDILSLLLSVTDEQGEHLTKGEVMSELMTLLFAGHETTASALAWAFYWIHFHPEIYH

NLQDELDTINVDTDPMEIAKLPYLNAVVSETLRIYPIALFAFSRTLKTSWEFMGYSLETG

MSLAPCIYLVHHHPDIYPNSKQFQPERFLDSQFSPYEFIPFGGSNRRCLGYALALYEMKL

VLATVLKQVNLKLVDSQPILPVRRGFTMSPQGGVKMQVI

>CYP110A9(ACK69982.1) Gloeothece citriformis

MAKLVPIPVNRPSWWQMVQWISDPLSFQQKWAKKCGDIFLINLWRYNDNLVVVGSPQLIQ

EIFNKDATQFDIGRGNQIVAPLVGQNSLFLMDGTRHKRERKLLMPSFHGERLQTYAQQIC

EITKEVASQWQKDKPFIARTVMQDITLEVIIQVVFGLREGERYQKIKPLLAAMLNMIDSP

LPSSFLFLRFLQKDLGAWSPWGKLKHRQLQIRKLLEAEIEDRRTHNFIGTDILSLMMAVR

DENGEPMSDLELKDELMTLLFAGHETTATILAWAFYKIHQLPEVRDKIVQELDSLGEKPN

PMEIAKLPYLNAVCQETLRMYPVIPVLLPRITKTPMDIGDYHFEAGMALMPSIYLVHYRE

DLYPQPQEFKPERFLDRQYSPSEFIPFGGGSRRCLGYALALLEMKLVLGTILSSYQLALV

DNKPVKIQRRGLTLAPVGGVPLVMKSKRQPKSPSSQKTASLIP

>CYP110C24(ACK69983.1) Gloeothece citriformis

MTSMTKTVSGSKTPGFLQQINWAFDPVSYLETNHQKYPDLFFTQAASGNYPIVYISHPQG

IQQILTNDKKLFLASSETNDIFRPLLGDYSLILLEKEPHQQRRQLLAPPFHGERLYSYGQ

SICRITQEVMNRLGVGNQFTARKITQSITMQVILEVVFGIYEGERYQQLKTLLGKMLDIF

NSPVSSAFLLFSALQINLGSWSPWGQFLRQRAKIDELIYQEIAERRANFDPNATDILSLL

MSVRYEDGQPMTDKELRDELMTLLVAGHDTTATAMAWGLYWIHHLPEVKEKLLQELATLG

NNPDPMEMVRLPYLSAVCNETLRITPVAMLTFPRIAQEPVEVMGYTIEPGTAVLGCMFLT

HQREDLYPNPKQFKPERFLENKYSPYEFIPFGGGARRCVGDALAPFELKLVLGTIMSNYE

LSLADNRPEKLQRRGLTLGPARGVKMIFNGKRQQKSVMSQESLVSSH

>CYP120A23(ACK71092.1) Gloeothece citriformis

MKEKSLPPGNLGLPIIGETISFLNDSDFASKRHQKYGSVFKTRIFGRPTIFVSGADAVRF

VLTHENKYFASTWPKSTRTLLGPASLSVNTGEFHTSRRKIMFQAFQPRALASYIPTIERI

TDDYLAKWETMETFQWYPELRNYTFDIASSLFVGIENGSQTRLGELFEDWCAGLFSLPLP

FPWTAFGKALRCRDGLLEEIETIIKQRQQQDNFGNDALGILLTATDEKGNKLSLEELKDQ

ILLLLFAGHETLTSALASFCLFTVQYPQVSTEIKKEIERLQIGTSVTLEELKQMEYLEQV

LKEVLRFVPPVGGGFREAVVDCELEGYLIPKGWIVQYQIRRTHRDTTVYNEPQQFDPSRF

NNNRAEDKQKTFAYVPFGGGLRECLGKEFARLEMRIFASRLVKDYQWNLIPNQDLSMTAT

PTPHPRDGLKVNFHRLKSEVKVN

>CYP110D17(ACK73512.1) Gloeothece citriformis

MTFPPRPTEPQLLRTLKLLFRPTDYLDDYGKRFGDCFAIGSSERGFVYINHPTAIQKIFT

APAEQFESGRGNGVLRFLLGDNSLVLLDGESHQRQRKLLMPPFHGERLRTYQHLICEITQ

QVTEQLTVGKTFRVRPIMQEITLRVILQAVFGLTEGDRYAKLRGLLTTVLDSVSSPISSS

LLFFRFLQKDWGPLSPWGRFLRLKAEVDQLLCDEIQERQKQEKLDGDDILTLLLSARDEN

GEPMTLLELRDELMTLLVAGHETTASALTWAFYWVHSLPEVQDKLRFELSNLGDNPDLSE

ISRLPYLNAVCLETLRIYPVAVMAFARILKSEMELLGYKFRANTALGPCIYLLHRREDLY

PEADKFKPERFLERQYSPYEFIPFGGGNRRCIGMALAMLEMKLVLASILSNFQLELINSR

PLKPVRRGLTLAAPNSFKMRVSSRH

>CYP110K6(PCC7424_0827) Gloeothece citriformis

MTTVISSETKSHQPLPKITAPVWLQTVEALFQPLEYLEKQRSRMGDIYWGESFAFPHLVI

IGDPKGIEQVFTADPNCFEIGNSNKIVHPIVGGYSLILLDGQSHQQQRKLLMPPFHGERM

RNYGQTIYNVTQQVMEKWPKNQPFLMRQSTQEITLRVIIETVFGVHQGEKLERLQHLLNE

FLEIFNKVWGAMFLFYPILQKDLGAWSPWGKYLRLRQEIDEILFTEIKERRHQLPTEDIL

SLMISAKDENGQSMSDQELRDELMTLLFAGHETTASAIAWAFYWIHRQPEIKQKLLEELK

EVDVENEGITVAKLPYLSAVCAETLRIYPVAIFAFSRKTKVPMEIMGYQIEPGMTLLPSI

YSVHHREDIYPNAKQFRPERFLERQFSPYEYLPFGGGNRRCLGYAFALFEMKLVLATILK

QTQLTLLEPKEVKPMRRSVTFTPSTGIKMRVNQ

>CYP110A9(PCC7424_1542) Gloeothece citriformis

MAKLVPIPVNRPSWWQMVQWISDPLSFQQKWAKKCGDIFLINLWRYNDNLVVVGSPQLIQ

EIFNKDATQFDIGRGNQIVAPLVGQNSLFLMDGTRHKRERKLLMPSFHGERLQTYAQQIC

EITKEVASQWQKDKPFIARTVMQDITLEVIIQVVFGLREGERYQKIKPLLAAMLNMIDSP

LPSSFLFLRFLQKDLGAWSPWGKLKHRQLQIRKLLEAEIEDRRTHNFIGTDILSLMMAVR

DENGEPMSDLELKDELMTLLFAGHETTATILAWAFYKIHQLPEVRDKIVQELDSLGEKPN

PMEIAKLPYLNAVCQETLRMYPVIPVLLPRITKTPMDIGDYHFEAGMALMPSIYLVHYRE

DLYPQPQEFKPERFLDRQYSPSEFIPFGGGSRRCLGYALALLEMKLVLGTILSSYQLALV

DNKPVKIQRRGLTLAPVGGVPLVMKSKRQPKSPSSQKTASLIP

>CYP120A17(ACL44781.1)Cyanothece sp. PCC 7425

MAKLKLILLQVTVRHPPLVNFNYRKLPVHPIMTTAPAAHSLPLPPGRSGLPFIGETISFL

TDPDFADKRHKQYGELFRTHLFGRPTIYLAGAEAVRFLLLHENQYFRTSWPASTQALLGP

ASLSVQQGSTHQQRRKLLAQAFQPRTLASYITTMMDITRHYLDRWQQQGTLTWYPELRNY

TLDIACKLIVGISSGSQTHFGEWFEIWLQGLFSIPLKLPGTRFSRALRSRELLLAEIERI

VLERQQQEDAGQDSLGLLIQARDDEGNGLSVAELKDQVLNLLFAGHETLTSALTAFCLLM

AQHPTVLERIRAEQETFKQRQSLTLEDLKQMEYLEQVLKEVLRVMPPVGGGFREVIQTCE

IDGYKIPQGYSVLYQIGRTHQDSTIYPEPKQFDPDRFDGNRTDKTIPFSYVPFGGGVREC

LGKEFARLEMKIFAALLVRDYEWELLPDQNLDFEMIPTPRPKDGLKVKFR

>CYP110E21(ACL46340.1)Cyanothece sp. PCC 7425

MTLPAGPPTLPLVQLLQWIFTPFAYLEDNQRRYGDIFTARFAGFPPFVLVSDPQAIDAIL

TAPVQQYDSGRANVILRPTLGDHSLLLLDGELHQRQRQLLMPPLHGERMRAYGQLICQIT

EAVMDQWQVGQNLTLRPWMQEISLQVILKAVFGLADHRRYQELKQLLSSLLELLTSPVGN

VLAFFVNLQMDLGPWSPTGKFLHLKAQIDQLIYAEIAERRQEGSDRSDILSLLLAARDEV

GQPMSDQELRDELMTLLIAGHETTATALTWALYWIHRSPEVKARLLVELDSVGEDADPGI

LAKLPYLNAVCAETLRIYPIALITLLRIARQPVDLQGVHFAPDTYLAPCIYLVHQRPDLY

PQPREFRPERFLERQFSAYEYLPFGGGHRRCIGAAFALYEMKLVLATLLRHWQFRLTDSR

PVRPQRRGLTMAPAGGVKITAVATTARSPQKRPQREVPPDSARTPIAP

>CYP110D18(ADN12630.1)Cyanothece sp. PCC 7425

MTLPPRPTQPQMLRMLKLVFRPTDYMDENGKRFGDCFAVGRSETPFVYISNPKAIQQIMT

APAEQFDSGRGNGILKFFVGENSLILLDGVAHQRQRKLLTPPFHGERLQTYQQLICDIAA

QVTEPLQVGKPFRVRTIMQEITLRVILKAVFGLQEGERYHKLRQLLTTVLDSISSPLSSS

ILFFRQLRKDWGPLSPWGRFLRLKAQVDQLICEEIRERQQQEKLDGEDILTLLLSARDEN

GEPMTLLELRDELMTMLIAGHETTASILTWALYWIHYLPEVGDKLRFELSSVEDKSNLAH

ICKLPYLNAVCSETLRIYPVAPLTFPRILKSDMELLGYKFAANTVLAPCIYLLHHREDLY

PQPDQFKPERFLERQYSLYEYIPFGGGNRRCIGMALALLEMKLVLATLLQRFQLELLNSR

PLKPVRRGLTIAPPNNFKMVLKSVNYH

>CYP1136A1(ADN13000.1)Cyanothece sp. PCC 7425

MTNQLLNNFLLLEEGQTPPSETTLPPRQPQWFDTFSYIADPDQFCRQNLAKYGPIFKTGV

FGETTVFVGSAKVNQMAFNGDQHYTEIALPPTTMDMFGQYSLFQRTDLHRSRKSALSPAF

TGKMLEGYLPLINQVVLEGIQSWKTTDLLSVFPAVEKICFDVLVPLLLGVDLNQKDSLKG

LPISSKTELKALYKTFFDGFYGLVKWKSPLTVYGRGYQAREKLLDFMGSVIQQRRAQGEV

INSQADFLSMMLAGQEENPTGVFQDIFIKNQCLLQLWASHYEICGLVSSLIYQIGRHPEV

KKRLVQEQIEVMGEQTSDKMITSQQLKAMVFLEATIKETLRTLSPSSTVNRRLTKSVVLD

GVLYQKGWVLIAEQRIAHILPEHFKQPDVFDPERFLSPRNEGKMYEFIAFGGGVHACLGA

QLAMLITKVFACYLLQLLNWEVTQAASFVQFPLKRLKSNYQIPIHSRATA

>CYP120B15(ADN15271.1)Cyanothece sp. PCC 7425

MTQRTLDGQSYPLPPGNLGLPILGETLSFLQDRNFANKRHKKYGSVFKTHLFGRPTVILM

GPEANRFILSTHFDHFSWREGWPKMFRELLGRSLFLQDGEEHRRNRKLLMPAFHGPALNQ

YITTMEEIIDRYLNNWEKQGSIAWFFELKKMTFEIASILLIGSEPGELTDMLSQWFTELT

SGLFTLPIALPGTTYSKALKARDRLLNHIEKVVQERQKHPTSDALGLLVQTRDEEGNSLS

LEELKVQALLMLFAGHETTTSMLASFNMVLAQNRQIRARLNTEIENISPKGSITLEQLRQ

MTYLDQVLKEVERFYPPVAGGFRGVVKPCVFGGYYIPEGWQLLYRIDATHLDQRVYTNPE

QFDPDRFSPERAENKKMEYSLVGFGGGSRICLGYTFAQMEMKIFAVHLLRHYDWELLPDQ

DLSFHPISTINSGSGLLVKFYRYN

>CYP110AM2(ADN15584.1)Cyanothece sp. PCC 7425

MTSVISTQAAAKLPDGPKIPAIIQTILALMDQFGSLERNYQKYGDIFYTPKSSLFPAFVA

LSDPKAVEKVLTANPRLFEVGKQSSLAVRVLLGDNSLVLLDGIEHQKRRKLLMPPFHGER

MKSYGQTIVDVTKEVMAQWQEGKSFSIRDYTQQISLRVILRTVFGLDEGERYARLEKILT

AWLNIFNSPFNAFFLFFPVLQKDFGAWTPWGQFVEQKRLIHEILQSEIERRRHNPDSLGE

DILSLLLSVEDEEGQPMSDTEIKDELMTMLFAGHETTANTLAWAFYWIHYRPEIYQKLLA

ELNSLDENADFNAINKLPYLNAVVSETLRLYPVVPFLSRQLKEPFEIMGYQFEAGTALLP

CIYLIHQREDIYPQPKQFKPERFLEKQFSPYEYLPFGGGHRRCLGYAFALFEMKLVLATV

LSQVHLELPFQRPPKAIRRGITFAPSGGLKMRLKKQV

>CYP110C26(ADN16249.1)Cyanothece sp. PCC 7425

MTTVLPGSKTPGLLQQLHWAFDPVGYLESNQQKYPDLFLANVAGWKQPILFVSHPQALQY

ILTNDRKQLSAPKKTNEIVRPLVGDYSILLLEGQRHQQRRQLLAPPFHGERMYSYGESIC

QITHKVLSELPLNKPFSARQVTQSITMQVILEVVFGLYEGERYQQLKKLLSKMLDIFNSP

IASALLLFPALQLDLGTWSPWNKFLRQREQIDHLLYQEIAERRANPNPEATDILSLLMSV

RYEDGQPLSDQELRDELMTLLMAGHDTTATAMAWGLYWIHHLPHVKEKLLQELATLKEKP

DPMEIVRLPYLSAVCNETLRITPVAMLALPRVVQEPLEILGYALEPGTAIFGCMFLTHRR

SDLYPDPKQFKPERFLERKYSPYEFIPFGGGTRRCVGDALAPFELKLVLASIVSGYDLSL

ADTRPEKLQRRGLTLGPARGVLMILKGQRTLEKPSENLVTV

>CYP110C13(ACK65183.1) Rippkaea orientalis PCC 8801

MKSIPQSEKPLLLQRLQWIFDPVTHLENTHAECPDIFYSQVMGVEGSVIVSHPQAIQQIL

TNDRKQFSSPSQYNQLLQPLVGDNSTIMIDGDRHRKRRQLVMPSFHGERLKTYGELTVRI

TKEVLNQLPTGQPFLGRPTMQSISLKVIMEAVFGITQGERYEKLQRLLGQLTDLFESPVT

SALLFFPSLQKDWGSWSPWGKFLRQRQQIDELIYAEISDRRSHQDPNRTDILSLLIEARD

EAGEPLSDQELRDELMTLLIAGHETTATAMTWGLYWLHRTPEVKEKLYQELTSLGESPDA

MEIFRLPYLTAVCNETLRISPVTMLTFPRVAEEPIELLGYKIEPGTLIMGCMYLTMQRED

LYPNPREFRPERFLERQYSPYEFLPFGGGVRRCLGEALAQFEMKLVLATIIANYQLKLAE

KQPEKLQRRGFTLAASRGVKLIKLGLLSNNKVVHNDEQQPIRDRLQSVDAIAN

>CYP110K3(ACK65418.1) Rippkaea orientalis PCC 8801

MTTTTQPLAKQTNSFPNGPESYGFRETLKLIFNPLPALEESRNRYGDIYSFPGISNLPPS

IILGNPQSIEELFTANTDLFDSGASNAALKPLLGDMSILQLDGNPHQQRRKLLMPSFHGQ

RLQSYGNIIIEITEKVIKQWKINQPFLMRNVTQEITLKVILKAVFGVNEGEYYDQLRQKF

SHYLDLFNSPIYTTTLFLNYLQKDLGSWSPWGHFQRQKQAIYELLSQEIETHRQQDGGED

ILSLLLSTTDEDGNPLSKAEIMDELVTLLFAGHDTTASALAWAFYWIHSQPDVYHNLMNE

LGTIDAETDPTNIAKLPYLNAVVSETLRIYPIALFAFGRMLKSPLDLMGYSFDKGTIFSP

CIYLVHHHPEVYPNSKTFKPERFLERQFSPYEFIPFGGSNRRCLGYALALYEMKLVLATV

LQKTALKLASSRPVVPVRRGFIMTASGGVKMIATKIN

>CYP110AK1(ACK65747.1) Rippkaea orientalis PCC 8801 MITIPGDRRNPSWLHKIKFVLDPIGYMESNYKQFGEIFHAPISGNYDPYIWVSHPQAFQK

LFSTNPSYFDTVGSVYIKNFIGKDSVVAAEGKRHQRKRKLLLPPFHGEYLDNYGKSIILI

TNNAVKNLINNQLFLAHHLMQNITLEVILKVIFGVPEKPRIKQLKKAIIAWLEALNSPLI

SAAILVPSLQINLGSWSPWGKYSQSKQKIAEIIHEEIAERRQDNNDSYSDILSLLMNAKD

EQREAMSDEELHDEILGLLFAGHETSAAGLTWAIYQLYKCSNVRKKLLQEIDTLGNNFEV

KMIVNLPYLSAVCNETLRMYPPLPKTITRIANQSVNLMGYNLPMGTPVNGSIYLCHHRED

LYPNPQEFRPERFLERKFSPYEFIPFGGGTRSCIGQALAILEMKLVLATILARYQLKLLE

NKTVKPKMRGTTLVPTGGVKMSFQGLR

>CYP1007A2(ACK65934.1) Rippkaea orientalis PCC 8801

MIALTQKPEWTIPGPSTLPLIGRSLNVIRFGKDCIGLSNELFNTYGKVVSLAASGGTNLY

SADNNCPGTILAYGPEIVRQVTTQHNIYHKRPLSGTLYRHKDDSPRTEPLKNYGVGLFGV

NGEEHLQQRKLMMPAFHKTQVESYRDEMVAMTQLEIDQLAINQPCEISQLMQRLTLRIAT

KTLFGEDINSVDSTAGELLQQVLNCQRSSSIMLFPFDIPGLIFHRYLNLLAQYEAKIKKI

IDDKRAKGANDNDVLSMLIQAREEESGHPLSEAELIAHTGVIFLAGHETTANALTWTMFL

LSQHPQILGDLVNELESVLQGEPPTLEQLPQLPLLDRVIKESMRILTSVPWNGRVTSETT

ELDGYVLPKGTEVLVSIYHTHHMSEIYPDPEAFKPERWETITPSIYEYNPFSAGPRLCIG

ATFAMMELKIVLGMLLQRFRWQYIGGQQIDRAGVISLKPKYGLSMRVCPQDHEFNQGVGE

VQGNIRETVKLS

>CYP110D24(ACK66175.1) Rippkaea orientalis PCC 8801

MKPLSTIPATSGWRRTIRLLNSILRPLAFMEERTQKYGDFYQVTFKNAPPTVMTSNPRAI

EEILTASEDTFEVGRGNQGLKFLVGDHSLLLLDGKTHQNRRRLLMPSFHGEALQKCSSQI

VETTQEITANWRINQPFKVRRVMQEITLRVILKAIFGQDSGERYTRLRELLTNLLEIFNI

PLTAIFIFFPSLQKDLGRLSPWGRFLAWKKEIKTLIYEEIQERRERLSSGQEQATDILSL

LLLAKDEDGLPLTDEELHDELITLLFAGHETTASALSWLFYWVHSLPEVQDKLRFELNSI

GDLSDYKTINKLPYLDAVISETLRIYPIAATTFARILTKPKRIMGYDFAPKTWFMMSVYS

LHHREDLYPNPKQFQPERFLQKTYSLYEYLPFGGGNRRCLGSALALLEMKLVTATILQQF

QLELTSKKPMFPVRRGLTIAPPAYFSIKVKGLNN

>CYP1159A1(ACK66447.1) Rippkaea orientalis PCC 8801

MFKGLKIALQADTGRWFTRCNNCQQTIGNNPDTVTVHVEGSVSEHPYAQFEVVDVGNGKI

ALKADTGKYVGRCNGCIVGGAYPDFLTIHVDDPSMPWAQFTPERLANGKYAFKADTGKYF

GRCNGCSPTSAYPDTVAVHVDNPHNSPWAQWTVSYVPFSYLERYDAIPADNVAEKAKLVG

RAMATDSRNFFKELRANRPIFITPKFVLVTLFPDVQEVFSRPEVFSVRLYAPKMDPNHGP

AMLSRDNTVYNWREKSIMKTMLDWEDLPRIKQAAGEVAKAALDKFAPTKKIETVNELAKW

VLVRMSGDYYGFPGPDRETMYRWSSATQSGMLRNLANDPQIHEASVQAGKEMRDYLTQLL

QQKKANNTPSTAPKDIFTRLVQANLTSDIPFDESRILTNMALLLISTLDTTAQAIVQSLE

QLLRRPDILPKAVAAAKANDDVTFAKYVWEALRFNPVSPALPRFCESDYTVAAGTSRATR

IPANSLVLVSLGSAMMDGAIVQNPEQFSIERPKHNYMHYGYGDHTCLGEHIGNVVVSEVI

KQVLLRPGVRLIPGDEGKLQAQPNAILKSFVIAYDG

>CYP1146A1(ACK67088.1) Rippkaea orientalis PCC 8801

MSNDLNRIPGPSPQFLLGNALDFANTPSHIRLYEYSLAYGDIMLFWLFTQPSILINDPAL

IEQVLVIDRDRYYKNAPRKAAEPVMGDSLFLSNGQDWEFKRRNHPFSAPQIDTYFEQILP

TIQTTTNRYLQALQPSSTPKSVELFGELVKLSFEIFCLTVLGAEMDSSYFDAFSLLMEEM

NNRGGQAFLPYPLSLNPQFWVARQRWNSFIEEQIKERQQRSPQQGIDLLSFVIQDTKLST

KQLREELSTAYTAGTRNVAEAVAAVLFLCAKNPQVMQTLLSEITLFLRESGSDFSLAQIN

QFNYLDLVVKEALRLYPAVPFFIREVLPKQSPMLGGYTLPEKTQIFVSSWSFHRNPKYWQ

NPHEFMPERFINPPPAFHYFPFGAGPRQCIGMAFTLTCTKVMVVNILSQYSVELEPNTTF

ETQYFSGTIMPRDGLKIKLSRQLL

>CYP110C13(ACV00069.1) Rippkaea orientalis PCC 8802

MKSIPQSEKPLLLQRLQWIFDPVTHLENTHAECPDIFYSQVMGVEGSVIVSHPQAIQQIL

TNDRKQFSSPSQYNQLLQPLVGDNSTIMIDGDRHRKRRQLVMPSFHGERLKTYGELTVRI

TKEVLNQLPTGQPFLGRPTMQSISLKVIMEAVFGITQGERYEKLQRLLGQLTDLFESPVT

SALLFFPSLQKDWGSWSPWGKFLRQRQQIDELIYAEISDRRSHQDPNRTDILSLLIEARD

EAGEPLSDQELRDELMTLLIAGHETTATAMTWGLYWLHRTPEVKEKLYQELTSLGESPDA

MEIFRLPYLTAVCNETLRISPVTMLTFPRVAEEPIELLGYKIEPGTLIMGCMYLTMQRED

LYPNPREFRPERFLERQYSPYEFLPFGGGVRRCLGEALAQFEMKLVLATIIANYQLKLAE

KQPEKLQRRGFTLAASRGVKLIKLGLLSNNKVVHNDEQQPIRDRLQSVDAIAN

>CYP110K3(ACV00304.1) Rippkaea orientalis PCC 8802

MTTTTQPLAKQTNSFPNGPKSYGFRETLKLIFNPLPALEESRNRYGDIYSFPGISNLPPS

IILGNPQSIEELFTANTDLFDSGASNAALKPLLGDMSILQLDGNPHQQRRKLLMPSFHGQ

RLQSYGKIIIEITDKVIKQWKINQPFLMRNVTQEITLKVILKAVFGVNEGEYYDQLRQKF

SHYLDLFNSPIYTTTLFLNYLQKDLGSWSPWGHFQRQKQAIYELLSQEIETHRQQDGGED

ILSLLLSTTDEDGNPLSKAEIMDELVTLLFAGHDTTASALAWAFYWIHSQPDVYHNLMNE

LGTIDAETDPTNIAKLPYLNAVVSETLRIYPIALFAFGRMLKSPLDFMGYSFDKGTIFSP

CIYLVHHHPEVYPNSKTFKPERFLERQFSPYEFIPFGGSNRRCLGYALALYEMKLVLATV

LQKTTLKLASSRPVVPVRRGFIMTASGGVRIIATKIN

>CYP110AK1(ACV00623.1) Rippkaea orientalis PCC 8802

MITIPGDRRNPSWLHKIKFVLDPIGYMESNYKQFGEIFHAPISGNYDPYIWVSHPQAFQK

LFSTNPSYFDTVGSVYIKNFIGKDSVVAAEGKRHQRKRKLLLPPFHGEYLDNYGKSIILI

TDNAVKNLINNQFFLAHHLMQNITLEVILKVIFGVPEKPRIKKLKQAIIAWLEALNSTLI

SAAILVPSLQVNLGSWSPWGKYSQSKQKIAEIIHEEIAERRQDNHDFYSDILSLLMTAKD

EQREAMSDEELHDEILGLLFAGHETSAAGLTWAIYQLYKCSNVRKKLLQEIDTLGNNFEV

KTIVNLPYLSAVCNETLRMYPPLPKTITRIANQSVNLMGYNLPMGTPVNGSIYLCHHRED

LYPNPQEFRPERFLERKFSPYEFIPFGGGTRSCIGQALAILEMKLVLATILARYQLKLLE

NKTVKPKMRGTTLVPTGGVKMSFQGLR

>CYP1007A2(ACV00811.1) Rippkaea orientalis PCC 8802 MIALTQKPEWTIPGPSTLPLIGRSLNVIRFGKDCIGLSNELFNTYGKVVSLAASGGTNLY

SADNNCPGTILAYGPEIVRQVTTQHNIYHKRPLSGTLYRHKDDSPRTEPLKNYGVGLFGV

NGEEHLQQRKLMMPAFHKTQVESYRDEMVAMTQLEIDQLAINQPCEISQLMQRLTLRIAT

KTLFGEDINSVDSTAGELLQQVLNCQRSSSIMLFPFDIPGLIFHRYLNLLAQYEAKIKKI

IDDKRAKGANDNDVLSMLIQAREEESGHPLSEAELIAHTGVIFLAGHETTANALTWTMFL

LSQHPQVLGDLVNELESVLQGEPPTLEQLPQLPLLDRVIKESMRILTSVPWNGRVTSETT

ELGGYVLPKGTEVLVSIYHTHHMSEIYPDPEAFKPERWETITPSIYEYNPFSAGPRLCIG

ATFAMMELKIVLGMLLQRFRWQYIGGQQIDRAGVISLKPKYGLSMMVCPQDHEFNQGVGE

VQGNIRETVKLS

>CYP110D24(ACV01076.1) Rippkaea orientalis PCC 8802

MKPLSTIPATSGWRRTIRLLNSILRPLAFMEERTQKYGDFYQVTFKNAPPTVMTSNPRAI

EEILTASEDTFEVGRGNQGLKFLVGDHSLLLLDGKTHQNRRRLLMPSFHGEALQKCSSQI

VETTQEITANWRINQPFKVRRVMQEITLRVILKAIFGQDSGERYTRLRELLTNLLEIFNI

PLTAIFIFFPSLQKDLGRLSPWGRFLAWKKEIKTLIYEEIQERRERLSSGQEQATDILSL

LLLAKDEDGLPLTDEELHDELITLLFAGHETTASALSWLFYWVHSLPEVQDKLRFELNSI

GDLSDYKTINKLPYLDAVISETLRIYPIAATTFARILTKPKRIMGYDFDPKTWFMMSVYS

LHHREDLYPNPQQFQPERFLQKTYSLYEYLPFGGGNRRCLGSALALLEMKLVTATILQQF

QLELTSKKPMFPVRRGLTIAPPAYFSIKVKGLNN

>CYP1146A1(ACV01859.1) Rippkaea orientalis PCC 8802

MSNDLNRIPGPSPQFLLGNALDFANTPSHIRLYEYSLAYGDIMLFWLFTQPSILINDPAL

IEQVLVIDRDRYYKNAPRKAAEPVMGDSLFLSNGQDWEFKRRNHPFSAPQIDTYFEQILP

TIQTTTNRYLQALQPSSTPKSVELFGELVKLSFEIFCLTVLGAEMDSSYFDAFSLLMEEM

NNRGGQAFLPYPLSLNPQFWVARQRWNSFIEEQIKERQQRSPQQGIDLLSFVIQDTKLST

KQLREELSTAYTAGTRNVAEAVAAVLFLCAKNPQVMQTLLSEITLFLRESGSDFSLAQIN

QFNYLDLVVKEALRLYPAVPFFIREVLPKQSPMLGGYTLPEKTQIFVSSWSFHRNPKYWQ

NPHEFMPERFINPPPAFHYFPFGAGPRQCIGMAFTLTCTKVMVVNVLSQYSVELEPNTTF

ETQYFSGTIMPRDGLKVKLSHK

>CYP1159A1(ACV02486.1) Rippkaea orientalis PCC 8802 MFKGLKIALQADTGRWFTRCNNCQQTVGNNPDTVTVHVEGSVSEHPYAQFEVVDVGNGKI

ALKADTGKYVGRCNGCIVGGAYPDFLTIHVDDPSMPWAQFTPERLANGKYAFKADTGKYF

GRCNGCSPTSAYPDTVAVHVDNPHNSPWAQWTVSYVPFSYLERYDAIPADNVAEKAKLVG

LAMATDSRNFFKELRANRPIFITPKFALITLFPDVQEVFSRPEVFSVRLYAPKMDPNHGP

AMLSRDNTVYNWREKSIMKTMLDWEDLPRIKQAAGEVAKAALDKFAPTKKIETVNELAKW

VLVRMSGDYYGFPGPDRETMYRWSSATQSGMLRNLANDPQIHEASVQAGKEMRDYLTQLL

QQKKANNTPSTAPKDIFTRLVQANLTSDIPFDESRILTNMALLLISTLDTTAQAIVQSLE

QLLRRPDILPKAVAAAKANDDVTFAKYVWEALRFNPVSPALPRFCESDYTVAAGTSRATR

IPANSLVLVSLGSAMMDGAIVQNPEQFSIERPKHNYMHYGYGDHTCLGEHIGNVVVPEVI

KQVLLRPGVRLIPGDEGKLQAQPNAILKSFVIAYDG

>CYP110E29(AFZ26509.1)Cylindrospermum stagnale

MKLPPGPQTPALLQQLQWIFKSVEFLDECGKHYGDVFTVQVFDSKKIVFFSNPQAIQAIY

TDAAKQFESGRENGIFSPVVGDSSVILLDGDRHQRQRQILLPPFHGERMRNYGKLIFDIS

QEVTNEWVIGKPFSIRPYMQEITLEVILRAVFGIQKGERFQQLKSLLKKLLLSISSPSYS

VIFFLPALQRDLGAWSPWGNFLRQRQQIDRLIYAEISERRQQFDKSGTDILSLLMAARDV

DGEPLTDIELRDELITLLLAGAENTVSALEWAFYWIHQQPQVYQSLMEELNQADDSEPSE

LAKLPYLTAICQETLRIYPIALVSLPRIVTLPIDLMGYQLEAGTLLYACIYLVHRREDIY

PEHQKFIPERFLQRKFLPYEYMPFGGGNRGCIGSAFVLFEMKIVLASILSRWQLSLTNHL

PQRPIRRGITTATASDVKMIATSKF

>CYP110E18(AFZ26515.1)Cylindrospermum stagnale

MQLPAIPQSSKFIQRIQWVFNPLQLMETSARVHGECFALCLTSEQPIVLFSNPQAIQEIF

TASLETFDAGKSNQIIKPLLGEKSLLLLDGVSHQSQRRLLTPPFHGERMKAYGQTIANIT

NQVISNWKIGTPFSVRSAMQEISLKVILQTVFGLHEGERFAQLEERLRSLLNLSASPMRA

SMLFFSALQVDLGAWSPWGKFLRQKQQIEKLLDLEIQERRDHPDASRNDILSLMLSARDE

KGEPMTNEELRDELMTLLVAGHETTASALTWALYWIHRLPEVREKLLAELDNFGDNADLS

EFARLPYLTAVCQETLRIYPIAMITSPRIVKTPMKISGYEFQPGIMLAPCIYLTHRRPDL

YPEPQQFKPERFLERQFSQYEYIPFGGGNRRCLGMAFAMFEMKLVLATVLSNLEFALVDN

IPVKPIRRGVTLAPSGGKWLVATRQRQRVGIPVGV

>CYP1156A2(AFZ27762.1)Cylindrospermum stagnale

MHTPITPLMPPEHLGFEEDNQSVLAKPQSVPTYDLFAPEVIANPYPLYKRIREENPVYYE

ASLGYWILTRYQDVEAALRDERLSSERRTLFIQQLGNLDVSLIQNFLQLTDNSMIEKDPP

EHSRMRKLANQGFTTRALESWRSIIQKTTDTLLDQVQHQGYMDIVSDLSVPLPSLIIAKI

FGVPESDRAHLIHWAKDISSFWGAPSGKNIEELVRKADTSAAQFTAFINQLVAERRQKPG

TDMISLLTMAYTEQNLDFAELPSLCILILNAGRSTTTDLIPNGVSALLNHPQQLQKLKDN

PALISSAIEEIIRYDSSVSFVFRIAKEDIEIGGQKIPAGSVIALGLAAANHDPAKFTLPD

NFDIERSPNEHLAFGPGIHFCLGAVLARMELNICFSTLLKRFPNLQFDSTKPPLPRRNTF

VFKGFDALPVRF

>CYP197E3(AFZ23787.1)Cylindrospermum stagnale

MVLASKAKIDPNYPPGPKHHWLMGVVSEYTRNPIGFMSECAKEYGDIVYWQWPLLSFYQL

NHPDHIEEVLVKKNNLFSKHLSLQILQRMFGNGLLSSEGDFWQRQRRLTQPAFHRDRIFS

YGEVMVDYTNRLLTNWSDGKIIAIHEEMMHLTLEIVAKTLFGAEVTEVETVEKIMQISMA

YFDDRNNNFLLFVIPDWVPLPHNLRFQKAAQQFDEIIYPIIQRRRESGEDQGDLLSMLLQ

MQDENGNRMSDKQLRDEAVTLFIAGHETTALAISWGWYLLSQHPEIEQKLHVELQTVLAG

RTPTFADLPQLPYTDRVIMEIMRLYPPAWAMVRTALEDCEIAGYPVRAGDSMIMSQWIMH

RDSRYFDQPEVFNPDRWEGDLAKRIPTFAYFPFGGGPRICIGQSFAKMEAVLLLATISQK

FRLTLMPDQEITPWPAFSLRPKYGMKMLLNQR

>CYP120B4(AFZ24004.1)Cylindrospermum stagnale

MKNDLTPPGSFGLPLFGETFAFAADPYIFVNKRYQKYGSIFKTNIIGRPTVVMVGPQALE

FFLSSHMESFSWREGWPNNFKVLLGESLFLQDGEEHRRNRRLIMPALHGPALANYADAMV

DITDKYLKKWEQKQEFTWLEEFKQLTFDIASQLLLGTNPGAEAARLSRLFENLTNGLLAI

NPLSLPFTKFGKAIAARNQILEHLTKVVRQRQLNPTNDALSLLVQARDEEGNCMSEKELI

AQAVLLLFAGHETTTSMLTWLCLELARHPEVMQRAREEQLQLASIGALSLEQLGQMPYLD

QVLWEVERRNTPVAGGFRGVIKEFEFNGFRIPAGWQLYYSIFMTHQLKELYPDPERFDPE

RFSPQRQEHRQHPFSLIGFGGGPRICVGIAFAKMEMKIVAAQLLRRYDWGILPNQSLTPV

RIPINHPKDGFKVRFQPR

>CYP110AG1(AFZ24837.1)Cylindrospermum stagnale

MRNSKKHPQLRRPDGPKNPRLVQMLQWIADPIGYLETASKHYGDIFTAQVGWGVAPHVFV

SNPQAIQQILTSESKQFSPYSELFINFMKPFLGEHSIMRVEGDRHRRQRQLLMPPFHGER

MRAYGAQISSITERVMSRLDQSKPFKARNVMLNISLEVISQVVFGLQSGERSERLKQLLN

AWLDAMSSPAFPIFLLLPFLQKDLGARSPWGYIQNRNKALSELLYAEIRERRQKYDPSDN

DIMTLLLSAKDEAGVGMTDEELYDELLTLLLVGHETTATAITWALCWVHHQPEIRNKLFE

ELDTLGDSSDPMAISRLPYLTAVCFESLRIYPVLPLNLPHVVREPVELMGYQLEPGTKVV

ANIYLTHHRQDIYPEPERFKPERFLERQFSAYEYLPFGSGSRRCVGAALAQLEMKLVLAT

ILSHYQLALSDSRPVRPQLQFGSFIVPQGGVKMVIEGERYRVAPKAGKSDVKTENLI

>CYP120A21(AFY64957.1)Geitlerinema sp. PCC 7407

MTATNNVKPVPPGSFGLPVIGESLAFVTDGKFAQKRHQKYGDVFKTQIFGQPTVVLKGAD

ANRFVLTQENQSFEVTWPTSTRRLLGNASLALQHGAEHQSRRKILYQAFQPRALASYAET

MLAITQRYVRAWTQTGELTWYPELRKYTFDVACKLLVGVDNASQTRLCDDFETWCAGLFM

LPIELPWTRFGKAMKARSQMMVELERLIRQRQQMPASQTDALGLLLSAEDENGDRLGLEE

LKEQVLLLLFAGHETLTSAIASFCLLMAQYPEVFAKVRAEQESLGVDLPLTFENLKQMTY

LEQVLKEVLRLIPPVGGGFRRVLKACEFQGYQIPEGWTVLYQIGSTHSDGQLYPNPDQFD

PERFGSEAGSPAFGGYVPFGAGLRECLGKEFARLEMKLFAALLAREYTWELLPNQDLSLA

AVPTPHPQDGLKVRFGRR

>CYP120A14(BAQ61940.1)Geminocystis sp. NIES-3708

MTINSNLQKSPLPPGSFGLPLIGETINFLFDRNFSSKKIAQHGNIYKTSIFGNPTVIMIG

AQANEFLFRHENEYVVSTWPKTTKVLLGQTSLAVKNGDFHTSRRKILAQAFQPRVLASYL

PTIEAITANYLQKWAKLGELTWYPELRNYTFDIAAKLFVGVDNGSQTRLCNLFEEWCQGL

FSIPVNLPWTKFGKALKCRENMLQEIEAIVKQRQAGDNTHSDALSLLLKAEDENGDRLTL

EELKDQVLLLLFAGHETLTSAIASFCLLTAQHPQVLAKIRQEQQKLNYQVPFNIEQLKEM

TYLEQVLKEVLRLIPPVGGGFRKAIEDFSFQGYHIPKGWTVQYQILQTHQDETIYHDKDK

FDPDRFSPENSLEKQKKFSFVPFGGGLRECLGKEFARLEMRVFASLLSHNYQWELLPNQD

LTMAIIPTPHPKDGLKVKFSTLLQN

>CYP120A16(BAQ64702.1)Geminocystis sp. NIES-3709

MNEKSDLQSLPLPPGDFALPFIGETLNFLFDRNFYQKKIAKYGNIFKINIFGNPTVTMIG

GEANEFLFRHENQYVVSTWPKTTKVLLGKTSLSVKNGDFHTSRRKILAQAFQPRVLATYL

PTIETITQNYLKKWATLGEFTWYPELRNYTFDIATKLFVGVDNCSQTRLCHLFEEWCEGL

FSIPVDLPWTKFGKALKCREEMLQEIEMIVKQRQKEGNVKSDALSLLLTAEDENGDRLTL

EELKDQVLLLLFAGHETLTSAIASFCLLTAQHPEVLAKIRQEQKTLNYQPPFSLEQLKEM

TYLEQVLKEVLRLIPPVGGGFRQAIQDFSYQGYHIPKGWTVQYQIAQTHQDKTLYHHKDK

FDPDRFSPENSVEKQKKFGFVPFGGGLRECIGKEFARLEMRVFASLLTQNYQWELSPNQD

LTMQLIPTPHPKDGLKVKLTMN

>CYP110C31(BAQ66208.1)Geminocystis sp. NIES-3709

MSQINPLTTSVIQQKIKWILDPVGYLKNAYNQHPDIFTATVSGLGSGSLVFVTHPQAIQQ

ILTNDRQQFFANGQLNNILTPVVGFSSLLSLDGENHKRERKLLMPSFHGERMQIYSNLIT

EITENIFNQLKPGEVFIARELMQEISLQVIVKIVFGLSEGDRFEKMKELIKAILDRFNNP

INISFLFYDWLKKDFGAWSPWGGFIRTRMQLDELIHSEINLRRQENNTNRTDILSTLLTA

VDEEGKGMSDQELRDELMLMLFAGHETTAIAMTWTLYWLHRQPDIKTKLLTELSENHDRR

GETIFKLPYLTAVCNETLRIHPVAMLTFPRQVMRDTELLDQKIPKDTVLLGCIYLTHHRE

DLYPEADKFIPERFLNRQYSPYEFMPFGGGVRRCLGEVLALYEMKLSIAHIITKYDLKLA

ENKTLKPKRRGVVLSPEGGVKMIFNEKKQ

>CYP107NL1(AGY57714.1)Gloeobacter kilaueensis

MSRLESVQIASPAFKADPYPFYARLRAETPVYRVALPNKQPAWLVTRYDDVAALLKDPRF

AKDRQNALTKKQMASQPQVPALFAPLTRNLLDSDDPDHARLRRLAQTAFTAKRIEVIVDR

TQAACDERLDRLKGRRFDLMGEFALPVPVMVISELLGVPQADREKFGRWSKTLSQNTMTP

MRMLLSFPHLVCFVRYLRRLIAQKQKQPQDDLVSALVQVQATGQLDEDELLAMVALLLTA

GHETTTNLIGNGMLALLRHLDQFERLRAQPELIETAIEELLRFESPVEMSTNRYAREDLE

IAGTPIARGELVLGVIASANRDESQFPDAHTLDIARRPNLHLAFGQGGHYCLGAALARLE

GKIALETLLRRLPNLRLTQATNSLRWRRGLVLRGLESLAVSF

>CYP120M1(AGY59171.1)Gloeobacter kilaueensis

MQVEQMPGDLGLPGLGRLWQVVATEGFGMLSDYRRYGPVFKSSFLGRHCAVLIGPQANRR

VLIEAGDQLSSYEGWGPFTEHVFGQPMMLQDGERHRRTRRLMAPAFHGAAIATYSRTMQQ

IFKQGFTSWTEQRSVPIHQECRKLALVIGIRLLLGVEAEAQVEQIERWYSALLAGTTALL

RLEGPLTAYGRARFAREQLQALLGRIVAERQRRGGLEDSADALGLFLAAVDEQGEPLDRA

QVVDELVHLVNGAHFTTATALTWALVELAARPGWRERLRGELERVTGSEPLDVAHLRQLV

QMGWFLKEIERFYSPAGAILFRGVRQPFEFGGYVIPAGWLVAVSPFVSHRMAELFADPDH

FEPERFAPPREEDRQDPLALVGFGAGPHVCIGREFALMELKIALAILLRDYDWTVAPAEQ

AVTPRLFPARTRDRYRARFVKRTAPN

>CYP110E15(AGY59321.1)Gloeobacter kilaueensis

MSLPAGPASPSPFQLMRWLGRPTEYLEATRGRFGDAFTMRIGVFPPLVLLSDPQAIQQLF

SAESGTFDAGASNQALKPTLGSNSLLLLDGERHQQQRKLLMPPFHGERMRSYGELIRQLT

SQVIAGLQPGSPFLIRSTMQRISLSVILQAVFGLEGGARLRQLRRILSNMLDTMSAPLLM

GLLLLLPDDLGPWSPRGQLNRSLEQIDALLYAEIRERRQRPDPAANDILSLLLAARDERG

EPMSDVELRDELMTLLVAGHETTATSLVWALYWIHYLPEVYQRLRAELDGLGTDFDPEAV

ARLPYLNAVCSETLRIYPVALITSPRVVRKPVRILGHEYTTGERLTAAIYLTHHRSDIYP

ESDRFRPERFLERTFSPYEYLPFGGGNRRCIGMAFALYEMKLVLATFLSQLEMHLVHPRP

LLPVRRGVTLAPPDGLYLMPGTRRVCGRVPASV

>CYP107DX3(AGY59406.1)Gloeobacter kilaueensis

MTLIPGSSVHPDPREGEGFFLNSPQKLDNPFPDLQYFRENRPIFFYPPLDQWFVFKYDAV

NELLSDPRLSADRMKGFVDKVPEEVREEFKTIAPLLTMWVLMQDGEDHARLRNFLYLGFN

GTVVHDLKEQTQKSADELLDRVERQGYMDGSSDYGFVLTAYVLSDFLGVHKEDRDQVIQW

SVDFVDFFNIVPITVDTTRRLVRSTNGLSQYTRGLIAERRANPQNDFLTTLIRAENEGGH

FSDDEIVANAMLFLLAGHLAVRNLIGNAIYLLLTHPEQYRQLLAQPELLENAVEETLRYE

PPVILIPRIANEDFLFNGNRFRQGQLIQLSIASANRDADHFSMPDQFDITKKPGKILSFG

HGPHTCLGAVLARQEAIIALETLFRRFPTIKIAEDKQIQWYRNAGNRGPQKLPLVF

>CYP233A1(BAC89876.1)Gloeobacter violaceus

MSALPPPRFNPFDSEFRQDPYRVYAHLRVAAPIHRSLGMWVLTRYADVLAVLKDPHFSSS

QIPLAVRQRSERPDQAQSHPLARLAAKSIVFTDEPDHTRLRHLVVRAIKRRTPEQEQAHL

TRIASALLERVGPKGRMDAVADYAERLPLQFMAESMALPPDSWQTVRDWTHQLRYLLEPG

LMGRGDFERVQAVLDEVIAFFEDMLAVRRQQPGDDLISALDAAHREAQADRLSDEEIVYC

CIMMFVAGHETTRSLIASGLLALLQHPEQLAYLRMHPERMGAAVTEMLRYESPLQQTKRR

ATAAVAVGGRTIQPQEQVLLCLGAANRDPARFEQPDRFDITRTDNGHLAFGQGMHHCLGA

ALAQMEAQVALRVLLERFANLTLQDTPEWLEHSFILRGLKTLPVQWDR

>CYP1011B1(BAC89883.1)Gloeobacter violaceus

MDSVANLNQDAFGNTLPQTEAPFKFNVFDPAFHEDPYPFYDRLRRESPIYRNFMGAWVFT

RYSDIKSILRDRRFRVLDKPGWIKNKNRYLTPDQGNFDALVRSSSKFFFFLEPPDHGRLR

GLITKAFSASFVDRLRPHVEATLADLLGKVREQGAMDIMADLACPLPAIVIARLIGVPAA

DYARLGHLSDELARIFDPVISLEGYLHLNAVVEEFGSYFLDLVAEHKRQPGTDLIDSLIA

AQEEGNRLSEEEVVAVCMQLFAGGEETTVNLIGNGMLALLTHPEQLELLRSKPEIIAGAV

EELLRYDSSIQLVARAAIEDIEIEGCTIGAGEHVHLYLGAANRDPAQFFDPHSLDLTRVD

NRHLAFGDGIHHCFGGPLARVEGQVVFQTLVQQFPKLRLAESRRPERREGTLLRGLKTLP

VTF

>CYP110E4(BAC91004.1)Gloeobacter violaceus

MSLPPGPSSPSPFQLMQWIGCPTDYLHTTAARYGDPFTMRVGVFPPLVMFSDPRAIQQLF

TAEAGTFDAGASNVALRPTLGANSLLLLDGERHQQQRRLLTPPFHGERMRAYGELIRQVT

EEVIVRWQPGKPFLVRNAMQRISLAVILQAVFGLHDGTRLVRLRQALGSMLDAMSSPLSM

AMLLMLPEDFGPWSPRARLQAHLGAIDELLYAEIRERREHFDAGAGDILGLLLAARDEAG

AAMGDAELRDELMTLLVAGHETTATAMAWALYWIHYLPQVRERLLAELDSLGSDPDPEAI

ARLPYLGAVCSETLRIYPVALIASPRVARHTVRILERDYEAGTRLAAGIYLAHHRPETYP

EPERFRPERFLERTFSPYEFVPFGGGSRRCIGMAFALYEMKLVIATVLLERDLRLVQPRL

LRPVRRGVTLAPPEGLYLVPTGERSASRLLSRTSTAGQ

>CYP110E5(BAC91005.1)Gloeobacter violaceus

MSLPAGPASPPPLQLLQWIGRPTDYLERTARRYGDPFTMRLGLHSPVTGVFFSSPEAFQQ

LFNTEPGLFDSGGANASSTFNLLFGTNSLILLDGERHQQQRRLLTPPFHGERMRSYGELI

RTLAEQVTARWNLGTPFQARRSMQRISLGVILKAVFGLHDGTRYLRVCRLLGNLIDASAS

PLLFGLRLIFPQDAGPMSPMGQLKAQIDAIDELLYAEIRERRERPDPRADDILSLLMAAR

DEAGQGMGDVELRDELMTLLVAGHETTATAMAWALYWIHRLPQVRERLLAELDSLGSDPD

PEAIARLPYLGAVCSETLRIYPVAMVAFARVPRRPVRILDREYPAGTFLIPNIYLAHRRP

EAYPDPERFRPERFLERTFSPYEFVPFGGGSRRCIGVAFALYEMKLVLATVLSRVELRLA

DPRPRLPVRRGLTLAPPEDLHLIPTALRSGHRDLLPAC

>CYP120B5(AFZ28793.1)Gloeocapsa sp. PCC 7428

MSHYRLPPGQTGLPVIGESLSFRFDPHFIEKRYQQYGPIFRTQIIGRPAVFMIGPEAVEF

VLSSHMDHFSWREGWPDNFKLLLGESLFVQDGEEHRKNRRLIMPAMHGAALESYFGAMET

LTQQYLQKWQQKGEFVWYEEFKQLTFDIASQLLLGTNTGLEAARLSQLFTALTNGLFTIN

PLPLPGTKLGKAIAARNQILQHLAQVVKERQQNPTKDALSLLVQARDEDGNKMSDRELVA

QAMLLLFAGHETTTAMLTWLCLELALHPEVLQRARNEQFELAQQRFSIEQLGKMPYLDQV

LAEVERLHPPVAGGFRGVIKPFEFHGYYVPAGWLVSYSIKLTHQLPEIYLEPQHFDPDRF

SPQRQEHKQRSYSLIGFGGGSRICVGIAFAKMEMKVIAAHLLRNYQWKLLPHQNLEDTRF

PTSRPKDGLRIQFQCIR

>CYP197B3(AFZ29452.1)Gloeocapsa sp. PCC 7428

MEQDIFELPGPEGKALVGNLLDLSQDPLGFLTHCAREYGDIVPIRLGLTPTCLLTHPDLI

EEVLKDRDSFIKSRGFRALRTLLGEGLLTSEGDSWFRQRRLAQPVFHQKRIAGYATIMVE

YAERMLTTWQNGETRNVHADMMRLTLNIVMKCLFNQDIDEGNAQVVANALDVAMDWFESK

RKQNFLIWEWFPRPENIRYRNAISQMDATIYSIIEQRRTSGEDPGDLLSMLMQARDEDDG

TGMSDRQLRDEVATLMLAGHETTANALTWTWMLLAQHPEVLSKLEAELQQVLDGRSPTVA

DIPQLRYTDMVVKESMRLYPPVAIFGREAAVDCQIGGYSVPKGCTITISQWVTHRDPRYF

EDPETFKPERWVDDLEKQLPRGVYIPFGDGPRVCIGKGFALMEAILLLATIAQKFSLNLV

PEFPIVPQPSITLRPEYGIKVVVKRR

>CYP110D20(AFZ31581.1)Gloeocapsa sp. PCC 7428

MDNQILDGPSPSFIQRRINMLRWIFNPLAILEKRYQQYGDVYVVAKNVTPLVVYISNPEA

LQQIFSAPAGTFDSSGANRVLLPLLGDRSLILLDGVAHQRQRKLLMPPFHGDRLKAYGEI

IRDITQQVISRWEVGKPFNVRASMQEISLRVILSAVFGLHQGDRFEELRKLLTRLLDAVG

SPISSMLLFFPSLQKDWGVWSPWGRFLNLRSQIDQLIYAEITARRAADVQSDDILSLLLQ

ARDEHDQPMTDRELRDELVTLLFAGHETTASALSWALYWIDHLPEVREKLLQELDTLPPD

ADPSAVVRLLYLNAVCCETLRIYPIAINTFPRIVRSPIEIMGYHFEPGTLLLPSVYLTHH

REDLYPEPKRFKPERFLERQFSPYEYLPFGGGNRRCIGLAFAQFEMKLVLATILAQYDLQ

LATKTPIKPTRRGLTVAPSGNLRMVVKQVRVQKTPALV

>CYP1007A3(AFZ31725.1)Gloeocapsa sp. PCC 7428

MTMVTDNRVLKVPGPQPSKFLGRAANVFHFAKDSVGYTRQLFETYGSIVSLADGGGTNVY

SPLPDCPGTVCVYGSEFVQQVATRHEIYYKYPLSGRMYRRRNSSKRTEPLKHFGVGLFGV

NSNQHRQHRQLMMPAFHKQRIESYRDDIVDITQSVLKQLPVEVPCDIAEIMRLLTLRVAT

KTLFGEDIGSGGGTGRLLQDVLALLGTPKVVLLPFDLPGFPYHRLLNLMAQLDDEMRAII

QHKRSTNSAERDVLSMLIQARDEESGTVLNEDELLGHTGVLFAAGHETSSNALTWTLFLL

SQHPQVAADLYDELAGKLKGEAPTVEQLQQLPLLERVIKESMRILPPVPWNGRVTSTTTE

LGGYTLPAGTEVFVSIYQTHHMPEVYSEPEVFNPDRWQKIAPTAFEYNPFSAGSRTCIGA

AFAMMEIKIVLAMLLQQFRLQCIPQTKIDRTGLIVMAPKYGMPMIVHKQDGCFTQAVDSI

RGNVREMVKLPS

>CYP120A18(AFZ32049.1)Gloeocapsa sp. PCC 7428

ENRYFSDGVSASAPRHVKLLMGTGAIVMQTGDKHLQQRKLLAQAFQPRSLAGYINTMAAT

TCNYLDKWEHMTITDKMRSLPVPPGNFGLPVIGETLSFLRDPNFIQRRQQQHGNIYKTHV

FGRPTVVMIGAEANRFLFSNTGNLNWYDELRKYTLDVACKLLIGIEANNDFGKLYENWGQ

GLLSIPLPLPGTKFSKALRCRKLLLAKIEAIILERQQQSTTKQDVLGLLLQARDEEGHGL

SLQELKEQLLTLLFAGHDTLSSSLTALCLLLAQYPQVKEAIFAEQKQLGLEQPLTLDLLK

QMTYLEQALKEVLRLYSPASGPRKAIESCEFNGYLIPEGWQVFYHPAATHQDSSIFTQPE

RFDPERFAPPRAEDKQKSMSYIPFGGGVRECIGREFAKLEMKLFAALLVRNYNWELVPGQ

NLNMVMLPTPHPRDGLKVKFWRRG

>CYP110F6(AFZ32474.1)Gloeocapsa sp. PCC 7428

MRVNFKTQTPALLQTLQLISEPTKFLESCMQRYGDPFTVRVLGLKSPPVVFFSSPQAIKE

IFALPGDQFDFKKATHVFKPLMGEKSIILQEGRSHQRQKQFMMPPFHGDRMKAYGEIIAQ

ITNHVIDQWSVGKIISLQHEMSDITLQIILQVVFGISPGTRYDKIKTLLGSLLDDVTKPL

FSSLFFFPPLQNDLGAWSPWGKFLRRRQAIDELIYSEIAQRRKEDDATRSDILSMLIAAR

DENGQPLTDIELRDQLVSLLLLGYETTAAALSWAFYLIHSSPQVLAKLLQELATSSVNPE

DIAALPYLTAICQETLRIYPIGLICTPRMVRDSAQIVGDTFDAGTIVVPCIYLAHRRPET

YPQPEEFLPERFLARKFSPYEYLPFGGGIRGCIGVAFSMYEMKLVLATILSRFHLALADS

RPARPVRRGITIVPSVSGMNMIVKAQQPAKVPVKM

>CYP120C2(AFZ33270.1)Gloeocapsa sp. PCC 7428

MVVQTKPASQFQSAEEMPGSFGKLFGGETKELFRDEELFYWEHFQRYGSVFKSRIFGKNF

AFLIGPDANRLVLGEKADHLSARLGWIFLEPIFGKGLLLQDGAEHQATRRLMYPAMHGRS

LTNYFDTIQEIVDKFFADWTAGKTISLIEEFTNLSTTIAIRLILGTETDSEFAEATQYFL

AMLTGRRAKLKIDIPQTLYGRSQQARRNLQAFLRRKIAQRKQQGSLQESRDFLGLLLAAV

DENGNSLSESDIIDQLLMVLFAGHENPAVLLSWLMFELVAHPEWRDRLRNEYAQVVGNEP

LNLSHLKQLPLTSYALKEVERLYPPVQNISRGVVKDIHYAGYCIPAGWYVDISPLLTHRL

PEIYTDPDRFDPDRFAPPREEDKKHPFALVGFGSGPHSCLGWQFAQMEMKIILSKLLRYD

WSISPEPSTAFPVRQPSQFQDSLQAHIKPPA

>CYP120C2(ASC70519.1)Halomicronema hongdechloris

MHQRYGKVLRFWMGSSDLMVSISDQDILSQIASTLHSRPKAAKKALGWLGHESPTFKSHE

ELRTIRSKVMPLLMGESLKYLCLVGQERTKRMLDGWKTATDAVEVASDFSEITFDIIGVA

LFGQEFSSTDLGQKFKKLFVHVLREAHPRSEEVIPSFWDPKYWQWRKSISRLQDCAEQLI

KQRRQALNMNKRKDLLSLVLSEKDGSGNPFFSDEQARATIVTFVFAGFDTSASSLAWICY

LLSQHPEVQARAQDEVDKVLAGRLPEFEDLDKLNYLTCVVKEAMRLYPPVPEALRALESD

LEVGGYSIPKGATFVIPISMLHEDEQIWEEPRKFLPERFTQENGKDHPRYAYLPFGTGSK

SCMGARFAMTEIRLVLAMMLQRFSLQLVPEQEVIPEMQSIILQPKYGLRLNVVTRNMNGT

RGQTLEYSASAGVANA

>CYP1159A2(ASC72620.1)Halomicronema hongdechloris

MVYIAANIPFYEVPIIMSYLEQYDNIPMDRLAEKLQLVNQWIRTEWRPFFQELRENRPIF

VTPKFTLVTLFSDVQEVLSRENVFTVKLYASKMDAAVEGPFMLARDNTEINWREKSIMKT

MLQPEDLPAVRKMAGDIAKTSLDNHAQAGEIEVVSQLGRYVPVRVCGDYFGFPGPDLESM

YRWSRATQTNFFKNLPNDPQIHEAAVQAGREMTTYLTQLLAEKQAQVTQTSPQNLSFGDL

LRALLDKLFSGNRSASSSTPPEPNAMDDVFTRLVKTQFADDILFDDKRIVTNMAGLLIGA

VETTSQAIVQALEQILLTPTILQEALQAAQANDDETFDRYVWEALRFNPINPLVFRLCEQ

DYVLAAGTPRETPIPANSLVFACTASAGFDAHELPQPETFSIDRLPYHYMHFGYGHHTCL

GKYVGMMQIPETIKQVLLRPGVRLLPGDAGKIDFQGTPFPERFVIAYDR

>CYP110AE4(ASC73385.1)Halomicronema hongdechloris

MSTLPASATPKLLQMLNWIFRPLAYMETNGHRYGDLFRARGLYNTVFVSHPEAIRFLLTN

DTNGIFTAPGETNEILRPLLGGNSLMLLSGQAHRQRRQLVMPPFHGERLKVYADLIRDIT

REAMIGFSPQTPFQARKLMQKITMRVILQAVFGLHQGDRYQRLQTLLAQRLDMASKPLAS

TLLFFPILRTDLGPWSPGGRALALAGEIDDLLYAEIRERRQALAADRNDILSLLLQARNE

DGNGLSDEELRDELMTLLVAGHETTATALAWALYWSHRDPAIKARIREEIAEANATDHPL

QLTKLPYLEAVCKETLRIYPVALLTFARRLEQPADLLGHSLEPGTTLMGCIYLLHHREDL

YPQPQQFRPQRFLERQYSAFEFMPFGAGARRCVGAALAMYELKIVLGTLLAEFDLSLSSD

QPVVPERRGVTLGMKGGVDMVFAGRRTPVASALV

>CYP110AS2(AFZ44527.1)Halothece sp. PCC 7418

MKQTIPSLETPPLLQTLELIANPIRFFAKYQHRYGDIFSARILGNNSPDVFFIGEPQALE

TIFTAPSGTFQLGKITHVFRPFTGDQSLIMLDGEDHLRQRKLLIPPLHGKRMSFYQNVIC

ELTEEVLPTLPKNQPFSSRKLMAQITLKVILRVVFGLKEGSRFQKLEQLISELLDAITNP

FYSSLFFFPPLQIDWGKYSPWGHFVRKQSAIDNLIYAEIQDRRQQDYSQQTDILSLLLSA

EDENGNGMSDQELRDQLITLLFLGHETTASSLAWMFYWVYSSPQVWTKLSAEMQPLGSSP

SPQALIDLPYLEAICKETLRLYPIALISQPRVVKETIQLHHQSFSPETILVPCIYLAHHR

EETFLDHHCFRPERFLENQFSAYQYFPFGGGNRACIGAAFSLYEMKLIFGTIFANLRLKL

ASQPAIKPVRRGITIVPSGGVMLVNQ

>CYP110A12(AFZ44529.1)Halothece sp. PCC 7418

MTITNSVTQQLPPTPQTSNFRQLIQWTADPLNLLEKSAQTFGDPFMLEFRKGRPFVFISH

PETIQEILTNDRANFDSGRGNYILIPIVGETSMLVTDGEDHNRQRKLIYPPFHGEKIRHY

GEIIAETTQQVTQTWDANKPFTMRSSMQDITLEVIMQAVFGISDGKRHQELKAPLVKLLE

LTGGSVLRSSLLFFPIFQQDFPGSPWRNFLNRQQGINKLLQGEIEERRNNQQTTGNDILS

LLMSAEDEDGNPMSDTELGDQLITLLFAGHETTATALAWAFYWIHKFPEVREKLLAELET

ISDSSDIKALNQLPYLDAVCKETLRIYPVAIITFPRITKSVVTVGNYEYPPEMFLAPCIY

LLHHREDIYPNPNQFQPERFLEKEFSPYEFMPFGGGIRRCVGDVFAMMEMKIVIATILKQ

YSLKLLEKRAVKPVRRGVTIAPKGGIKMALENY

>CYP110AE2(AFZ45049.1)Halothece sp. PCC 7418

MNTIPTPKEPALVQIAQWIFDPVRYMSKNFQKYGDLFQAYVSWGSSDPLLMVSEPKAMQY

MLTHDTTKQLTAPGDVNSILEPLIGRQNLILLSGKEHRRRRQLVMPPFHGERLQAYGEII

QQITQQVIAEWSTEEPVNVRNAMQKMTMRVILQAVFGLYEGERYNRLETLLSQRLNMTGS

PLGAVLLFLPWLRKDFGAWSPGGRIRQIAEETDRLLFEEIRERRANPDPNRVDILSLLLM

AEDEAGHGLTDQDLRDELMTLLTAGHETTATALTWAMYWIHSLPEVKEKLLAELDQVSNP

NDPSSFLKLSYLNAVCNETLRIHPVAMLTFPRRVEEPIELCGYQLEPGILIMGSIYLLHQ

REDLYPEPQQFRPERFLERQFSPYEFMPFGGGVRRCVGAALAQYEMKIILGTILSTLDLE

LLNQKPVAPTRRGITLGQNAQIWIQKIGQHSPQMAVPS

>CYP120B10(AFZ45737.1)Halothece sp. PCC 7418

MSEVSKPLPPGSLGLPIIGETLSFLLDRNFAYKREQEFGSIYKTNILGRKTIFMTGAEAN

KFILSSHMDHFSWGQGWPENFRKLLGESLFLQDGEEHRRNRKLLMPAFHGQALVNYTQTM

EEIIQKYFKKWHSQENFAWFTELKQMTFEIASVLLLGTTPGEQTERLSQWFTDLTNGLFA

IFPIEASWTKYGKAIAARDRLLDYLDEEIERRKSNPGKDTLGLMLQTSDENGDYLTREEI

KVQALLMLFAGHETTTSMLTSLCMSLAQNPNLLAKARKEQEDLGIEGELTLEKLKQMTYL

DQILKEVERLYPPVAGGFRGVVKSFTFKGYYVPKGWIVSYRITSSHQDSQIFSNPKTFDP

DRFSPERAEHKKKEYSLVGFGGGPRFCLGYAFAQMEMKIFASLLLRYCQWDILPDQDLTL

EPIPTLHPKSGLKVTFSSSLSV

>CYP110A7(BAS55657.1)Leptolyngbya boryana

MMSQLPNQITAPAWVQLFNWIVDPLGFLDKYTQKYSDVFTMQLAGLGLSVVIANPKAIQE

IFNQDAKFDMGRGNELAEPLLGRNSLLLLDGDRHRRERKLLMPPFHGERLHTYATQICKV

ADQVASQWQTGQPFIVRTSMQNISLEIILQVVFGLSEGERYQQLKVLLTEWLDMTDSPSR

SSMLFLKFLQKDWGAWTPWGRMQQRQRQIHDLLQAEIDERRTKGDEKRSDVLSLMMAVRD

ETGQAMSDEELRDELLTILFAGHETTATTLSWAFYQIHQQPEVREKLLKELESLDAHASP

MSMAQLPYLNAICQETLRMYPVIPVLFPRISKVPVKVADYSFDAETTLWISPYLVHYRED

VYPNAHHFEPERFLDRQYSPAEYFPFGGGSRRCLGYALAQLEMKLVLATILSKYQLALAD

DKPVKLQRRGFTLAPTGGVRMVLTEKP

>CYP120A23(BAS56690.1)Leptolyngbya boryana

MSSSLPLPPGSSGLPVIGETFGFLNDPDFSTKRHKQFGNVFRTNLFGRSTIVLSGVDAVR

FVLLNENQYFVISWPPSVKTLLGSASVPVQQGSLHQQRRKLLVQAFQPRALASYIPTMTS

ITQNYLDRWAQKETIRWYPELRNYTLDIACQLIVGISSGSQTRFGELFEIWVKGLFSVPL

KLPGTKFSQALTSRQLLLDEIERVVKARQSQENIESDSLSLLLNAKDEEGNRLSIDELKD

QVLTLLFAGHETLTSAIASFCLLMAQHPAILERLRAEQSQFKNDDLLTLEDLKQMDFLDQ

VLKEVLRVVSPVGGGFREVIQECEFDGYKIPKGSQVLYEIGQTHQDSTLYPNPKQFDPER

FNPEQKLEPFSHVPFGGGIRECLGKEFARLEMRIFAALLVRNYEWELVPGQNLDLQMIPT

PQPKDGLQVKFRRKAS

>CYP110C16(BAS58682.1)Leptolyngbya boryana

MVEGLMNLPNVLRAPSLLQTLHWVADPVGYMEKSAQQYPDIFSAPILGGKHLVFINHPQA

IQEILTSDRKRFIAPGEVNRILSPLIGDASVIMLDGDRHKRRRQLLMPPFHGERMRAYGK

LICQLTEKVMSQCALAQPLNARSMMQEISLQVILEAVFGVCEGERFEQLRASITQMCELF

RNPLGASFLFFPWMQQDLGTWMPWGRFLRARSNIDKLLYAEIADRRQQDLRDRVDILSLL

METRDEDGQPLSDSELRDELMTLLFAGHETTATAMAWALYWVHHQPEVREKLLQELDALG

ANPDPMSIVRLPYLSAVCNETLRLYPVAMLTFTRIVTEPVELLGYPLEPGMSVVGCIYLL

HHREDLYPNSHEFRPERFLERQFSPFEFMPFGGGARRCIGEALAVAEMKLVLATVLSQYQ

LALADSRPEVPRRRGVTLGPSGRVKIQILGQRTTRPSEAIVSG

>CYP110M6(BAS59442.1)Leptolyngbya boryana

MTLPIGSSTPALFQTLSLVADPIAFFDRQSVQYGDTFTTRVLGLNSPPVVFLSHPDAIQA

VFTTLADAFEFGKVTDVFRPLVGNESLIMQEGARHQRQRQLLMPALHREQLHSQGHLICQ

LAQKRMIDWNVGETIALRSEMSEISLQVILQVVFGLVPGARYERLKQLLAQLLEAITSPL

YSTQFFFPILQQNLGDWSPWGKFLDQMAQIDELIYAEIRDRRSHSLQNRTDILSVLMTAR

DEQGESMDDQELRDQLMTLLLLGHETTASALTWAFYWLHRNPNCLDRLRQELDEFGENPD

PIVLSQAPYLTAVCKEALRIYPIALISQPRKVKRTIEIEGYTYEPGTVLIPSIYLAHRRE

ATYEQADQFKPERFLERKFSAYEYLPFGGGSRSCVGMALSMFEMKLVLATVLSWYEFEST

LNREIRPARRGITFVPPDSFQLKVVRDRAIIPELIHNP

>CYP110M6(BAU11055.1)Leptolyngbya boryana

MNLPNVLRKPLLLQEWQWVADPVGYMEQSAQQHPDIFSAGLLSGQHLVFVNHPQAIQEIL

TSDRKRFVAPGEANRILSPLIGDASVIMLSDDRHKRRRQLVMPPFHGDRMRNYGDLIRRL

TEKAMSQYAIAQPFNARSAMQEISLQVILQTVFGVTEGERYEQIRAAITKMCDLFHNPLS

ASFLFFPWMQRDWGTWMPWGRFLRDRSEIDTLLYAEIADRHQQDNSDRVDILSMLMAARD

ENGEPMSDSELRDELMTLLFAGHETTATAMAWALYWVHHLPDVREKLLQEIEALGANPDP

MSIARSPYLSAVCNETLRLYPVAMLTFSRIVKEPLELLGYPLQPGTAVVGCIYLLHHRED

LYPESHQFKPERFLERQFSPYEFMPFGGGSRRCIGEALAMFEMKLVLATILSNYELALVD

RRPEVPRRRGVTLAPGTGVKIKIVEQKTGHLTEAIAQTS

>CYP2725A1(LBWT_19990)Leptolyngbya boryana

MEVKDLTQCPVKDIVNVYLSKSAMNRSKDPRQNRLFYSMSPVGHRFLENIDSEDPQFEAI

SSLAKQVSKVLESNCKQGFENRLKAYWASNLPAYPVCSIRDSAFGAVLDEVWFAFFARAC

PNPDLVVKAAHNILMVVKNRETVDIKSRRNAIALVADAVKLGLNPQLELQEIELQETFTN

IHEVIFALIVSSVDELSEGFAHTVIALAQHAPTPTSLDEFALAFYEALRLYPLFTRSTRP

SNDNTCLYSLNYVNYHRRTDIFGKDALQYNWQRWQAKNWLGQTFIFGVASNRSCPGRGSA

IEMVPRMVQVWMQTYQTQSYIRHTRNLPCGGLAIVTPIGKRVPWFLSWLHFGAWIYSHAS

FCLIQRAWVNHQNAKASNKRREADHYYASLKALRIPAAASHGSVQPSRLSAKS

>CYP110AL1(LBWT_X2040)Leptolyngbya boryana

MPDLPIGPEVPTLFWQLQGILNPLSLLREAHDRYGDVFRLPYTKFTAICVSSPTGLQSIF

SASPEVLSSHQRGGIFDLILGQNALVFLEGREHQRHRRLIVPSFHGEALNQWGRDICSTT

KYVFDQPQYQTVLPLRQPLKEIALRVILQVLFGGLRTPLLRELYHLLYSFFQDVESPLSA

VGMLFPALRVDLGSWSPWGRFLQQKQQINQLIQQQIDRQRTSTLLEAHPSVLAMLLQVRD

EFDQPLSDDEIRDELLMLVLAGYETTTSAIAWALYWIHHDLTVQEKLRQELEGIDDPMEI

ARLPYLSAVCHEALRIYPVAIGCFARQVLQPFSIDGYELPIGTVISPSIYLAHHRTTVYP

DPDTFRPERFLSQQFSAYEYLPFGGGSRRCVGGEFTKFEIKLIVATTLKQIRLQAFDSKP

VLPIRYGITMAPPVDLKLKVLPR

>CYP120B9(BAU13991.1)Leptolyngbya sp. NIES-3755

MPPLPPGKFGLPLIGETLEFLIDPKFVEKRYHKYGAVFKSHILGKPAVFMVGAEAVEFLL

SSGFDNFSWREGYPETFHKLLGRSLFAQEGEEHRRNRRLIMPAFHGAALARYFEIMDKLI

CKYLVKWEQQYEFKWFDEFKQLTFEIASQIFLGTDTSDEAKRLSDSFATLTAGFFSFPKL

PGSRFHQSMKARQALLDHLDQVIDRRRKQPTDDALSLLIQAEDENGDRLSHKEVRDQALL

LLFAGHETTTAMLTWFALELARHPDVLEKARSEQNQFDHPITSDQLTKMPYLDQILNEVK

RLYPPVPGGFRGVIKPFEFSGYHIPQGWLAQYSILFTHRLPELYPNPETFDVDRWKDTKQ

KPFSLIGFGGGSRICIGLAFAKLEMKLIAAHLLRNYNWELLSNQSLKPVLIPTCRPKDGL

KVQFQKL

>CYP110M5(BAU15584.1)Leptolyngbya sp. NIES-3755

MMLPPGLRSPALVQTLSLVADPIAFFDRASAQYGDTFTTRVLGLNSPPVVFLSDPDAIQA

VFTTLADAFEFGKVTHVFRPLVGNESLIMQEGARHQRQRQLLMPALHREQLHSQGHLICQ

LTQKRMIDWNVGDAIAVRSEMSEISLQVILQVVFGLVPGVRYERLKQLLAELLEAITSPL

YSTQFFFPMLQQNLGRWSPWGQFLEQMAQIDELIFAEIHDRRSQSLSNRTDILSVLMMAR

DEQGESMSDQELRDQLMTLLLLGHETTASGLTWAFYWLHRHRESLDRLRQELDQLGENPD

PVVLSQAPYLTAVCKEALRVYPIALISQPRKVKCTIAIEGYTYQPGTILIPCIYLAHRRA

KTYEQADSFVPDRFLERKFSAYEYLPFGGGSRSCVGMALSLFEMKLVLATVLSWYEFETN

LDRAVRPARRGITFVPPDTFRLKVVGDRATIPELIHNS

>CYP110M5(LEP3755_61430)Leptolyngbya sp. NIES-3755

MMLPPGLRSPALVQTLSLVADPIAFFDRASAQYGDTFTTRVLGLNSPPVVFLSDPDAIQA

VFTTLADAFEFGKVTHVFRPLVGNESLIMQEGARHQRQRQLLMPALHREQLHSQGHLICQ

LTQKRMIDWNVGDAIAVRSEMSEISLQVILQVVFGLVPGVRYERLKQLLAELLEAITSPL

YSTQFFFPMLQQNLGRWSPWGQFLEQMAQIDELIFAEIHDRRSQSLSNRTDILSVLMMAR

DEQGESMSDQELRDQLMTLLLLGHETTASGLTWAFYWLHRHRESLDRLRQELDQLGENPD

PVVLSQAPYLTAVCKEALRVYPIALISQPRKVKCTIAIEGYTYQPGTILIPCIYLAHRRA

KTYEQADSFVPDRFLERKFSAYEYLPFGGGSRSCVGMALSLFEMKLVLATVLSWYEFETN

LDRAVRPARRGITFVPPDTFRLKVVGDRATIPELIHNS

>CYP120A26(BAU42683.1)Leptolyngbya sp. O-77

MGEKGDDYQVAVDRRVVDALLQFPIFLMNTLPPGTFGLPFLGETLNFFTDPNFAQKRHEQ

YGNLFKTRLLGKPTIFMRGVEANQFVLSNENTYFSVDWPPSTKALLGKLSLALQTGHEHQ

SRRKLLAQAFMPRALSGYIRTMETITQRYTQRWQQQGELTWYPELRNYTLDIACKLLVGL

DKGSQTRLGHVFETWCQGLFSIPLNLPWTAFGKAKRARRLLLQEMEQIICDRQQRLSQSL

NSSDGPESDGPEASDALDLLIRARDEDGQSLSLEELKDQVLLLLFAGHETLTSAIASFCL

LTAQSPDVLEKLRAEQRGFDPAAPLSLDLLKQMTYLEQVLREVLRRIPPVGGGFRTVLQD

CTYGGYTIPKNWSVLYQIGPTHQDASLYPQPERFDPERFSAAQLGDRSADQQRYGYVPFG

GGIRECLGKEFARLEMKIFATHLLRHWQWTLLPDQDLSLVVVPTPHPRSGLRVAFTALKA

G

>CYP110AF1(AFY38256.1)Leptolyngbya sp. PCC 7376

MAIATDLSKTIPMLPQPKLLQKFRVILNPTGYLQEVLDYAPDLGYMPSAGYEQPLILVHH

PQALKEMLVDNRKVFTAPGELNSIIEPLTGAHSLLSLSGDRHRRARKLIMPSFHGERMYN

YGTLIQQIIREEVERLPINQPFLAVELTQRITLRTIIEVVFGIRSGDRYEPIIELTRGIL

NRFQSPAATSFLFFSSLQKDLGAWSPWGNFVRARDALDELIYAEISDRQANPDPTRTDIL

SLLIQARDEDGEAMTPLELRDELMVLLFAGHETTAISMAWSLYWMHVQPDILQKVRDELE

ALGDRPDPMTVYRLPYVAAVGNESLRINPVAMFTFARLATQTTQLLDYEIPADSILMGCV

YTLHQRPDIYPNPREFRPERFLDKTFTPYEFMPFGGGDRRCVGEALAQFELRLGIASFAT

LGKFELLETEPVIAQRKGLVLSPKNGIKMQFLGT

>CYP120A8(AFY40660.1)Leptolyngbya sp. PCC 7376

MTSPNELPLPPGKFGLPFIGETIEFFTDRNFQQKRLDEHGDVFKTNIFNKPTVVMVGAEA

NQCLFRNENKYVKATWPKSTRILLGSSSLATQEGGVHSSRRRILFQAFQPRALESYIPTI

EKITQRYLDKWEQKKEFAWYNELRKYTFDVASTLFIGKDGGADTPLANLFEEWVQGLFSL

PINLPWTTFGKAMKCRTQLLKELEVIIGDRLANQKSDDQPTDALDLLIRAKDEDGNALSI

EELKDQILLLLFAGHETLTSSLVSFCLFVGQNRNVFEKICAEQTALDISGELDMNTLQQM

TYLDQVFKEVLRIVPPVGGGFREVIQTFEYKNFQIPKGWAVQYQILQTHKDEENYPDHER

FDPERFSPERAAEKQKNYQFIPFGGGMRECIGKEFARLEAKVLGSMLVRGYDWELRPDQD

LSMQVIPTPLPKDGLQVRFWRRKKTT

>CYP197E1(AFZ17691.1)Microcoleus sp. PCC 7113

MKSINLPPSPKGHFLFGILNEYVRDSLGFLTQSAQEYGDIVYFPGIRFVGYKAYFINHPD

YIEEVLATKTHQFGKFNQGLGIIGRILGNGIVTSEGDFWRHQRRLIQPAFHRERIAAYGE

VMVAYTNRMLTRWQAGEIHDVHEDMMRLTLEIAAKTLFDADMADQADEVGQALAFAIAYF

DQWQRNPIAMLLPENVPTPGNLRSRKVIQRLDAIAYELIRQRRETGQDTGDLLSVLLHTQ

YEDGSPVTDQQVRDEVMTILLAGHDTTALAMTWMLYLLSQHPEVEAKLVTEWQTVLNGRD

PTFADLPQLRYTDSVVKEAMRLYPPVWGMARRANTDSEIGGYPIPKGSVIILSQWVMQRD

SRYFNQPEVFNPDRWADGLAQRLPTYAYFPFGGGPRVCIGKSFAQMEAVLLLATMAQKFQ

FTLVPGQKVEPWPAFTLRPKQGIKMVLSERSLQDTPVLSSNL

>CYP110A4(AFZ17821.1)Microcoleus sp. PCC 7113

MLSPLPNRITSPSWWQLLNWIADPLGFQDRYSRKYGDIFTMRLSGLGSYVVVGNPQAIQE

IFSLDSKFDVGRANELAKPLIGQNSVMLMDGNRHRRERKLLMPPFHGERLQTYAQQICLI

TEQVANQWQVGQPFVARTAMQKVSLEVILQIVFGLSEGERYQQLKPLLTDWINMTDSPLR

SSMLFLRFLQQDWGAWTPWGRMKQRQRHIHDLLQAEIEERRTKENEGRTDILSLMMAARD

ENGQAMSDSELRDELLTILFAGHETTATTLAWAFYQIHQHPDVLEKLLHELDSLGENSNP

MEIAKLPYLTAVCQEILRMYPVLPVIFPRITKSPMKIAGYEFDAETTFMPSIYLVHYRED

LYPNAQEFLPERFLERQYSSCEYLPFGGGIRRCLGYPLAQLEMKLVLATILSKYQLTLAE

DKPVKLQRRGFTLAPTGGVKMVMNGKREKKLSGYQLSDISVKS

>CYP110C29(AFZ18689.1)Microcoleus sp. PCC 7113

MIQSQAKLPDGFQSPRWLQKIQWINNSLAYMDVAGQKYGDIFNAPVIGNYRQLLLVSHPQ

ALQQIFSNPHHFIAPSNPLLHPIVGDYSVFVLEGDRHRKERKLLMPPFHGEQMLSNGQLI

CELTEKVMQSLHPGEVFVARDLMQNVSLEVILTVVFGLHKGERFRQLKDCIVSLLSEFKS

PLTSGLLFFPALQKDLGRWSPWGSFLRKQQQINQLLVAEIRDRRQQYDSSRSDILSLLLE

ARDEDGAPMTDEQLRDELMTLLLAGHETTATAIAWALYWVHRFPNVHSQLLQELDSLGNT

PTPIEISRLPYLTAVCNESLRIYPVAILTTPRAVKEPVELMGYQLEPGTKVYGCIYLTHH

RPDLYPEPKQFKPERFLQRQFSPYEFLPFGGGVRRCIGEALALFEMKLVLATIISRYELT

LVQQEPERPKRRGVTFSPHTGVRMIMQGRRSV

>CYP110B8(AFZ18751.1)Microcoleus sp. PCC 7113

MTLPPSPSTPPLVQMLQWIARPMALMEDYTRRYGDCFTLPVGRNFGSVVFVSHPQAMQEI

LSDSKHWEAPGEFNEIFAPILGKHSVITVSGARHQRQRQLLMPPFHGERMRAYSQLIEKV

TEQVMSQWRQDEPFCVRSSTQAIALRVILKAVFGLDEGPRGQQLEHLLAALLDETSSPFS

AGILYFPILQRDLGPLTPWGNFLRRQRETDKLIYEEIQERREHPDPTRTDILSLLMAAQD

EAGESMTDVELHDELMTLLVAGHETTATALAWALYWIHKLPSVRERLLEELDSLGDDPDP

NTIFRLPYLSAVCAETLRIYPVAMLTFPRVVKVPLSLMGYDLKPGTVVIGSIYLTHRRED

LYPEPEQFKPERFLERQFSAYEYLPFGGGSRRCIGLAFAQFEMKVVLAKILSRVQLKLAD

NREVRPTRRGLVTAPDSSIRLVMMGQRQEKMPVSQTVASVG

>CYP110F5(AFZ18752.1)Microcoleus sp. PCC 7113

MKLPDGPQTPSLLQTVQLIAQPTQFLDNCREHYGDTFTTRVLGLNSPPVVFFGNPDAIQE

IFALPSSKLDFRKATHVFEPLMGEQSIILQEGRSHNRLRQLMMPPFHGERMRSYSQLICE

ITQQAIEGWSIGSTVSMQEVMPQITLQIILRVVFGIDPGPRYQDLEQRLSSLLDDVTTPW

YSSLFFFPPLQRDLGAWSPWGHFLRRRQQIDSLIYDEIKERREQADASRTDILSMLISAR

DENGQPMSDVELRDQLVSLLLLGYETTAAVLAWAVYWIYSTASGDEKLRKELEALGDDIQ

PEAIAQLPYLTAVCAETLRVNPIALICTPRRVLESVQVAGYHFDTGTILIPCIYLAHRRP

EVFPDAKQFQPERFLNQKFSPYEYLPFGGGARGCIGMAFSMLEMKLVLATILSRYQLALA

DPRPVRPVRRGITLVPSGGVPVVVKHERTTKPFPAIKVWDYKLTSNH

>CYP107DV1(AFZ19572.1)Microcoleus sp. PCC 7113

MTAIHQTITLEHVDITSPSFRANPFPTFRRWRDTRPVVPVRAFGERAWIITRYDDVLAAL

TDERLVKDRRNAQDPNKKQRGMWTPGFVKPLQRNMIDSDAPDHTRLRSLVHQAFMPRLIS

QMQTRIHRLAHELIDRVEAKGEMDLVDNFALPIPLVVISEMLGVAEPDRAAFHRWSNVMV

NVTKPSNGILAIPSLYQFVRFLRRLFREHRLNPQDDLTSALLQAESEGSQLSEDELIGMV

ALLLSAGHETTVNLIGNGVLALLTHPAELDRLRTEPELMKSAVEELVRYTPPVLFATTRY

AREDIVIAGTCIPKGESVLAALGSANHDESKFENPETLILDRQNNKHLGFGLGMHYCLGA

PLARLESSIAFQVLFERLPNIRLAVNPENLRWNSNLITRGAKAIPVKF

>CYP120B8(AFZ19625.1)Microcoleus sp. PCC 7113

MTTNDALASRPLPPGSFGLPFIGETLSFLSDPEFADKRHQKYGPIFKTQILGRPTVVMVG

PEANRFILSTDMHRFSWREGWPNTFKELLGESLFLQEGEEHRRNRKLLMPAFHGPALANY

LTSMEGIVQNYLEKWESLGTFTWFGELKQMTFDIASTLLMGSEPGPLTALLSQWFTELTA

GLFSVPLRWNWTTYGKALKARDQLLAHIEQAIIKRQKEPAQDALGLLVQSRDEEGNGLSL

EEIKVQALLMLFAGHETTTSMLTLLCMALAQHPDVLARARAEQQALAAEGGLTLEQLKQM

SYLEQVLREVERLYPPIGGGFRGVVQEFEFNGYTIPKGWQALYRINSTHKDSRVYTEPEQ

FDPDRFSPERAEHKKQEFSLVGFGGGPRICLGIAFAQMEMKIFAAHLLRHYTWELLPGQN

LTLDVIPTLHPRSGLQVKFHKIS

>CYP1136A4(AFZ20976.1)Microcoleus sp. PCC 7113

MVQQISEVLVLEDGQTPPSGTTTPPRKPNWYDTFSYIANPDRFCRQNLEQFGPIFNTGVF

GGTTIFLGDAKAIQMAFNGDLKYTEIALPSTTMDMFGEYSLFQRPDLHRQRKSALRPGLT

GQALDGYLPYINDAIAQGIQSWTTPSRMALYPAVEPICFDVLVPLLLGVRLDDSDPTTFE

GLPVSSKAELKALYKSFFDGFYGLSKWKSSLTAYGRGLKARAALIDFMRAVVKKRRIEGK

AIDPTTDFLAMMLVSQQENPDGVFSDTLIENQCLLELWASHYNISGLVCSLMYQLGRHPQ

IVQKLREEQERVVDGQSNVSTFSSAHLKQMEFLEVAIKETLRTLPPSSTANRRLTKSVVL

DGVLYEKGCVLIAEPRLAHIRSEHFQEPEVFAPERFLPERGEGKMYEFIPFGGGVHACLG

AQMAMLVTKVFASHLLRRFDWTLTGEPQFVQFPLKKMKDNYQIDITRRGN

>CYP110D16(AFZ21649.1)Microcoleus sp. PCC 7113

MTLPDGPRSRRLPRLLRTLKLVFRPLDYLDEYSLRYGDIFKIGGEKSPPFVYVGNPEAVK

QIFTAEPAQFESGRGNGVLRYLLGDNSLIMLDGESHERQRRLLMPPFHGDRLRTYSQLIC

DITQEVTDEWTIGKPFFVRPFMQEITLRVILRAVFGLDEGERFQQLRHLLNFMLDALGTP

LSSTLLFFPSLRQDWGRFSPWGRFLRIKQQVRQLLYDEIRERREQGDFSRTDILTLLLSA

RDEAGQPMTDEELHDELMTLLVAGHETTASALTWAFYWIHHLPEVHDKLMADLGTLGDSV

DPMEMARLPYLSAICSETLRIYPIAPSTFIRILKSPMEIGGYQFEAGTALMPSIYLIHQR

EDIYPEPKHFKPERFLERQYSPYEYLPFGGSNRRCIGAALAQLEMKLVLASIVSRFQLAL

TSNRPIKPVRRGLTLAPPAGMQMVAIAPRLEKSPVRA

>CYP197E1(Mic7113_1834)Microcoleus sp. PCC 7113

MKSINLPPSPKGHFLFGILNEYVRDSLGFLTQSAQEYGDIVYFPGIRFVGYKAYFINHPD

YIEEVLATKTHQFGKFNQGLGIIGRILGNGIVTSEGDFWRHQRRLIQPAFHRERIAAYGE

VMVAYTNRMLTRWQAGEIHDVHEDMMRLTLEIAAKTLFDADMADQADEVGQALAFAIAYF

DQWQRNPIAMLLPENVPTPGNLRSRKVIQRLDAIAYELIRQRRETGQDTGDLLSVLLHTQ

YEDGSPVTDQQVRDEVMTILLAGHDTTALAMTWMLYLLSQHPEVEAKLVTEWQTVLNGRD

PTFADLPQLRYTDSVVKEAMRLYPPVWGMARRANTDSEIGGYPIPKGSVIILSQWVMQRD

SRYFNQPEVFNPDRWADGLAQRLPTYAYFPFGGGPRVCIGKSFAQMEAVLLLATMAQKFQ

FTLVPGQKVEPWPAFTLRPKQGIKMVLSERSLQDTPVLSSNL

>CYP110C27(BAG02079.1)Microcystis aeruginosa

MKILPKVKAPTFLQMAQWIINPVAFMENAARKHGDIFSTKVGLTVDNFIFVSSPSALQQI

LTNDRKQFSAPGEANRIIAPIIGDYSVVMLDGDIHKKRRQLLLPPFHGERMRFYGDLIRD

ITLRVMAELPQNQPFKARSATTAIALQVIMEAVFGISQGERYQTLKKTLAEMLDIFNSPV

MASLLFFPILRADFGAWSPWGKYQRYQEKIDDIIYTEIAERKANHNPNRTDILSLLMSAW

DEEGNPMSDKELRDELMTLLFAGHETTATAMSWALYWTHRYPEIQAKILQEIATLGDNPN

PIDITRLPYLSAVCSETLRIHPVGMLTFPRVVEKPVELDGYPLEKGTILMGCIYLAHHRE

QTFPDSHTFKPERFLEKQFTPYEYMPFGGGARRCIGEVLAIYEMKIAIATILANYQLTLV

NNTPEKPSRRGVTLAPSRGVPMVLKGRREPLVTAPMLAEIS

>CYP120A24(BAG04705.1)Microcystis aeruginosa

MTISKDLPLPPGSFGLPLLGETIAFLTDGDFASKRHNKYGQLFRTHIFGSPTIILSGAEA

NRFLLSNENKYFAATWPKSTKTLLGSASLAVHTGDVHASRRRLIYQAFQPRSLASYIPTV

ETITAHYLERWQTATTLSWYPELRNYTLDIACKLFVGLDDGSATKLGEAFDTWCAGLFTL

PIPLPWTAFGKALRCREELLEAIETIILERKKNDDLGQDALAILLQAKDENGQSLSLAEL

KDQVLLLLFAGHETLTSAIATFCLQMALHPDIFQLVLEEITNFDLSTPLSVDTLKQMTYL

DRVLKEVLRFTPPVGGGFRRVIEDCQFNGYHLPKGWVVQYQISNTHKDNNIYSHPETFDP

DRFLAEEKPYGYIPFGAGLRECIGKEFARLEMKILAVRLVQKYDWQLLPNQDLTLTSIPT

PHPRDGLQVTFKPR

>CYP120A24(AKV68544.1)Microcystis panniformis

MTISKDIPLPPGSFGLPLLGETIAFLTDGDFASKRHNKYGQLFRTHIFGSPTIILSGTEA

NRFLLSNENKYFAATWPKSTKTLLGSASLAVHTGDVHASRRRLIYQAFQPRSLASYIPTV

ETITAHYLERWQTAKTLSWYPELRNYTLDIACKLFIGLDQGSATKLGEVFDTWCAGLFTL

PLPLPWTAFGKALRCREELLQAIETIILERQKNDDLGQDALAILLQAKDENGESLSLAEL

KDQVLLLLFAGHETLTSAIATFCLQMALHPDIFQLVLEEITNFDLSTPLSVDTLKQMTYL

DRVLKEVLRFTPPVGGGFRRVIEDCQFNGYHLPKGWVVQYQISNTHKDNNIYSRPESFDP

DRFLAEEKPYGYIPFGAGLRECIGKEFARLEMKILAVRLVEKYDWQLLPNQDITLTTIPT

PHPRDGLQVTFKPR

>CYP110C27(AKV69964.1)Microcystis panniformis

MKIIPKVKAPTFLQMAQWIINPVAFMENAARKHGDIFSTKVGLTVDNFIFVSSPAALQQV

LTNDRKKFSAPGEANRIIAPIIGDYSVVMLDGDIHKKRRQLLLPPFHGERMRFYGDLIRD

ITLRVMAELPQNQPFKARSATTAIALQVIMEAVFGISQGERYQTLKKILAEMLDIFNSPV

MASLLFFPILRADLGAWSPWGKYQRSQEKIDDIIYTEIAERKANPNPNRTDILSLLMSAQ

DEEGNPMSDKELRDELMTLLFAGHETTATAMSWALYWTHRYPEIQAKILQEIATLGDNPN

PIDITRLPYLSAVCSETLRIHPVAMLTFPRAVEEPVELDGYPLEKGTILMGCIYLAHHRE

QTFPDSHTFKPERFLEKQFTPYEYMPFGGGARRCIGEVLAIYEMKIAIATILANYQLTLV

NNTPEKPSRRGVTLAPSRGVPMVLKGRREPLVTARMLAEIS

>CYP110P2(AOW99702.1)Moorea producens

MKFPDGPKTPALLQSINLIVNPLNYLEDCAKRYGGIFTIGFLNYPPTVVVSHPQGMKALF

TASPQTFKTAEAMQELPSTFSGKTALTTIDGDPHRRLKQMLMPAFHGDRMRAYSQLICDI

TQQQMTQWKKGERFDVQGSIQDISLQFIIRAIFGVNEEASFQQLKQTLISQLKLSSNPWA

SIILYFHTLQQDWGPWSLWGRFVRLRQQVDNIIYGEIKRRRDNPALLGEDILSLMMSARD

EQGEPMTDEEMRDNLITLLIGGHETTSISITWALYWIHRLPEVYEKLMVELDTLGDNPDS

SEITKLPYLNAVCHESLRIYPPVILASFRIAQKPIEIMGYQFEPGTFILPCTYLTHRQED

LYPEPNHFRPERFLERQFSAYEFVAFGGGSRRCIGYALSLFEMKLILARILTNVKLALPN

NRVPKPVRSTFTVAPSPIYLIPA

>CYP1136A3(AOX00265.1)Moorea producens

MNQQKGEVTILSDGQATPAGITKPPRQGRWYDTFSYIANPEGFCHHNLEKYGPIFNTGVF

GGTTIFVGSSRAIQMVFNGDSKYTEIALPKTTMDMFGEYSLFQRPELHRERKSALKPGLT

GRILEGYIPRINEVITDGLSNWSDTVQVSLYPAVEKICFQVLVPLLLGVNLDDINPKSFE

GLPLSNCHELKSLYKTYFDGFYGLWKWKSPLTAYGRGLKARAKLLEFMGAVIGLRRASKE

EINPKADFLSMMLASQQQNPDGIFSDSLIENQCLLQLWASHYEISGLVSSLIYQLGRFPQ

VLDQLRSEQATIVGEPTNLNTFLSEQLKQMVFLEAVIKETLRTLPPSSTANRRLTKSVVL

DGMLYAKGCTIIAEPRLAHIMPEHFHQPELFEPERFLPPQNEGRMYEFIPFGGGVHACLG

AQMAMTITKIFASHLIYLFDWQLTGEASFVQFPLKKIKNNYQIILQKRW

>CYP284C1(AOX00584.1)Moorea producens

MFEHSWTILAFLVGTGISLLLWRWQQRQLALSSIYALPSPKGKWLTGNAMELLAAAKQGT

YSLTIFRWMQQYGSMISLRIFTRPMVMVAKPQLIESILTEGQAQGIFTRSPSFYHAYKDV

FGVHIGNQVGEAWKWRRQTAAPAFRASRFTQKFDLIRQGCQQVITQLQSSAQTGKEVQVD

PLFVDLTMNIIAYFFLGVTFDKTSNFAGEPPFDAKRLYAALALLEKHVLLQTAGRSRWFK

FLPTSEGREYRQAQDYLQQNLKPRVAMALQVARASEAESPSVSSSFQDSMLVQFAKNPQH

DQDSLMAETRAFIFAGHDTTAHTMSFAVGELGLNPQVFQAAQQAVDQAWEKEGELNLSTL

KHFDYIEAVVKETLRLHPVATGIPLVTTQETELDGVKMPKNVGVEPFFWAAGQDPEMFPK

PEEFRPERWLQTETDQQPLPLLFGFSRGSHFCVGAPLALLEATVMLSLLLRHFNWELVNG

RDSLEDVNQYLTVFPRDRMPIRFVSRTKSRA

>CYP1320B2(AOX01338.1)Moorea producens

MPQSPIPSIKAARKSKFNLPPGPKGYLLFQLSKLQHQPIEYFGHMWQEYGDLVRLPIMPG

LSLNLASHPDHAEHILSSHQERYGKPDLFLKSMNLLQGQGLFTSEGEVWLRQRRLMQPAF

HQKQLVKLHNVILDCVESLLREWSEKPEGEIIDIAAEMSRLTLKIVSSTLFSIDISSKTD

KLGQSLRTALEYVYYRMNSPLALPVWMPTPRNFEFRKAKQTLDRLVLDIIQYRREHPSEQ

NDLLSMLLTAQDEETGIGMSDRELQDEVITLINAGHETTATALAWTWYLIGTHPDAMAQM

QDEIQTVLNGNYPTVENLFQLEYTRRVFDESSRLCPIGLGLPRVALSDDEIQGYFIPKGT

IFTIAQYFIFRHPDFWDNPEQFNPDRFLPEKVKQRPKFAFFPFGAGQHICIGKNLALMES

TLILAAIMQRFHIELVPNQSIEIDPRFSLRPKYGINVRVRKRY

>CYP110A11(AOX01845.1)Moorea producens

MSKLPDGPQIPKWLQLIYWIADPLKYLEQCVERYGDTFTLRLTALGPLVMVSHPQGIQEV

LTAPPSQFKSGERNQLLRPLLGKTSMTLLDGQPHQRQRKLLIPPFHGQRINSYSQLICDI

TQQVASQWVIGQAFTARSAMQDITIQVIMQGVFGLGHGPRYQKLKPLLTAMLDMTSSPLR

SSMLFFKFLQRDLGAWSPWGKLVRPRQQIYNLLQEEINQRREQPELMGNDILSLMMSTRD

ENGELMSDHELQDQLMTLLFAGHETTATALAWAFYWIHRLPTVRQKLLEEIDSLGDNPDP

MAIAQLPYLTAVAYETLRIYPVAFVIFARITTAPITIMGHHYQANTTLFPCPYLTHRRED

LYPDSDQFKPERFLERQYSAYEYFPFGGGNRRCIGAALAMLEMKLVLAKVLSNYHLALAQ

DKIIKPARRGFAIAPENGVPMVMTGQRVAKDSPKPATVNSI

>CYP284A6(AOX01848.1)Moorea producens

MFQEIAAQIAFSPSLPYLSTALGITSIAGLLGWGWWQRKNTYKSLESLPCPPKHWLLGNV

PQVLAAVKQKKYFQLLFDWSKQLGPIYVLWIDPPILVLSQPKVIENTILNGIKDGSLVRS

KQMRQIWNDLGGAPVLLGENGTEWQWRRKAWNPEFNSSGLSKYFEIINLACEQVIARIQQ

DAPQTEVKVDSVFVELTMRVIFCLVLGIPVEGKSSSSEGPPLDILKAYEAMSIIGYRVLR

LITGEKIWMKYLPTKASGDYWRAMSYLEGLIGPRVDLALKITEKNPTNWGQVSPMFQESM

LVKIAAKEPKYTRKTLIAEAIELLAGGTDTTAHTLSFAVGELALNPRVFQKAQAVVDQCW

QSNGGINTESLKKLTYISAVIKETLRLYSVASGSTSLEAKRDTVIEGNLIPRGTIISWSM

LAAGRDPEVYGNPKEFLPERWLDQGKGSNSLPMITFGSGSHRCLGEHLAMVEATVMLGML

LRYFDWELVNGRSSVEQLQQNLLIYPSDGMPVRFKARDFQCIEET

>CYP284A7(AOX01849.1)Moorea producens

MFQEIAAQITFSPSLPYLATALGVTSIGGLLGWRWWQRKNTYKSLESLPCPPKHWLLGNL

PQVLAAVKQKKFFQLWFDWSKQLGPIYVFWMNPPLLVLGQPKVIENTIVNGMRDGSLVRY

ERIRKVSNEFGGAPVLVGQNGSEWQWRRQAWNPEFSSRGLSKYFNIINLACEQVIARLQQ

DAPQTEVKVDFLFVELTMKVISSLVLGIPVDGKSSSSEGPPLDILKAHEAISILGYRTLR

LITGEKRWMKYLPTKASRDYWRARRYLEGLIGPRVDLALKMRAQNPTNWGQVSPMFQESM

LVKIAAKEQKYTRKTLIAEAILLLIAGTDTTAHTLSFAVGELALNPRVFQKAQAVVDQSW

QSPGGLKGGINTESLKKLSYIGGIIKETLRLYSVASGSTSLEAQRDTVIEGNLIPRGTII

RFSMLAAGRDPEVYANPEEFLPERWLDQGKGSSPLPMINFSSGFHRCLGEHLAMLEATVI

LAMLLRYFDWELVNGRSSLEQLEQNLLMYPSDGMPVRFKARDLQCIEETY

>CYP120A20(AOX02035.1)Moorea producens

METTTQDMQSLPLPPGNVCLPIIGETISFLTDRNFHKKRLDKYGRIYKTHIFGSTTVTMT

TAEANQFLFTNENKYVAAISPKSTQTLLGTASRVNQTGVVHQKRRKLMYQAFQPRALASY

LPTMANITSNYLQKWEEMGTLTWYPELRDYTFDIASTLLMGTDAGSQTPLAQLFKNFSEG

LFTMPISLPWTKFGKALRARQGLLSHIETIVRQRQQQKNSGEQDALGLFLQARDDEGNSL

SLDEIKDHVLLLLWAGYDTLTSAIASFCLLTAQHPDVLAHLRAEQQQFSGSEPLTIEQLK

QMTYLEQVLKEVLRIIPPVSGLFRKVIQSFEFEGYLIPQGWTVLCQITETHNNGEIYQDH

QRFDPDRFSPDRTEDKHKTFGYIPFGGGLWECLGREFAKLEMKIFAAQLLRDYDWTLVPG

QDLEMVVSPTPHPRDGLKVKFSRRVGS

>CYP1719A2(AOX02516.1)Moorea producens

MNTYPSTNVLDLLRLLGNLASGFIRNPGGFDLEKVLGGWIGDVIKRYGSKNVILNFLLKK

VLLVSGRDLSDHILQDSPNSQGYIEGNLKKDGMSFLAPNALTISHDQQWQRLRPYNEGVL

GTGCQHQYQQAFLEQVHRAFSKPVSNIEDIRKCMGQAMLGIVFGENVAPERLIKDIQVLF

SMVGNPIKRILLGRFETKRLEKFYETLEQLWEGSQASEKPCLLSMAHGIKPYTTQEELLQ

QIPHWMFTFTGSGTDLLARTLTMITSRPEVLERVRQEIKEAGSVEQASTIAKLGYLEACL

LETGRLFPPVTRTFHITTAADTFNHNRIPPDMEILHFFPIMQREKSLDPSTDSFVPQRWL

DPRDQDTSTYSNLFLRGSRTCPGRDLILFVCKSAIAILLDQQQLTSKTNLLSRDPLPAYF

QEQDIQFVNP

>CYP110C4(AVZ30628.1)Nodularia spumigena

MSTPNRLKTPAFFQQLQWVADPVGYMEKAAQQYPDIFTAQVVGFGNNLVFVNHPQAMQEI

LTNDRKKLFAGGKENKILQPLLGDYSMIMLDGDRHRKRRQLVMPSFHGDRMRSYGEIISN

ITEEVWSNLPTDKSFLARNVTQDITLQVMIQAVFGVYQGERSQQLKKQLELMANIFRSPL

SSSMLFFSSLQQDLGAWSPWGKFVRDRQELDNLIYTEIAERRQQNLENRIDILSLLMSAE

DESGNPMTVQELRDELMTLLFAGYETTATALAWGLYFIQKHPEVQEKLLQELDTLGDSPD

PMSIFRLPYLTAVCNETLRIHPVAMLTFPRTVKEPVEISGYALDPGTILVGSMYLTHQRE

DLYPEPKQFKPERFLERQFSPYEFIPFGGGVRRCVGEALAVFELKLVLATILSRYELALT

DDQPEVPRRRGVTLAPGRGVNMMITGQRLA

>CYP120A5(AVZ31021.1)Nodularia spumigena

METTNSQTQNKLPLPPGNLGLPLIGETISFLRDPEFAEQRYQQYGPIFKTHLFGQPTIMM

IGAEANRFLFSNENQNFTISWPDSMKTLLGPASLALQTGGTHQKRRKLLSQAFQPRALAG

YTSKMEEITHNYLHKWQRMGTFKWYPELRNYTFDVACKLLIGTDAASDSHFGELFEEWCA

GLFTIPINLPWTKFGRALRCRQQLLVKIEEIILQRQQQPHSDADALGLLLQAEDEDGNRL

SLAELKDQVLLLLFAGHETLTSAIASFCLQLAQHPEVLAQARAEQQQLAIEESLTLEHLK

QMQYLDQVLKEVLRTIPPVGGGFRRVIQSSEFNGYQIPEGWSVLYQIGKTHQDSSTYTEP

ESFDPQRFAPERVEDKQKPFGYVPFGGGVRECLGKEFAKLEMKLFAALLIREYHWELVPG

QNLDLIMVPTPHPRDDLQVNFSTQRGAEGSAKVRGVLDLGGEVAM

>CYP110E7(AVZ31196.1)Nodularia spumigena

MPALQLPDGPKNHPWLQTYRWLTSPLEYMEDCAKNYGDIFTIRVGPLSTPQVFVSNPQAI

QQIFSTDPKYLDSGAAAGFKSPLLGNQSLLSLDGKPHQRQRKLLTPPFHGERMLAYGELI

RDISQQVTNKWQVGETVSVLSSMQAISFQVILKAVFGLAEGPRYEKIKEALIAILNPKKP

LLRSMLLMFPSLRRDLGAWSPWGEFLRLRQQIDELVYAEIQERKAQLDSSRTDILSLMMA

TRDEAGEPMTDLELRDELMTLLVAGHETTATALSWALYWIHHQPQVREKLLQELDTLGEK

PDPNAIFRLPYLNAVCSETLRLYPVAMLLLSRLVKSPLQIGEYQFEPGTLLIPCVYLTHH

REDLYPDSQTFKPERFLERQFSNSEFIPFGGGNRRCIGMAFALFEMKLVLATVLSNWEME

LANTQPVLPVRKGLLFGPKGGVQMVVKGRREQNQLVPETTSV

>CYP227A3(AVZ31230.1)Nodularia spumigena

MCLLKLNPQVKIMAIPEIAKNHQRQILLSRLLAKFGYNTKIGKLLRLIAYYSEAVVMLNA

WKEFLYTRKKHIGDKYFAVDQAIMHSTHSQVQELMQVEPQLRGNDLGIIRILAPSYLLNN

PLSLGMNGNEHTGVRALFLHALPNPSVEVDLLGDLVNQRLLKAAQQGQIHIGNDLPQMML

WILHQVVFQISLSDKEITASNTYIKSLPLASLPNFISKYLLSFRTAPSIKHRQSLIKKYK

QSPRWASYLEIAAQYQLNEHQIANSLFDMIHIAGTAGTSALLGSVIGVLCLDSTLRTEVI

SEVNTVWNGQGTPNASALEQSTLLNKVILETARLYPPVRFVSQLSTESGEVEIGEQRCPF

QKGTRLLGSIFTANRDANRYTNPDDFDVTRDFSDILSWNSKGHERACPGRGLSIGLIKIF

CLYLFQNYQWESSTPVKWDFAKVTAVTPNDLVLQGFAQK

>CYP110E17(ADI66158.1)Nostoc azollae 0708

MQLPPSSEIPRFIQRLQWVFNPLKLLETSAKTYGDFFTLQLTNAEPIVFISNPQGIQEIF

TTPPDYFDAGRSNKLIKPLVGDNSLLLIDGATHQHQRRLLTPPFHGERMKAYAQIITNIT

KQVISDWQLGQPFSVRDSMQEISLRVILQAVFGLCEGQGLTKLQKLVASLLDLSGSPLRS

AVTFFPALQVDLGAWSPWGKFLREREKIHQILYTEIQERRNNPDSSRTDILSLMMSAKDE

NGEGMTDIELCDELMTLLLAGHETTASALTWALYWVHNLPIVREKLINELNSITENTELN

EIYRLPYLTAVCQETLRIYPIAMITLPRIVKTPTKIMGYEFVPGTLLAPCIYLTHRRPDL

YPEPKKFKPERFLERQYSQYEYLPFGGGNRICIGMAFAMFEMKLVLATVLSQLYLELVDN

IPVQPIRRGVTLAPSGGKWLVATGKREKVKIAVGV

>CYP197AC1(AUB35397.1)Nostoc flagelliforme

MSQVKARPESPAGWPVIGNLPDFARDPFAFLRRLHLEFGDVPGFSLVGRKFVLICHPDDT

ERVLFDTGKHFHKGYQDNFAPRLLLGNGLATSEGEFWQKQRRLAQPAFHTQQVSAYANTI

IQITERLLQSWQDGEVRDVHRDMMLFTQQVVAKTLFDADVTDEALEIGQTFDVILQEMTA

EVMSLRQFLPSFVPLPGRARFRAAVNHMEEILQSKIKERRTTSDNHGDLLAMLMEARDDD

GNGMTDKQLRDEIITLYFAGYETTANTLCWMWLLLSYHTETRAKLEAELKRILNGRYPSI

SDVKALTYTEAIIKETLRLYPPGWIIARVASEDVEISGYLVSKGTEVWMSSWLAHRDPRW

FNNPETFIPERWLDDLENRLPKYAYFPFGGGPRVCIGKSLAQLTSILLLSTIAQRYRLEV

LPNQMIEPDPGVTLRPKHGIKVRLHQR

>CYP2029A3(AUB35401.1)Nostoc flagelliforme

MLTKEIPVAKGLPLIGNLVFLSKNNRKFEQFCIENYRKLGPLYKACVLGKEFKIFAGPEA

NMFVTQQGIHHLTTREAFMVMEKEFGENTIMVLDGDSHRRSRKIVDQFLNAAAIAQYVNP

MIQVTLNFIKDWQIGQRIELFHAALPKIILNQLSSALLSCTLTDDFVEDIYICISTLLKV

TSEQKGESALRKPTYLKVKNRMLTFVKQLVDERRKTGQVTDSPDVVDFLLSVKDDDGQLL

TEEEIFSYIPIIIAAGLDTVAHTSAFLIYEILKHPEIYQQVMAEVSAVFDNRIPDLEDMK

NMKVLRGAAMETLRLHPVVFMTQRYVQHPFDYQDYRVESGQIMYFASGVSHFLPELFPNP

YTFDVERYAPPREEHKQPGAFAPFFLGPHRCAGARLAEIQLMLTAATILYTVQPQLDPPN

YTMKTALYTEESPTLAVKGNFYIKTRRKIPLSSLA

>CYP2029A4(AUB35402.1)Nostoc flagelliforme

MLTKEIPVAKGLPLIGSLVFLSADSQKHEQFFLNNYQKLGPLFKVRVLGQELIIFAGPEA

NMFVTQHGTRYLTSREAYLNIAQEFGETSVIVLDGAAHRRSRKVVDQFLNSTAIAQFVKP

MIKTTLEYIKDWQIDQRIELFHMLPKIIFNQFTSVLLSQTSLTDDFYNDVYTCITTVLKV

NFQQKSKSALRQSAYLKAKNRVIVFIQKLVEERRKTEQLRDSSDVVDFLLSVKADDGQPL

TEEEIFSCVLIILSAGLDTVATTSVFLIYEILKHPEIYEQVMAEVSAVFDNRIPDLEDIK

KMKVLRGAAMETLRIHPMVFMTPRYVQHPFDYQDYRVESGQIMYFALGVSHFLPELFPNP

YTFDVGRYTPPRQEHKQPGAFAPFFVGSHMCAGAKLGEVQLMLTAATILYTIQPELDSPN

YTIKKVLYTEKRSALTGKNNFYVRSKRKALI

>CYP1011G1(AUB36053.1)Nostoc flagelliforme

MDKIANLNQKVINSGKTQKSGAARAFKFNPFDSEFRADPYPTYHRIRFEEPVHRNFLGSW

VLTRYADVQAVLRDPRFRSDNLPQRLKNKNPYLKDQHKDLNALAGFSSKWLLYLEPPDHT

RLRSLVGKAFSPSVVERMRPQIQEVVDELIGKVQQRGSMDIISELARPLPMRVISKMLGI

PQEIQSQVYQWANDLSTIFDPLNSLESFAHMNQVILKFSESLSELIAQREKKPQQDLISS

LIAARNEADKLSNNEILSLCMLLFATGEETTVNLIGNGMLALLRHPDQMEKLKREPTIIQ

SAVEELLRYDSPIQGISRIAIENVEIGGKKITAGEQVIVYLGAANRDPAEFSEPDQLELT

RSDNRHLAFADGLHYCLGAALARVEGQIAINSLLQRLPDLKLHIDTLEWRKNISLRGLLA

LPVTFTPD

>CYP120C1(AUB37155.1)Nostoc flagelliforme

MRQLKSAEEMPGSYGLPILGETLEIFRSSELYLWRRFQQYGSVFKTSVMGRKRAYLIGPD

ANRLVLVEQAEHMSSGIGWYFLESTFGNNILLQDGEEHRLTRRLMYPAFHGKAIAKYFDT

IQNIVQDFLKDWGQRTIPLNSSFRQLTLMVATRLFLGSQNKSEVEQTSQWFTQLLESSMA

IFKLNVPFTLYGRGQNARGKLVAFLRQAIAQRIEQGNLEESKDVLGLLLAAVDEDGNKLS

EAQIINEALLLLFAGHETTASLLSWVIFELGNHPEWRERLRQEQLAVVGNNPLNLSHLKQ

LPDLTNVLKEVERLYPPVYAYNRGVLKDIEYAGYRIPAGWFVTISPMLTHRLPELYTDPD

RFDPDRFAPPREEDKKHPLALMGFGYGSHRCLGMEFAQMEMKIVLSTLLRHYDWTVKPDY

SAIAPIRQPSKVKDTLEAYIEPLLIKHPLKSQT

>CYP1912A2(AUB37424.1)Nostoc flagelliforme

MDIGTPLKTSSSQDTETCKIKFNPFATGFKEDPYTTYRLLRQHEPIHRALDMWVVTKYQD

ITHVLASDAVSVNYIPQYIIQQNAGDIPGIVELGEKAIVFTDPPEHGRLRRLASQAFNTI

SIQAHTFYIQQLIETLIGPKINAESINFAQIAHQIPLLTISHLLDIPDEYIGVLDENLHR

VRLVLEPGLLTKLRIRRIEDDLQVCLELFLDIIQYRQSHLGEDLLSKLIQARIGEDKLSH

RELAIACVMTYVAGHETSKGLLGNGVLAFATHPHQWSIFRDGKVSSKQVVDEILRFDPPL

QQTVRLAVSNLQVGQQTIGKGEKMLLCIASANRDEDQFANADDFDITRDASSQIAFGHGM

HNCIGQMLARLEAQFLLSYLVKHVQQIELSSTDYRWIDDGFITRTLSYLPVNLKSA

>CYP110D7(AUB38751.1)Nostoc flagelliforme

MKTRNNKIQKPSIPQTPMTATYNLPDGPQMPRWLRMIKFIGQPIKYVDDFAKIYGETFTI

RSSRSNNHIVYFSQPQALEQIFTADSSHFEVGRGNIGLKFLLGDRSFMLADGDRHQRQRQ

LLTPPFHGERMRAYGQDIEKITRQVSNQWPIGKPFKIRESMQEITLRVILRVVFGLNEGE

LFESLRRSLSDLLDFISSPVMSSAFFFRFMQKDFGAWSPWGRILQQRQKIDQLIYALLRE

RRAESDQNRQDILSLMMAARYDDGQGMSDEELHDELMTLLVAGHETTASALTWAFYWIDH

FPEVREKLLQELNTIGVNPDLSSVAKLPYLTAVCQETLRIYPIAMTAFVRIVKTPIEIMG

YELPEGTAIVPSIYLAHHREEVYPQSKQFKPERFLERQYSPYEYFPFGGGNRRCIGMAFA

QYEMKIVLVTILSELQVSLVNKRPVHPVRRGLTIAAPAGMRMVATLQSKRANTPALV

>CYP110E12(AUB40523.1)Nostoc flagelliforme

MSALKLPDGPQTHPWVQTYQWLTNPLEYMEACAKRYGDIFTLRIGPVFTPQVFISNPQAI

QQIFTTDPKQLDSGEPAGIKSPLLGRQSLLALEGKPHQRQRKLLTPPFHGERMLAYGQLT

REITEQVISQWQVGESFSVLPSMQEISLEVILKAVFGLADGPRYEKLKEVLLEILNPQQP

FITAMMLVFPSLQRDLGSWSPWGKFLRLREQVDELIYAEIRDRKQQADPSRTDILSLMMA

ARDEEGQSMTDVELRDELITLLAAGHETTATSLAWALYWIHHLPEVRAKLLQELDSLGEN

PDPNAILRLPYLSAVCSETLRLYPVVMLALNRLVKSPLEIMGYELNPGTLVIPCIYLTHH

RKDLYPQSKQFKPERFLERQFSPSEYLPFGGGNRRCIGMAFALFEMKVALATVLSHWQME

LADSKPVQPVRKGFLFSPGGGVQMVVTGRRQQNQAVRETSFV

>CYP110AP1(AUB40739.1)Nostoc flagelliforme

MKLPIAAKTPPLLQIIHWIADPIDFYRTYSQRYGDIFIANISPLCSGFETTVFTSNPQAI

QEILTADPELFSSEDNDLINLLAGDKSIFLMNGEYHKEQRKLLMPPFHGKRLTDMGDVIC

KIAEKVSSKWTIGKPFSVRQFMQEMSLQLMMKVVFGISEGQRYEQLEQLLLQKMAVIESP

LNLSLLFFKFLRKDLGDWSPWGNFLRLQRQIAQILDVEIQQRRLQPDIERRDILSMLMSA

RNEQGNAMSNVELHDQLMTLLIAGYESSATALSWALYWIYHQPRVREALLCELNSLAGNL

DPTAISCLPYLSAVCQETLRISPPTMFVPTRLLTKDFNLIGYQLPAGTRLSISIYLAHHR

EDIYPQPEQFQPERFLQKQYSAYQYLPFGGSNRLCIGAALATFEMKLILAVILSRHQLIL

AGKKSIRPVRYGPFVVPSNNFQLVTT

>CYP110D7(COO91_04726)Nostoc flagelliforme

MKTRNNKIQKPSIPQTPMTATYNLPDGPQMPRWLRMIKFIGQPIKYVDDFAKIYGETFTI

RSSRSNNHIVYFSQPQALEQIFTADSSHFEVGRGNIGLKFLLGDRSFMLADGDRHQRQRQ

LLTPPFHGERMRAYGQDIEKITRQVSNQWPIGKPFKIRESMQEITLRVILRVVFGLNEGE

LFESLRRSLSDLLDFISSPVMSSAFFFRFMQKDFGAWSPWGRILQQRQKIDQLIYALLRE

RRAESDQNRQDILSLMMAARYDDGQGMSDEELHDELMTLLVAGHETTASALTWAFYWIDH

FPEVREKLLQELNTIGVNPDLSSVAKLPYLTAVCQETLRIYPIAMTAFVRIVKTPIEIMG

YELPEGTAIVPSIYLAHHREEVYPQSKQFKPERFLERQYSPYEYFPFGGGNRRCIGMAFA

QYEMKIVLVTILSELQVSLVNKRPVHPVRRGLTIAAPAGMRMVATLQSKRANTPALV

>CYP227A1(COO91_10100)Nostoc flagelliforme

MTLKDKVLKGHARQLWLAPILDKVGYDTAIGKFLRLIFYYTDASIILKAWRDFLYIRKDN

IGNKYFAVDQAIMHSSHSQVSELMQTQPQLRGNDLGIIRILAPSYLLDNPLSLGTNGNEH

TGVRTVILQALPEPSQKIDFLGNLVEQSLLEAAKQGKLHIGNDLPKIILSILHQLVFQIF

LSEEEITASRSYIKGLPLASLPNFISKYVLAILTAPKIRHRQHLTKRYKQSAKWASYFET

GAQYQLNEHQIANTLFDMIHIAGTAGTSALLGSVIGVLCLDNALRNDVVSELNAIWNGKK

TLDPDALERSTLLNQVILETARLYPPVRFVSQLTTEGGEVEIGEQKCPFQKGTRLLGSIF

TANRDANRYQNPDDFDLTRNFSDILSWNGEGHERACPGKSLSIGFIKIFCLHLFQNYQWD

SITEVKWDFEKVTAVTPNNLVLQGFAQRL

>CYP1094E1(COO91_10679)Nostoc flagelliforme

MKHLSDYNFFDPEVLVCPYEFYKLAQEEAPIMELPSPKTGAKLFLVTHYDLVIEILKDTR

VFSSNFSTLLAGKEEHDPELQKISDQGWPQMNTLLTADPPEHERFRSLVNKAFTSSRINK

MRDLIEQIVDELIDSFIDRGKCEFVSEFAVPLPLKVIAQQLGVPQADLPNFKQWSDAFIA

RLGQILSREQEIECAKDVVAFQHYFHDVMESRQKQPQDDLITDLIQAKVDGERSLDTAEL

LSIIQQILVAGNETLTSALAAGMLLLTNNKEQIPLVQTDISLLENFVEEVLRMESPTAGM

WRVVTEDTKLRDVDLKAGSLVMLRFDAANRDSIKFIQGERFDVRRHNASNHLSFGHGIHF

CLGAMLARKEMQIAYGRLLIRLKDIRLAQEGYQYLPNVLMRTLKHLYIEFDKAT

>CYP197B1(ACC80102.1)Nostoc punctiforme

MVADVFELPAPSVNSIVGHLFELGQDPLGFLTRCRDYGDIVPLQLGLTPSCLIINPEYIE

EVLKNRNDFIKSRGLRALKSLLGEGLLSAEGESWFWQRRLAQPVFHQKRINGYSQTMVEY

TNRMVQTWHDGETHDIHEDMMRLTLQIVMKCIFSDDIDAGEAKVVADALDVAMQWFESKR

RQNFLVWEWFPRPENIRYRDAIAQMDEAIYKLIQERRNGGEKTNDLLTMLMEAKDEQTLQ

QMDDKLLRDEVATLMLAGHETTANTLSWTWMLLAQNPGVREKLESELNQVLQGKLPTLED

LGQLVYTQQIIKESMRLYPPVPLMGREAAVDTQIGDYEIPQGMAIMISQWVMHRHPKYFE

NPEAFQPERWTQEFEKQLPKGVYIPFGDGPRICIGKGFAQMEAALLLATIAQRFQIDLVP

GYPIVPQPSITLRPENGLKVQLKQIALDTSK

>CYP110D2(ACC81277.1)Nostoc punctiforme

MNIPLSVTLSNMKSRNNKIQKPSNLQTPMTATYNLPDGPQMPRWLRTIKFISQPVKYVDD

FAKTYGDTFTIRSSRSDNHIVYFSQPQALEEIFTADSRHFEVGRGNTGLRFLLGDRSFML

VDGDRHQRQRQLLAPPFHGERMRAYGEDIRKITQQVSHEWKIGKPFNIRESMQEITLRVI

LRVVFGLNEGELFEELRRSLSDLLDFISSPIMSSAFFFRFIQKDFGAWSPWGRILLQRQK

VDLLIYTLLRERRAQTDQNRQDILSLMMAARYDDGQGMSDEELHDELMTLLVAGHETTAS

ALTWAFYWIDHLPEVREKLLQELNTIGVNPDLSSVAKLPYLTAVCQETLRIYPIAMTAFV

RIVKTPITIMGYELREGTAIVPSIYLAHHREEVYPQSKQFKPERFLERQYSPYEYLPFGG

GNRRCIGMAFAQYEMKIVLATVLSEFQVSLVNKRPVHPVRRGLTVATPAGMRMVATPQVK

RANTPALV

>CYP110B2(ACC82131.1)Nostoc punctiforme

MKLPKGPQSPAVLQMLRWITSPMSFMETCAKRYGDMFTIRLDSKSPPLIFVSKPEVLEQI

LTNDIKGLEAPGDTNLVFESLLGKHSVITISGAEHQRQRQLLLPPFHGERMRSYSQIISD

ITEKVISQYQIGQPFNIRSVTQAITLRVIMQAVFGLDEGPRAEKLQHCLAEMLEKGSSVL

SAALLYFPALQRDFGPINFWGKQMRRQQAADKLIYEEIRERQEQPDPSRTDILSLLMAAR

DEAGQPMTDEKLRDELMTLLVAGHETTATALAWAFYWIQKIPTVRQKLLKELDSLGDNPD

PSTIFKLPYLNAVCSETLRIYPVAMLTFARVVRTPLSLGGYELEPGIGVIGSIYLTHHRE

DLYPEPKQFKPERFLERQFSPYEYLPFGGGARRCIGLAFAQLEMKLALAKILSTRELELV

DNSEVRPKRRGLVTGQDRPIQMVVTSQRQVKFPILQTATV

>CYP110C2(ACC82215.1)Nostoc punctiforme

MQLPNILKSPSLLQKLHWVSDPIGYMENAAQEYPDIFTGKIVGFGDTVVFVNHPQAIQEI

LTNDRKKFTAVGELNGILKPLLGDNSVLMLESDRHKRQRQLVTPSFHGERMQAYGQLICN

VSKKIFNQLPLNKPFVARNLTKEISLQVILQSIFGFYEGEKIQKLRQLLPLLLELFESPL

SSSLFLFSFLQQDLGAWSPWGNFLRVREKIDQFLYTEIAECQQQADPERIDILSLLISCR

DEAGQPMTDQELRDQLITLILAGYDTTATAMAWGLYWIHKQPLVCEKLLQELDTLGDSPD

PMSISRLPYLTAVCNETLRIHPVTMFSFPRVVQEPLELLGHSLEPGTILLPSIYLTHHRE

NLYPQSKQFKPERFIERQFSPYEFLPFGGGVRRCMGEALALFEIKLALATIVSHYHLALV

DQRPEQPQRRGFNLAPGSGVKMVMTDQRARKESLINMTTTPLS

>CYP110E2(ACC82780.1)Nostoc punctiforme

MSLLKLPNGPQTHPWIQMYQWLTNPLEYMEACTKRYGDIFTLKLGQNFAHQVFISNPQAI

QQIFTTDPKQLDSGESAGIKAPLLGQQSLLALDGKPHQRQRKLLTPPFHGERMLAYGELI

REITEQVSSQWQVGETFAVLPSMQAISFQVILKAVFGLEDGPRYKKLNELLIKILNPKIP

LLRTVLLIFPSMRQDLGAWSPWGKYLRLRQQIDQLIYAQIQERKAQPNLSGTDILSLMMA

ARDEAGEPMTDLELRDELMTLLVAGHETTATSLSWALYWIHHRPQVREKLLQELDNLGEK

PDPNAIFRLPYLNAVCSETLRLYPVAMSALNRLVKSPLQIGEYNFEPGTILIPSIYLTHH

REDLYPESKQFKPERFLERQFSPYEYLPFGGGNRRCIGMAFALFEMKLVLATVLSRWQME

LADSKPVRPVRKGLLFSPAGGVQMVVKGKRLQNQPILQTSSSSV

>CYP120C1(ACC82922.1)Nostoc punctiforme

MQQLKSAEEIPGSYGLPILGETLEIFRDSELYLWRRFQQYGSVFKTSVLGRKRAYLIGPS

ANRLVLVEQAENMSSRIGWYFLESTFGNNILLQDGEEHRLTRRLMYPAFHGKAIATYFDT

IQNIVQDFLKDWGERGTISLNSSFRQLTLMIATRLFLGSQNKSEVEQTSQWFTQLLDSSM

AIFKWNVPFTLYGRGQNARGKLVAFLREAIAQRIEQGNLEESKDVLGLLLAAVDEDGNKL

SETQVINEALLLLFAGHETTASLLTWVIFELGNHPEWRERLRQEQLAVVGNNPLSLSHLK

QFPQLTNVLKEAERLYPPVYAYNRGVLKDIEYGGYRIPAGWFVTISPMLTHRLPELYTEP

DRFDPDRFAPPREEDKKHPLALMGFGYGSHSCLGMEFAQMEMKIVLSTLLRHYDWTVKPD

YSAIAPVRQPSKVKDILQAYIEPLLIKHPLDS

>CYP120B1(ACC82984.1)Nostoc punctiforme

MKTNQIPPGSFGLPVLGETLSFVFDRDFAKKRYHQYGPIFKTHLLGRPTVVMAGPEALEF

VLSSHIENFSWREGWPDNFKTLLGESLFLQDGEEHRRNRRLMMPALHGPALASYFSTMED

ITRSYLQKWEKKQEFTWFQEFKQLTFDIASQLFLGTRPGPECVRLSQLFTTLTNGLLAIN

PLPLPFTTFGKAIAARNEILEHLTQVVRERQQNPTQDTISLLIKAKDEDGNSLSEKEIIA

QAVLLLFAGHETTTSMLTWLCTELACHPEVLEKARVEQLQLASQGDLDLEQLGKMPYLEQ

VLWEVERLHQPVGGGFRGVIKDFELNGYHVPTGWQLYYSIGVTHQIEEIYSEPELFDPDR

FSPQRQEHKKYPFSLVGFGGGPRICIGIAFAKMEMKIVAAHLLRSYHWEILPNQSLEVVA

VPTNRPKDGLRVRFQPR

>CYP284A2(ACC84600.1)Nostoc punctiforme

MFQQIAAQITFSDSFPYLVTALGITSTAGIFGWRWWKQKNTYKSLQSFPSPKRHWLLGNI

PQVLAAVKEKKFFQLLFDWSQQLGPMYVYWTGFPVLVLSKPKVIEDTIVNGMRDGSLIRS

QRASKAWNDIGGPILLGQNGSEWQYRRKAWNPEFSSSGLSKYVEIINQACEQIIEKIQSV

ASPEVQVDPLFVELTMRVISCLVLGIPVDKNIATNEGQPLDVLKVYEAMSIVGYRFLRVA

TGEKIWMKYLPTKNSRDYWAARRYLEEFITPRVDLALQMREQNQTDLTQVSPLFQESMLV

KIAAKEPKYNRETLVAEVIELLIAGTDTTAHTLSFAIGELALNPRVFHQAQAVVDQVWES

QGTINGESLKELNYIRAILKETLRLYSVASGSTSLEAQRDTVIEGTVIPRGTKIYWSMLA

AGRDPEVYSHPDEFLPERWLEKGKENSQLPMIDFGSGSHRCLGEHLSMLEGTMMLALLVY

YFDWELVNGRSSLEQLQQNLLIYPPDRMPVRFRLRK

>CYP227A1(ACC84809.1)Nostoc punctiforme

MTVNIMTLKDKVLKGQDRQLWLAPILAKVGYDTAIGKFLRLIFYYTDASIILKAWRDFLY

IRKDNIGDKYFAVDQAIMHSSHSQVRELMQTQPQLRGNDLGIIRILAPSYLLDNPLSLGT

NGNEHTGLRTVILQALPEPSQKIDFLGNLVEQSLLEAAKQGKLHIGNDLPKIILSILHQL

VFQISLSEEEITASDSYIKGLALASLPNFINKYLLAILTAPKIRHRQYLTNRYKQSAKWA

SYFETGAQYQLNEHQIANTLFDMIHIAGTAGTSALLGSVIGVLCLDNDLRNDVVSEVNAV

WNGKKTLDPDALEQLTILNQVILETARLYPPVRFVSQLTNEGGEVEIGEQKCPFQKGTRL

LGSIFTANRDANRYQNPNDFDLTRNFSDILSWNGEGHERACPGKSLSIGFIKIFCLHLFQ

NYQWDSITEVKWDFEKVTAVTPNNLVLQGFAQRL

>CYP110F1(Npun_F3743)Nostoc punctiforme

MKILDSLTTPSLLQTLQLIAKPTKTLENYATKYGDIFTMRVMGLKSPPIVFFSHPQAISD

CFAVPAHKLDFKKATHVFKPLFGENSIVFKEARSHQQQRQLLLPAFHGDNLKSYGQAICQ

IAEELTQSWTSGTNICIHKLMSKITLEIILQVVFGITHGVRYQQLKEQLSALLEDVTKPW

YSSLFFFPSLQKDLGAWSPWGIFLKRREQIDKLIYAEISERRWQNDAMRTDILSLLMSAH

DVNGQQMTDEELRDQLVSLLLLGYETTSGVLAWIFYLIHSHPEVKHRLMQELSTLDNLTN

PEAITQLPYLTAVCQETLRIHPIALICTPRMLKEPVEIMGHKFTSETVLVPCIHLAHRRT

DTYPEPEQFRPERFLNQKFSPYEYLPFGGGYRGCIGAAFSMYELKLVTAIILSRFELSLT

DKRPAYPVRRGITIVPSGGVKMVVTKKAKFKRQTILST

>CYP110AT1(AUS99558.1)Nostoc sp. CENA543

MNLPQGPKLPAWWLRMQFTADPLRFLDQMNQRYGDIFTIMSESTPLIFVGNPQGMKQIFT

NTTEIIAAGELNAESAPLVGQNGLLLLDGIRHRSRRKLLMSPLHSNKVKAYGQQICDVTE

KVMNQLPLGKSFLAYPTMQKITLEVILNTLFGLHTGDRYEQLKQLLTNLMNYARSPFVEI

PLSFPFLQQDLGRWSPWGYFRYLWRQFDNLIYAEISDRRQQPNPNANDVLSELIFARNET

GELLTNEEIRDLFPSLLFGGRDASATAITWALYWLHREPQVREKLRQEINTLGESPNPMS

IVELPYLNAVCNEILRIYPTQFVTFPRIVESATNLMGYHLTPGAIIIGCIYLTHQNPELY

PQPQQFQPERFLEKQYSPYEFMPFGGGARRCPGEALATFEMKLVLAKIISRYQLSLTHTQ

REKPQPQGANYPPASKLKMVLCNIQQHQQSPQEAVHF

>CYP110Q4(AUS99559.1)Nostoc sp. CENA543

MKIPEVNLPLWLNIIKIANNPLNHLDAISKKYGNIFIINFGETPIVFVSEPQAIKQIFTN

TKDIKTPGELNAEAALITGNQGILQLDGLRHKHRRKLMMPAFHGVKMQAQGKLICHLTKQ

VIDEYLAKKTFTAYPTIEDITLQVSMKVVLGLQEGERYAKLKYLLPTILKSMRSPIMQTT

SFLPFLQKDLGKWSPWGYLMHLRQEIFELLYAEVQERRQQADTSRSDILSELIFARDETG

ELMTDEEVRDLLLSPLFAAQDASAVAITWLLYWVYRQPQVREKLQQELATLGDSPDPMSI

AQLPYLSAVCNEVLRIYPAQLFTFPRRVESPIELMGYELAPGTLIMGCIYLTHQQPDLYP

NPQQFQPERFLEKQYTPYEFFPFGGGTRSCIGAALAIFEMKLILATILSNYELALVKKQP

EQPKFEGLMCYPASGVKMHISAKTPRPSAETSAPLCV

>CYP1912A2(AUT00714.1)Nostoc sp. CENA543

MITTAQSPVKFNPFAPGFREDPYTTYKQLRQHNPIHNTLDMWVITKYQDIIQVLNSDAVS

VNYIPQYVVQQKVGDIPGLVELGKKAIVFTDPPDHGRLRRLAGQAFSNTNINAHTAHVQQ

LITELVTPKIHSESFDFAQIAHQIPLYTLSHLLGVPQEYIQPIDEYIHRVRSILEPSLLT

KMRIRRIEQDLQSCLAIFQEIIDYRKTHLGGDLLSKLIQARVGEDQLSDSELGIACVMTY

VAGHETSKGLLTNGVLAFIQHPEQWQIFRQGKATAKQVINEILRFDPPLQQTVRLAVSDL

QIGSETIHKGEKMLLCLASANRDEAQFENADTFDITRDAATQIAFGHGIHNCLGQMIARL

EGQLLFTYLCEQVQEMRLATDNYRWHPEGFITRTLTNLPVTFTPADSTI

>CYP110D11(AUT00994.1)Nostoc sp. CENA543

MMVTYNLPDGPKLPYFVRLFKFITHPTEYLEDFARVYGDNFTIWRQNKENQIVYFSHPQA

LEQIFTADGKYFQNGGGGGLLKVLLGDNSLILLDGDRHQRQRQLLTPPFHGERMRAYGQA

IQEITQQVSSEWEIGKPFNIRNSMQEITMRVILRVVFGVDEGEMYQKLRGLLTSVLDLMG

SAVFSTALFFNFMQKDLGAWSPWGRVLRLIQQIDELIYALIAQRRTESGENRQDILSLLI

SARYEDGQPMSDAQLRDELMTMLVAGHETTASALTWAFYWLDQLPDVREKLLQELVTLGV

NPEPSQISHLPYLTAVCQETLRIYPIVLAGFLRIVKSPIEIMGYHLPKGTLVVPSIYLAH

YRESVYPQSKQFQPERFLQRQFSPYEYLPFGGGNRRCIGLAFAQYEMKIVLATVLAQYQV

SLVNKRPVRPVRRGLTLAAPAGMRMVATRQLISVNTSVTV

>CYP110B3(AUT01536.1)Nostoc sp. CENA543

MQLPKIPPTPAVLQILNWVFRPMAYMEECTQRYGDVFALKLDKSIPPLVFVSNPQDLQII

LTQDTKELEAPGDLNGLLEGLLGKRSVVTISGVEHQRQRQLMMPPLHGERMRAYSQIINN

TTTKVISQYPQGQPFNIRHVSQEITLRVIMQAVFGLDEGVRAEKLQQLLGEILENTSSVW

RIALLYLPALQRDFGPIKIWGRQLELQEQADRLMYDEIQERRENPDSSRTDILSLLMDAR

DEAGQPMTDIELRDELMTLLVAGHETTATAIAWAMYWIHKLPQVKEKLIQELDSLGDNPD

PSTIYKLPYLSAVCAETLRIYPVGMLTFARRVKTPITLGGYELPPGTPVIGAIYLTHQRE

DLYPQPKQFKPERFLERQFSPYEYLPFGGGARRCIGLAFAQWEMKLAIAKILTMRELELV

NNREVKPQRRGLVTGPDHPIQMVIKNQRQIKSRSLETSTVG

>CYP110E11(AUT02500.1)Nostoc sp. CENA543

MQLPDSPKMPKFMQLVKWINNPLQLMEASTKAHGDCFTLWLTNKQPMVFLSHPQAIEEIF

TTHLENLDSRASAQILQPLLGNNSLLLLSGETHQRQRKLLTPPFHGDRMRAYGDIITNIT

KDVISNWQIGKAFSVRDSMQEIALRVILQAVFGLNEGERYTQLQTRLCSILELMSSSFRA

SLSFIPALRLDLGGWSPWGNFLRQRQEIDNLLYAEIQERRNHPDSSRNDILSLMMSARDE

HGEPMSDLELRDELMTLLVAGHETTASALTWALYWIHHLPSVREKLVAELDSFHNADFNE

ITRLPYLTAVCQETLRIYPIGMITIPRIVKSPIEIMGHKFEPGTLLLGCIYLVHRRPDLY

PQPAEFRPERFLEKQYSLYEYLPFGGSNRRCLGMAFALFEMKLVLATILSQVDLKLVDNY

PVKPIRRGVTLAPSGGKWMIATGQRQKLEKSAVEV

>CYP227A6(AUT03066.1)Nostoc sp. CENA543

MQLQEKVLKAHDRQVRLASILAKVGNTSPIGRFLRLIVYYLEAAVMLRNWRNFLYIRKDN

IGEKFFAVDQAVIHTSFSEVKKLMQTQPQLRGNDLGIIRILAPSYLLNNPLSLGMNGQEH

QGARALFSEALPDPLEVSELLGELVNQNLADAAKKGELHIGNDLPEMLIRILHRLVFEIT

LTEEEVKASKGFVNDIAIASLPNFVVKYLLAFRTAPTIKHRQHLIERYKQSPRWAALLET

GSKYQLNEHQIGNTLFDMIHIAGTAGTSALLGSVIGVLTLKEDIRNHVISEVNTIWNNQE

TFNKYSLENATLTEKVILETARLYPPVRFVSQLATQAGEITVKETKCPFQKGTRLIGSVF

TANRDTNRYENPDSFDITRDFSDNLSWNSHGHERICPGRSLSIGIIKTFCLHLLQKYQWQ

SSTEAKWDFDNVTAVTPNDLVLQEFTQKP

>CYP110E24(AUT03481.1)Nostoc sp. CENA543

MTTTLEKPTNQIPSQFRVKTFVPKWLQTLRVINNPMYYLNSRRQKYGDMFISEFSGFPSL

LIISNPQAIQELFTADASLFDSGAGNSILQPLIGANSLVLLDGDRHLKQRKLLMPPFHGE

RMRNYAQVICDITEQVTSKWSINQPFVARRSMQDISLQVILRTVFGLQEGERYQQIKQIL

VELLDTFNYPLSAVFLFFKALQKDLGAWSPWGKFIRRRQLLDELIYQEIHERRTQVATQG

NDILSLLLTARDENGEPMTDIELRDELMTMLFAGHETTAIALSWALYWIHYVPEVREKLL

QELNTIDVANTDPTVIAQLPYLNAVCSETLRIYPVAFFSFSRILKTPMKFMGYDLPKGMS

ISPCIYLTHHRPDIYPEPEKFKPERFLERQFSPYEFMPFGGGNRRCLGMAFALFEMKLVL

ANILSNYSLELLDKAPLKPVRRGLVFTPNGGVNLMVKEKK

>CYP110E24(CLI64_25455)Nostoc sp. CENA543

MTTTLEKPTNQIPSQFRVKTFVPKWLQTLRVINNPMYYLNSRRQKYGDMFISEFSGFPSL

LIISNPQAIQELFTADASLFDSGAGNSILQPLIGANSLVLLDGDRHLKQRKLLMPPFHGE

RMRNYAQVICDITEQVTSKWSINQPFVARRSMQDISLQVILRTVFGLQEGERYQQIKQIL

VELLDTFNYPLSAVFLFFKALQKDLGAWSPWGKFIRRRQLLDELIYQEIHERRTQVATQG

NDILSLLLTARDENGEPMTDIELRDELMTMLFAGHETTAIALSWALYWIHYVPEVREKLL

QELNTIDVANTDPTVIAQLPYLNAVCSETLRIYPVAFFSFSRILKTPMKFMGYDLPKGMS

ISPCIYLTHHRPDIYPEPEKFKPERFLERQFSPYEFMPFGGGNRRCLGMAFALFEMKLVL

ANILSNYSLELLDKAPLKPVRRGLVFTPNGGVNLMVKEKK

>CYP120B3(BAT51864.1)Nostoc sp. NIES-3756

MTKLKHNQMPPGSYGLPILGETLAFLFDKNFIEKRYRRYGSIFKTHLIGRPTVVMVGSKA

VEFVLSSHMENFSWREGWPDTFKALLGESLFLQDGEEHRRNRRLMMPALHGPALTNYVST

MEEITRKYLQKWEAKQEFTWFDEFKQLTFDIASQLLLGASPGEDCAILSKLFTTLTNGLI

TINPLPLPFTKYGKAIAARNQILERLTQIVRQRQQNPSKDALSLLIQAKDEDGNSLSEKE

LIAQAVLLLFAGHETTTSMLTWLCLELARHPKVYQRAREEQLQLASQGDLTLEQLGQMPY

LEQILLEVERCHQPVNGGFRGVIKDFEFNGFHVPAGWQLLYSILMTHRLEEIYLEPERFD

PDRFSPQRQEHKKYPFSLIGFGGGPRVCIGIAFAKMEMKIIAAHLLRSYHWEILPNQSLD

ALLVPTNRPQDGLRVKFQPL

>CYP110E9(BAT52153.1)Nostoc sp. NIES-3756

MKLPDSPQIPKFMQLVQWIYQPLQLMEASAKAHGDSFTLWLTNKQPMVFLSNPQAVQQLF

TAPLEQLDAKASAQILQPLLGENSLLLLSGETHQRQRKLLTPPFHGDRMRAYGDIITNIT

KEVISTWKLGQPFSVRDSMQQIALRVILQAVFGLSEGERYNELQKRLCDILDLTASGLRA

SLSFFPALRVDLGHWTPWGNFLRQREEIDQLLYAEIQDRRDHPDSSRTDILTLMMAARDE

NGEPMTDVELRDELMTLLIAGHETTASALTWALYWIHKLPSVREKLVTELDNFGDGDLNE

LTRLPYLTAVCQETLRIYPIAMITIPRIVQTPLEIGGYQFAPGTMLVGCIYLMHRRPDLY

SQPQEFQPERFLEKQYSLYEYLPFGGSNRRCVGMAFALYEMKLVLATVLKNLDLALVDNY

PVKPIRRGVTLAPSGGKWLIATAQHQTTKNPIEV

>CYP110E22(BAT53018.1)Nostoc sp. NIES-3756

MITLQNNSANEAAIKLPDGSSRFKWLQILKVILRPIETLEEIEQRYGDIFTSDLGVFPTQ

IIISNPQAIQELFTADAQLFDSGSGNFIIQPLVGTNSLILLDGDRHLQQRKLLMPSFHGE

RMRAYGQTIRNITEEVTSRWAIGKQFTARPYMQDISLQVILRTVFGLKEGERYQQIKQVL

VEMLDSFNTPISVIFLFFKSLQRDLGPLTPWGRFIRRRQQLDELLYQEIRERRQKSEANG

EDILSLLLSARDENGQPMTDVELRDELMTMLFAGHETTAIALSWALYWIHYVPEVCEKLL

QELNSIDVANADPMAITQLPYLNAVCSETLRITPVAFFTFPRVLKQPMKFMGYNLPKGMM

IAPCVHLTHHRSDIYPEPKRFKPERFLERQFSPYEFIPFGGGNRRCLGMAFALFEMKLVL

ATILSQYSLELLDKVPLKPVRRGIVFAPPGGVHLMVKDKSSFG

>CYP110D8(BAT53186.1)Nostoc sp. NIES-3756

MTVTYNLPEGPRIPRLVRLFKFITQPIQYVEDFAKVYGDNFTIWSSRESHFVYFSHPQAL

EKIFTSNANCFDTGGGGSPLLELLLGKSSLILLEGDRHQRQRQLLTPPFHGERMRAYGHA

IREITGQVTQQWQMGKPFNIRASMQEITMRVILRVVFGVDEGSMFEELRQLLTILLDFMG

SPLMSSTLFFGFTQKDYGAWSPWGRMVRLIEKIDELIYTLIDQRRADVAENRQDILSLLI

SARYEDGQPMSNAELRDELMTMLVAGHETTASALTWAFYWIDSLPEVREKLSQELDTIDE

NLEPSSIAKLPYLTAVCQETLRFYPIVLNAFFRRTNNPVEIMGYKLPKGTLVVPSIYLAH

HREEVYPQSKQFRPERFLERQFSPYEYLPFGGGNRRCIGLAFAQYEMKIVLATILSQFRV

SRLSKRPVRPVRRGLTLAAPGGMRMVASKWGSRE

>CYP1007A6(BAT55293.1)Nostoc sp. NIES-3756

MTTTTDRPRLTVPGPNPLPIFGRIAPLVEYVQDSIALSRRLFKKYGSVVSLVAGGGTNLY

SSEDYCPGTVLAYGPENVREVTSQHEIYHKYPLTGGLYRKRNESPRTETLKHFGVGLFGV

NSTTHRQHRQLLMPAFHRQRIESYCDDIVTITQSVLEQLSIGKSVNLCEVMRMLTLRVAT

KTLFGADIGDEGGKSAKLLQQIGTLLGSPAIAILPFDIPGLPFHRMLSLMAQLDDEMRTL

IRQKQTQGLDSGDVLSMLIQARDAESGLGLTEDELLGHVSVIFAAGHETSANALTWTLFL

LSQHPQIAADLLDELESVLQGAPPTVEKLQQLPLLERVIKESLRVLPPVPWNARVTSQPT

SLGGYELPTGTEVFVSIYHTHHMPEIFPNPEKFDPKRWEQKEPTMYEYNPFSAGSRICIG

APFALMEIKIVLAMLLTQYRLQYIPQQRLDRDALIVMAPKYGMQMLINKQDRQFTKGVGS

VRGNVCEMVELNN

>CYP110C9(BAT56052.1)Nostoc sp. NIES-3756

MELKYQMQLPNLLNKPAFLQRVQWVLNPIGYMENAVQQYPDIFTAQIIGFGNQLVFVNHP

QAIQEILTNDRKKFAALGEYNELLQPLVGNYSLFMLDGDRHRKRRQLVMPSFHGDRMRSY

GDLIRDITKKVWHQLPKNQVFTARNVTQDISLQVILQTVFGVHEGEKSQRLKKRLALMAD

MFRSPYSSSFLFFPFLQKDLGAWSPWGKFIRDRQQLDELIYAEIAERRAENNPNRIDILS

LLMSSKDEAGNSMTDQELRDELMTLLLAGYETTATAMAWGLYLTHQHPQVLEKLLAELDT

LGDTPDPMSIFRLPYLTAVCNETLRFRPVTMLTLPRAVQEPVELLGHSLESGTILCGSMY

LVHQREDIYPQAKQFKPERFLERQYSPYEFMPFGGGVRRCLGEALAVFEMKLVLATILTN

YELALADKKPEVARRRGVTLAPARGVRMMITGKRTPKESRIMATVQN

>CYP109AL1(AFY40770.1)Nostoc sp. PCC 7107

MNSVLHLQASDSQFFLNLDHWLAQMRREAPVFYSTEHQCWMVFRYDDVSRVLSEWKNFSS

QLPQLHEREDFSQSLATTDPPKHQSLRSIVQKVFTTNQVEALAPRITELTYELLGQMKQH

KQVDLVNTFATPLPVIVIAEILGIPVEDREDFKRWSDGIIAGSQTAIKDMADYFRQLLKE

RQQHPSKDLISDLIAAYAEGEKLTAQELVDFCMILLLAGNETTTNLITNTIWSLHEYPDE

NRRLRNDLSLLPTAIEEVLRYHSPITYLQRYTKVETQIGDQIIPPGKLVWAWISSANRDE

LQFENSDRLIIDREPNKHLAFGYGIHFCLGAPLARLEAKIALKALLEEFPNLQVDATQPL

KPISTLLVHGLETLNVLL

>CYP110B4(AFY43331.1)Nostoc sp. PCC 7107

MQLLKTPNTPAVLQLLNWVFRPMPYMEECAKRYGDVFALKLQKNIPPIVFVSHPQDLQQI

LSHDTKELEAPGDLNSLFQGLLGKRSVISLSGEEHQRQRQLMMPPLHGERMRGYSQIIND

ITAKVISQYPVGQPFNIRTVSQDITLRVIMQAVFGMDQGLRAEQLQQRLKELLENGSSVW

RVVSLYFPALQRDFGPIKVWGKQQALQQTSDQLIYEEIQERREHPDSSRTDILSLLMDAR

DAEGQPMTDVELRDELMTLLVAGHETTATAIAWAMYWIHKLPQVKEKLIQELDSLEDEPD

PNTIYKLPYLSAVCSETLRIYPVGMLTFPRRVKTPITVSGYELPPGTPVMSAIYLAHQRE

DIYPQPKQFKPERFLEKQFSPYEYIPFGAGARRCIGLAFAQWEMKLAIAKMLTIRELDLV

DSTEVKPQRRGLVTGPNRPIRMVVKSERQIKSHNLETTTVG

>CYP110Q3(AFY44060.1)Nostoc sp. PCC 7107

MKLPPGPKIPAWLLALQFEANPFTYMDANYQRYGDIVTIMFGSTPMIYVSNPLGIKQIFT

NTKEITASGTLNEDFAFFTGERGILQLDGFVHKNRRKVLMQAFHGTRMQACGRRICELTQ

KIIERQTIGKPFVAYSIAEEITLSIGIEVVLGLQEGKRYEKVKHLFFSMFKSEQSKLFQL

LTKLPLGTLNLGRWSPQGYLLHLRQEIFQFLDTEVQERRSPSDASRTDMLSDLIFATDET

GELLSNAEVRDLLLSPLFAASDASATAITWALYWIHRSPRVRDRLLSEIDSLGENPDPMN

IIALPYLSAVCYEVLRIYPTQLFAFPRLVESPVEVMGYELPSGTILIANIYSTHQREDLY

PQPKKFQPERFLEKQFSPYEFLPFGGGSRVCIGGTFALFTMKLVLATILKNYQLTLVSQR

PERPKYGGLLAYPANGVQMVMQSKRHLQKQSQPLTSGSF

>CYP110D13(AFY45488.1)Nostoc sp. PCC 7107

MTVTNSLPDGPRVPYWLRIIKFIFRPIEYVEDFAKVYGDNFTVWRKGDNHLVYFSHPQAL

EQIFTADASHFATGGGGGILKYLLGDNSLILLDGDRHQRQRQLLTPPFHGERMRAYGQAI

QEITQQVSNEWVIGKPFNIRASMQEITMRVILRVVFGVDEGSKYQQLRQLLSSVLDLISS

SRMSAALFFQFMQRDWGTWSPWGKFLSQLQQIDQLIYSLIQERRAESGQNRQDILSLLIS

AHYDDGKPMSDAELRDELMTMLVAGHETTASALTWALYWIDCLPEVREKLLQELHTLGVH

SEPSNIAKLPYLTAVCQETLRIYPIVMNGFVRVVKSPIEIMGYKLPKGTLIIPTIYLAHH

REAVYPQPNLFKPERFLERQFSQYEYLPFGGGNRRCIGLAFAQYEMKIALATILSQFQVS

LINKRPVRPVRRGLTLAAPAGLQMVATPQVKQANTPVEV

>CYP110C17(AFY45838.1)Nostoc sp. PCC 7107

MPLPNLIQTPSFIQKLQWIVDPVHYMESAAKKYPDIFTANIVGFGCPVVFVNHPKAIQEI

FTNDRKKFAALGEANSIFQPLIGNTSTIMLDGDRHKQRRQLLMPPFHGERMQAYGSLICD

LTEKVFSELPLNKSFSARTVVQEVSLLIILEAVFGLHKGERYYQLRRLLALLMDVFKSPL

TSSFLLLPFLQKNLGDWSPWGKFLRQRQQIDQLLYTEIAERREQADPNRIDILSLLMSAR

DEAGNAMTDQELRDELMTLLLAGHETASTAMAWALYWIHYQPEVRAKLLQELATLGNKPD

PTSIFRLPYLTNVINETLRMYPVLMATLPRVVQEPAELLGHPIEPGTVLVGGIYLTHHRE

NLYAEPDKFQPDRFLNYQFSPYEYMPFGAGVRRCMGEALALFEMKLVLATILSGYQLELE

SQQPEKAQRRSVTLGPANGVKMVIKGRRVRQRSQVPTATAPTH

>CYP197B2(AFY45902.1)Nostoc sp. PCC 7107

MPKDVFELSAPPVNSIVGHLQQLGQDPLGFLSRCRDYGDIVPLQLGLTSACLVTNPEYIE

EVLKNRTDFIKSRGLRSLKTLLGEGLLTAEGESWFWQRRLAQPVFHQKRINVYSQIMVDY

TNQMLQTWGDGETHDIHADMMGLTLEIVMKCIFSAEVDAGEAKVVAHALDVAMNWFESKR

KQNFLVWEWFPRPENINYRQAIAQMDEAIYKLIQARRNSKEKTNDLLTMLMEAKDEQTGQ

QMDDKLLRDEVATLMLAGHETTANTLSWTWMLLSQNPQVREKLQSELDQVLQGKSPTLED

LGKLVYTQQVIKESMRLYPPVSLMGREAAVDTQIGDYEIPQGTSIMISQWVMHRHPKYFE

NSEVFQPERWTEELEKQLPKGVYIPFGDGPRICIGKGFAQMEAALLLATIAQNFQIDLVP

GYPIVPQPSITLRPENGLKVEIKKIKLNAAEDGKKPVSGANIV

>CYP284A1(BAB73318.1)Nostoc sp. PCC 7120

MLQYITAQIDNSSSFPYLVTVLSVTTIAGTFAWRWWKQKKKYKSLQSLPSPPKHWLLGNL

PQVLAAVKQKKLFQLFFDWSQQLGPMYVVWNGSSPVVILSKPKVIEDTIVNGMRDGSLIR

SARLRQAWNDISGPILIGETGNEWQWRRKVWNPEFSSSSLAKYLKIINQACVQVIDTLKE

TALPKEVEVDPLFVELTMRVISSLVLGIPVDRKITTNEGPPLEVLKVYEAMCVVGYRFLR

QATGEKIWMKYLPTKNSQDYWASRRYLEEFLTPRVDLALQMREQSTDFPQVSPLFRESML

VRIAAKEPKYNRQTLIAESVEFLIAGTDTTAHTLSFAVGELSLNPRVFQKAREIVDQAWQ

GQDNINTESFKELAYISAILKETLRLYSIASGSTSLEAQRDTVIEGKVIPSGTRISWSML

AAGRDPEVYANPEEFLPERWLDKSKETSSLPMIDFGSGPHRCLGEHLSMLEGTMMLALLL

RHFDWELVNGRSSLEQLQQNLLIYPSDKMPVRFRLRN

>CYP110A1(BAB73407.1)Nostoc sp. PCC 7120

MLTQLPNPISVPSWWQLINWIADPIGFQKKYSKKYGNIFSMQLAGIGSFVILGEPQALQE

IFTQDSRFDVGRGNTLAEPLIGRTSLMLMDGDRHRRERKLLMPPFHGERLQAYAQQICLI

TNQIASEWQIGQPFVARSAMQKLSLEVIIQIVFGLADGERYQQIKPLFTDWLNMTDSPLR

SSMLFLKSLQKDWGTWTPWGQMKHKQRSIYDLLQAEIEEKRTKENEQRGDVLSLMMAARD

ENGQAMTDEELKDELLTILFAGHETTATTIAWAFYQILKNVNVQEKLQQELDRLGANPNP

MEIAQLPYLTAVSQETLRMYPVLPTLFPRITKSSINIAGYQLEPDTTLMASIYLIHYRED

LYPNPQQFRPERFIERQYSPSEYIPFGGGSRRCLGYALALLEIKLVIATVLSNYQLALAE

DKPVNVQRRGFTLAPDGGVRVIMTGKKSLKFEQSSKIFN

>CYP110B1(BAB75445.1)Nostoc sp. PCC 7120

MHLPKGPQTPVFVQVLRWVFSPMSFLEDCAKRYGDIFSVKLAKDVPAIVFLSNPKDIQQI

LTNDNNQLDSPGDWNDLFEPLLGKRSVITLSGAEHQRQRQLLMPPFHGERMRGYSQVITD

VTEKVISQHQIGQPFQVRSVTQAITLRVIMQAVFGLYEGSRAEKLQHLLSDLLEKSSSPF

SVALLYFPSLRRDFGPIKFWGEQVQIQQQADELIYQEIQERRENPDPSRTDILSLLMDAR

DADGQPMTDVELRDELMTLLVAGHETTATALAWAMYWIHKLPPVKARLLEELDSLGDNPD

STTIFKLPYLNAVYSETLRIYPVAMLTFARRVIETMALGGYELPPGTPVLGSIYLTHHRE

DLYPEPKKFKPERFLERQFSPYEYLPFGGGTRRCLGLAFAQWEMKLALAKILTSYELELV

NNSVEVRPKRRGLVTGPHRPIEMVIKSQRQITSRILETTTVS

>CYP110C1(BAB76385.1)Nostoc sp. PCC 7120

MKYQIQRPNPLKTHPFLQKLQWIADPVEYMKKASLQHPDMFTAEVIGFGDTVVFVSHPQG

IQTLFANDRKKLVAVGEANRILYPLVGNNSMFLLEGVKHKQRRQLLMPSFHGERMREYGH

LIRNITENLFSQLQQDVTFSALTAMREISMQVILQAVFGFYEGERCQQFKHLLPIFLSEL

FQSPLASSILFFPSLQKDLGNLTPWGRFVRQREKIDKLLYAEIAERRQEINSDRIDILSL

LISARDETGDSMSDKELRDELITLMISGHETTGTAMAWSLYWILQTPEVFQRLIQELDSL

GDSPDPMSIFRLPYLTAVCNETLRINPVAMLTLPRVVKEPIELLGNRLETSTTVVGCIYL

THHREDLYPESKLFKPERFLKREFSQYEFMPFGGGVRGCIGQALAMFEMKIVLATVLSRY

QLALADRKPERPQRQGFTLTPTNGVKMLITGQHKRQNYSMAASTTFNA

>CYP110D1(BAB76465.1)Nostoc sp. PCC 7120

MTVTQNLPNGPRIPRLLRLFKFITQPIQYVEDFAKVYGDNFTIWGSGESYFVYFSHPQAL

EQIFTNVSCFESSGGGSPLLELLLGKNSLILLEGDRHQRQRQLLTPPFHGERMRAYGQTI

REITQQVTQAWQMGKPFNIRASMQEITMRVILRVVFGVDEGELFQELRQLLTTLLDFMGS

PLMSSTFFFSFTQKDYGAWSPWGRMVRLIKKIDQLIYALIAQRRAEFGENRQDILSLLIS

ARYDDGQPMSDVELRDELMTMLVAGHETTASALTWAFYWIDSVPEVREKLFQELDTLNDD

SEPSIIAKLPYLTAVCQETLRFYPIVLNAFFRRTKNPMEIMGYKLPKATLVVPSIYLAHH

REEVYPQSKQFRPERFLEKQFSPYEYLPFGGGNRRCIGLAFAQYEMKIVLATILSQFQVS

RLSKRPVQPVRRGLTLAAPGGMKMVANKRMRNS

>CYP110E1(BAB76532.1)Nostoc sp. PCC 7120

MKLPDSPKIPKFMQLVQWIYQPLQLMEASAKAHGDSFTLWLTNKRPIVFLSNPQAIQELF

TTPLEQLDARGTAQVLQPLLGENSLLLLSGETHQRQRKLLTPPFHGDRMRAYGDIITNIT

KEVISNWQLGKPFSVRDSMQEITLRVILQAVFGLREGERYTQLQKRLCDILDLSGSALRS

TLSFLPALQIDLGRWSPWGHFLRQREAIDQLLYAEIQDRRDHPDPSRTDILSLMMAARDE

NGEAMTDVELRDELMTLLVAGHETTASALTWALYWIHKLPQVREKLLAELDNFGDNGDVN

EITRLPYLTAVCQETLRIYPIAMVTIPRITKTNLEIGGHQFAPGTMLVGCIYLMHRRPDL

YPQPQEFKPERFLEKQYSLYEYLPFGGSNRRCVGMAFALYEMKLILATVLANVDLALVDN

YPVKPTRRGVTLAPSGGKWLIATAQHQKIKNPVEV

>CYP1177A1(AFY47549.1)Nostoc sp. PCC 7524

MFIFLEINLIQPDKLAESLGLVAVLSIVALLMRSHYFPKGSNPNLPPGSYGLPLIGETIP

LALDIHSFYRERVQKYGPIFKTHLFGKPVIIFNGPEAFTFFLNQEYFTRANASPKPIQEL

LAWDALPLLDGDEHRRRKRIILQAFTPQAFDQYIPLIEQTANYYLPRWEKLGSFVWLTEY

RKFSASLTNALFIGTAPGASSEAVGEITDTFIKGFSAIPINLGFNAYGKALKNRDWLLNY

IDQAIEQHRQQPRKDLLGLVLTSRGEDGSTLTDDQLRREVMHLFFAAYSGFYVALTLLSL

TLAKYPEMTARARKEVNQYVPDGALDMAKLQKLVYLEQITKEIRRFYPINAATFFGQVKQ

DCEFHNFRIPKGWGAVAGIHTTMQMPQVFAEPQTFKPCRFAPENFATLPENSYVPQGGGP

RDGHRCAGEDLVTVLLQVMGVYLLRRYTWELPPQNLEFNQDLFPTPKDGLKVKFGIHQQA

V

>CYP227A2(AFY47820.1)Nostoc sp. PCC 7524

MELKDKVLKNHDRQIKLASRLAKVGYDTTIGKLLRLPIYYMDAFIMLNAWKDFLYIRKKH

IGDKFFAVDQAIIHTSHSQVKELMQVEPQVRGNDLGIIRILAPSYLLNNPLSLGMNGNEH

TGVRAVFLQALPDPTQKADVLGGLVNQCLSKAAQQGQVQIGEDLPRMLLEILHQLVFQMA

LSEAEITASNAYIKSLAIASLPNFITKYLLAFRTAPSIKHRQRLIERYQQSPQWAAYLKT

GSQYQLNTHQIVNSLFDMIHIAGTAGTSALLGSVIGVLCQNEALRNDVITEINAVWDGKQ

PPTGQTLEQLTLTHKVILETARLYPPVRFVSQLATQSGEVEIGEQKCPFQKGTRLLGSIF

TANRDERRYQNPDNFDVTRDFSDILSWNSQGHERMCPGRSLSIGIIKTFCLYLLKNYQWN

SITEVKWDFEKVTAVTPNNLVLQGFAQKI

>CYP110E10(AFY48695.1)Nostoc sp. PCC 7524

MKLPDSPKMPKFMQLVQWISNPLQLMEASAKAHGECFTLWLTNQQPMVFLSNPQAIQEIF

TKPLEQIDARGSAQILQPLLGDNSLLLLSGETHQRQRRLLTPPFHGDASRQDLAGQRMKA

YGDIITNITKDVISNWQLGKPFSVRDSMQEITLRVILQAVFGLHEGERYTQLQKRLCSLL

ELTSSSLRASLSFIPALQVDLGRWSPWGNFLRQREEIDKLLYAEIQERRDNPDPSRSDIL

SLMMSARDENGEPMTDVELRDELMTLLVAGHETTASALAWAFYWIHHLPSVREKLLAELD

SFPDADCSEITRLPYLTAVCQETLRIYPIGMITIPRIVKSPIEIMGHNFEPGTMLLGCIY

LVHRRPDLYPQPEQFKPERFLEKQYSLYEYLPFGGGNRRCLGMSFALLEMKLVLATVLSH

LDLALVANYPVKPIRRGVTLAPSGGKWLIATGQRQKMKSPVEV

>CYP1007A5(AFY48758.1)Nostoc sp. PCC 7524

MAITTDRPKLTVPGPNPLPIFGRTALLVEYVKDSIALSRRLFKKYGSVVSLAAGGGTNLY

SSEDYCPGTVLAYGPENVREVTSQHEIYHKYPLTGGLYRKRNDSPRTETLKHFGVGLFGV

NSTTHRQHRQLLMPAFHKQRIESYRDDIVGITQSVLEQLPLGKPVNLCEIMRLLTLRAAT

KTLFGADIGDEGGSSARLLQKIGTLLGSPAIAILPFDVPGLPFHRLLNLMAQLDDEMRTL

IRCKQASGTDSGDVLSMLIQARDADSGLGLTEDELLGHVSVIFAAGHETSANALTWTLFL

LSQHPQVAADLLDELESVLQGAPPTIEQLQQLPLLERVIKESLRILPPVPWNARVTSQPT

SLGGYELPTGTEVFVSIYHTHHLPQIFPNPEKFDPQRWETKEPTMYEYNPFSAGSRICIG

APFALMEIKIVLAMLLMQYRLQYISRQQLERDALIVMAPKNGMQMLIHKQDRQFTQGVGG

VRGNVCEMVRLNN

>CYP110D10(AFY49187.1)Nostoc sp. PCC 7524

MTVTHNLPDGPRKHYLLRVFKFITQPVEYLEDFAKIYGDNFTVWRKNRENQIVYFSHPQA

LEQIFTSDAKCFETGGGNGILRFLLGDHSVILLDGDRHQRQRQLLTPPFHGERMRAYGQA

IQEITQQVTDEWEIGKPFNIRASMQEITLRVILRVVFGVDEGEMFQKLRELLTSVLDFIS

SPLMSTAFFFNFMQTDLGAWSPWGRVVRLIQQIDQLIYALIAERRAESGENRQDILSLLI

SARYDDGQPMSDVELRDELMTMLVAGHETTASALTWAFYWLDQLPEVRDKLLQELATLGV

NPEPSNIARLPYLTAVCQETLRIYPIVIGGFLRTATAPIEIMGYQLPKGTFVVPSIYLAH

HREAVYPQPQQFKPERFLERQFSPYEYLPFGGGNRRCIGLAFAQYEMKIALATILLQYQL

SLVNKRPVRPVRRGLTLAAPAGMQMIPTPQHNVVRT

>CYP110E25(AFY49268.1)Nostoc sp. PCC 7524

MITAQENSTNQILPKLPIKPPIPRWLQTLRVLINPIYYLQKTQQEYGDIFISEFGGFPAQ

IIISNPQAIQELFTADAQLFYSGEGNNTIEPLVGTNSLILLDGDRHLQQRKLLMPSFHGE

RMRAYGELIRDITKQVASQWNIKKPFVARPTMQDISLQVILRAVFGLKEGERYQQIKQVL

TEMLDSFNYPLSAVFLFFQFLQHDLGDWTPWGRFVRRRKLLDKLLYQEINERRTQVENQG

EDILSLLLSARDENGQPMTDVELRDELMTMLFAGHETTAIALSWALYWVHYIPEVRDKLL

QELNSIDIANADPTTITQLPYLNAVCSETLRIYPVAFFSFTRILKVPMKFMGYDLSPEMR

ISPCIYLTHHRPDIYPEPERFKPERFLERQFSPYEFFPFGGGNRRCLGMAFALFEMKLVL

ATILSNYSLELLDKVPLKPVRRGIVFAPNGGVNLMVKEKK

>CYP120B2(AFY50183.1)Nostoc sp. PCC 7524

MKSNQIPPGKFGLPILGESLEFIFDPKFIEKRYHHYGEIFKTHILGRPTVVMVGPKAVEF

VLSSHMENFSWREGWPENFKILLGESLFLQDGEEHRKNRRLMMPALHGPALANYVSTMAD

ITQTYLQKWENKQEFTWFEEFKQLTFDIASQLLLGTNPGEECIRLSQLFTTLTNGLFAIN

PLPLPFTKFNKAVAARNQILEHLTQVVRERQQQPTKDALSLLVQAQDEDGNSLSEKELIA

QAVLLLFAGHETTTSMLTWLCVELARHPEVMQRARDEQLQFASQGELSLEQLGKMPYLEQ

ILLEIERLHQPVGGGFRGVIKDFEFNDFHVPAGWQLLYSILTTHRLEEIYPEPERFDPER

FSPQRQENKKYPFSLIGFGGGPRICIGIAFAKMEMKIVAAHLLRKYHWEILPNQSLDTVR

IPTNRPKDGLRVRFQPL

>CYP1536A2(ARV57260.1)Nostocales cyanobacterium HT-58-2

MNTILQQQKNHHRQALRPPLAKGLPVLGNALDLAGDVGDFLRKKYLELGPVFRVRALNRE

MVVLAGREANLFFSKEGTKYFASRAVWQDFNAQLGISRSLINMDGPDHTKYRNQLKPGYS

VAMLEQNLDQAVAIVRQEVATWSTIKPQPVFNLMQRIIIQQLGLLITNYSPHEYLDDLIL

FSRILTATTIGKQRPKFLTYLPAYQRAKARVFELARKVIATHSTELRGDRHPDLIDHLLQ

FVKQEPEFLSKQEDRINAAMTPFLAGVDTAASVTAFLLYSLLKHPDILEQATVEANALFA

NDTLTPDNLRKLDVLQRVTLETLRMYPIAPVLFRSVTVPFEFAGYRIEAGQRVLIAISLP

HHLPEFYPNPFQFDIERYSKGREEHRQPGVFAPFGLGSHLCLGMNFAQALIPLNIATILH

AVRLSMVPANYQLQIDPIPTPSPNKKFQIRVAEQLE

>CYP110C10(ARV57511.1)Nostocales cyanobacterium HT-58-2

MQLPNLVKTPSFIQKIQWVTDPVGYMESAVQQYPDIFTAKVVGFGDTVVFIQHPQAIQKI

LINDRKKFAALGEENSILLPLVGNNSILLLERERHRRQRQLLMPPFHGERMRAYGELIRH

LTEKVWCQLPLQKPFSARTIMQEISLQVILKTVFGLYEGERYQKLKYLLSLMTDVFQSPL

ASGLLFFPFLQKDLGAWSPWGRFLRQCQQIDELLYMEIAERRAQADPNRVDILSLLMYAL

DEEGQHMTDQELRDELITLLLAGHETTASAMAWGLYWLHRLPEVREKLLEELDTVGDLAD

PMSVARLPYLTAVCHETLRIHPIAMLTFPRVVQEPVEILGHQLEPDTIIVGCIYLTHQRE

DLYPQPKQFKPERFLEHQFSAYEFIPFGGGVRRCIGEALALFEMKLVLATILSRYELTLA

SHQPEQPQRRGVTLAPSGRVKMIITGERERIESPKAVASV

>CYP1184A3(ARV57857.1)Nostocales cyanobacterium HT-58-2

MSGKGSTQSQKNTCPHLGEAYQPFVNPQLDDPYSFYERARSEEPLFYSPLLDAYVLTRYD

DIQTVLKDPTRFSSADALRPIVHFPPEVLEILSQGYPDVPTMLNSDGENHKRLRLPFTKA

FAPEQLEALEDSVRAIANRLVDNFVNDGHADIISQFACPLPLEVILIMYGIPLEMGTDIK

KWCDDRTALRSSQLTKERQLECARSHVAMQQAIAALIEERRIKPRNDLISDIVTSDLGMS

ELVILLIGIILAGHQTTTHLIGNALKLLLEQQQFWQALCKDPSLIPFVLEEVLRYDTSVP

SMIRTTTQEVSLAGVTLPKGSRIFLMYSSANRDEAQYPDADRFDIERFQHVSVNHLAFGY

GIHHCLGANLARREGRIALEVLSKRLPNLRLRPNQQLDHIPTMMVRGFTQLYVEWDVT

>CYP110D14(ARV58185.1)Nostocales cyanobacterium HT-58-2

MRVTNNLPNGPKMPVFLRRLKFIFQPVEYVEGFAKKYGDTFTLWGRNDSPLVYFSHPQAL

QQIFTADSGLLDAGRGNQDLRFLLGSNSLLLLDGDRHQSQRQLLTPPFHGDVSRQDPKGL

RMRAYGETIREITQQVGDEWKMGQSFNIRASMQEITLRVILRVVFGLDEGPRLEKIRQLL

SALMDSTTSSFMSVGLFFGFMQKDLGAWSPWGRVLRLRQQVDEIIYELIQERRTESGQNR

QDILSLLMSARYDDGQPMSDEELRDELMTLMVAGHETTASALTWAFYWIDFLPQVRDKLL

KELDTLGDKPDLNTIAKLPYLTAICQETLRIYPIAMNAFPRIVRSPIEIQGYTLPQGTVI

LPSIYLAHHREETYPQSKQFRPERFLERQFSPYEYLPFGGGNRRCIGLAFAQYEMKLVLA

TILSRFQVSLVNKRPVRPVRRGLTLAPPAGMQMVATPMAKRATTVGKQFAT

>CYP110E23(ARV58547.1)Nostocales cyanobacterium HT-58-2

MIELQEISPEKIGNKLPNIAREHKWLQTVWGITRPLDYLEKMQERYGDIFIADFASSPPQ

VIISNPKAIQELFTADYKLFQSSPGNQIIEPLVGPNSLLLKDGEPHLQQRKLLMPPFHGE

RMQAYGQLMRDITQQVMSKLTVGSSFVARPIMQEISLTVILRAVFGLKEGERYQRIRQLL

TAMLDSFQSPLSVVFLFFKSLQRDLGPLSPWGNFLRQRGRLDQLLYQEIQERRTQEQFSG

DDILTLLLSARDEQGQPMTDVELRDELMTLLFAGHETTATALAWALYWIHSLSDVREKLL

QELHSIDIANAQLTEIARLPYLNAVCCETLRIYPVAFFTFARTLKTPMQLMGYELPEGLR

LSPCIYLTHHRPDIYPEPKQFKPERFLERQFSPYEYLPFGGGNRRCIGMAFAMFEMKLVL

ATILSHYSLALAETRPLQPVRRGVTFAPAGGVRLVVKERH

>CYP110E20(ARV58969.1)Nostocales cyanobacterium HT-58-2

MVQKKQPEGPQTPALLQILQWSTRPLEFMETCAKRYGDCFTVKLGGSTPYVFFSNPQAIE

KIFTTAPNLFGVYSILRPTVGDNSLIILEGDRHHQQRQLLMPPFHGERMRAYGQLIFQIA

EKVTSQWTKGAEVTIRPFMQEISLDIILQAVFGLNQGERSQQLKPLLISLLNLTTSPLMF

ALAFFPALIKDLGPLSPGRYFVQRKQKIDQLIYAEIHERREKLDPSRSDILTLLMSARDE

AGQPMTDQELRDELITLLLAGHETTALGLTWALYWIHSLPTVKEKLLKELDTLGSDLDPN

TITRLPYLNAVCSETLRIYPILLQTVPRVVKSPMHFMGYEFEPGTFLSPCIYLTHRRQDL

YPEPEQFKPERFLERQFSPYEYLPFGGGNRRCIGAAFSLYEIKLVLATILSRFELALAES

GPVRPVRRGVAMAPASGVKMVFKGERQN

>CYP110AQ1(ARV62959.1)Nostocales cyanobacterium HT-58-2

MKLPNRLRTPWFAQVYQWGMRPTHFLDSCTQRYGDIFTTQWPALGPIVFVSHPKAIEKIY

TAPPEMFNIAETYETLRPLVGDKSLMVMDGQVHHRQRSLVMPPLHGERLRSYGKLICNLT

EQMMQQWKLGEVINLTSWAHDISINVMFQVVFGLDEEERLIKLRQKITQMLDYFSSPVMV

LHHTAPVLRKDFGPLSPWGRFLCLIHEVDQLLYAEIAHRRQHPQLDCTDVLSLLMTARDE

QGEVMNDQELRDEVMTVLSGKGVAATGILFGLYSLQKHPAVCQRLQEELDSISDPSDTNA

IAKLPYLTAISQETMRLYPLTAVAMRVATTPFEIMGYEFPSGTKVFVNIYSVHHRHDLFP

NPEQFKPERFLERKFTSYEYSPFGGGNRRCIGYAFAPFFMKLVLAKIVSRYQLKLTDDRP

ISLVRHAAGVAPDRDIYAMVTDYYHQKSANELVGVV

>CYP110AQ2(ARV58991.1)Nostocales cyanobacterium HT-58-2

MKLPPCPRTPSFVQVYQWATRPTHFLDSCAQRFGDIFMARWPGYGLTVFVNHPEAIKQIF

TAPPETFSTAQIYEILRPVVGDQSLMLMDGKPHRRQRSLVMPALHGERMRLYGQLICKLT

EQMMEQQELGEEINLSSCMQTLSLNIVLQIVFGLDETDQLIQLRQKFMEMFNWIRSPLLI

LHLVVKSLRINWGPWSAWGRYLRLRNEVDKLLYAEIAQRRKYPQPERTDILSLLMAAHDE

EGQPMSDKELHDEVMTVLTGYESVATATLCSLYCLQKHPEVLQKLRQELDSISDPSDTNA

IAKLPYLTAISQETMRLYPPVVVAMRVAKTPFEVMNYRFPVGSQIVADIYSVHQRQDLFP

NPKQFKPERFLERQFSQYEYFPFGGGDRRCIGYAFAPFQMKLVLATIVSRYKLQLTDDRP

IRLVRNAAGVAPDKDIKMMLTGYRHQKPKSTSISTPINVR

>CYP110AR1(ARV62985.1)Nostocales cyanobacterium HT-58-2

MKLTSASHKLSFPKLLQWTNRPADFLDTCTSRYGDIFTVQFPGGKPVTFVSHPKALEDVL

TAPLGTFTSGEGNEMFSPLMGNSSIMLLDGKPHQRLRKLIMPPFHGERMRFYGQHICELT

EQMMQSWTIGQSFDLRACTENVPVNVLPKVVFGLTEGERSKQLHEKLLALVNLSTSPLIA

LHLFFPPLRKDLGAWSPWGRLLRLRDEVDQLIYAEIAQRRQQPESERTDILSLLMLTHDE

DGEPVSDQELRDQLISLLMAGFDTTSTALTWALYWIHKLPRVREQLLEELDTINDFSDTR

AIAQLPYLTATCQETLRIYPSIMLPFLRVAQTPFQMMGDEFSAGSRIWPNIYSAHHRREV

YPDPEQFKPERFLERQFSPYEYLPFGGGNRLCVGSAFALFLIKLVLFTILSRYQLTLVDP

RPIHPIRHGPGLAPSRALQMMVTGHHHRRSNVAVLT

>CYP110F2(ARV59285.1)Nostocales cyanobacterium HT-58-2

MMHSLLQTFRLIANPTQFLEDCANQYGDQFTVRVLGINSPPVVFLSHPQAIADCFAVPAK

ELDFKKATHVFEPLFGSSSIVLQEGRSHNRQRQLLMPPFHGDCMKTYGQAICQITAEVTQ

NWTQGTTISMQQVMPDITLQIILQVVFGLSPGARYQKLKELLSSLLEDVTKPLYSTLFFF

PPLQKDLGAWSPWGNFQRRREEIDKLIYAEIDERRLSHDASRTNILSLLMSARDENGEQM

TDKELRDQLVSLLLLGYETTAGVLAWVFYLIHSHSEVKDKLMQELHTLSDTPNSETITQL

PYLSAVCQETLRVHPIALICTPRMVKDTVEIASQKFTSGTVLVPCIYLAHRRAETYPEPE

KFKPERFLNQKFSPYEYLPFGGGYRGCIGAAFSMYEMKLVIATILSQFQLELADNHPVYP

VRRGITIVPSGGVRMFVTKQMNQAKNFVYL

>CYP110B5(ARV59286.1)Nostocales cyanobacterium HT-58-2

MKLPNGPQTPALVQMLQWIFSPMSFMENCAKRYGDFVTLRFDPQYSPFIFVSDPQALQQI

LTSDTKQFVAPGSINKIFKPLVGNYSVLTLDAEAHQRQRQLLLPPFHGERMRTYGQAISD

VTQQIISQLQIGKPFSARTVTQAVTLRVILQAVFGLYEGPRAQQLEQLLRNMLEHSISLL

FVVLLYFPVLQRDLGPLTPWRTFVRIREQVDQLLYAEIRERREHPDPSRTDILSLLMAAR

DEEGQPMTDEELRDQLITLLIAGHETTATALVWALYWVHKLPSVREKLLEELDSLGDNPD

PSTIFKLPYLNAVCCETLRIYPVAMLSMPRVVKTPVSLCGHELEPGTFLFGSIYLIHQRE

DLYPEPKKFKPERFLERQFSPYEFIPFGSGARRCIGLAFAQFEMKVALATILSRWELALV

DNGEVRPKRRGLATAPDRPIQLVVKGKRTVKSQTLASISG

>CYP1007A4(ARV61260.1)Nostocales cyanobacterium HT-58-2

MTTITTQPRWTIPGPRPFPILGRPAMVWRFATDSIGLSRDLFQTYGPVVSLTAGGGTNFY

SPLPNCPGTVLTYGSEYVRQVTSQHEIYYKYPLSGRLYSKRDDSKRTEPLKHFGVGLFGV

NGLTHRQHRQLLFPAFHKQRIDSYRDDIVAMTQSVLEQLPIDQPIDIVEVMRLLTLRVAT

KTLFGCDVGEEGGRTTKILQEAFALEGSVGVALLPFDLPGLPYHHLLNLIAQLDDEMRVL

IQGKQVSGTDDGDVLSMLIQARDAESGVALSEDELLGHVGAIFAAGHETSANALTWTLFL

LSQHPEITADLLDELQSILHGGPPTVEQLQQLPLLERVIKESMRVLAPVPWNGRVTSQPT

ELGGYTLPAGTEVFVNIYQTHHMPEVYPEPERFHPKRWEGFEPTMFEYNPFSAGSRICIG

AGFAMLTIKIVVAMLLQKYRLQYLSQQRVERSGFIVMVPKHGMRMIVRKQDRQFTQGVGG

VRGNVREMVQLP

>CYP120A9(BZZ01_31750)Nostocales cyanobacterium HT-58-2

MTTASNEQLKTQPPEGRSLPLPPGKFGLPFIGETISFLGDPDFAKKRQQRYGPIFKTHLF

GRPTVVMIGAEANRFLFSNENQYFSMSWPDSTKILLGASSLTVQTGDEHKNRRKLLSQAF

QPRALTGYLSTMQNITQSYLHKWERMGTLTWYPELKKYTFDVACKFLVGTDGASDSRFGE

LFEDYSNGLFSIPLRLPWTRFGRALRCREQLLEKIEEIVRQRQQQPNSNEDALGIMMQAQ

DEDGNSLSLQELKEQVLLLLFAGHETLTSAIASFCLLLAQHPEVLATARAEQQQLGLEEP

LTSEHLKQMTYLEQVLKEVLRMVPPAGGGFRETIKPCEFNGYYIPQGWAALYQILKTHED

SSVYTQPERFDPQRFAPERAEDKSKPFSHIPFGGGVRECIGKEFAKLEMKLFAALLLREY

EWELLPEQNLDIVPTPLAHPRDGLQVKLRRLVG

>CYP110C21(AFY81354.1)Oscillatoria acuminata

MNLPRSGTPRFFQKLQWVINPVGYMETHAQRYPDLFKAEVVGFGDTLVFVSHPQAIQYIL

THDNKQLSTPGKVNEIVEPLLGEYSVIMLDGDRHRRQRQLLMPPFHGDRMRSYGEMIREI

AATVMKEVPTSQPFLARSVMSQISSQVMLKTVFGLDVAGENYHEIKYLMTSMLDVFNTPA

ASGLLFFKQLQVDLGRWSPWGYFLRIREKIDKLLYAEIANRRQQDLSDRLDILSLLMSAR

DTEGEAMTDKELRDELLTLLFAGYETTASAMSWGLYWIHALPEVKNRLREEIASLGDNPA

PMDIFGLPYLTAVCNEVLRLYPVAMLTFPRRVEEPMELLGYSLKAGTDLVGCIYLLHHRE

DLYPDSYQFKPERFLERQFSPYEFMPFGGGKRRCIGAALAAYEMKLVLATILSGYDVKLA

EKGPVKPQRRGVTLSPAGGIKMIKVGDRTPAERPLVASVG

>CYP110M4(AFY81560.1)Oscillatoria acuminata

MKLPAGPRVPFFLQSLQMIANPIAYLERNASRYGDPFTLHLLGGNSPPVVFLSHPEGIQQ

IFTSAAGAFELGKITDPFRPLTGSQSLIMQDGAKHQGTRQLLMPPLHGERLQTYGNQICD

ITSEAIAHWQPGSNLNLRQALSSISLKTILRVVFGMNPGERAHRLELLIEPFLESVNSTL

NSVQFFWNPLQQDLGPWSPWGKFLRQQQQIDELIYAEIRDRRGQTDGSDVLSMLMSAQDE

AGEAMGEAELRDQLITLLLLGHDTTASALTWAFYWIHQDSEVRSQLVEELDSLGSHPNSM

AVAQLPFLQAVCQEALRLYPIALISQPRVVKESVEIQGYEFPPGTVLVPCIYLAHRRPEV

YPEPLAFKPQRFCDRKFSPYEYFPFGGGSRSCIGMALAMYEMKLVLATVLSRYELALCDQ

GPIRPHRRGITFVPSANFQLVMKQRRSAVLSVASR

>CYP120A25(AFY81966.1)Oscillatoria acuminata

MTSNETLRSLPLPPGKLGLPLIGETVSFLRDRDFQKKRREKYGTVYKTHLFGQPTVVLVG

SEANRFLFTHDNSYFSATWPYSTRTLLGPQSLATQSGNEHTSRRRLMVQAFLPKAIAGYL

PGMEQLTHRYLQQWETLGEMTWYPLLRTYMFDIASTLLIGTENGSETAYLSQIFKTWCEG

LFSIPLNLPWTQFGKAFRCRTLLLKKVEEIVERRQQETDFKTDALGLLLAAKDEEGNGLT

LEELKDQVLLLLFAGHETLTSSLCSFCLLLAQHPQVITKLREEQQKVGFTGSLTMEMLKE

MTYLEQVIKEVLRLIPPVGGGFRTVIKSCEFSDYQIPEGWMALYQINQTHQDSSIYPNPQ

EFDPDRFSSDRTEEKHKTFGFIPFGGGARECVGKAFAMLVLRVFGTHLVHGYDWELLPDQ

NLELAIVPTPSPRDGLRVKFSRRG

>CYP2287A4(AFZ05465.1)Oscillatoria nigro-viridis

MNLNLDTIFSSLMNPALNEFQVWALQNPEESLSKTMLPPIALIEGLIANEPTYLQIKRLA

FGANFCCAGQVVMGEFDTLEKALTSPQARGWRLGTSVLDPDRAPNQDVGGRNLFLLSLSD

RETDGSSNHAAFRSCMQKYLFNSATTDRQEDEISRRLLDRLAADYAEMSHGAGGAFFTDV

KRGWMGFLVRYLHYVIFGINPDDSESIELLTELHYTRQSPLHYFAVIGNLLQSLNLFGHG

DLSALIERAATIYENSPALADFEESSENNGMTKRELAKLMTSIMGIAGLQGPLHLGYTAM

GYRPLPAYKGQQTAEINPTDFWDRLDLDDRQSIELFLLECARLWAPVSATHRVATEPFTA

TVAGKERTFPAGTKVLIPLSLGLLDESFWGSTVYEFNPKRENLCPFHMGFHSVGDRSAGR

ICPAKDIALKMLVDVVSTVGKVRRSSEPNLRR

>CYP110E27(AFZ07690.1)Oscillatoria nigro-viridis

MTLLTNNSQSQNEAINKVLDGPKDNRFVQTVRGILNPLYYLERNYQSYGDIFTSKFSNFP

PQVIVSNPQAIQEIFTADSKLFESGTGNQIALPLVGPNSLLLLDGDRHLQQRKLLMPPFH

GERMKAYGQIIRNTTEKVINNWTPGNSVIAHSTMQEISLEVILHAVFGLSEGERYQQIQQ

LLVKMLNFFSNPLSATFLFINSLQKDLGAWSPWGGFLRQRQRLDELLYQEIRDSLRDSFA

SRKTLSQPLGEDILSLLISARDEAGQPMSDVELRDELMTMLFAGHETTATALAWALYWIH

YIPEVREKLLQELNSIDLENSDPAEITKLPYLNAVCCETLRIYPIVFFAFPRILQAPMQL

MGYNIPKGMILSPCIYLVHNRPDIYPEPKRFKPERFLERQFSPYEYLPFGGGNRRCIGAA

FALFEMKLVLAKVLSQYSLELTENSPVLPVRRGLTMAPAGGVHLTVKGRI

>CYP110C25(AFZ09983.1)Oscillatoria nigro-viridis

MTLPNGPKAPPFVQLIQWIGDPLTFMDKCAKQYGDIFTAQVNNGKPLVMIHHPQAVQEML

NSNSFHAPGRLNSLLKPLVGEQSMFLLSGERHKRERQLLMPPFHGEGMRNYGQLICDIAR

DVASKWSVDRPFVARSAMQEVTMRVILQAVFGMDDGPRLQALKPLLASVLDMTNSPLRSS

MLFFGWMQQDWGSWSPWGRMKQQQQKIDELIYAEIAERKEQPDANRTDILSLMMAARDEN

GQPMTDEELRDELMTLLLAGHETTATALAWAFYWIHHLPSVRQKLLQELDSLGENPDPME

IFRLPYLSAVCQETLRIYPVAMLTFSREVLAPVELMGHQLEPGTIVVGSIYLTHRREDLY

PEPLQFKPERFLERQFSPYEYLPFGGGSRRCIGLALAQLEMKLVLATILRDFDLVLAEKK

PVQAKRRGVTLGPAGGVRMALLGRRISRQKREPVASGV

>CYP110C11(BBD54407.1)Planktothrix agardhii

MNTQLPPASPRPAFQEKVYWISDPLNYMDTHTQKYGDIFSNYILGAKTPWVFVSHPQAIQ

KILTDDIFEAPGAFNKILQPLTGDNSIFMLEGDRHKRERKLLMPSFHGERMRDYGELITD

ITQKVIGQCSEGQSFTARETLQAISLDVILKVVFGVYAGERYSQLKRLLSGILDVFKSPF

SSSFLFFPILQQNLGSWSPWGYFLKQRQQIDELLYTEIRERRENLDESRKDMLHLMMAAR

DEEGNPMTDQELRDELLTLLFAGHETTATAMSWALYWIHSQPEVHKKLIQELKSLPENAE

GMDIFRLPYLTAVCQETLRIYPVAMLTFARTNKESVDLMGYQLPPKTPVVGCIYLTHRRE

DLYPQADQFKPERFLERKFSPYEFLPFGGGSRLCIGMALAQYEMKLVLATILLNYELNLW

EKQPVKAVRRGVTLAPKGGIKMQLLKKLPLHIIF

>CYP120A19(BBD54618.1)Planktothrix agardhii

MTSTQSITSLPLPPGKLGFAIIGETISFLRDPDFADKRQKQYGSIFKTHLFGRPTVIMMG

AEANRFLFANENKYFIVAWPLSTRILLGQGSLSMQLGDIHKSRRKILSQAFQPRALARYA

TTMEDFTHRYLHKWEQKSTLIWYPELRKYTFDIACKLLIGKQQATDTNLEVLFEDWCNGL

FTIPLRLPGTKFNQACKSHKLLLTEIETLIRQRQQQPHSGEDALGLLLQAQDEDGNQLGI

EELKDQILTLLFAGHETLTSAIASFCLEVGQRPDIIAKIRAEQQQFDPTQPITFEDLKSM

TYLEQVMKEVLRFVPPVGGGFREVIQDCEYNGYFIPKDWSILYQIAKTHQDETVYFQPQE

FDPERFSETRNEDKPKPFSWVPFGGGMRECIGKEFAKLEIKLFAALLVRNYDWQLLPNQS

LDLITIPTPRPRDGLKVQLRRLND

>CYP110G2(NIES204_43780)Planktothrix agardhii

MKQVETLNSPVWLQRFQYITNPVGYWKSAYQTHSDGFWAKGIDFGSPLLVFYTPEAAQQI

IENRDKHLSTTTFESELKAIFGESSFFTLEGTRHLKARKLLIPMLHGKHIQSYGQLICDL

VNDVLESLPSDRTFSVFDVAQDISMQVMINLLFGSYQEERYQEIKHLMFEMVNLFASNIA

GIPLFFRFLQRDLGAMSPWKKFLKQRQQMKEIIYGEIADRRAHPDQNRTDILTLLMSVSD

EQGNTLTDEELMGQVLSLLFTGNESTAASMSWLWYNVYRDAEIKTKLLAELNSLELSSDP

LNIVRLPYLSAVCNETLRMYPVTMFMIPRMVKSVTEIMGYRVDPGTLITVGTYVIHHRED

IYPEPNVFKPERFLDHSFSLYEFLPFGGGLRGCIGGEIALYLLKLTVAIAVSRHSLKLVN

QHPINPQRRNTILTPMGLKMIKHIA

>CYP110A10(AFY76667.1)Pleurocapsa sp. PCC 7327

MSKLPDGPESFAWWELIKWIADPLAYLEAGARRYGEVFSIKLRGFAPFVIISNPQGIQEI

LSVDAKLFDSGTANDIVRPLVGDNSLLLLDGDRHKRQRKLLMPPFHGERLQAYGQIICEI

SEKIASQWTEGNSFIARSAMQDITLEVILQAVFGLCEGERYQRLKPLLAAMLDLTGSPLR

SSILFFKFLQQDWGAWSPWRRMKRRKQEIYELLQAEIDERRADREIPGNDILSLMLLARD

ENGEPMSDEELKDEMMTLLVAGHETTATALAWAFYWIHKLPEVREKLLQELDSLGENPDP

MAIYQLPYLTAVCQETLRIYPIVPIAFFRLPRKPVEIMGRSFAPGTMLASCIYLVHHRED

LYPQSKQFKPERFLERQYSPYEYLPFGGGNRRCLGYALAQLEMKLVLATILSRYQLTLAD

NKPVKPQRRGLTIAPDSGVRMVLTGKRDRATRAIEPKLVKKEAVAQ

>CYP110D18(AFY79304.1)Pleurocapsa sp. PCC 7327

MLKTKSQQTEPENLTTLPPRPKTPQLLRMLNLIFRPIDYMDDYGKRYGDFLTVGSEKNPF

VYVSNPQAIQEIFTGDPYRFDTGMRGGFIYLLLGANSLLSLDGARHQRERKLLMPPFHGD

RLRAYGHLICEIARQVTEPWQVGKSFQVRSYMQEITLRVILQAVFGLQQGERCDRLRQLI

GSMLDSISSPLSSTTMFFPALRKDWGNWSPWSRFLRQQERVDRLLFDEIRERRERGHLDG

EDILTLLMSARDEEGKPMTDQELRDELMTLLFAGHETTASALSWALYWIHYLPEVQEKLR

SELASLPEGAAPGEIVRLPYLTAVCQETLRIYPIALAAFPRLLKAPLELMGYQFEAGTVL

APCIYLTHHREDLYPEPKRFQPERFLERQYSPFEYFPFGGGNRRCIGMALAMMEMKLVLA

TI

>CYP213A3(ABM77108.1)Prochlorococcus marinus MIT 9303

MASSDDAKLRPLPNTAALSGVLEAFAFFRDPAFAQKRFKRHGNVFETSLLGQPMVFIQGG

QAISDLLAQPNAVEGWWPESVRKLLGSHSLANRNGASHRARRRVIGQLFSASALQRYSAG

IISMVKDLADELQAATTALPLAERMRRFAFSVIATTVLGLEGTDRDELFVDFEIWTRALF

SIPIALPGSSFAKALKARERLLKRLQKVLLKASNGNGGLDLLAGGLDEAGIPLTDEDVVE

QLLLLLFAGYETTASSLSCLMRELLLNPQVETWLREEIDGLDWPPAPEQATTAYDQVNAP

KLDAVVSEIMRLTPAVGGFFRRTKCALVIDGVEVPKNRVVQVALAASNRHGAGDLEAFRP

QRHLDDGCSATLLPFGGGERVCLGKPLAELEIRLMVVGLFHQLRLHLIPDQDLTLQMLPS

PTPRDGLLTKVL

>CYP213A2(CAE21740.1)Prochlorococcus marinus MIT 9313

MASSDDAKLRPLPNTAALSGVLEAFAFFRDPAFAQKRFERHGNVFETSLLGQPMVFIQGG

QAIRDLLAQPNAVEGWWPESVRQLLGSHSLANRNGASHRARRRVIGQLFSASALQRYSAG

IISMVQDLADELQAAKTALPLAERMRRFAFSVIATTVLGLEGTDRDELFVDFEIWTRALF

SIPIALPGSSFAKALKARERLLRRLQKVLLKASNGNGGLDLLAGGLDEAGIPLTDEDMVE

QLLLLLFAGYETTASSLSCLMRELLLNPQVETWLREEINGVDWPPAPEQATTAYDQVNAP

KLDAVVSEVMRLTPAVGGFFRRTKCALVIDGVEVPKNRVVQVALAASNRHGAGDLEAFRP

QRHLEDGCSATLLPFGGGERVCLGKPLAELEIRLMVVGLFHQLRLHLIPDQDLTLQMLPS

PTPRDGLLTKVL

>CYP120B6(BBC23459.1)Pseudanabaena sp. ABRG5-3

MTRYPAEQPIEKPLPPGDMGLPILGQTLQFLFDRNFPAKQYAKYGAISKTNLLGRPTVMM

IGSEAAECVLSSQMECFSWKEGWPDNFKMLLGESLFLQDGEEHRRNRRLMMPAFHGQALN

GYISTMDEITQRYLDKWIEKGEFTWFNEFKKLTFEIASQLLIGSKAGDDTEVERLSQLFT

TLTNGLFVILPLKLPFTTLGKAIKARDRLLQHLTKVVKERQQHPTHDVLSMLIQAQDEDG

SRFSLEELKAQAMLMLFAGHETTTSMLTWFCLELGRHPQVLEQARQEQIALAQTGELNLE

QIGKMTYLDQIFQEIERLHPPVGGGFRGVVKPFEFNGYHVPKGWLALYSIIITHKQPDIY

PNPDQFDPDRFSPDRQEHKQKTFSLIGFGGGARICIGIAFAKLEMKIIASHLLRRYHWEI

LPNQNLEPFPIPTLRPKDGLKVKFKPI

>CYP110C18(BBC24479.1)Pseudanabaena sp. ABRG5-3

MLSLINPVSTIEFLQKMQWVIDPVGYMESAAKEYPDIFTARIIGFNSSIVFVNEPQAIQQ

ILTNDRKQFSAPGELNGILRPIVGDYSVFTLEGDRHKKRRQLVMPAFHGSRMQNYGQVIF

DLTTKVLNNLTPQQPFIAREVMQDISLQIILQTVFGLHDGDRCQQLKSLLTEMTALFTSP

LSSSLLFFPSLQKDLGAWSPWGNFLRQIEAIDQLIYAEIAEHRAKHDSDRTDILSMLISA

IDEEGKSMTDVELRDELLALLLAGHETTATAMSWALYWIHRLPEVKEKLLQELQSVSDRH

DWMSIFKLPYLTAVCNETLRIHPVAMLTFPRMVEEPVELLGHKLDPYTIVIGCIYLLHHR

EDLYPNASQFQPERFLDRQFSPYEFMPFGGGVRRCVGEALAQFEMKIVLATILTNYNLDL

SDRHKVKPQRRGVTLAPAGGIKMVINSI

>CYP284A8(BBC24991.1)Pseudanabaena sp. ABRG5-3

MHTLLSPAMISLSTVFPYLFPTTMIMIAGILGWQYWAKRKRYQSLKALPSPSGHWLLGNI

PQILAAVKKKQFFQLLFDWSKQYGSIYVYWAGAPVVVLSKPSVIESTVINGMRDGSLIRS

QTATRAWNDISGAILLGQSGAEWQWRRKAWNPEFSPVGISAYIGVVEQACSQITNKITAD

VSSESIPVDRLFVELTMRVIASLLLGIPVDSKVVGPEGSPLDVPKVYEAMSVLGYRFLRV

ATGEKPWQKYLPTQTSRDYWAARRYIEQFLTPRVDLALQLRDQQVKDSSSISPLFQESML

VKIASKEPRYTRETLIAEVIELMIAGTDTTAHTLSFTVGELAAHPEVFQKAQAIADQVWQ

KHGAISIESLKELNYIRAIVKETLRLYSVASGSTSLQVVKPTTIEGMTIPVGTKIFWSML

AAGRDAETYSQPDKFLPERWLEEGHGSLSLPMIDFGSGSHRCLGEHLAMLEATIMLAQLL

RNFDWELVNGRASLENLQQNLLIYPADGMPLRFRLRESISI

>CYP1762B2(BBC26498.1)Pseudanabaena sp. ABRG5-3

MIDNLWSREVNNIPLPPLVKGLPILGSSLELAKDNLGFFVKQYHEVGAIFRVRALNRQFT

VIAGAEANQFVNQLGTEFLSGQDFWQDFCKELGTDNLLISLDGDRHLQQRRFLQPSYSRN

SIIHAFPETIQLISDLTANLRSGQRVQVLPFFQQIICEQLGMLLADCSPSHYREDLVRFL

QTALNVTVVKQLPSFLLWLPSYQRSQKRVLELAKLAIKKNRMAQTKRQKPNLIDDLIADS

QQPHPLLSESEMIAAAVGPYLAGLDTVANTCSFMLYALLKSPEIMAKVIQEVDWLFQDGI

PNAEGLRGLSALHGAVMETLRMYPVAAAIQRYAIADFTFKGFRVDAGTHVIIATTVPHFL

PQYFPNPYTFDIDRFHPPRNEHKQNGAFAPFGVGAHLCLGNGLAEVQIMLTMAMLLHRFE

FSLESPNYKIQPISNPTLSPSNRFYVRVQTRSTQRQK

>CYP1007A7(AFY53209.1)Rivularia sp. PCC 7116

MTKLTVPGIQPLPVFGRTILVSKFVKDSLGFTSQLFQKYGKLVSLARGGSTNLYSPLPNC

PGTVFTYGAQNVQTVTSQHEIYHKYPLTGGLYSRRDESPRTEPLKHFGVGLFGVNNDEHR

RHRQLLMPAFHQKRIEYYCNTFKEITESISQQIEVNKVTDIAKLMRMLTLRIATKTLFGA

DIGEKGSKSAELLKQIGILLGNPTTMVFPFDLPGFPFYRMMNLMAELDEDMREIIRSRKA

SDSDTGDVLSMLIKASDAETGMTLSEDELLGHVSVIFAAGHETSANALTWTLFLLSQHPE

VAADLLDELDTVLQGEAPTIEQLQQLPLLERVIKESMRILTPVPWNARATSQPTTLEGYE

LPTGTEVWVSIFHTHRTPDIYPEPLKFNPQRWETLEVDNFQYNPFSAGSRKCIGAGFALM

EIKIVLAMLLQKFRFQCLPQQKITRSALIVLAPKGGLPMLICPQDREFSQGVGGLRGNIR

ELVELQ

>CYP284B1(AFY54046.1)Rivularia sp. PCC 7116

MSLIVLAGSGIAIATTSVFALRWWSNYQKYSSLRNFPSPKGHWLSGNFSDLVAALKAGKL

PQLFIDWSKELGDVFVIWNFDKPNLVIGNAKLIKQILLKGQKDGDFVRGGSAYSAYADVF

GVHLGNQIGEEWQWRRKAWTPALASSKFLNQFDVINQASLAFIEKLEQKAINNEAIEIDP

LFLKYTMSIIAYFMLGVPLIPQEDPIKPILDSEKIYSSLAVLEKQVLVQGTTGFNWLKYL

PTKKNQVYREAQNYLNGFLHPRIDLAFKLARNQELNQNELSQISQPLRNSIMVQMAKAPR

YTPNHLINDVRAGLFAGHDTTSHSLSFAMGELALNPQVLQKAREKIEPVMKQGLSIESLK

KLVYIEAIFKETMRLHPVASQLSIVAKHDTSMVEKFIPADTAIRLNLMLAGLDSDMYAQP

EAFHPERWLDVEENGGIEPTLFGFSLGAHYCLGAPLAILEATVILSLLIYHFDWELVNGR

SSLEQLGQNITVFPQDRMPIKFKARKFANMTT

>CYP110E19(AFY54671.1)Rivularia sp. PCC 7116

MEQVKGPKTPKFAQLIEWIFNPLQMMEKSAKAYGDKFKLFLVGDNPSVFVHHPQAIKEIF

TASPDKFDSGRGNQIIASLLGEQSLVLLDGSQHQRQRKLLTPPFHGDRMKSYGDLICKIT

EEVISDWKIGEPFAVRTSIQEISLRVILQAVFGLNEGERYDEIKQLIASVLDVSGSPLRA

MLVFVPLLQKDLGAWSPWGHFLRQQKRLDDLLYAEIEERRDNPDASREDILSLMMAARDE

QGQPMTNKEIRDELMTLLVAGHETTASSLTWAFYWIHHLPEVKEKLLAELDKVDTSDDLS

LIAKLPYLNGVCAETLRIYPIALIAFPRINKAPIKIMDCEYPAETWLTPCIYLTHHREDL

YPEPKQFKPERFIEKQYSPYEYLPFGGGNRRCIGMAFALFEMKLVLTTVLQKLDLAIVNN

QEVKPTRRGGTLAPSDGKWLVATAKRNMKVKVEV

>CYP120A13(AFY55027.1)Rivularia sp. PCC 7116

MATINPKKLPVPPGNLGLPLIGETISFVRDADFTEKRYQKYGSMFKTRIFGNPTIIMIGS

EANRFLFTNDNKYFSNQWPPSTRILLGPASVAVQRGNIHQKRRKILSQAFQPRALSEYTS

TMEEILQDYISKWEKTDTLTWYPEIRKYTFDVACKLLIGTNKASDSELLELFEEWIAGLF

TLPIRLPGTKFSKALRCRQLLLQKIEEIVLQRQQQPASNKDALGILLQAKDDDGSSLGLE

EIKDQVLTLLFAGHETLTSALASMCLLLAEHTDVFRKIREEQQQLGFSQPLTAENLKQMT

YLDQVIKEVLRFSPPVGGGFREVIESCEFNGYLIPKGWTVSYAVPKTHQDSSIYTEPLKF

DPERFAPSRAEDKSKPFAHIPFAAGMRECIGKEFAKLEMKLFAALLAREYDWQLISENNL

GNNLTSASVLKDKLKVKFRKIGNRE

>CYP110F3(AFY55460.1)Rivularia sp. PCC 7116

MTNSLVQTLQLIANPINFFDNHAQRYGDTFSLRVLGINSPPVVFFSSPQAINDCFAIPAN

KLDFKKATHVFKPLFGSDSIVFQEGKSHNRQRQLLMPPFHGDRLKSYGDTICNITNNVTS

NWKTGNTVSMQEVMPDITLQIILQVVFGITPGERYEKLINLLTALLNDITKPLFSSLFFF

PPLQQDLGKWSPWGNYLKRREEIDKLIYAEIYERRLSDDNLGEDILSLLMSASDEDGGKM

TDKELRDQLVSLLLLGYETTSGVLAWLFYMIHSHSEVYSKLIEELKTLENTASPENIAQL

PYLSAVCSETMRLHPIALICTPRMAVDKVEVAGEKFGEGTVLVPCIYLAHRQQTTYQQPD

KFIPERFLDRKFSAFEYLPFGGGYRGCIGAAFAVYEMKLILATIIQKFQLELVDKKPVKP

VRRGITIVPSGGVSMTVKSCI

>CYP110B14(AFY55465.1)Rivularia sp. PCC 7116

MKLPNGPKIPAFIQQIRFIAFPMSLLEDCAKGYGDIFTLTFLNISIVIVSNAKALQKLLN

DKEFTAPGDANKIIEPLVGSNSVITISGEKHRRQRQLLMPPFHGDRMRTYGETINNVTQE

IISDWEVAKPFNVREAMQKVTLRVIMQAVFGLYKGKRAEDIENHIKEFLEAGGSPFGTMA

LFFPKLQRNLGSLTPWGSFVYHRDLADKLLYQEIDERRENPDDSRTDIMTMLMASKDEQG

EAMTDEELRDELMTLLFAGHETTATALTWALYWIHKIPEVKQKLLAELDSLGENPDSNAI

LKLPYLNAVYCETLRIYPVALLTFRRDVESPVSLCGHELEPGTAVMGSIYSIHNDEKLYP

EPKKFKPERFLERQYSPYEFIPYGGGARRCIGMALAQFEMKLILARILSDLDLDLITEGE

VKPKRRGIVTGPNRPIQMVVKGKRMAKSRSLETVS

>CYP110D15(AFY56144.1)Rivularia sp. PCC 7116

MPIAHCELIFSSQYKEVFKPQVIMTSNYNLPPTVEMPIFLRRMKFIFQPFQYLEQNAQLY

GDTFSLPQKDGSSTVYFGQPEALQQIFTADPKSLETGRGSRILRFLVGDNSVLLLHGDRH

QRQRKLLTPPFHGDRMRAYGMTICEIAQQVSDEWQTEKPFNIRESMQEITLSVILRAVFG

LDEGNRLQKIRLKISSLLDYLSSPLLSAGMFFPVLQKDWGSWSPWGRVLYLRQQIDDLIY

DLIEQRRAESNPNREDILSLMMSARYEDGGEMSNQELRDELMTLLFAGHETTASALTWAF

YWVDYLPEVREKLLKEINDLGENAEPNLVAKLPYLTAVCQETLRIYPIVPSATPRIASCS

MEIGGYTLPKGTWIIPSIYLAHYREEVYSQPQQFKPERFLERQFSPYEFFPFGGGNRRCI

GLAFAMHEMKLVLATILSQYEVSRIDKRPLKPVRRGLTLATPAGMKMVAKPIKKVANTPV

AV

>CYP1136A5(AFY56215.1)Rivularia sp. PCC 7116

MQQAKSEVIILEDGQTPPAQTQAPIRKPQWYDTFSYIANPDKFCRHNLEKYGAIFKTSVF

GGTTIFVGESKAIQMVFNGDSNYTEIALPPTTMDMFGEHSLFQRPDLHRQRKNSLGPGLT

GRFLEGYIPHINNEIKKGLHKWNTPGKIAVYPEVEKICFDVLTPLLWGVKLDDDNPESFN

GLPIKNKQEVKDLYKTYFDGFYGLLKWESPLTAYGRGIKARKKLIEFMRAVIKQRKKEEI

NPKSDFLAMMLASQQENPDGIFSDALVENQCLLEVWASYYQISALVSSLIYQLGKYPQFV

KKLRQEQNELINEKDISLELLKQMIFLDATIKETLRISPPSSTANRRLIKSVVLDGILYD

KGCTVIAEPRLAHIMKEYFTEPNKFDPERFLAPRNEGKMYEFIPFGGGVHACLGAQMAMI

VTKIFAYHLLNLFDWESTGEASFVQFPLKKIKDNYQINLQSYQI

>CYP107NM1(AFY56909.1)Rivularia sp. PCC 7116

MSISTIERLNPFVKEVISNPYPYYSRYRKADPVHWGVAANPKLDGAWYLFRYRDVMKVME

SPKFGREASKVRNDGAGAPVPKAYRGFSSMVSNWMVFRDAPTHTRLRTIVNKVFSAKMVE

NLRPAIIGIADCLLDRVHLQGEMDLVEDFAFPLPIMVIADMLGVDPKDRPLFRKWALALQ

HASASRLTPSPEVYEQAEAATQGFIEYFKKEIAKRHQEPGKDLITALVKARDEGNKLNDE

EILATCIHLLTAGHETTVNLISKGILALLRNPRQLKLLRSHPELMPAAVEELVRYDSPVQ

MISRWAYEDFEIDGKVIKRGDSVNLILGSANRDEEQFENPDELNFEREAKKHSGFGSGIH

YCLGSSLARAEVTIALNTLINRLPNLTLKEDSIEWANNIVFHGPKHLQVGFRSPRDAKGM

WGHDHV

>CYP1184A2(AFY56911.1)Rivularia sp. PCC 7116

MNVQDKTQSKIQGCPFHKEKIGEEYQPFVNPQLEDPYSFYDFARSEQPVFYSSVLSGYVI

TRYSDIISILKDSVKYSSKDNLQPIGEYTKETIDVLRTGFPFVSDLVNSDGDRHKFLRAP

LQKAFAPAKLKSMEGSIAAIVNRLIDNFINDGRVDILDKFAYPLPLEVIFTLYGVPLEMI

PDFKHWGYQTTKLFSSALSPEEQLECARSFVSIQYAVANLIEEKRFAPKDDLISTIIDSE

LTTPDMVIVLYGLIVAGHKTTSHLIGNALKVLLEKPGYWKAISENPSIIPAVLEEALRYD

APIPAMIRTNNEEVEIAGVKIPENSKLFLMYGSANRDENHYQNASEFDIKRFQNQTSDHL

AFGHGVHRCIGSGLALMETRIAFELLSKRIPNLKIQPNQELNYIPTLMTRGFTSLILEW

>CYP1184A1(AFY56912.1)Rivularia sp. PCC 7116

MIQQNNTQSTKTCPFHAGKEYQPFVEPQLENPYSFFQIARNQEPVFYSSVVNAYVITKYE

DVLNILKNPTIYSSAKSLQTAGDMTPEAGKVLQQGFPFVSLINSDGEKHRRLRSPFLKVF

AADKLAKVEGSIYAIANRLVDNFINDGSVEIVSKFGHPLPLEVILTMYGVPLEKMEQVKK

SGSDISMFFSSKLTPERQIECAQSYVSLQHYIASLIEQKRSAPGNDLISQLLTSDLTTPE

IVLMLCEMIIAGHKTSANLISKALKLLLDTPGAWQALHENLSLIPIAVEEVLRYDTPAQS

MIRVTTQEVTISGVQIPKDSRLLVLYGSANRDSEKYENADKFNIERFKDAPVDHLAFSHG

THHCTGSNLARREVRIALEVLSQRLPNLRITSNQELHHLPILTNRGYESLYLEWD

>CYP110A6(AFY57624.1)Rivularia sp. PCC 7116

MSSQLPNCISTPPWWQLINWIGDPIQFQKKYSQKYGDIFSMRLFGLGSYVVIANPQTIQE

LFSKDAKFDIGRANALAEPLIGRNSLMMMDGDRHRRERKLLMPSFHGEKIQVYAQQIIDI

TENITSKLQIGESFIVRSTMQKVSLEVILQVVFGLSEGSRYEQLKPLLTDWLDMTDSPLR

SSMLFFKFLQKDWGSWTPWGKMRKRQQQVRDLLQAEIAEKRSKTETLNKNNNVLSLMMSV

RYEDGQAMSDEELTDELLTILFAGHETTATTLAWAFYQIYQNPDVLEKLQIELASLGENP

NPLEIAKLPYLSAVCQETLRMYPVLPVLFPRIAKSPIKIGGYQFDAETTFMPSLYLVHYR

EDLYPNPQKFQPQRFLERQYSPSEYFPFGGGSRRCLGYALALLEMKLVLATIISKYQLAA

LDNKPVKIQRRGFTLAPAGGVKMVVKGIGNR

>CYP1185A1(AFY57880.1)Rivularia sp. PCC 7116

MSKLANNQTAPLEFNPRSPQFRANPYPTYDYLRTHHPIYYRSERNDWVLTRYDDIVEVFR

NPSFGRSQQKPKLQTANQQPINHFLSLRQESQQLMKLWLVLLNPPDHTRIRHLLRNPFTP

SRIQTLRSHISANVNNFIDGVKNSGKVDIIKDLAYPLALGVNCKILGIPEQEWHPRFKQW

SDNLSIIADVDVTPIANEQGLLTIAGLAEYFRSWIAKCRSCSQPQDNLIGSLIEAEANGE

ISEEELLGTCIFMFAVGHSSTANLIGNTILTLLNHPQQLYLLQADPSLIETTISEVLRYE

SPVQGISRTALSDIQLSNQTIHRGEVVNCIIAAANRDPAKFLEPNKFDIRRKPNPYLSFG

QGIHNCIGKHLGRLVAEIAVGTVVKRLPELSIATESFEWDDSFLGRGLKSLPVIF

>CYP197B4(AFY58376.1)Rivularia sp. PCC 7116

MTSNQGQSILSLSGPDAATAIEEYGQDPLAFMTGCARKYGEIVPIQLENDLFCLLTNPEH

ITQVLRDRQLFVKAEDMELLKTLLGNGLLTSEGSFWQRQRRLAQPIFHQRRINGYGETMV

EYTQRMLENWKAEDTLDIHQEMMHLTLNIVMKTIFNQDIAGGDAGNVAQAVEEAMNWFVE

KTNSLLAGDETKTPADKRYEDAIVLLDETVYAMIEHRRETGEYGNDLLGMLMKVEDADDG

SRMTNRQLRDEVATLIVAGHETTANTLSWAWMLLGENPDIRAKLDEELKAVLQGNAPTIE

DLQRLPYTTMVIKEALRLYPTVTDLSRQATEDCEIGGYSIPKGTTLNISQWVMHHDSRYF

TNPEVFNPERWANDFEKTLPRGVYFPFGDGPRVCIGKSFAMMEAVLLLATIAQSFHLELV

PNQVIEKQPSVTLRPKTGIQVVLKSV

>CYP227A5(AFY58434.1)Rivularia sp. PCC 7116

MTIKEQVIKSQKRQIELSRMLAKVGYDSKIGQTLRLRFYYQDAFLLLRKFKSFLYTAKEL

FGDKFFGGDQAIIHTSHAEVKKLMQVEPQLRGNDLGIIRMLAPSYLLNNPLSLGMNGTQH

TGARALFLQALTNPAEDIENLSNLVNQCLSEAAQKKELHIGKDLPRMMLKILHQVVFQIS

LSEEEVTASTGYNKKLFLATLPNFINKYTLGLLTAPSVKHRKQLTQRYKQSPLWSSYVET

GAKYNLNEHQIANSLFDMIHIAGTLGTSALLGSTIGVLCLDEALRNNVISEVNTVWNEQE

TPNPDALKNSILINKVILETARLYPAVRFVSQLAQEGGEIEIGESKCPFQKGTRLIGSIF

TANRDAARYENPDSFDSERDFSDILSWNGHGHERACPGRDLSIGLINIFVLYLFKKYKWD

SITEVKWEFEKVTAVTPNELVLQGFAKR

>CYP110D23(AFZ34062.1)Stanieria cyanosphaera

MRQPPGPPNSRWLRKLRLFKFIFQPLEYFNENYQRYGDIYQLGRQNSPPFVVLSNPQAIK

EVFTTPSEQFEIGKNNTDLKFLVGENSLIIQDGEFHQRQRKLLMPVFHGECLQSYGEQII

EITKQVTNQWQTGKLIRVRTYMQEITLRVILQIVFGLNSQQSRYEQLRQLLCAWLEIISS

PLSSSLIFFEFLRQDWGASSPWGRFVRLRKQIKELLYEEIKARREQQPIGNDILSLLLAT

TDESGKPMTDEEIHDELITMLMAGHETTASALVWALYWVHYLPQVKEKILHELNELGEER

TWHQIVQLPYLNATIAETLRIYPITTGTFTRRSKAPLQVLDYQFPAGTAFGISIYLTHWR

EDLYPQPELFRPERFLERQYSPYEYLPFGGGNRRCLGSALAQMEIKLVIASILSDWQLAL

TNHLLLKPVRRGLTIAAPNNFKIKVCDRFNN

>CYP110C23(AFZ35316.1)Stanieria cyanosphaera

MTRPPKLNIHPLIQRLKWVADPVGYMETAAQQHPDIFAAEVIGFGDQFIFVNHPQGIQQL

LTQDRQQFFASGKENAILKPLLGEYSIVMLEGNPHRKRRKLLLPPFHGERMQAYGKLIWN

LTDKIFAQLPINQTFTARKITQEISLQVILEAVYGLYEDEKSQKLKYLLTKVTDVFSSPL

SSALLFFDWLQKDLGAWSPWGNFLRQQQEIDSLIYGEITERRAKGYENRNDILSLMMSAR

DESGDPMSDRELRDELMTLMFAGHETTATAMAWALYWIHRLPEVRQKLVAEIDSLGSNPE

PMAIAKLPYLTAVCQETLRIYPVAMLTFPRVVTEPIELLGYQLEPGMIAMGCIYLAHQRE

DIYSNHHQFKPERFLEKQYSQYEFLPFGGGARRCIGEALAQLEMKLVLANILSNYELTLV

SQSPEKPHRRGVTLAPTGGVKMLLKAKRTLSTVAEKPNQTILTN

>CYP110AM1(AFZ37131.1)Stanieria cyanosphaera

MTTITPSPTQTKPPICKTPVFIQTIQVMFDQFGVLEKYHQKYGDIFYTPRSPGFPPFVIF

NDPKAIEKVFTADPNLFEVGRQTSLPIRVLLGERSLVALSGVQHQRHRKLLMPPFHGERM

KSYGETMIEITKQVISQWQVGKPFSLRTYTQDISLRVILETIFGLDKGEKFDRLRKLLVE

WLDIFNSPFRSSLLFFPLLQKDLGAWSPWGQFIRLKRMIHEILDTEIDRRRNNPATLGED

ILSLMLLARDQEDQPMSNEEIRDELMTMLFAGHETTANSLAWAFYWLHYVPEIEEKLKQE

LNSLEGNLDFNTINKLPYLNAVVSETLRLHPVVALIGRQLKAPFEIMGYTFEAGTSLFPA

IYLTHQREDIYPEPKKFKPERFLERQFSPYEYLPFGGGNRRCLGYAFALFEMKLVLATIL

SEVELELLDRRFPKTVRRGITFAPAGGVRMRVKKYKN

>CYP120A12(AFZ37324.1)Stanieria cyanosphaera

MTISQKTKQSLSLPPGNLGLPLLGETISFFTDPNFNQKRLNKYGKLFKTSLFGRPTVVMV

GAEANTFLFKNENKYVVATWPKSTKILLGSTSLAVKTGDFHTSRRKLLYQAFQPRALASY

IPTMEHITQEYLNKWEKLNTFTWYPELRNYTFDIASSLLVSTDGGSQTPLGEYFEEWCAG

LFTLAIPLPWTKFGKALYCRKKLLQYIEDIVVKRQQAKNPGEDALGLLIQAKDEEGNSLS

LEELKDQVLLLLFAGHETLTSAIASFCLLTAQHPEVLQRLREEQQQLSLASPLTLENLKQ

MTYLEQVLKEVMRIIPPVGGGFREVIESFEFQGYQIPQGWNIQYQIAQTHKDQAVYPDCD

RFAPDRFSPDKAEDKQASFAYIPFGGGLRECLGKEFARLEMRIFASMLLKNYQWELLPNQ

SLELLTIPTPHPRDGLKIKFSPLV

>CYP152E4(Sta7437_4667)Stanieria cyanosphaera

MTQIPQDRSIDSILALAFDDYKFISQRCQRYQTDIFQTRLLFKKTICLRGEEAAKVFYDP

EKFIRKNAAPKRVQKTLFGEGGVQGMDGDPHRHRKQMFMSLMSDENIQQLADLLDQQWKA

YAKKWSTMDKVVFFKEARAVFCRAVCTWAGVTLPESQVKQKTEDLGAMIDASGAVGLKHW

RGRLSRQRTEKWIEGIIEQIRNNRLEIPQESAAYIIANHRDLEGELLDIHTAAVELINIL

RPTVAIARYATFAALALHEHPQCRQKMHDGGEEYCEWFVQEVRRFYPFFPFAAAIVNHDF

DWHGYHFTQGTRVLLDLYGTNHDPQLWSNPEDFYPERFRDWNENRFNLIPQGGGDYYINH

RCPGEWITIALMKVTLNFLTQSLKYDVPEQNLEISLSKMPTIPKSGFVISNIVAPN

>CYP120A12(BAU63288.1)Stanieria sp. NIES-3757

MTISPKAPQFLPLPPGNLGLPLLGETISFFTDPNFNQKRLNKYGKLFKTSLFGRPTVVMV

GAEANTFLFKNENKYVIATWPKSTKVLLGSTSLAVKTGDFHTSRRKLLYQAFQPRALASY

IPTMEKITQEYLEKWEKLNTFTWYPELRNYTFDIASSLLVSTDGGSQTPLGEYFEEWCAG

LFTLAIPLPWTKFGKALHCRKKLLQYIENIVVKRQQAENPGEDALGLLIQAKDEHGNSLT

LEELKDQVLLLLFAGHETLTSALASFCLLTAQHPEVLQRLREEQQQLSLASPLTLENLKQ

MTYLEQVLKEVMRIIPPVGGGFREVIESFEFQGYQIPKGWGVQYQIAQTHKDPEVYPDSD

RFDPDRFSPANAEDKQASFAYIPFGGGLRECLGKEFARLEMRIFASMLLKNYQWELLPNQ

SLELLTIPTPHPRDGLKIKFSSLV

>CYP110D23(BAU63586.1)Stanieria sp. NIES-3757

MRQPPGPPNSRWLRTLRLFKFIFQPLEYFNENYDRYGDIYQLGRHTSSPLVVLSNPQAIK

EVFTTPSEKFEIGKNNAGLEFLVGNNSLIIQDGEFHQRQRKLLMPVFHGECLQSYGEQII

EITKQVTNQWQIGELIRVRTYMQEITLRVILQIVFGLNSQQPRYAQLRQLLCAWLEIISS

PLSSSLIFFEFLRQDWGAWSPWGRFVVLRKQIKELLYEEIKARREQTQPIGKDILSLLLA

TTDETGKPMSDEEIHDELITMLMAGHETTASALVWALYWVHYLPQVKEKILHELNELGEE

RTWHQIVQLPYLNATIAETLRIYPITTGTFTRRAKAPLQILDYQFPAGTAFGISIYLTHW

REDLYPQPELFRPERFLERQYSPYEYLPFGGGNRRCLGSALAQMEMKLVIATILSNWQLA

LTSNQPLKPVRRGLTIAAPSNFKMKVCDRSMPQ

>CYP110C19(BAU65893.1)Stanieria sp. NIES-3757

MTQPPKLNIHPLIQRLRWVADPVGYMETAAQQHPDIFAADVIGFGDGFIFVNHPEGIQQL

LTQDRQQFFASGKENAILKPLLGEYSIVMLEGNPHRKRRKLLLPPFHGERMQAYGKLIWD

LTDKIFAKLPINQTFTARKITQEISLQVILEAVYGLYEDEKSQKLKYLLTKVSDVFSSPL

SSALLFFDWLQKDLGAWSPWGKFLRQQQEIDNLIYSEIKERRAKDYENRNDILSLMMSAR

DESGNPMSDRELRDELMTLMFAGHETTATAMAWALYWIHRLPEVRQKLLAEIDSLGSNPE

PMAIAKLPYLTAVCQETLRINPVAMLTFPRVVTEPIELLGYQLEPGMIAMGCIYLVHQRE

DIYPDHQEFKPERFLEKQYSQYEFFPFGGGARRCIGEALAQLEMKLVLAKILSNYELALV

SQSPEKPHRRGVTLAPTTGVKMLLKAKRTSPTATEKSNQTILTK

>CYP110AN1(STA3757_48960)Stanieria sp. NIES-3757

MTSIEFITKNVSNKSTNGFTLHRLNMLRWVLSPLEYMEEYAAKYGDTFMLRLPYDGSKLA

IFSHPQAIQEIFTATSDFVESGPANRTIEPILGSNSVLMLDGKPLAKRRKLLLPPFHGER

MQAYGDIIKTTSIEVSDRWKVNRTFRLHSFMKETTLQVIFQALFGLKSESHHQQLKQALE

KMLWLFNSPINNLLIFLPALRQDWGKFSPWGRFLRQRKQLENLLVAEIERCREQANPERT

DVLSLLVSARDETGEALTNQEICDELITMMIAGYDTTAKALAWAFYWIHHVRGVREKLLA

ELATLGENADSMAIFRLPYLSAVCSETLRISPIAIIPFRRIVKSPIEIMGNLFEPGTQLV

PCIYLTHQRCDLYPEPKQFKPERFLERQFSPYEYFPFGGGSRRCIGQTFALFEMKLVLAT

ILSRCELQLTDRRPIRAVRHGFTVSPSGGVKMIMTGRKERN

>CYP110M2(BAD78378.1)Synechococcus elongatus PCC6301

MLPPGPSQLALLQTLRIITQPVSFLLSCADRYGDWFTLRVLGPQSPPVVFVSDPEAILAI

FSSLADQLELGRIADVFRPLVGNESLIMQNGDRHRQQRQLLMPALQGERLFDYTPAMTAI

TQAAIAQWPLGQPLDLRRQMSQISLAVILQVVFGLTPGPRYRDLYQRLDQLLEAITDPLY

SLQFFWPALQQDWGNWSPWGRFCRQREAIDALITAEIQEGRQSQQPRQDVLELLLAARDR

DGNPLSDQELRDQLMTLLLLGHETTASALTWAVFWLLRHPDCLNRLQSELVAIGDNDRAI

AKAPYLDAVCREALRLQPIALIAQPRRVASPLSLGGYDFASGTILVPCVLTAHRRAATYP

NPDQFQPNRFLERRFSNGEFLPFGGGQRSCIGMALSLIEMKMVLATLLRQCQIAEVSQRP

VRPARRGITFVPSQDFRIQVQQWHNPSAQTAIASV

>CYP110M2(ABB57397.1)Synechococcus elongatus PCC7942

MLPPGPSQLALLQTLRIITQPVSFLLSCADRYGDWFTLRVLGPQSPPVVFVSDPEAILAI

FSSLADQLELGRIADVFRPLVGNESLIMQNGDRHRQQRQLLMPALQGERLFDYTPAMTAI

TQAAIAQWPLGQPLDLRRQMSQISLAVILQVVFGLTPGPRYRDLYQRLDQLLEAITDPLY

SLQFFWPALQQDWGNWSPWGRFCRQREAIDALITAEIQEGRQSQQPRQDVLELLLAARDR

DGNPLSDQELRDQLMTLLLLGHETTASALTWAVFWLLRHPDCLNRLQSELVAIGDNDRAI

AKAPYLDAVCREALRLQPIALIAQPRRVASPLSLGGYDFASGTILVPCVLTAHRRAATYP

NPDQFQPNRFLERRFSNGEFLPFGGGQRSCIGMALSLIEMKMVLATLLRQCQIAEVSQRP

VRPARRGITFVPSQDFRIQVQQWHNPSAQTAIASV

>CYP213A8(ABI45311.1)Synechococcus sp. CC9311

MMAHAHLPTTGALTGVFESLAFFRDPSFAKRRFRAHGDVFETCLLGQRIVFIQGDQPIAD

LLAQGGCLEGWWPKSVQLLLGSRSLANRNGDGHKVRRRVVGQLFSSAALRRYTPEISVLV

QKLVDELLNSTSPQPLAPRMRRFAFSVIANVVLGLDDRNRDECFADFEIWTKALFSIPIS

IPGTPFAQAMNARTRLLTRFKQLLANDTPLRGGLDLLRAGVDESGISLDDDDLAEQLLLL

LFAGYETTASSLSCLFRALLIDPTVNTWLLSELDAKSAQDDLDRSLPRLDATVLEVMRLT

PPVGGFFRRTKAPVILSGIEVSADRVIQVVLTPSSPSDDSDFFDFRPQRHLDGSFHQTLL

PFGGGERVCLGKALADLEIRLMALGVLKNIELQLHPNQDLSLQQIPSPTPRDGLVITSQR

RLE

>CYP110L1(ABI47297.1)Synechococcus sp. CC9311

MLEAILRPIAYYRRCFFRNSGVVRVRMSPTLPPQQVLISDPSVIKELINEDGGRHITAPG

QLNGLLSQVLGKHSIILLAPATHRQRRKLLTPPFHGERLKAYGQLISSLADQSLIDLNVG

DVFDAREQMQGITMRVILTAVFGLHEGDAFRQLERSLASSISIRSGPLGSLLLFFPFLRR

DLGRWSPGGRIKAADAAIRRLLLEQIASRRRAVERCGEENMSADILSLLLSCKDDEGQGL

SDDELHDELLTLLFAGHETTATALTWAFYWIHRNPLVLERLMNELNGLLDHSDPEAIARL

PYLSAVVNEVLRIHPVAMLTFPRRIESAITLGGYAFRSGDVVMACIQAVHERPDLYPKPL

QFNPERFMNQTYGLHEFLAFGGGSRRCIGAALALYEMKLILAKILLNNQFELTPKSNRWN

KPRRRGFTLGPSIPVKLKMVSLRP

>CYP1851A2(ABI45493.1)Synechococcus sp. CC9311

MFMASYPIHSLAFVLVPSFTILFLTISEDLSSFVDVALGSVGKWIELSGQRTEPFLDALL

SHYEFVLFAAVLLYFFVRSLRMLFLYSLQLIEFVVRVLRFVGDLLSELFWVNPRFRINRE

FSYRSDTSFTLVSIFARFVKEVLLLPIDWIRSLGINGVFGPDPLTESKHYRKTAFYPSLL

QRLLVIKNKHITVYFFLSMMRLFVHRFTLPAWFPGVNIKENSHGDEFPVMRQLHLLKRHD

VEEVLERSEDFRVVYGPRMCDVTQNGHSSGNFLLGMQSTSPTYIRDISNMRLIFRRDDVH

RCRRIAADASVDSFLAFESRVDVLRDLDGSKVLSLPKDLVLPVIKSFIEQYFGVRLPLQA

ENADGTTEIDFNFVWFSHLFNYIFYDLNGDDSREAALASAVLLNQDLDRQIAAVKQQSSK

HLEPANTVLARCLRLQESGTPGMDDLAIRINLTGFLVGAVLPLINTICQVIDELISRPRI

LEQAVSTATSATEGGQSFLNPLQGFVLEALRFSPGDPVIYRHCKTRTELVSASCRSSVSA

NTLVMAWNSSAMFDPKYVDQPWQFNPTRPPRDYLHFGHMYHVCAGKYIVMSVIPAIVQEL

LSRYELFRIPGARGYPVKHGIMVSGFDVLVRPRGSAD

>CYP213A4(ABB35278.1) Synechococcus sp. CC9605

MATTPLPSTGALSGLGETLAFFTQADFAQRRFETHGDVFETKLLAQRMVFIRGERAIGDM

LGQGDALQGWWPESVRQLLGSRSLANRSGPGHKARRRVVGQLFSSAALARYTPSIEQLVA

ELCHELITTNTALPLAARMRRFAFAVIATTVLGLDGASRDALFADFEIWTRALFSIPLAI

PGTPFAKAMAARQRLLKRIKGVLQAGTNQGGLDLLSGGLDEAGIPLDDDDLAEQLLLLLF

AGYETTASSLSCLFRALLLNPEVESWLRDGLNKDPASPKLDATVMEVMRLTPPVGGFFRR

SLAPIELAGVAVPEGSVIQVVLSPTSDGDDDDLASFRPQRHLDGSFKQTLLPFGGGERVC

LGKALAELEIRLMAVGLLQAVELQLQPGQDLALQLIPSPTPKDGLLVQAAAR

>CYP213A9(ABB25927.1)Synechococcus sp. CC9902

MSRPLPSTGAVTGIVEAINFFRDPDFATRRFTEFGDIFETSLIGQRLVFIRGDKAIADLL

AQGDAVEGWWPESVRQLLGSQSLANRNGSDHKARRRVVGQLFSSAALKRYTPGMIGLVDE

LSQELLEETKPIRLADRMRRFAFRVIATTVLGLEGSDRDALFHDFEIWTQALFSVPIAIP

GTPFANALAARRRLLDRLRDVLEQADQNRGGLDLLAGGLDEAGQPLTADDIVEQLLLLLF

AGYETTASSLSCLMRACLIEPHVEPWLREELDGLEWPPQGDATTAFDGNRAPRLQAVVTE

VMRMTPPVGGFFRRTCRPIELANILIPKGHVVQVALASSNRAGSSELNEFRPQRHLDGSN

KPTLLPFGGGERVCLGKALAELEIRLMVVGLLKRVQLSLAGDQDLDLQLIPSPSPKDGLK

VVAKHYAPADAKA

>CYP213A17(AII45007.1)Synechococcus sp. KORDI-49

MTGTLRPLPTTGAVTGILEALGFFRDPDFASRRFHEYGDVFETTLIGQRLVFIRGEQAIR

DLFDQSDAVQGWWPKSVQTLLGSRSLANRNGAAHKARRRVVGQLFASAALRRYSPSIVAM

VDGLADELLQSETAVPLAERMRRFAFAVIATTVLGLDEDDRDALFTDFEIWTKALFSVPL

AAPGSPFARALAARSRLLKRLQTVLQEADGGRGGLDLLSGGLDEAGLPLTDEDLVEQLLL

LLFAGYETTASSLSCLMRALLLDQELMPWLDEELDQLTWPPQGDPTSAFDPSRAPRLQAL

SSEVMRMTPPVGGFFRQTIEPIGLADVEIPAGRVIQVALAASNRQGAGDLETFRPQRHLD

GSSQQMLLPFGGGERVCLGKALAELELRLMTVGLLKRVRFSLVPGQDLDLQLIPSPSPKD

GLLVSSAPR

>CYP1851A3(AII48273.1)Synechococcus sp. KORDI-52

MAMDIQIIHPVASVLMQPSTLSSMLSDDMTAFLYALTANVWTYIETLAFKLKPWLNSLIS

HYQLLILAAALVYFVARSSRMVFLYSLQWIEFAVRLLRFLGDAFSQLFWVNPRYRTRRAI

QCSSQASFSFGFIFSRFIREGLWLPVDWIRSLGFTDVFAPEPLAQDKCYDKRAFYPSFPQ

RFLIVKNKHLTIYFFLSLIRLFVHRFTVPSWIPGITIKENSHGDEFPVMRQLHLLKRHDV

EEVLERSEDFSVIYGPRMCDVTQNGRSSGNFLLGMQVSSSTYTRDISNMRLIFRRDDVDR

CQMIAANASQDSFFVFEERKDVKRDLDRGKVLSLPNDLVQPVIKSFVEHYFGISLPMQAQ

NANGTTSTDFNLTWFADLFYYIFYDINGDDSREAALASADLLNQDLDRQIAAAKEPSMND

LENVDTVLHRCLKLQRSDTPGMDDLSIRINLTGFLVGAVLPLQNTICQVIDQLLARPHIL

QQAVTAADHNEWEMLQGFVLEALRFSPGDPVIYRHCNTKTELVSASYRSPVSPNTLVMAW

NSSAMFDPKYVDQPWQFNPKRQTRDYLHFGHMHHVCAGKYIVMSVIPAVVRELLSCYELA

RIPGRCGYPVKKGITVSEFDVLVRLRDSDDSLRDKFP

>CYP213A15(AII49204.1)Synechococcus sp. KORDI-52

MTTSTLPSTGAVTGLGETLAFFTRADFAQQRFKTYGDVFETKLLGQRMVFIQGEQAISDL

FRQSDVLEGWWPESVKQLLGSKSLANRTGPGHKARRRVVGQLFSSAALARYRPSIEGLVD

ELILELVTSDAALPLAPRMRRFAFSVIATTVLGLDGASRDALFNDFEVWTKALFSVPLAI

PGTPFARAMAARQRLLERIKTELQRADKAGGLDLLSGGLDEAGIPLDDDDLAEQLLLLLF

AGYETTASSLSCLFRALLLHPEVETWVREGLAESTTSERLDATVLEVMRLTPPVGGFYRR

CLEPISLADVALPQGSVVQVVLRSAAPEDDADLAAFRPQRHLDGTFGQTLLPFGGGERVC

LGKALAELEIRLMATGLLQSVELQLEPEQDLGLQRLPSPTPKDGLLVQATAR

>CYP110L2(AII49737.1)Synechococcus sp. KORDI-52

MRTDVKQIPELHVPPYRQVLEAILRPIAYYRRCFRASSGVIRVRMSPTLPPRQVLISDPA

VIKDLINHDGGPSISAPGELNGLLSQVVGQHSIILLSPAMHRQRRKLLTPPFHGERLKAY

GSLITALAEQVMADLSPGDVFDARSRMQRITMRVILTAVFGLHDGESMRQVEQTLNASLG

IRSGRLGSLLLFFPFLRRDLGSWSPGGRIKAAEAAVRRVLLGQITARRRALECSRADDQP

VDILSLLLACTDEQGQGLSDDELHDELFTLLFAGHETTATALTWALHWLHRRPDVRRRLL

EELDGLPDPDDPEAITRLPYLSAVVSEVLRIHPVAMLMFPRRVEQAVTMGGFVFHPGDVL

LVCIQALHERSDLYPDPLRFNPDRFVGRSYGSHEFLSFGAGSRRCIGAALAAYEMKLILV

SLLRGANYRLTSPSDRDNKPRRRGFTLGPSNPVRLQVVAMRR

>CYP213A16(AII43834.1)Synechococcus sp. KORDI-100

MLEAVGFFRDPEFASRRFEAFGDVFETTLIGQRLIFVRGDQAIEDLFQQSDAVQGWWPGS

VRTLLGSRSLANRNGPDHKARRRVVGQLFTTAALRRYSPKIMAMVNSLAEEVKAADTPIA

LAERMRRFAFSVIATTVLGLDGRDRDDLFRDFEIWTKALFSVPLAIPGSPFAKALKARSR

LLSKLQQVLSEADDRRGGLDLLAGGLDEAGLPLSDEDLVEQLLLLLFAGYETTASSLSCL

MRELLLQPELLIWLQEELDHLVWPHTDDPTSSYDSARAPRLQAVVNEVMRMTPPVGGFFR

ITTRPIALAGVEIPAGRVIQVALASSNRHGSGDLERFRPQRHLDGSFRKTLLPFGGGERV

CLGKALAELEIRLMTVGLLKQVSISLCTDQNLDLQLIPSPSPKDGLLVNARH

>CYP110AH1(AFY74897.1)Synechococcus sp. PCC 7502

MTIPPTINSPKVLQLWKWIVEPLDYLHKFDRECGDIFTVNMSSVFNGAVFVSHPQAIQQV

LTSDTKQFSAPGSINQILKPFLGDRGVILLDGREHRQRRQLLMPQFHGDKVRNYTNAIQQ

ITRNLIAGWQVGEKLNVRKEMEKITLSVILQTVFGLDRDKRYDQIREKLSKMLSLIESPI

NAAFLFLPFMQKDLGAWSPWRRFVRDRAELDRLIYDEISDHRQNSNEERSDILSMLISSQ

DSDGNRMSDLELHDELMTLLFAGHETTATALAWAIYWINYLPEVKQKLLTEIATLGTEPD

AISLSKLPYLNAVCAETLRIYPVGMLTFPRITNESISIRGYEIKPNTVVMGCIYLTHHRE

DTYPDPHLFKPERFLERQFSPYEYLPFGGGSRRCVGMALAQLELKLVIVEILSHCQLQLI

GKLPIMPVRRGVTLAPNGGVSARVKSKYV

>CYP213A15(KR52_08615)Synechococcus sp. PCC 7502

MTTSTLPSTGAVTGLGETLAFFTRADFAQQRFKTYGDVFETKLLGQRMVFIQGEQAISDL

FRQSDVLEGWWPESVKQLLGSKSLANRTGPGHKARRRVVGQLFSSAALARYRPSIEGLVD

ELILELVTSDAALPLAPRMRRFAFSVIATTVLGLDGASRDALFNDFEVWTKALFSVPLAI

PGTPFARAMAARQRLLERIKTELQRADKAGGLDLLSGGLDEAGIPLDDDDLAEQLLLLLF

AGYETTASSLSCLFRALLLHPEVETWVREGLAESTTSERLDATVLEVMRLTPPVGGFYRR

CLEPISLADVALPQGSVVQVVLRSAAPEDDADLAAFRPQRHLDGTFGQTLLPFGGGERVC

LGKALAELEIRLMATGLLQSVELQLEPEQDLGLQRLPSPTPKDGLLVQATAR

>CYP110L2(KR52_11385)Synechococcus sp. PCC 7502

MRTDVKQIPELHVPPYRQVLEAILRPIAYYRRCFRASSGVIRVRMSPTLPPRQVLISDPA

VIKDLINHDGGPSISAPGELNGLLSQVVGQHSIILLSPAMHRQRRKLLTPPFHGERLKAY

GSLITALAEQVMADLSPGDVFDARSRMQRITMRVILTAVFGLHDGESMRQVEQTLNASLG

IRSGRLGSLLLFFPFLRRDLGSWSPGGRIKAAEAAVRRVLLGQITARRRALECSRADDQP

VDILSLLLACTDEQGQGLSDDELHDELFTLLFAGHETTATALTWALHWLHRRPDVRRRLL

EELDGLPDPDDPEAITRLPYLSAVVSEVLRIHPVAMLMFPRRVEQAVTMGGFVFHPGDVL

LVCIQALHERSDLYPDPLRFNPDRFVGRSYGSHEFLSFGAGSRRCIGAALAAYEMKLILV

SLLRGANYRLTSPSDRDNKPRRRGFTLGPSNPVRLQVVAMRR

>CYP110AF2(AMA08013.1)Synechococcus sp. PCC 73109

MAIATEFKSIPRLSTGKFQQKLQSLLNPVGYLTRLASLAPDLAYMPAAGYDQPLILAYHP

EAIRQLLSNSNQAFAAPGELNGIIEPLVGQQSLVSLSGDRHRQERKLIMPAFHGARMHNY

GALITQIIEEELSQLKPGQAFQMQDITQKITLRTIIEVVFGVQGGDRYAPIMAMTRKILN

RFTSPIANSFLFFTSLQKDLGAWSPWGSFIRDRQALDELIYAELQERRAHPDPERTDILS

LLMAATDEAGNTMDDQQLRDELMLLLFAGHETTAIAMAWGTYWLHTHPEVMAKVRAEITA

LGENPDPMDIYRLPYLAAVCNETLRINPVAMFTFARQAVTITQLLDYSIEGGTVLMGSIY

LLHQRPDLYPNPREFRPERFLERQFTPYEFMPFGGGVRRCAGEALAMVELRLGLAAIATQ

ADLTLLDQVPVAAKRKGLVLSPATGVKMRFNGSKIYYSN

>CYP120A6(AWQ23_15110)Synechococcus sp. PCC 73109

MTSPSKLPLPPGKFGLPVIGETIEFFTDRNFQKKRLEKYGDVFKTNIFTKPTVVMVGAEA

NEVLFRNENKYVKATWPKSTRILLGADSLATQEGGIHSSRRRIIFQAFQPRALESYIPTI

EKITQRYLTEWEQKQEFAWYDELRKYTFDVASTLFIGKDGGAETPLANLFEEWVKGLFSL

PINLPWTAFGKAMKCRRKLLRELEMIIGDRLKTYDANAEPTDALDLLIRAKDEDGNALSI

DELKDQILLLLFAGHETLTSSLVSFGLLVGQHRDVFEKIRTEQDALDIGNGLDMATLQQM

TYLDQVFKEVLRLVPPVGGGFREVINTFEYKNFQIPQGWAVQYQIAQTHKDEALYPDHEK

FDPERFSPERLADKQKKFGFIPFGGGMRECIGKEFARLEAKILASMLARDYDWELLPDQD

LSMQVIPTPLPKDGLQIRFYRRKESSSTTA

>CYP110AF2(ACA98184.1)Synechococcus sp. PCC7002

MAIATEFKSIPRLSTGKFQQKLQLLLNPVGYLTRLASSAPDLAYMPAAGYDQPLVLAYHP

EAIRQLLSNSNQAFAAPGELNGIIEPLVGQQSLVSLSGDRHRQERKLIMPAFHGARMHNY

GALITQIIEEELSQLKPGQAFQMQDITQKITLRTIIEVVFGVQGGDRYAPIMAMTRKILN

RFTSPIANSFLFFTSLQKDLGAWSPWGSFIRDRQALDELIYAELQERRAHPDPERTDILS

LLMAATDEAGNTMDDQQLRDELMLLLFAGHETTAIAMAWGTYWLHTHPEVMAKVRAEITA

LGENPDPMDIYRLPYLAAVCNETLRINPVAMFTFARQAVATTQLLDYSIEGGTVLMGSIY

LLHQRPDLYPNPREFRPERFLERQFTPYEFMPFGGGVRRCAGEALAMVELRLGLAAIATQ

TDLTLLDQVPVAAKRKGLVLSPATGVKMRFNGSKT

>CYP120A6(SYNPCC7002_G0105)Synechococcus sp. PCC7002

MTSPSELPLPPGKFGLPVIGETIEFFTDRNFQKKRLEKYGDVFKTSIFTKPTVVMVGAEA

NEVLFRNENKYVKATWPKSTRILLGADSLATQEGGIHSSRRRIIFQAFQPRALESYIPTI

EKITQRYLTQWEQKQEFAWYDELRKYTFDVASTLFIGKDGGAETPLANLFEEWVKGLFSL

PINLPWTAFGKAMKCRRKLLRELEMIIGDRLKTYDANAEPTDALDLLIRAKDEDGNSLSI

DELKDQILLLLFAGHETLTSSLVSFGLLVGQHRDVFEKIRAEQDALDIGNGLDMAVLQQM

TYLDQVFKEVLRLVPPVGGGFREVINTFEYKNFQIPQGWAVQYQIAQTHKDEALYPDHEK

FDPERFSPERLADKQKKFGFIPFGGGMRECIGKEFARLEAKILASMLARDYDWELLPDQD

LSMQVIPTPLPKDGLQIRFYRRQESSSTTA

>CYP213A5(CAK28009.1)Synechococcus sp. RCC307

MSASAEQAESITCSLARSGSMPAKPLPNTGALSGLKETLDFFGDPGFAQRRFETYGDVFA

TKLLAQPIVFIRGERAINDLFSQSDSLEGWWPESVKKLLGSRSLANRSGAGHKARRRVVG

QLFSSAALARYTPSIIGLVDELADELINADGPVSLAGRMRRFAFAVIATTVLGLDSESRE

ALFADFEIWTKALFSIPLAIPGTPFAKAMGARQRLLNRIKAVLQEGSNQGGLDLISGGLD

EAGIPLDDDDLAEQLLLLLFAGYETTASSLSCLFRALLLHPEVMEWLSSDVMASPWPAAT

SPQSEKLDATVLEVMRQTPPVGGFFRRSKQAIELADVAVPENSVIQVALTPTAATSGTDL

SEFRPQRHLDGSFEQTLLPFGGGERVCLGKALAELEIRLMAVGLLQKVQLQLVPDQDLAL

QLVPSPTPRSGLMVTATER

>CYP213A1(CRY92309.1)Synechococcus sp. WH 8103

MTGLGETLAFFRDPSFSQRRFSELGDVFETKLLAQSIVFIRGERAIGDLLKQEDCLQGWW

PDSVRQLLGSKSLANRSGADHKARRRVVGQLFSSAALSRYTPAIEALVNDLANELQQAEG

PIPLAARMRRFAFSVIATTVLGLEAENRDALFADFEIWTRALFSIPLALPGTPFARALAA

RQRLLARLKTVLQTNNNRQQGGLDLLSGGLDEAGLPLDDDDLVEQLLLLLFAGYETTASS

LSCLFRALLLNPEVEQWLMQDLNNHERPSRLDATVLEVMRMTPPVGGFFRQNTQSIELAD

VAIPQGRVIQVVLSSSSTTNQTDLETFRPQRHLDGSFQQTLLPFGGGERVCLGKALAELE

IRLMAMGLLQRVQLHLEPDQDLNLQLIPSPTPRDGLLVRATAR

>CYP213A13(AHF63490.1)Synechococcus sp. WH 8109

MATPTLPSTGAVSGLGETLAFFTQADFAQRRFETHGDVFETKLLAQRMVFIRGERAIGDL

LGQGDALQVWWPESVRQLLGSQSLANRSGPGHKARRRVVGQLFSSAALARYTPSIEQLVV

ELCQELITTNTPLPLAARMRRFAFAVIATTVLGLDGASRDSLFADFEIWTRALFSIPLAI

PGTPFAKAMAARQRLLKRIKGVLQAGTNQGGLDLLSGGLDEAGIPLDDDDLAEQLLLLLF

AGYETTASSLSCLFRALLLNPEVERWLRDGLASEAASPRLDATVLEVMRLTPPVGGFFRR

SLAPIELAGLTIPEGSVIQVVLSPTSASDDDDLAAFRPQRHLDGSFEQTLLPFGGGERVC

LGKALAELEIHLMAAGLLLAVELQLQPDQDLALQLIPSPTPKDGLLVQASAR

>CYP213A6(CAK23432.1)Synechococcus sp. WH7803

MVTFVLPCVMAAKTLPSTGAVTGIKETLDFFGDPQFAQKRFKAHGDVFETRLLNQRLVFI

RGEQTMADLFAQGDALEGWWPESVRQLLGSRSLANRSGDGHKARRRVVGQLFSAAALSRY

TPSIAALVEELADELIQAKAPVQLVPCMRRFAFAVIATTVLGLDASDRQALFADFEIWTR

ALFSIPVAIPGTPFAKALEAKKRLLGRLKQVVAQTSAERGGLDLLSGGLDEAGIPLTDDD

LVEQLLLLLFAGYETTASSLSCLMRALLLNPEIEQWLLPELLDQPWPNLESRSCPRLDAT

VLEVMRLTPPVGGFFRRTKQTIQLAGVAVPPDRVIQVALAPDLGDGSCDLAEIRPERHLD

GSFTQTLLPFGGGGRVCLGKALAELEIRLMAIGLLQRVHLGLTPNQDLTLQQIPSPSPRG

GLLVTASARHKS

>CYP213A1(CAE07923.1)Synechococcus sp. WH8102

MTAAPLPSTGAVTGLGETLAFFRDPSFSQRRFSELGDVFETKLLAQSIVFIRGERAIGDL

LKQEDCLQGWWPDSVRQLLGSKSLANRSGADHKARRRVVGQLFSSAALSRYTPAIEALVN

DLANELQQAEGPIPLAARMRRFAFSVIATTVLGLEAENRDALFADFEIWTRALFSIPLAL

PGTPFARALAARQRLLARLKTVLQTNNNRQQGGLDLLSGGLDEAGLPLDDDDLVEQLLLL

LFAGYETTASSLSCLFRALLLNPEVEQWLMQDLNNHERPSRLDATVLEVMRMTPPVGGFF

RQNTQSIELADVAIPQGRVIQVVLSSSSTTNQIDLETFRPQRHLDGSFQQTLLPFGGGER

VCLGKALAELEIRLMAMGLLQRVQLHLEPDQDLNLQLIPSPTPRDGLLVRATAR

>CYP120A1(AVP90553.1)Synechocystis sp. IPPAS B-1465

MITSPTNLNSLPIPPGDFGLPWLGETLNFLNDGDFGKKRQQQFGPIFKTRLFGKNVIFIS

GALANRFLFTKEQETFQATWPLSTRILLGPNALATQMGEIHRSRRKILYQAFLPRTLDSY

LPKMDGIVQGYLEQWGKANEVIWYPQLRRMTFDVAATLFMGEKVSQNPQLFPWFETYIQG

LFSLPIPLPNTLFGKSQRARALLLAELEKIIKARQQQPPSEEDALGILLAARDDNNQPLS

LPELKDQILLLLFAGHETLTSALSSFCLLLGQHSDIRERVRQEQNKLQLSQELTAETLKK

MPYLDQVLQEVLRLIPPVGGGFRELIQDCQFQGFHFPKGWLVSYQISQTHADPDLYPDPE

KFDPERFTPDGSATHNPPFAHVPFGGGLRECLGKEFARLEMKLFATRLIQQFDWTLLPGQ

NLELVVTPSPRPKDNLRVKLHSLM

>CYP120A1(BAL30280.1)Synechocystis sp. PCC 6803 GT-I

MITSPTNLNSLPIPPGDFGLPWLGETLNFLNDGDFGKKRQQQFGPIFKTRLFGKNVIFIS

GALANRFLFTKEQETFQATWPLSTRILLGPNALATQMGEIHRSRRKILYQAFLPRTLDSY

LPKMDGIVQGYLEQWGKANEVIWYPQLRRMTFDVAATLFMGEKVSQNPQLFPWFETYIQG

LFSLPIPLPNTLFGKSQRARALLLAELEKIIKARQQQPPSEEDALGILLAARDDNNQPLS

LPELKDQILLLLFAGHETLTSALSSFCLLLGQHSDIRERVRQEQNKLQLSQELTAETLKK

MPYLDQVLQEVLRLIPPVGGGFRELIQDCQFQGFHFPKGWLVSYQISQTHADPDLYPDPE

KFDPERFTPDGSATHNPPFAHVPFGGGLRECLGKEFARLEMKLFATRLIQQFDWTLLPGQ

NLELVVTPSPRPKDNLRVKLHSLM

>CYP120A1(BAK51282.1)Synechocystis sp. PCC 6803 GT-S

MITSPTNLNSLPIPPGDFGLPWLGETLNFLNDGDFGKKRQQQFGPIFKTRLFGKNVIFIS

GALANRFLFTKEQETFQATWPLSTRILLGPNALATQMGEIHRSRRKILYQAFLPRTLDSY

LPKMDGIVQGYLEQWGKANEVIWYPQLRRMTFDVAATLFMGEKVSQNPQLFPWFETYIQG

LFSLPIPLPNTLFGKSQRARALLLAELEKIIKARQQQPPSEEDALGILLAARDDNNQPLS

LPELKDQILLLLFAGHETLTSALSSFCLLLGQHSDIRERVRQEQNKLQLSQELTAETLKK

MPYLDQVLQEVLRLIPPVGGGFRELIQDCQFQGFHFPKGWLVSYQISQTHADPDLYPDPE

KFDPERFTPDGSATHNPPFAHVPFGGGLRECLGKEFARLEMKLFATRLIQQFDWTLLPGQ

NLELVVTPSPRPKDNLRVKLHSLM

>CYP120A1(BAL33449.1)Synechocystis sp. PCC 6803 PCC-N

MITSPTNLNSLPIPPGDFGLPWLGETLNFLNDGDFGKKRQQQFGPIFKTRLFGKNVIFIS

GALANRFLFTKEQETFQATWPLSTRILLGPNALATQMGEIHRSRRKILYQAFLPRTLDSY

LPKMDGIVQGYLEQWGKANEVIWYPQLRRMTFDVAATLFMGEKVSQNPQLFPWFETYIQG

LFSLPIPLPNTLFGKSQRARALLLAELEKIIKARQQQPPSEEDALGILLAARDDNNQPLS

LPELKDQILLLLFAGHETLTSALSSFCLLLGQHSDIRERVRQEQNKLQLSQELTAETLKK

MPYLDQVLQEVLRLIPPVGGGFRELIQDCQFQGFHFPKGWLVSYQISQTHADPDLYPDPE

KFDPERFTPDGSATHNPPFAHVPFGGGLRECLGKEFARLEMKLFATRLIQQFDWTLLPGQ

NLELVVTPSPRPKDNLRVKLHSLM

>CYP120A1(BAL36618.1)Synechocystis sp. PCC 6803 PCC-P

MITSPTNLNSLPIPPGDFGLPWLGETLNFLNDGDFGKKRQQQFGPIFKTRLFGKNVIFIS

GALANRFLFTKEQETFQATWPLSTRILLGPNALATQMGEIHRSRRKILYQAFLPRTLDSY

LPKMDGIVQGYLEQWGKANEVIWYPQLRRMTFDVAATLFMGEKVSQNPQLFPWFETYIQG

LFSLPIPLPNTLFGKSQRARALLLAELEKIIKARQQQPPSEEDALGILLAARDDNNQPLS

LPELKDQILLLLFAGHETLTSALSSFCLLLGQHSDIRERVRQEQNKLQLSQELTAETLKK

MPYLDQVLQEVLRLIPPVGGGFRELIQDCQFQGFHFPKGWLVSYQISQTHADPDLYPDPE

KFDPERFTPDGSATHNPPFAHVPFGGGLRECLGKEFARLEMKLFATRLIQQFDWTLLPGQ

NLELVVTPSPRPKDNLRVKLHSLM

>CYP120A1(AGF52788.1)Synechocystis sp. PCC 6803(syn)

MITSPTNLNSLPIPPGDFGLPWLGETLNFLNDGDFGKKRQQQFGPIFKTRLFGKNVIFIS

GALANRFLFTKEQETFQATWPLSTRILLGPNALATQMGEIHRSRRKILYQAFLPRTLDSY

LPKMDGIVQGYLEQWGKANEVIWYPQLRRMTFDVAATLFMGEKVSQNPQLFPWFETYIQG

LFSLPIPLPNTLFGKSQRARALLLAELEKIIKARQQQPPSEEDALGILLAARDDNNQPLS

LPELKDQILLLLFAGHETLTSALSSFCLLLGQHSDIRERVRQEQNKLQLSQELTAETLKK

MPYLDQVLQEVLRLIPPVGGGFRELIQDCQFQGFHFPKGWLVSYQISQTHADPDLYPDPE

KFDPERFTPDGSATHNPPFAHVPFGGGLRECLGKEFARLEMKLFATRLIQQFDWTLLPGQ

NLELVVTPSPRPKDNLRVKLHSLM

>CYP120A1(BAA10496.1)Synechocystis sp. PCC 6803

MITSPTNLNSLPIPPGDFGLPWLGETLNFLNDGDFGKKRQQQFGPIFKTRLFGKNVIFIS

GALANRFLFTKEQETFQATWPLSTRILLGPNALATQMGEIHRSRRKILYQAFLPRTLDSY

LPKMDGIVQGYLEQWGKANEVIWYPQLRRMTFDVAATLFMGEKVSQNPQLFPWFETYIQG

LFSLPIPLPNTLFGKSQRARALLLAELEKIIKARQQQPPSEEDALGILLAARDDNNQPLS

LPELKDQILLLLFAGHETLTSALSSFCLLLGQHSDIRERVRQEQNKLQLSQELTAETLKK

MPYLDQVLQEVLRLIPPVGGGFRELIQDCQFQGFHFPKGWLVSYQISQTHADPDLYPDPE

KFDPERFTPDGSATHNPPFAHVPFGGGLRECLGKEFARLEMKLFATRLIQQFDWTLLPGQ

NLELVVTPSPRPKDNLRVKLHSLM

>CYP110E3(ABG49672.1)Trichodesmium erythraeum

MIKLPGPKSPALTQILQWTAKPIKFMEKCAREYGDTFEVKLNYPIVFISHPKAIEEIFKA

NPKKFDCGSSNKLAQPLLGDYSLLLLDDIPHQRQRKLLMPPFHGKRMQAYGELICNVAQE

VASKWEIGQVFSMREFTAEISLKVILQAVFGLYEGERYSKLEKLLGSLLESLSSPLKTSM

LFFQFLQIDLGPWSPWGNFIKNREEIYELLCAEISERRQKLDPERSDILTMLLLARDEEG

EGMSDIELRDELMTLLIAGHETTATSLSWAFYWIHHQPEIYQKLSRELETFGDDLNPMTV

INLPYMNAVCSETLRIYPVVIIVSPRKTKLPITIMGQTYPAGTALTPCIYLTHHREDLYP

EPKKFKPERFLERQYSSYEFLPFGGGNRRCIGAAFAMFEMKIVLATILRRYSLALAEKQE

VKPLRRGLTLAPAGGVKMVMTGKLMQTEKKFPSVATV

>CYP214A1(ABG51955.1)Trichodesmium erythraeum

MNMNFKSSNPASSLALPPGDLGLPFIGQNKKIFKNPQNFIEEVYQKYGPVYKTNFLGKNF

IYFQGYEAIKFILTNENKYFTYSQILRNYQRIFGENDITVLAGKEHRERQKILAKTIKSK

NLNNYIDIIHDLSQSYFLKWIKSDYVDLYSEINNYTLDMILKLLLGIDYASKSEISNYLK

DMSSGLNTIPVVFPWTKFGSALESKNKLFNQFEQIIVRRKKENNFGSDILGILLTVQEQM

NYELTPREIVGQMVNLLSLGKKELSSALSSFFILTSEHLDVLKLLQIEQEKMDVSEPLSL

DKYKKMVYLEQVIKEVLRLVPPVSGGLRKIIEDCSFQGFRIPKGWHAYYYISSVLKDPEI

YKQPEIFNPERFNPTNAEDKKKPLCYIPFGGGARECIGKEFAYLVIKIFISALLDNCSWK

FKENQDLTINTFPVARPAHKIEVCFTPK

>CYP110G1(ABG52252.1)Trichodesmium erythraeum

MKQVCALKTPLWLQRFNYITNPVSYWQKAYSSYKDAFYAQGINFGKPLMVFYTPSAAKQI

IENCQGDLTTTSFDSELTAIFGDSSFFILEGTNHKKMRKLLIPALHGKHIKTYGELICNL

VNNLIENLPFNQSFSALEIAQEISMQVMIKLLFGNYQQERYQKIKQLMINMVSLFAANVF

GFPLFFKFLQQDLGLVSPWGNFLQQRRKIQQLIYQEIAERRNHPNQERTDILSLLMTAQD

EKGNFLNDEELLGQLLSLLFTGNESTAASIAWSWYEVYRNSKIKEKLLEEINNLGDSPEP

LSLFNLPYLSAVCNETLRKYPVTMFMIPRIVKNTTEINGYQLDKGMLVTVGTYILHHRED

IYDQPEEFKPERFIEHRFSSFEFLPFGRGMRGCIGADIALYQMKLTLATIISHHRLELTN

YGQIFPKRRNTILTPIKLRIIKAC

>CYP110D3(ABG52754.1)Trichodesmium erythraeum

MTLPDGPSLSPLQRRLRTWKFIFSPLSAIEERYSEYGDIFRTNTNSLYPFIYFCNPKAIQ

QIFTADPDTFTSGSINGILKYFVGLNSLLLQDGDRHKRQRKLLMPPFHGDRMRKYGDLIY

NITSNVISQWKIEQPFPIRKSTQEISLKVILAAVFGLDQEGKSYEKLRVLMSDLLDSMSS

PLSSTFLFFNFLRKDWGPWSPWGRFLRKKQELHELIIAEIQTAKKEGNHRDDILSLLLEA

RDEAGNAMSDEEIKDELLTMLFAGHETTASALAWALYWIDMIPSVGEKLMAELATIPSNS

DQVAITKLPYLSAICQETLRIYPIAMNAFPRVVQKPIEIMGYQLEPGMVAIVPIYLTHHR

EDIYPEPKKFKPERFLERQFSPYEYLPFGGGSRRCIGSAFALFEMKLVLATILSQWELKL

LPNQRISPVRRGLTMAPPANMRMVVKPKKSWQKVSQPILTSG

>CYP120A2(ABG53950.1)Trichodesmium erythraeum

MANYAPAIETMTANYLEKWVEMGTLTWYPEIRNYTFDIASLLFMGSDESSQTKLVSLFEE

WVKGLFSIPLSLPWTRFGKSLRCRQKLLQHIEEIILQRQQQQNLGEDALGILLQAQDKEV

NGLSLDELKDQILLLLFAGHETLTSAIASFCLLTSQHLDVLTRLRQEQKQFSAIEPLTLE

NLKRMTYLDMVLKEVLRLIPPVGGGFRQVTQDCEFCGYSIPKGWLVQYQIAKTHQDETLY

PDDKNFDPERFAPENAVDKQKVFGYVPFGGGMRECLGKEFARLEMKIFAVMLLRGYEWEL

LPEQDLSVVAAPTPYPRDGLKVKFRKVE

>CYP110C3(ABA21603.1)Trichormus variabilis

MKYQIKRPNPLKTHPFLQKLQWIADPVEYMEKASLQHRDMFTAEVIGFGDTVVFVSHPQG

IQTIFANDRKKLVAVGEANRILYPLVGNNSMFLLEGVKHKQRRQLLMPSFHGERMREYGH

LIRNITETLFSQLQQNVTFSALTAMREISMQVILQAVFGFYEGERCQQFKHLLPVFLSEL

FQSPLASSILFFPFLQKDLGNLTPWGRFVRQREKIDKLLYEEIAERRQEINSDRIDILSL

LISSRDETGNSMSDQELRDELITLMISGHETTGTAMAWSLYWILQTPEVFQRLIQELDSL

GDSPDPMSIFRLPYLTAVCNETLRINPVAMLTLPRVVKEPVELLGNRLESGTTVVGCIYL

THHREDLYPESKLFQPERFLKREFSQYEFMPFGGGVRGCIGQAIAMFEMKIVLATVLSRY

QFALADGKPERPQRQGFTLTPANGVKMLITGKHQRQNYSTAASTTFTT

>CYP110E6(ABA21725.1)Trichormus variabilis

MKLPDSPKIPRFMQLVQWIYQPLQLMEASAKAHGDCFTLWLTNKRPIVFLSNPQAIQELF

TTPLEQLDARGTAQVLQPLLGENSLLLLSGETHQRQRKLLTPPFHGDRMRAYGDIITNIT

QEVISKWQLGEPFSVRDSMQEITLRVILQAVFGLREGERYTQLQKRLCDILDLSGSALRS

TLSFLPALQIDLGSWSPWGHFLRQRAAIDQLLYAEIQDRRDHPDPSRTDILSLMMAARDE

NGEAMTDIELRDELMTLLVAGHETTASALTWALYWIHKLPQVREKLLAELDNFGDNGDVN

EITRLPYLTAVCQETLRIYPIAMVTIPRIVKTTLEIGGHQFAPGTMLVGCIYLMHRRPDL

YPQPQEFKPERFLEKQYSLYEYLPFGGSNRRCVGMAFALYEMKLVLATVLANMDLALVDN

YPVKPTRRGVTLAPSGGKWLIATGQHQKVKSPVEV

>CYP110A2(ABA23526.1)Trichormus variabilis

MLTQLPNPISVPSWWQLINWIADPIGFQKKYSQKYGDIFSMQLAGIGSFVILGEPQAIQE

IFTQDSRFDIGRGNKLAEPLIGRTSLMLMDGDRHRRERKLLMPPFHGEKLQAYAQQICLI

THQIASQWQIGQPFVARSAMQKLSLEVIIQIVFGLANGERYQQIKPLFTDWLNMTDSPLR

SSMLFLKSLQQDWGNRSPWGQMKYQQRCIYDLLQAEIEEKRTKENERRGDVLSLMMAARD

ENGQAMTDEELKDELLTILFAGHETTATTIAWAFYQIFRNVNVREKLQQELDSLGENPNP

MEIAQLPYLTAVCQETLRMYPVLPTLFPRITKSSINIAGYQLEPNTTLMASIYLIHYRED

LYPHPQQFRPERFIERQYSPSEYIPFGGGSRRCLGYALALLEIKLVIATVLSNYQLALAE

DKPIKVQRRGFTLAPEGGVRMIMTGKKSLRFEQSNKIFN

>CYP284A3(ABA23667.1)Trichormus variabilis

MLQYVTAQMNNSSSFPYLVTIFSFTTIAGTFAWRWWKQKKKYKSLQSLPSPPQHWLLGNL

PQVLAAVKQKKLFQLFFDWSQQLGPMYVVWNGSSPVVILSKPKVIEDTIVNGMRDGSLIR

SARLRQAWNDISGPILIGETGNEWQWRRKVWNPEFSSSSLAKYLKIIHQACEQVIDTLKE

TAPPKEVEVDPLFVELTMRVISSLVLGIPVDRTITTNEGPPLEVLKVYEAMCVVGYRFLR

QATGEKIWMKYLPTKNSQDYWASRRYLEEFLTPRVDLALQMREQKTDFPQVSPLFRESML

VRIAAKEPKYNRQTLIAESVEFLIAGTDTTAHTLSFAVGELSLNPRVFQKARDIVDQAWQ

SQDNINTESFKELAYISAILKETLRLYSVASGSTSLEAQRDTVIEGKVIPSGTRISWSML

AAGRDPEVYAHPEEFLPERWLDKSKETSSLPMIDFGSGPHRCLGEHLSMLEGTMMLALLL

RHFDWELVNGRSSLEQLQQNLLIYPSDKMPVRFRLRN

>CYP110C27(AVQ74175.1)Microcystis sp. MC19

MKILPKVKAPTFLQMAQWIINPVAFMENAARKHGDIFSTKVGLTVDNFIFVSSPSALQQI

LTNDRKQFSAPGEANRIIAPIIGDYSVVMLDGDIHKKRRQLLLPPFHGERMRFYGDLIRD

ITLRVMAELPENQPFKARSATTAIALQVIMEAVFGISQGERYQTLKKILAEMLDIFNSPV

MASLLFFPILRADFGAWSPWGKYQRFQEKIDDIIYTEIAERKANPNPNRTDVLSLLMSAQ

DEEGNPMSDKELRDELMTLLFAGHETTATAMSWVLYWTHRYPEIKAKILQEIATLGDNPN

PIDITRLPYLSAVCSETLRIHPVGMLTFPRVVQEPVELDGYPLEKGTILMGCIYLAHHRE

QTFPDSHTFKPERFLEKQFTPYEYMPFGGGARRCIGEVLAIYEMKIAIATILANYQLTLV

NNTPEKPSRRGVTLAPSRGVPMVLKGRREPLVTAPMLAEIS

>CYP120A24(AVQ74246.1)Microcystis sp. MC19

MTISKDLPLPPGSFGLPLLGETIAFLTDGDFASKRHNKYGQLFRTHIFGSPTIILSGAEA

NRFLLSNENKYFAATWPKSTKTLLGSASLAVHTGDVHASRRRLIYQAFQPRSLASYIPTV

ETITAHYLERWQTATTLSWYPELRNYTLDIACKLFVGLDQGSATKLGEAFDTWCAGLFTL

PLPLPWTAFGKALRCREELLEAIETIILERQKNDDPGQDALAILLQAKDENGQSLSLAEL

KDQVLLLLFAGHETLTSAIATFCLQMALHPDIFQLVLEEITNFDLSTPLSVDTLKQMTYL

DRVLKEALRFTPPVGGGFRRVIEDCQFNGYHLPKGWVVQYQITNTHKDNNIYSHPETFDP

DRFLTEEKSYGYIPFGAGLRECIGKEFARLEMKILAVRLVEKYDWQLLPNQDLTLTSIPT

PHPRDGLQVTFKPR

>CYP110D5(EAM52765.1)Crocosphaera watsonii

MNLPPTLSQPRLLRLFKLIFYPLDYLEDNYQRYGDIFVAGKSETPFVYISNPQGIQTILT

RDKTDFKTGGGSGFLSTLLGDNSLLFLQGERHRRERKLLMPPFHGERLKSYANLIYSISD

KVTDKLQINRSFNVRDIMQEITLKVILKAVFGITEGERYQRLQELLKSWLSFFDSPANAI

LIFFPWLRKNWGNWTPWGRFLQIKAEIQELIYTEIRERREQKKYEGTDILTLLMLAKDEE

GKPLSDQELHDELITLLIAGHETTASALTWALYWIHFCPDVEDKLRFHFSNLNNNTDLLD

IVKLPYLDAVCKETLRIYPVLLTTFIRVLQTPLELMGYQFKPGTVFAPAIYLVHHREDIY

PNSQQFRPERFLERNFSPYEYFPFGGGSRRCIGMELAKMEMKIVLYTILSKHKLKLPSSR

PLKAVRRGLTVAPPSNFKMILSN

>CYP1011A1(EAM49187.1)Crocosphaera watsonii

MTKTKKNKTQNKLVFNPFYRAFHNNPYPIYERLRNEDPIHWSFLKAWIITRYQDVDTILK

DNLFQVDDLPLRLEEKSAYLKQGNFLPLAKTIDKWLFFQQPPNHTRLRSLVNKSFSPASV

GNMKEEIEAKVNHLLDKVIPTGKMDLIDDLASPLPAMTVTNILGLPPEDYYKLIHWSYEL

FFVFDQPMSLEGYEKQNKMAMEAREYLLRFIANIDENSQGLIADLVKAKDEENKLDEDEI

LGFCIMLLIVGQETTKSFISNSILALLQHPEKLQELKDNPEIIKEASEELLRYDTPVQVI

ARLAREDVEIGGKTILKGDKVILCLGGANRDENKFPNPEKIEFQRSNRNLPFGGGIHFCL

GAFLARLQGQISINRIVQRLPNLQLVNQTPDWRESITLRGLKSLPLTFDKNDIKTD

>CYP110C5(EAM47967.1)Crocosphaera watsonii

MKTIPTPKTPTLVQQLQWVLNPTGYLQTNHHRYPDLFKAKIIGLGNDIILISNPEIMQYI

LTHDRQEFTAPSSLNTLLKPLLGDYSVVMLDGDGHRQRRQLVMPSFHGERLKVYGDLTCR

ITREAMEKLPENQPFLAREVMQDISLKVIMEAVFGVTEGERYEELQYRLKELLDLFDSPI

TSGFLFFPSLQKDLGNWSPWGYFLRQRQALDKLIYAEISDRRANPDPERTDILSLLMFAK

DEQGESMKDQELRDELITLLMAGHETTASAMAWALYWLHHIPEIKDKLIEELNTLSPDAE

GMDIFRLPYLTAVCNETLRLSPSAMLTFTRLAQQTVEVGGYTFKPGDIVAGCLYLTHLRE

DIYANPKQFNPQRFLDHKYSAYEFIPFGGGSRRCMGEALAKFEMKLVIAIIISEYCLKLA

DTQPEKQQRRGLTLSPKRGVKMILEGKRQPQKARELELSTR

>CYP110D19(Cyan7822_0594) Gloeothece verrucosa

MTLPPRPTQPQMLRMLKLVFRPTDYMDENGKRFGDCFAVGRSETPFVYISNPKAIQQIMT

APAEQFDSGRGNGILKFFVGENSLILLDGVAHQRQRKLLTPPFHGERLQTYQQLICDIAA

QVTEPLQVGKPFRVRTIMQEITLRVILKAVFGLQEGERYHKLRQLLTTVLDSISSPLSSS

ILFFRQLRKDWGPLSPWGRFLRLKAQVDQLICEEIRERQQQEKLDGEDILTLLLSARDEN

GEPMTLLELRDELMTMLIAGHETTASILTWALYWIHYLPEVGDKLRFELSSVEDKSNLAH

ICKLPYLNAVCSETLRIYPVAPLTFPRILKSDMELLGYKFAANTVLAPCIYLLHHREDLY

PQPDQFKPERFLERQYSLYEYIPFGGGNRRCIGMALALLEMKLVLATLLQRFQLELLNSR

PLKPVRRGLTIAPPNNFKMVLKSVNYH

>CYP1136A1(Cyan7822_0990) Gloeothece verrucosa

MTNQLLNNFLLLEEGQTPPSETTLPPRQPQWFDTFSYIADPDQFCRQNLAKYGPIFKTGV

FGETTVFVGSAKVNQMAFNGDQHYTEIALPPTTMDMFGQYSLFQRTDLHRSRKSALSPAF

TGKMLEGYLPLINQVVLEGIQSWKTTDLLSVFPAVEKICFDVLVPLLLGVDLNQKDSLKG

LPISSKTELKALYKTFFDGFYGLVKWKSPLTVYGRGYQAREKLLDFMGSVIQQRRAQGEV

INSQADFLSMMLAGQEENPTGVFQDIFIKNQCLLQLWASHYEICGLVSSLIYQIGRHPEV

KKRLVQEQIEVMGEQTSDKMITSQQLKAMVFLEATIKETLRTLSPSSTVNRRLTKSVVLD

GVLYQKGWVLIAEQRIAHILPEHFKQPDVFDPERFLSPRNEGKMYEFIAFGGGVHACLGA

QLAMLITKVFACYLLQLLNWEVTQAASFVQFPLKRLKSNYQIPIHSRATA

>CYP120B15(Cyan7822_3321) Gloeothece verrucosa

MTQRTLDGQSYPLPPGNLGLPILGETLSFLQDRNFANKRHKKYGSVFKTHLFGRPTVILM

GPEANRFILSTHFDHFSWREGWPKMFRELLGRSLFLQDGEEHRRNRKLLMPAFHGPALNQ

YITTMEEIIDRYLNNWEKQGSIAWFFELKKMTFEIASILLIGSEPGELTDMLSQWFTELT

SGLFTLPIALPGTTYSKALKARDRLLNHIEKVVQERQKHPTSDALGLLVQTRDEEGNSLS

LEELKVQALLMLFAGHETTTSMLASFNMVLAQNRQIRARLNTEIENISPKGSITLEQLRQ

MTYLDQVLKEVERFYPPVAGGFRGVVKPCVFGGYYIPEGWQLLYRIDATHLDQRVYTNPE

QFDPDRFSPERAENKKMEYSLVGFGGGSRICLGYTFAQMEMKIFAVHLLRHYDWELLPDQ

DLSFHPISTINSGSGLLVKFYRYN

>CYP110AM2(Cyan7822_3646) Gloeothece verrucosa

MTSVISTQAAAKLPDGPKIPAIIQTILALMDQFGSLERNYQKYGDIFYTPKSSLFPAFVA

LSDPKAVEKVLTANPRLFEVGKQSSLAVRVLLGDNSLVLLDGIEHQKRRKLLMPPFHGER

MKSYGQTIVDVTKEVMAQWQEGKSFSIRDYTQQISLRVILRTVFGLDEGERYARLEKILT

AWLNIFNSPFNAFFLFFPVLQKDFGAWTPWGQFVEQKRLIHEILQSEIERRRHNPDSLGE

DILSLLLSVEDEEGQPMSDTEIKDELMTMLFAGHETTANTLAWAFYWIHYRPEIYQKLLA

ELNSLDENADFNAINKLPYLNAVVSETLRLYPVVPFLSRQLKEPFEIMGYQFEAGTALLP

CIYLIHQREDIYPQPKQFKPERFLEKQFSPYEYLPFGGGHRRCLGYAFALFEMKLVLATV

LSQVHLELPFQRPPKAIRRGITFAPSGGLKMRLKKQV

>CYP110C26(Cyan7822_4332) Gloeothece verrucosa

MTTVLPGSKTPGLLQQLHWAFDPVGYLESNQQKYPDLFLANVAGWKQPILFVSHPQALQY

ILTNDRKQLSAPKKTNEIVRPLVGDYSILLLEGQRHQQRRQLLAPPFHGERMYSYGESIC

QITHKVLSELPLNKPFSARQVTQSITMQVILEVVFGLYEGERYQQLKKLLSKMLDIFNSP

IASALLLFPALQLDLGTWSPWNKFLRQREQIDHLLYQEIAERRANPNPEATDILSLLMSV

RYEDGQPLSDQELRDELMTLLMAGHDTTATAMAWGLYWIHHLPHVKEKLLQELATLKEKP

DPMEIVRLPYLSAVCNETLRITPVAMLALPRVVQEPLEILGYALEPGTAIFGCMFLTHRR

SDLYPDPKQFKPERFLERKYSPYEFIPFGGGTRRCVGDALAPFELKLVLASIVSGYDLSL

ADTRPEKLQRRGLTLGPARGVLMILKGQRTLEKPSENLVTV

>CYP110A8(Cyan7822_4333) Gloeothece verrucosa MKKALPNLVNRPAWWLLAEWIIDPLNLLQKWAKKYPDLFTISLAGIGDEVIISNPEIIEE

IFNKDAKQFHIGRGNQIIAPLVGQNSLLLMDGERHKRERKLLMPSFHGERLQTYAQQICE

ITEAVASQWQEGKGFIARTAMQKITLEVITQIVFGLREGERYQKIKPLLAAMLNLTDSPL

RSSLFFIPFLQKDLGYWSPGGILKRYQLEIRELLQAEIEEKRHQKGEIGKDILSLMIAAR

DEQGEPMSNLELKDELLTLLFVGHETTATLLAWAFYQIHRLPQVRQKILQELDSLGENPN

PMQITQLPYLTAVCQETLRMYPVIPIVFSRITKTAMDIGNYHFEPQTILTPSIYLVHYRE

DLYPQPQEFKPERFLERQYSPSEYFPFGGGSRRCLGYALALLEMKLVIAKILSGYELALV

DNKPVKITRRGLTLAPSGGIPLLLKSKRHPQASSPEKTVTLVP

>CYP1158A1(Cyan7822_5890) Gloeothece verrucosa MTNLPCSPPLSPAQQAQKWIDQPLEFLDHCFQDYGDIFTLELGALGATIFLGNPQAVETI

FKLHGHLFECHQFNVSYAPLMGKNALFLQDGEAHKRLRRIMMPPFHRERVQKYADYIQEI

AQESVKDWTRGKVLKIRSLMHRLALEVTLRIFFSERTLPIEKIRYWFETKVFTETKSWKP

WFNYSRLQPQIRQLIREEIEFRKEAKFESVDLLNWLQLAQDEEGNFLSEEELQDQLLTLM

ITAVDPIAMSLTWALYWIHKLPKVKATLSEELKTLEGKLDPLAINQLPYLTAVCQETLRL

HPILPTVSGRRLTTPIEVMGYHFPEGTTVAPCAYLVHRREELYPEPRSFKPERFLQNQYS

AYEYFPFGGGNRLCLGAALAPLEIKIVLATILSSHDLSLMDQEPLSMVRYGTLVAPSETF

QMIVIK

>CYP120A7(D082_51160)Synechocystis sp. PCC 6714

MTTSPANLDSLPIPPGNFGLPWLGETLNFLNDGDFSKKRQQQFGPIFKTRLFGKNVIFVS

GALANRFLFTKEQETFQATWPLSTRILLGPNALATQMGEIHRSRRKILYQAFLPRALDGY

LPKMDGIIQGYLEQWEKADEVIWYPQLRRMTFDVAATLFMGEGAGQNPHLFSWFEIYIQG

LFSLPIPLPNTLFGKSQRARALLLAELEQIIKTRQQQPPKEEDALGILLTARDDNNQPLS

LSELKDQILLLLFAGHETLTSALSSFCLLLGQHSDIREKVRQEQNKLQLGQKLTAETLKK

MPYLDQVLQEVLRLIPPVGGGFRELIQGCQFQGFHFPKGWLVSYQISQTHADPDLYPNPE

KFDPERFASNGSATHNPPFAHVPFGGGLRECLGKEFARLEMKLFATRLIQQFDWTLLPGQ

NLELVVTPSPRPKDNLQVKLHSLK

>CYP110AE1(Dacsa_1677)Dactylococcopsis salina

MNHQTVPIPKSPTLLQIAQWIFNPLGYMKKNRTEYGDIFQAYVSWGKTNPLLMLSEPKAL

QYILTHDTGKEFTAPGEVNRILEPLLGRQNLILLSGNEHQRRRKLVTPPFHGERLKAYGN

IIQNITQQVIDQWSTEASINVRETMQKITMRVILQAVFGLHEGERFDRLERLLGQRLDMT

GSPLGSLLLFLPFLQKNYGSWSPGDRLQKLAKETDDLLFAEIEERRKNPDPDRIDILSLL

LMAEDEEGNGLTDQDLRDELMTLLVAGHETTATALTWALYWIHSLPEVKDKLLAELDSVT

DKTDSSQFLKLPYLGAVCNETLRFYPVAMLTFPRQVEQPVELCGYQLEPGMLIMGSIYLI

HQREDLYPQPEQFRPERFLERQFSPYEFMPFGGGVRRCVGAALAQYEMKIVLGTMLSQLN

LALLNQRPVSPARRGVTLGQSKSVWVKKVGVRSRSKSVAKV

>CYP110AS1(Dacsa_2941) Dactylococcopsis salina

MEKNLPSLSTPPFLQTLELIANPIRFFDKYQNKYGDIFSARILGNQSPSVHFIGDPKALE

TIFNAPSGQFQLGKITHVFRPFTGDRSLIMLDGEEHRRQRKLLIPPLHGKRMHFYQDVIC

ELTEETIKTLSKNQSFSCRKLMAEITLKVILRVVFGLKPGKRSNDLQKLIADLLDAITNP

LYSSLFFFPSLQIDLGKYSPWGHFIRKQSAIDALIYAEIKERRQQDNSQQTDILSLLLSA

TDEDGNTMSDQELRDQLITLLFLGHETTASSLAWMFYWVYSSPQVREKLATEIQALGNTP

QPQELIQLPYLDAICKETLRLYPIALISQPRVVQETITINHTSFLPDSILVPCIYLAHHR

EENFANSKTFQPDRFLDNQFTAYQYFPFGGGSRACIGAAFSLYEMKLIFGTIFSQLSLKL

ASQKPIKPIRRGITIVPSGGVKLGFAEKVHVFGVVRQKASCLRQEG

>CYP110A13(Dacsa_2943)Dactylococcopsis salina

MITTTPIKQQLPPGPKTSNLVQLMQWIFDPLRLLEKSKQIYGEPFTLEFRKDHPFVFLSH

PETIQEVLSHDRANFDSGRGNYILLPIVGENSILLLDGNDHSRQRRLILPSFHGDKIRRY

GEIMAEVTESVAKQWETKKPFSIRSSMQEITLEVIMQAVFGISDSERHTQLKSRLIKTLE

LTGGSVLRSSLLFFPALQKDFPSSPWRNFLQRQQATNDLLQAEIEARRNENNTAGTDVLS

LLMSARDEAGNPMTDEELKNQLITLLFAGHETTATALAWAFYWIHKSPEVREKLLAELAT

VSDVSDIKALHQLPYLDAVCKETLRIYPVAMITFPRITKHSIKIGDYEYPPETILAPCIY

LLHHREDIFPHSKQFQPDRFLNREFSNYEFLPFGGGSRRCIGDAFAPMEMKIVLATILKK

YPLTLAEKQPVKPVRRGVTIAPAGGIKMIVSH

>CYP197E2(IS3754_14070) Fischerella sp. NIES-3754

MVFVSKPKASASYPPGPKGGFIFGILPEYSRDPLGFISKSAKEYGDVVYWKVAWFSLYQL

NHPDHIQEVLVTKSNLFHKNRSLQLLRRIFGNGLVSSEGEFWQRQRRLMQPAFYRERIFA

YAEVIVDYAQQLLATWQDGDIRDVHEEMMALTLEIVAKTLFEADVEQQVKTIGTALQESI

EYFEARNNNLFLFLLPEWFPTRKNLRFLRAVQQLDEIVYTLIRQRRMSGEDKGDLLSMLL

QVKDEDGIGMSDQQVRDEVMTLFIAGHETTALTMSWTFYLLSQYPEVEAKLVQELQSVLG

GRMPTLADLPQLRYTEWVVMESMRLYPPVWALGRTVMQDCEIAGYRLRAGDSVILSQWVM

HHDPRYFDNPEIFQPERWQGDFAKKLPTFAYFPFGGGPRICIGKSFAIMEAVLLLASIAQ

KFRLMLVPEQKIIPWPAFTLRPKYGIKMLLAER

>CYP110Q2(FIS3754_18990) Fischerella sp. NIES-3754

MDYEFAVKLIPDCHFQDIPMKLPPGPKTPSWLLNLQFEANPFGYMDNIYRRYGDIVMIMS

DSTPVVYVSNSLGIKQIFSNTKEISARGALNQDFALMTGQQGILQLDGLRHKHRRKLLMP

AFHGARMQAYGRRICELTDKIMNQQAIAKPFVAYRTIEDITLRVAIEVVLGLEEGELYNK

IKLLFVSIVRYPQSPLYEFVTKLPWGRRDLGRWSPRGYMLHLRQELFDLLYAEVKKRREQ

ADPSRRSILSDLIFACDETGTVMSDEEVRDLLLSPVFAAQDASATAIAWSLYWIHRLPEV

RDRLLRELDSLGESKDPMSIIELPYLDAVCNEALRIYPTQLFTFPRLVESPVEVMGYDLS

PGTVLIGNIYLTHQQENLYPEPKQFRPERFLKQQFSPYEFLPFGGGSRVCIGATFALFEM

KLVLATILSRYQLTLVSKRPERPKYGNLICYPASGVKMVMHGRRQHQGQSQSFVVGSV

>CYP110C14(FIS3754_37430)Fischerella sp. NIES-3754

MSNCIIYLWIKTYMQLPNRLAIPSFLQKLHWAIDPVTYFERAAEQYSDIFTAQIVGFGDT

VVFITHPQAIQEIFTNDRKKFTAAGKLNRLMQPLLGEKSLLSLDGDRHKRRRQLLMPSFH

GERIRAYGQLICNITEQILSEVPQNQPFLAHTVMQKISLQVILKVVFGLNEGERCQKIQY

LMPLLLDIFRSPLTSSFFLFSFLQKDIGAWSPWGKFLCQRQQLDELIYAEIAERRNQEDP

ERIDILSLLMSACDENGQSMTDQELRDELMTLVFAGHETTATSMAWGLYWIHHLPEVHEK

LKKELDTLENSPDPMRIFHLPYLSAICNETLRITPAVPFTPPRMVRDTVELLGHQLEPGT

VVIPSIYLTHRREDIYPQPQQFKPERFLERQFSPYEFIPFGGGARSCIGQALAMFQMKLV

LTTILSHYQLALVEKQPERLQRRGVVLGPGNGVKMVITKRRKQQEPEFDYTYNAV

>CYP120A11(FIS3754_39010)Fischerella sp. NIES-3754

MTTTNTPTSLPLPPGSFGLPLIGETIPFLNDPNFTEKRYKKYGSLFKTHIFGRPTLIVTG

AEGNRFLFSNDHKYFSNNWPYSTRILLGPASLSVQKGTEHQNRRKLLSQAFQPRALASYA

ITMEQITHQYLDRWEKLATFAWYPELRNYTLDIACKLLVGVDCASQTQLGEWYKIWVNGL

FTLPINLPWTNFGKALRCRKLLLAEIEKIVRQRQKTGNSYQDVLELLLQAEDEEGKRLSL

NELKDQLLTLLFAGHETLTSALASLCLLLVQNPEVLATARSEQQQLGVDTPLTSENLKQM

TYLDQVLKEVMRLIPPVGGGFREVIQSCEFNGFRIPQGWSVLYQVGKTHKDHSVYNHPEA

FDPERFSPQRAEDKPKPFSYIPFGGGVRECLGKEFAKLEMKLFAALLLRHYQWELLPEQN

LEMIMVPTPHPQDGLKVSFKRFLS

>CYP110E26(FIS3754_46220)Fischerella sp. NIES-3754

MKLPDGPHIPPLVQLINVLLRPLETLEENAKLYGDWFTTRITGFPPLVVVSHPQAIQEIL

TADSKLFDAGQTNQILHPLVGDYSLLLMDGDRHQSQRRLLTPPFHGERMRTYGQLICDIT

EKVTDHWTIGKTFVARSILQEISLRVILRAVFGVDEGQRFEELRRLVSKILDTFDSPIKS

SFLFLKILQKDLGSWSPWGRFLRQREQVDKLIYAEIQERRQQSHSSSEDILSLMMSAHDA

SGQTMTDQQLRDELMTLLLAGHETTASALAWALYWIHKQPTVKEKLLQELESIGTHPDPS

EVAKLPYLTAVCQETLRIYPIGLFTFTRILKSPLQLMGYNFEPGTCFAPCIYLVHHREDI

YPQPKQFQPERFLERQYSPYEYLPFGGGSRRCIGMAFALFEMKLVLANILSHWQLDLVDH

RPVHPVRRGVATTPAGGVPMIVVGKKSPTSKPLQSVIS

>CYP110B9(FIS3754_46230)Fischerella sp. NIES-3754

MQQKQRMKLPNGPKTPRLVQMLQWISSPMKYMEDCTQRYGDIFTLQLTGPVVFVSNPQAL

QQMLTSDTKEFAAPGEPLFESFLGKHSVITVSGEVHRRQRQLLMPPFHGERMRTYAQVIT

KVTEEIISQWQIGLPFTVHPAMQAITMRIIMQAVFGLYDSPRAQELEELLTLMLNRAGSS

PLRALMIYFPALQKDLGPLTPWRTFLRRRSRVYQLLQEEIQARREQADSSRTDVLSLLIA

ARDEAGQPMTDAELSDELMTLLVAGHETTATAVTWALYWIHKSPAVREKLLQELDTVSDR

SDSSSIVKLPYLNAVCNESLRIYPVGMLTFARVVQQPLSLCGYELEPGTAVMGSIYLTHQ

REDLYPQPKQFKPERFLERQFSPYEFLPFGGGAKRCIGAAFAQFEMKLILATILSRLKLA

LLDNSDVKPKRRGLVTGPDRPIQMVVSNQRQVESRIPEAISG

>CYP1320A1(AFY58957.1)Rivularia sp. PCC 7116

MVLQTRKSLPTKPGYLLFNSKEIQQNVLNFYNRMWKEYGDIVRLPIMPNYSAHLLAHPSY

AEHVLFTHQELYAKPDMLNKPLNLMMGESILTSEGDSWLKDRRLMQPAFHMKQLANLADV

MVSCTESFIKEWENKTDGEVIDIAEETLRLTLKIAGNTLFSIDISDEDSILGKAFRTGYE

FVNYKINNLWTEPLWMPTLRNRRFIRAKQTLDNLVLDIINSRRQNPSERNDLLSMLMSAR

DEETGEGMSDRQLHNEAITLLVAGHETAASSLAWTWYLLAENPDIAENLQSELRTVLNGS

NLSFEKLPQLEYTRRIFDETLRLYPPAWGMVRTPRQDDEINGYLIAKNSIVTVGAFMIHR

HPEFWENPLGFNPDNFLPEKVNQRPKFAYFPFGGGKRICIGQNFALMEATIIIALVSQRF

KLELLPNQNIEIDPTFTLRPKNGIKVKVWKRN

**False positives**

>(AFY87593.1)Chroococcidiopsis thermalis

MRKIAIGVMGAGENATVIDCTSAYELGKAIAQQGWVLLTGGRNVGVMDAASRGAKSANGL

TVGILPGSDRHDASEAVDIAIATGMGNARNNINVLSSDVIITCGTGAGTISEIALALKAN

KPVILLNSEIESQNFWQKLSVNNIYFVSSVAAAIATVKTLIL

>(BAZ44547.1)Chondrocystis sp. NIES-4102

MNIIGVMGAGNSATPEDLELGYQLGQAIAQEGWVLLTGGRKVGVMDAASRGAKTKQGLVI

GILPGHNKVGMSQAVDIPIITDLGNGRNNINVLSAKVVIACGMGLGTASEVALALKNQKP

VILLNQNELTKQFFASLAPQQLYVSETVPGAIALTQKILISDF

>(AFY29173.1)Cyanobium gracile

MTNPTEELKDIIKNGLASDILKMERAYFLHKAIGTNADIFNASENGSFGELFGAFHGAME

SEAVLAVARVYDKPGKRHPTRCIRRALDLMEQNAESLPEIVEAYNTRLHLETSGANREVI

QSVSDGKAVFIPLYVPYMRGILDSDETLAKVKRLRDLRDKRIAHNDAATFVGPTWDALND

LIKQAQHFVGVVGWAFFSTVYINDNTYLLSSDAQRPARALHRLATLLSQSHGQ

>(ACK72884.1) Gloeothece citriformis

MKKIIIGVMGPGHQATATDINNAYQLGKLIAQQGWVLLSGGRNVGVMDAASRGAKSVGGL

TVGILPHNSLEGVSEAIDIPILTDMGQARNNINVLSSDVVIACGMGLGTASEVALALKND

KPVIILTEDPETIHFFMTLSRDKVLSATTPETAIELVREILRNSA

>(ADN12249.1)Cyanothece sp. PCC 7425

MKKTIIGIMGPGKQATPTDLTYAYQLGQLIAQQGWVLLTGGRNVGVMDAASRGAKSAGGL

TVGILPDDHPQGVSPGVDLVIITDMGNARNNINVLSSDVVIACGMGSGTASEIALALKNH

KSVILLTQHQEAIQFFTNLSRDKVFLATNPKEVIELVKVILSNSA

>(ACK66646.1) Rippkaea orientalis PCC 8801

MRKPIIGVMGPGEQATPTDLKNAYQLGQLIALEGWVLLTGGRNVGVMEHASQGAKKAEGL

TIGILPSKNTHNVSDAVDIAIVTGLGNARNNINVLSSDVVIACGIGLGTLSEVALALKNQ

KPVILLNDDLLSQELFANLSNNQVWIASSPENCIELIKSIITV

>(AFY67675.1)Geitlerinema sp. PCC 7407

MRKILIGVMGPGSAAQPEDVTHAHELGRRIAEAGWVLLTGGRKVGVMEAANQGAHAAGGL

TVGILPGTDDDSVSEAVDIAIFTDLGNARNNLNVLSSRVIIACGFGFGTLSEVALALKSD

RPVIVLTNNGNFKNFLMKISSQNLHLCQTPQEAISLVRQILQTSQTFSNLSKDIGV

>(gvip450)Gloeobacter violaceus

MFAGCGDRVKNFRMLLWCIAGGLLIVSLWVHWQPQPSLGPAGSTDSPASALPQHPLIRVY

FNQARSGRYVDYRGLERPGDDLERRIVEELGRARTSVDIAIHELNLPGIALAIGECDRRG

VAVRVILEHTYHRDFASVAFANRLKSADREAYDRWRQFVDANGDGRVDYGELQKRDAVGI

LKRAKIPVIDDTDGGTRGSGIMHHKFLIIDNRRVVTGSTNFTTSDVHGDPADPRTLGNVN

HLIVIESAQVAGLFAAEFTWMWGDGPGGAPDSLFGRRKPVRPLQSVEVGEARIGVQFGPG

EGEAGINALIARRVTKARRSLDFALFVFSAPEIAKAIQRAAGQGVRVRGALDSGFAYRDY

SMGFDLWGMRPCASKQSPPIKTVGVALLPRGDKLHHKFALLDNGTVLTGSHNWSVAADRR

NDESFLIIESPIVAAHFRREFERLYSRVLLGPPKRKPKTFTCTKEKAKPNHHNR

>(BAU13282.1)Leptolyngbya sp. NIES-3755

MPKTIVGVMGVGEQATPQEIQTAYELGQRIAQAGWIVLSGGRNVGVMDAVSRGAKSVGGM

TIGILPHVDSEVSEAIDIPIITDMGNARNNINVLSSHAIVACGIGAGTVSEIALAIKAKK

HVILLTENREAQEFLRSLNPELVSLAGNVEIALNQLKVILTTQE

>(AFY40326.1)Leptolyngbya sp. PCC 7376

MSQKKVVGVMGAGGGATLENLTDAYELGQAIATNGWATLTGGRPAGVMEAASRGAKGANG

LTVGVLPGGDRRQASEFVDIVICTDLGNARNNVNVLSSNAVVAVGMGMGTASEVALAIKN

SRPVILLKPDAETIAFFCKFAPDQIEIATTIKQAIAFLEHHLT

>(AFZ05656.1)Oscillatoria nigro-viridis

MSQEFVPDDNFSARPNDDSAASEQEVVRIAVFGSRAAVLETIHTLYRLGFAAVGDWSPPQ

RGAQPGQVISVLIRRSRKA

>(BBC24520.1)Pseudanabaena sp. ABRG5-3

MRYYTFGNLPNGSGGDGDRWSGVRFWLTTLLIFWTLSTIGLGWLVNSFFILIGLLTFVPI

IAFFGLQWWVKRSIVTADCPVCEASFTASRATQFQCPNCGEPLQEQQNKFVRITPPGTID

IDVQVVE

>(BAU67318.1)Stanieria sp. NIES-3757

MSKIIIGVMGPGELATPIDIDYAEQLGKLIAENSWVLLTGGRNVGVMNAASLGAKSGGGL

TVGILPDRTTARMSEFVDLAIMTDLGNARNNINVLSATVIVACGMGAGTASEIALALKQQ

KKVILLNADPVSQQFFSNLSPENIVIANDPENAIALIKSFLAQ

>(ABG50829.1)Trichodesmium erythraeum

MKKTIIGVMGPGNSATSTDLKNAYKLGQLIAQESWVLLTGGRNIGVMDAASRGAKFLNGL

TIGILPDNNLHSISEAVDIPIITDMGNSRNNINVLSSDVVIACGMGVGTASEIALAIKNN

KKVILLNNNLESQAFFVALSPQTVFVVNTPEAVIEVVKNLI

>(Cyan7822_0199) Gloeothece verrucosa

MKKTIIGIMGPGKQATPTDLTYAYQLGQLIAQQGWVLLTGGRNVGVMDAASRGAKSAGGL

TVGILPDDHPQGVSPGVDLVIITDMGNARNNINVLSSDVVIACGMGSGTASEIALALKNH

KSVILLTQHQEAIQFFTNLSRDKVFLATNPKEVIELVKVILSNSA

**P450 fragments**

>CYP120A-fragment1(SYNPCC7002_F0082)Synechococcus sp. PCC7002

MDVSISRASGSGIGVIKSRLAGLQIAHQFAEGSSEYSFIAEQDALDIGDGLDMATLQQMT

YLDQVFKEVLRLVPPVGGGFREVINTFEYKNFQIPKGWAVQYQVAQTHKDEALYPDHEKF

DPERFLPERLADKQKKFGFIPFGGGMRECIGKEFARLEAKILASMLARDYDWELLPDQDL

SMQVIPTPLPKDGLQIRFYRREKAS

>CYP2202A1_partial(AII48253.1)Synechococcus sp. KORDI-52

MGKGDDSIAFYEEYFLPMIHEKRSNPSCDLLSRLIADPEEGVHLSDEQLLLIISSNFYSA

SVFTLRLLVSTLAWVMAMNPDAYARVQDNRELVIPALEELLRWDPPAQATNASVALEDIE

IAGKLVKAGDSLSVLVGAANRDPKAFPDPDQFLLDRRPNSHVSFAPGLHQCLGLQIARME

GQAALNAFCDHFSSLSVIESESQRFVGDRFRGFDRLMLEVSHK

>CYP110C-fragment2(AA637_13205)Cyanobacterium sp. HL-69

MKDIKTIFTGLGKKVLWIVNPIEYMRQAVIKSPDIFMSKPAGFRQPLVFINHPQSIQQLL

TNDRKTFFAGGGIKFPFKSHCGQFFSSFP

>CYP120A-fragment2(EAM47592.1)Crocosphaera watsonii WH8501

MSPREDITSENLKEMPYLEQVLKEVLRLIPPVGGGFRKVIQEFEFEGYRIPKDWTVQYQI

AQTHQESDTFPNYQTFDPERFSPENMVDKQKRFGYVPFGGGLRECLGKEFARLEMKIFAS

AA

>CYP110F-fragment1(AUB37976.1)Nostoc flagelliforme

MKVLDNLRTPSLLQTLQLIAQPTKTLENCAAKYGDIFTMRVMGLKSPPIVFFSHPQAISD

CFAIPAKKLDFKKATHVFKPLFGENSIVFKEARSHQQQRQLLLPAFHGDNLKSYGQAICQ

ITEEVTQNWTSGTDICIHSFMSNITLEIILKVVFGITHGARYNTLADITNPEAITQLSYL

NAVCQETLRIHPIALICTPRMVKDRVEMMGHEFTSETVLVPCIYLAHRRAETYPEPEQFR

PERFLNQKFSPYEYFPFGGGYRGCIGAAFSMYEIKLATAIILSRFELSLTDKRPVHPVRR

GITIVPSGGVKMVVTKKAKLKKQTILST

>CYP120A-fragment4(AOX02032.1)Moorea producens

MIPQGWTVLCQITETHNNEEIYQYHQRFDPDRFSPERKEDKQKTFGYIPFGGGLRECLGR

EFAKLEMRIFAAQLLRDYDWTLLPDQDLKMVVMPTPHPRDGLKVKFSRRVGS

>CYP110-fragment1(AP012549.1)Cyanobacterium endosymbiont of Epithemia turgida

-IAFCQKTLYIYTIARFTFGRVTKIFV*IIKNL*QKRCLPFNIY*THHREDTCLKSKQFR

SEYFLEQGHFSCQYLPFNRRIYLCIGYALT*LEINLVLVIILSN*YVKRAYKKSVIPQSC

RLTLPFWGALKMI

>CYP110F-fragment1(COO91_03930)Nostoc flagelliforme

MKVLDNLRTPSLLQTLQLIAQPTKTLENCAAKYGDIFTMRVMGLKSPPIVFFSHPQAISD

CFAIPAKKLDFKKATHVFKPLFGENSIVFKEARSHQQQRQLLLPAFHGDNLKSYGQAICQ

ITEEVTQNWTSGTDICIHSFMSNITLEIILKVVFGITHGARYNTLADITNPEAITQLSYL

NAVCQETLRIHPIALICTPRMVKDRVEMMGHEFTSETVLVPCIYLAHRRAETYPEPEQFR

PERFLNQKFSPYEYFPFGGGYRGCIGAAFSMYEIKLATAIILSRFELSLTDKRPVHPVRR

GITIVPSGGVKMVVTKKAKLKKQTILST

>CYP110A-fragment1(COO91_09923)Nostoc flagelliforme

MRLSGIGSCVILSHPQAIQEIFSQDSKFDIGRGNKLAEPLIGRNSLMLIDGARHRRERKL

LMPPFHGERLQAYGQQICLITEQIASQWQIDQPFVARSAMQKVSLEVILQIVFGLSEGER

YQLILPLLTCSELQT

>CYP120A-fragment3(EAM50941.1)Crocosphaera watsonii

MTQLESKKPASLPPGDKGLPLIGESLSFLFDPDFGKKKLKKYGHVYKTNIFGNNAVIMIG

AEANQFLFRNENQYVVSTWPKSTRILLGKLSLSTNDGTFHTSRRKLLAQAFKPRALNSYI

PKMTEITQQYIDKWLQTKELTWYPELRDYTFDVACSLLISIDNASQTKLASYFETWVKGL

FRVC

>CYP120A-fragment5(BG53951.1)Trichodesmium erythraeum

MTNTNTKFPLPPGSFGLPLVGETLSFLRDNNFAEKREKKYGKIYKSHIFGNPTIFLAGAE

PNKFLFSNENKYFLAK

>CYP107DV-fragment1(AVZ30550.1)Nodularia spumigena

MQTRIHRLAHELIDRAQSKGKMDLIHDFALPIPMVVISEMLGVAEQDRAAFHHWSRVMTS

TSKPIDGILAIPCLYQLVRFLRRLFREHRRNPQDDLTSALLQAESDGSKLSEDELIAMVA

LLLTAGHETTVNDIYAGLTKLVDV

>CYP120A-fragment6(AOX02034.1)Moorea producens

METTTQDMCSLPLPPGNVCLPIIGETISFLTDRNFHKKRLDKYGRIYKTHIFGSPAVTMN

TAEANQFLFTNENQYVASIWPKSTQILLGAASLANQTGVFHQKRRKLMSQAFQPRALASY

LPTMANITSNYLQKWEQMGTLTWYPQLQDYTFEIASTLLMGTDAGSQTPLAQLFNKWGIS

TPQLRPPD
