# Supplementary material for: Comprehensive Analyses of Cytochrome P450 Monooxygenases and Secondary Metabolite Biosynthetic Gene Clusters in Cyanobacteria
Source: Int J Mol Sci. 2020 Jan 19;21(2):656. doi: 10.3390/ijms21020656 (PMC7014017; doi:10.3390/ijms21020656)
Supplement: Supplementary file 1 [file ijms-21-00656-s001.zip › Khumalo et al. 2019 supplementary files/Supplementary information.docx]

Article

Comprehensive analyses of cytochrome P450 monooxygenases and secondary metabolite biosynthetic gene clusters in *Cyanobacteria*

Makhosazana Jabulile Khumalo ^1^, Nomfundo Nzuza ^1^, Tiara Padayachee ^1^, Wanping Chen ^2^, Jae-Hyuk Yu ^3,4^, David R. Nelson ^5^*, and Khajamohiddin Syed ^1^*

^1^ Department of Biochemistry and Microbiology, Faculty of Science and Agriculture, University of Zululand, KwaDlangezwa 3886, South Africa; khosietens@gmail.com (M.J.K.); nomfundonzuza11@gmail.com (N.Z.); teez07padayachee@gmail.com (T.P.); khajamohiddinsyed@gmail.com (K.S.)

^2^ Department of Molecular Microbiology and Genetics, University of Göttingen, Göttingen 37077, Germany; chenwanping1@foxmail.com (W.C.)

^3^ Department of Bacteriology, University of Wisconsin-Madison, 3155 MSB, 1550 Linden Drive, Madison, WI 53706, USA; jyu1@wisc.edu (J-H.Y.)

^4^ Department of Systems Biotechnology, Konkuk University, Seoul 05029, Korea

^5^ Department of Microbiology, Immunology and Biochemistry, University of Tennessee Health

Science Center, Memphis, TN 38163, USA; dnelson@uthsc.edu (D.R.N.)

***** Correspondence: dnelson@uthsc.edu (D.R.N.) and khajamohiddinsyed@gmail.com (K.S.)

Table S1. Information on species and their respective genera used in the study.

| **Genus** | **Total No Species** | **Species have P450** | **Species don’t have P450** | **Species Code** | **Species Name** | **Genome IDs** |
| --- | --- | --- | --- | --- | --- | --- |
| *Synechocystis* | 8 | 8 |  | syn | *Synechocystis* sp. PCC 6803 | BA000022 |
|  |  |  |  | syz | *Synechocystis* sp. PCC 6803 | CP003265 |
|  |  |  |  | syy | *Synechocystis* sp. PCC 6803 GT-S | AP012205 |
|  |  |  |  | syt | *Synechocystis* sp. PCC 6803 GT-I | AP012276 |
|  |  |  |  | sys | *Synechocystis* sp. PCC 6803 PCC-N | AP012277 |
|  |  |  |  | syq | *Synechocystis* sp. PCC 6803 PCC-P | AP012278 |
|  |  |  |  | syj | *Synechocystis* sp. PCC 6714 | CP007542 |
|  |  |  |  | syo | *Synechocystis* sp. IPPAS B-1465 | CP028094 |
| *Synechococcus* | 21 | 16 | 5 | syw | Synechococcus sp. WH8102 | BX548020 |
|  |  |  |  | syc | *Synechococcus elongatus* PCC6301 | AP008231 |
|  |  |  |  | syf | *Synechococcus elongatus* PCC7942 | CP000100 |
|  |  |  |  | syd | Synechococcus sp. CC9605 | CP000110 |
|  |  |  |  | sye | *Synechococcus* sp. CC9902 | CP000097 |
|  |  |  |  | syg | *Synechococcus* sp. CC9311 | CP000435 |
|  |  |  |  | syr | *Synechococcus* sp. RCC307 | CT978603 |
|  |  |  |  | syx | *Synechococcus* sp. WH7803 | CT971583 |
|  |  |  |  | syp | *Synechococcus* sp. PCC7002 | CP000951 |
|  |  |  |  | cya | *Synechococcus* sp. JA-3-3Ab | CP000239 |
|  |  |  |  | cyb | *Synechococcus* sp. JA-2-3B'a(2-13) | CP000240 |
|  |  |  |  | syne | *Synechococcus* sp. PCC 6312 | CP003558 |
|  |  |  |  | synp | *Synechococcus* sp. PCC 7502 | CP003594 |
|  |  |  |  | synk | *Synechococcus* sp. KORDI-100 | CP006269 |
|  |  |  |  | synr | *Synechococcus* sp. KORDI-49 | CP006270 |
|  |  |  |  | synd | *Synechococcus* sp. KORDI-52 | CP006271 |
|  |  |  |  | syu | *Synechococcus* sp. UTEX 2973 | CP006473 |
|  |  |  |  | syh | *Synechococcus* sp. WH 8109 | CP006882 |
|  |  |  |  | synw | *Synechococcus* sp. WH 8103 | LN847356 |
|  |  |  |  | slw | *Synechococcus* *lividus* | CP018092 |
|  |  |  |  | syv | *Synechococcus* sp. PCC 73109 | CP013998 |
| *Thermosynechococcus* | 3 |  | 3 | tel | *Thermosynechococcus elongatus* | BA000039(NC_004113) |
|  |  |  |  | thn | *Thermosynechococcus* sp. NK55 | CP006735 |
|  |  |  |  | tvn | *Thermosynechococcus vulcanus* | GCA_003990665.2 |
| *Cyanobium* | 2 | 2 |  | cgc | *Cyanobium gracile* | CP003495 |
|  |  |  |  | cyi | *Cyanobium* sp. NIES-981 | LT578417 |
| *Dactylococcopsis* | 1 | 1 |  | dsl | *Dactylococcopsis salina* | CP003944 |
| *Chamaesiphon* | 1 | 1 |  | cmp | *Chamaesiphon minutus* | CP003600 |
| *Leptolyngbya* | 4 | 4 |  | lep | *Leptolyngbya* sp. PCC 7376 | CP003946 |
|  |  |  |  | len | *Leptolyngbya* sp. NIES-3755 | AP017308 |
|  |  |  |  | let | *Leptolyngbya* sp. O-77 | AP017367 |
|  |  |  |  | lbo | *Leptolyngbya boryana* | AP014638 |
| *Halomicronema* | 1 | 1 |  | hhg | *Halomicronema hongdechloris* | CP021983 |
| *Pseudanabaena* | 2 | 1 | 1 | pseu | *Pseudanabaena* sp. PCC 7367 | CP003592 |
|  |  |  |  | pser | *Pseudanabaena* sp. ABRG5-3 | AP017560 |
| *Prochlorococcus* | 14 | 2 | 12 | pma | *Prochlorococcus marinus* subsp. *marinus* CCMP1375 | AE017126(NC_005042) |
|  |  |  |  | pmm | *Prochlorococcus marinus* subsp. *pastoris* CCMP1986 | BX548174 |
|  |  |  |  | pmt | *Prochlorococcus marinus*MIT 9313 | BX548175 |
|  |  |  |  | pmn | *Prochlorococcus marinus* NATL2A | CP000095 |
|  |  |  |  | pmi | *Prochlorococcus marinus* MIT 9312 | CP000111 |
|  |  |  |  | pmb | *Prochlorococcus marinus* AS9601 | CP000551 |
|  |  |  |  | pmc | *Prochlorococcus marinus* MIT 9515 | CP000552 |
|  |  |  |  | pmf | *Prochlorococcus marinus* MIT 9303 | CP000554 |
|  |  |  |  | pmg | *Prochlorococcus marinus* MIT 9301 | CP000576 |
|  |  |  |  | pmh | *Prochlorococcus marinus* MIT 9215 | CP000825 |
|  |  |  |  | pmj | *Prochlorococcus marinus* MIT 9211 | CP000878 |
|  |  |  |  | pme | *Prochlorococcus marinus* NATL1A | CP000553 |
|  |  |  |  | prc | *Prochlorococcus* sp. MIT 0604 | CP007753 |
|  |  |  |  | prm | *Prochlorococcus* sp. MIT 0801 | CP007754 |
| *Acaryochloris* | 1 | 1 |  | amr | *Acaryochloris marina* | CP000828 |
| *Gloeocapsa* | 1 | 1 |  | glp | *Gloeocapsa* sp. PCC 7428 | CP003646 |
| *Geminocystis* | 2 | 2 |  | gen | *Geminocystis* sp. NIES-3709 | AP014821 |
|  |  |  |  | gee | *Geminocystis* sp. NIES-3708 | AP014815 |
| *Chondrocystis* | 1 | 1 |  | chon | *Chondrocystis* sp. NIES-4102 | AP018281 |
| *Microcystis* | 3 | 3 |  | mar | *Microcystis aeruginosa* | AP009552 |
|  |  |  |  | mpk | *Microcystis panniformis* | CP011339 |
|  |  |  |  | miq | *Microcystis* sp. MC19 | CP020664 |
| *Cyanobacterium* | 3 | 2 | 1 | can | *Cyanobacterium aponinum* | CP003947 |
|  |  |  |  | csn | *Cyanobacterium stanieri* | CP003940 |
|  |  |  |  | cyl | *Cyanobacterium* sp. HL-69 | CP024914 |
| *Halothece* | 1 | 1 |  | hao | *Halothece* sp. PCC 7418 | CP003945 |
| *Atelocyanobacterium* | 1 |  | 1 | cyu | *Candidatus Atelocyanobacterium thalassa* | CP001842 |
| *Crocosphaera* | 1 |  | 1 | cwa | *Crocosphaera watsonii* | CP000806 |
| *Cyanothece* | 6 | 6 |  | cyt | *Crocosphaera subtropica* | GCA_000167195.1 |
|  |  |  |  | cyp | *Rippkaea orientalis* PCC 8801 | CP001287 |
|  |  |  |  | cyh | *Rippkaea orientalis* PCC 8802 | CP002198 |
|  |  |  |  | cyc | *Gloeothece citriformis* | CP001291 |
|  |  |  |  | cyj | *Gloeothece verrucosa* | CP002198 |
|  |  |  |  | cyn | *Cyanothece* sp. PCC 7425 | CP001701 |
| *Trichodesmium* | 1 | 1 |  | ter | *Trichodesmium erythraeum* | CP000393 |
| *Microcoleus* | 1 | 1 |  | mic | *Microcoleus* sp. PCC 7113 | CP003630 |
| *Arthrospira* | 1 | 1 |  | arp | *Arthrospira platensis* | AP011615 |
| *Planktothrix* | 1 | 1 |  | pagh | *Planktothrix agardhii* | AP017991 |
| *Geitlerinema* | 1 | 1 |  | gei | *Geitlerinema* sp. PCC 7407 | CP003591 |
| *Oscillatoria* | 2 | 2 |  | oac | *Oscillatoria acuminata* | CP003607 |
|  |  |  |  | oni | *Oscillatoria nigro-viridis* | CP003614 |
| *Moorea* | 1 | 1 |  | mpro | *Moorea producens* | CP017600 |
| *Crinalium* | 1 | 1 |  | cep | *Crinalium epipsammum* | CP003620 |
| *Gloeobacter* | 2 | 2 |  | gvi | *Gloeobacter violaceus* | BA000045(NC_005125) |
|  |  |  |  | glj | *Gloeobacter kilaueensis* | CP003587 |
| *Nostoc* | 7 | 7 |  | ana | *Nostoc* sp. PCC 7120 | BA000019 |
|  |  |  |  | npu | *Nostoc punctiforme* | CP001037 |
|  |  |  |  | nos | *Nostoc* sp. PCC 7107 | CP003548 |
|  |  |  |  | nop | *Nostoc* sp. PCC 7524 | CP003552 |
|  |  |  |  | non | *Nostoc* sp. NIES-3756 | AP017295 |
|  |  |  |  | nfl | *Nostoc flagelliforme* | CP024785 |
|  |  |  |  | noe | *Nostoc* sp. CENA543 | CP023278 |
| *Trichormus* | 2 | 2 |  | ava | *Trichormus variabilis* | CP000117 |
|  |  |  |  | naz | *Nostoc azollae* 0708 | CP002059 |
| *Anabaena* | 3 | 3 |  | anb | *Anabaena* sp. 90 | CP003285 |
|  |  |  |  | acy | *Anabaena cylindrica* | CP003659 |
|  |  |  |  | awa | *Anabaena* sp. wa102 | CP011456 |
| *Cylindrospermum* | 1 | 1 |  | csg | *Cylindrospermum stagnale* | CP003642 |
| *Calothrix* | 3 | 3 |  | calo | *Calothrix* sp. PCC 7507 | CP003943 |
|  |  |  |  | calt | *Calothrix* sp. PCC 6303 | CP003610 |
|  |  |  |  | calh | *Calothrix* sp. 336/3 | CP011382 |
| *Rivularia* | 1 | 1 |  | riv | *Rivularia* sp. PCC 7116 | CP003549 |
| *Fischerella* | 1 | 1 |  | fis | *Fischerella* sp. NIES-3754 | AP017305 |
| *Nodularia* | 1 | 1 |  | nsp | *Nodularia spumigena* | CP020114 |
| Unclassified *Nostocales* | 1 | 1 |  | ncn | *Nostocales cyanobacterium* HT-58-2 | CP019636 |
| *Chroococcidiopsis* | 1 | 1 |  | cthe | *Chroococcidiopsis thermalis* | CP003597 |
| *Pleurocapsa* | 1 | 1 |  | plp | *Pleurocapsa* sp. PCC 7327 | CP003590 |
| *Stanieria* | 2 | 2 |  | scs | *Stanieria cyanosphaera* | CP003653 |
|  |  |  |  | stan | *Stanieria* sp. NIES-3757 | AP017375 |
| Unclassified *Cyanobacteria* | 2 |  | 2 | ceo | Cyanobacterium endosymbiont of *Epithemia turgida* | AP012549 |
|  |  |  |  | cer | Cyanobacterium endosymbiont of *Rhopalodia gibberula* | AP018341 |
| **Total number** | **114** | **88** | **26** |  |  |  |

Table S2. Information on cyanobacterial species P450s and those associated with secondary metabolite biosynthetic gene clusters. Standard abbreviations representing type of clusters as indicated in anti-SMASH (antibiotics & Secondary Metabolite Analysis Shell) [66] were used in the Table.

| **Species name** | **Species code** | **No. of P450s** | **P450 fragments** | **False positives** | **No. of P450 families** | **No. of P450 subfamilies** | **No. of BGCs** | **No of BGCs have P450s** | **BGC Type** | **No of P450s in BGC** | **P450 name** |
| --- | --- | --- | --- | --- | --- | --- | --- | --- | --- | --- | --- |
| *Anabaena cylindrica* | Acy | 3 |  |  | 2 | 3 | 12 |  |  |  |  |
| *Acaryochloris marina* | Amr | 11 |  |  | 7 | 10 | 10 |  |  |  |  |
| *Nostoc* sp. PCC 7120 | Ana | 6 |  |  | 2 | 6 |  |  |  |  |  |
| *Anabaena* sp. 90 | Anb | 2 |  |  | 2 | 2 | 1 |  |  |  |  |
| *Arthrospira platensis* | Arp | 2 |  |  | 1 | 2 | 2 |  |  |  |  |
| *Trichormus variabilis* | Ava | 4 |  |  | 2 | 4 | 14 |  |  |  |  |
| *Anabaena* sp. WA102 | Awa | 3 |  |  | 2 | 3 | 12 |  |  |  |  |
| *Calothrix* sp. 336/3 | Calh | 5 |  |  | 1 | 5 | 8 | 1 | NRPS,T1PKS | 2 | CYP110Q4, CYP110AT1 |
| *Calothrix* sp. PCC 7507 | Calo | 5 |  |  | 2 | 5 | 11 | 1 | terpene | 1 | CYP110C21 |
| *Calothrix* sp. PCC 6303 | Calt | 5 |  |  | 4 | 5 | 11 |  |  |  |  |
| *Cyanobacterium aponinum* | Can | 2 |  |  | 2 | 2 | 8 |  |  |  |  |
| Cyanobacterium endosymbiont of *Epithemia turgida* | Ceo |  | 1 |  |  |  | 3 |  |  |  |  |
| *Crinalium epipsammum* | Cep | 4 |  |  | 3 | 4 | 9 |  |  |  |  |
| Cyanobacterium endosymbiont of *Rhopalodia gibberula* | Cer |  |  |  |  |  | 3 |  |  |  |  |
| *Cyanobium gracile* | Cgc | 1 |  | 1 | 1 | 1 | 7 |  |  |  |  |
| *Chondrocystis* sp. NIES-4102 | Chon | 4 |  | 1 | 2 | 4 | 10 |  |  |  |  |
| *Chamaesiphon minutus* | Cmp | 4 |  |  | 2 | 3 | 16 |  |  |  |  |
| *Cylindrospermum stagnale* | Csg | 6 |  |  | 4 | 5 | 23 | 3 | NRPS,T1PKS | 1 | CYP197E3 |
|  |  |  |  |  |  |  |  |  | terpene | 1 | CYP110AG1 |
|  |  |  |  |  |  |  |  |  | terpene,thiopeptide,T1PKS,NRPS | 2 | CYP110E29, CYP110E18 |
| *Cyanobacterium stanieri* | Csn | 2 |  |  | 2 | 2 | 3 |  |  |  |  |
| *Chroococcidiopsis thermalis* | Cthe | 6 |  | 1 | 2 | 6 | 12 |  |  |  |  |
| *Crocosphaera watsonii* | Cwa | 3 | 2 |  | 2 | 3 |  |  |  |  |  |
| *Synechococcus* sp. JA-3-3Ab | Cya |  |  |  |  |  | 1 |  |  |  |  |
| *Synechococcus* sp. JA-2-3B'a(2-13) | Cyb |  |  |  |  |  | 1 |  |  |  |  |
| *Gloeothece citriformis* | Cyc | 6 |  | 1 | 2 | 5 | 9 | 1 | NRPS | 1 | CYP110K6 |
| *Rippkaea orientalis* PCC 8802 | Cyh | 7 |  |  | 4 | 7 | 8 |  |  |  |  |
| *Cyanobium* sp. NIES-981 | Cyi | 2 |  |  | 2 | 2 | 8 |  |  |  |  |
| *Gloeothece verrucosa* | Cyj | 7 |  | 1 | 4 | 7 | 7 |  |  |  |  |
| *Cyanobacterium* sp. HL-69 | Cyl |  | 1 |  |  |  |  |  |  |  |  |
| *Cyanothece* sp. PCC 7425 | Cyn | 7 |  | 1 | 3 | 7 |  |  |  |  |  |
| *Rippkaea orientalis* PCC 8801 | Cyp | 7 |  | 1 | 4 | 7 | 8 |  |  |  |  |
| *Crocosphaera subtropica* | Cyt | 7 |  |  | 4 | 6 | 7 |  |  |  |  |
| *Candidatus Atelocyanobacterium Thalassa* | Cyu |  |  |  |  |  | 3 |  |  |  |  |
| *Dactylococcopsis salina* | Dsl | 3 |  |  | 1 | 3 | 2 |  |  |  |  |
| *Fischerella* sp. NIES-3754 | Fis | 6 |  |  | 2 | 6 | 13 | 2 | NRPS,T1PKS | 1 | CYP110Q2 |
|  |  |  |  |  |  |  |  |  | terpene | 1 | CYP110C14 |
| *Geminocystis* sp. NIES-3708 | Gee | 1 |  |  | 1 | 1 | 3 |  |  |  |  |
| *Geitlerinema* sp. PCC 7407 | Gei | 1 |  | 1 | 1 | 1 | 5 | 1 | bacteriocin | 1 | CYP120A21 |
| *Geminocystis* sp. NIES-3709 | Gen | 2 |  |  | 2 | 2 | 5 |  |  |  |  |
| *Gloeobacter kilaueensis* | Glj | 4 |  |  | 3 | 4 | 6 |  |  |  |  |
| *Gloeocapsa* sp. PCC 7428 | Glp | 7 |  |  | 4 | 7 | 8 | 1 | T2PKS | 1 | CYP120C2 |
| *Gloeobacter violaceus* | Gvi | 4 |  | 1 | 3 | 3 |  |  |  |  |  |
| *Halothece* sp. PCC 7418 | Hao | 4 |  |  | 2 | 4 | 3 |  |  |  |  |
| *Halomicronema hongdechloris* | Hhg | 3 |  |  | 3 | 3 | 9 |  |  |  |  |
| *Leptolyngbya boryana* | Lbo | 7 |  |  | 3 | 5 | 12 |  |  |  |  |
| *Leptolyngbya* sp. NIES-3755 | Len | 3 |  | 1 | 2 | 2 | 9 |  |  |  |  |
| *Leptolyngbya* sp. PCC 7376 | Lep | 2 |  | 1 | 2 | 2 | 8 |  |  |  |  |
| *Leptolyngbya* sp. O-77 | Let | 1 |  |  | 1 | 1 | 2 |  |  |  |  |
| *Microcystis aeruginosa* | Mar | 2 |  |  | 2 | 2 | 11 |  |  |  |  |
| *Microcoleus* sp. PCC 7113 | Mic | 10 |  |  | 6 | 10 | 8 | 1 | NRPS,T1PKS | 1 | CYP110C29 |
| *Microcystis* sp. MC19 | Miq | 2 |  |  | 2 | 2 | 9 |  |  |  |  |
| *Microcystis panniformis* | Mpk | 2 |  |  | 2 | 2 | 11 |  |  |  |  |
| *Moorea producens* | Mpro | 9 | 2 |  | 6 | 8 |  |  |  |  |  |
| *Nostoc azollae* 0708 | Naz | 1 |  |  | 1 | 1 | 5 |  |  |  |  |
| *Nostocales cyanobacterium* HT-58-2 | Ncn | 13 |  |  | 5 | 11 | 17 |  |  |  |  |
| *Nostoc flagelliforme* | Nfl | 12 | 3 |  | 8 | 11 | 16 | 2 | NRPS,T1PKS | 1 | CYP1011G1 |
|  |  |  |  |  |  |  |  |  | terpene | 1 | CYP110AP1 |
| *Nostoc* sp. CENA543 | Noe | 9 |  |  | 3 | 8 | 18 | 1 | NRPS,T1PKS | 2 | CYP110AT1, CYP110Q4 |
| *Nostoc* sp. NIES-3756 | Non | 6 |  |  | 3 | 5 | 15 |  |  |  |  |
| *Nostoc* sp. PCC 7524 | Nop | 7 |  |  | 5 | 6 | 15 |  |  |  |  |
| *Nostoc* sp. PCC 7107 | Nos | 6 |  |  | 3 | 6 | 12 | 2 | NRPS,T1PKS | 1 | CYP110Q3 |
|  |  |  |  |  |  |  |  |  | terpene | 1 | CYP110C17 |
| *Nostoc punctiforme* | Npu | 10 |  |  | 4 | 8 |  |  |  |  |  |
| *Nodularia spumigena* | Nsp | 4 | 1 |  | 3 | 4 | 7 |  |  |  |  |
| *Oscillatoria acuminata* | Oac | 3 |  |  | 2 | 3 | 9 | 1 | NRPS-like | 1 | CYP110C21 |
| *Oscillatoria nigro-viridis* | Oni | 3 |  | 1 | 2 | 3 | 7 |  |  |  |  |
| *Planktothrix agardhii* | Pagh | 3 |  |  | 2 | 3 | 8 |  |  |  |  |
| *Pleurocapsa* sp. PCC 7327 | Plp | 2 |  |  | 1 | 2 | 11 |  |  |  |  |
| *Prochlorococcus marinus* subsp. *marinus* CCMP1375 | Pma |  |  |  |  |  | 4 |  |  |  |  |
| *Prochlorococcus marinus* AS9601 | Pmb |  |  |  |  |  | 4 |  |  |  |  |
| *Prochlorococcus marinus* MIT 9515 | Pmc |  |  |  |  |  | 5 |  |  |  |  |
| *Prochlorococcus marinus* NATL1A | Pme |  |  |  |  |  | 5 |  |  |  |  |
| *Prochlorococcus marinus* MIT 9303 | Pmf | 1 |  |  | 1 | 1 | 23 | 1 | bacteriocin | 1 | CYP213A3 |
| *Prochlorococcus marinus* MIT 9301 | Pmg |  |  |  |  |  | 4 |  |  |  |  |
| *Prochlorococcus marinus* MIT 9215 | Pmh |  |  |  |  |  | 5 |  |  |  |  |
| *Prochlorococcus marinus* MIT 9312 | Pmi |  |  |  |  |  | 4 |  |  |  |  |
| *Prochlorococcus marinus* MIT 9211 | Pmj |  |  |  |  |  | 5 |  |  |  |  |
| *Prochlorococcus marinus* subsp. *pastoris* CCMP1986 | Pmm |  |  |  |  |  | 4 |  |  |  |  |
| *Prochlorococcus marinus* NATL2A | Pmn |  |  |  |  |  | 5 |  |  |  |  |
| *Prochlorococcus marinus* MIT 9313 | Pmt | 1 |  |  | 1 | 1 | 17 |  |  |  |  |
| *Prochlorococcus* sp. MIT 0604 | Prc |  |  |  |  |  | 4 |  |  |  |  |
| *Prochlorococcus* sp. MIT 0801 | Prm |  |  |  |  |  | 4 |  |  |  |  |
| *Pseudanabaena* sp. ABRG5-3 | Pser | 4 |  | 1 | 4 | 4 | 3 |  |  |  |  |
| *Pseudanabaena* sp. PCC 7367 | Pseu |  |  |  |  |  | 2 |  |  |  |  |
| *Rivularia* sp. PCC 7116 | Riv | 16 |  |  | 11 | 15 | 14 | 2 | ladderane | 1 | CYP120A13 |
|  |  |  |  |  |  |  |  |  | lassopeptide,bacteriocin | 1 | CYP1185A1 |
| *Stanieria cyanosphaera* | Scs | 5 |  |  | 3 | 5 | 12 |  |  |  |  |
| *Synechococcus lividus* | Slw |  |  |  |  |  | 3 |  |  |  |  |
| *Stanieria* sp. NIES-3757 | Stan | 4 |  | 1 | 4 | 4 | 9 |  |  |  |  |
| *Synechococcus elongatus* PCC6301 | Syc | 1 |  |  | 1 | 1 | 2 |  |  |  |  |
| *Synechococcus* sp. CC9605 | Syd | 1 |  |  | 1 | 1 | 7 |  |  |  |  |
| *Synechococcus* sp. CC9902 | Sye | 1 |  |  | 1 | 1 | 11 |  |  |  |  |
| *Synechococcus elongatus* PCC7942 | Syf | 1 |  |  | 1 | 1 | 3 |  |  |  |  |
| *Synechococcus* sp. CC9311 | Syg | 3 |  |  | 3 | 3 | 10 | 1 | T3PKS | 1 | CYP213A8 |
| *Synechococcus* sp. WH 8109 | Syh | 1 |  |  | 1 | 1 | 6 |  |  |  |  |
| *Synechocystis* sp. PCC 6714 | Syj | 1 |  |  |  |  | 3 |  |  |  |  |
| *Synechocystis* sp. PCC 6803 | Syn | 1 |  |  | 1 | 1 |  |  |  |  |  |
| Synechococcus sp. KORDI-52 | Synd | 3 | 1 |  | 2 | 3 | 7 |  |  |  |  |
| *Synechococcus* sp. PCC 6312 | Syne |  |  |  |  |  | 3 |  |  |  |  |
| *Synechococcus* sp. KORDI-100 | Synk | 1 |  |  | 1 | 1 | 12 |  |  |  |  |
| *Synechococcus* sp. PCC 7502 | Synp | 3 |  |  |  |  | 6 | 1 | NRPS | 1 | CYP110AH1 |
| *Synechococcus* sp. KORDI-49 | Synr | 1 |  |  | 1 | 1 | 5 |  |  |  |  |
| *Synechococcus* sp. WH 8103 | Synw | 1 |  |  | 1 | 1 | 8 |  |  |  |  |
| *Synechocystis* sp. IPPAS B-1465 | Syo | 1 |  |  | 1 | 1 | 3 |  |  |  |  |
| *Synechococcus* sp. PCC7002 | Syp | 2 | 1 |  |  |  | 3 |  |  |  |  |
| *Synechocystis* sp. PCC 6803 PCC-P | Syq | 1 |  |  | 1 | 1 | 3 |  |  |  |  |
| *Synechococcus* sp. RCC307 | Syr | 1 |  |  | 1 | 1 | 6 | 1 | bacteriocin | 1 | CYP213A5 |
| *Synechocystis* sp. PCC 6803 PCC-N | Sys | 1 |  |  | 1 | 1 | 3 |  |  |  |  |
| *Synechocystis* sp. PCC 6803 GT-I | Syt | 1 |  |  | 1 | 1 | 3 |  |  |  |  |
| *Synechococcus* sp. UTEX 2973 | Syu |  |  |  |  |  |  |  |  |  |  |
| *Synechococcus* sp. PCC 73109 | Syv | 2 |  |  | 2 | 2 | 3 |  |  |  |  |
| *Synechococcus* sp. WH8102 | Syw | 1 |  |  | 1 | 1 | 8 |  |  |  |  |
| *Synechococcus* sp. WH7803 | Syx | 1 |  |  | 1 | 1 | 12 | 1 | T3PKS | 1 | CYP213A6 |
| *Synechocystis* sp. PCC 6803 GT-S | Syy | 1 |  |  | 1 | 1 | 3 |  |  |  |  |
| *Synechocystis* sp. PCC 6803 | Syz | 1 |  |  | 1 | `1 | 3 |  |  |  |  |
| *Thermosynechococcus elongatus* | Tel |  |  |  |  |  | 7 |  |  |  |  |
| *Trichodesmium erythraeum* | Ter | 5 | 1 | 1 | 3 | 5 |  |  |  |  |  |
| *Thermosynechococcus* sp. NK55 | Thn |  |  |  |  |  |  |  |  |  |  |
| *Thermosynechococcus vulcanus* | Tvn |  |  |  |  |  | 3 |  |  |  |  |
| **Total** |  | **341** | **13** | **15** |  |  | **770** | **24** |  | **27** |  |

Table S3. Information on P450 families and subfamilies in cyanobacterial species.

| **Family** | **Subfamily** | **Count** |
| --- | --- | --- |
| CYP1007 | A | 7 |
|  | M | 1 |
| CYP1011 | A | 1 |
|  | B | 1 |
|  | G | 1 |
| CYP107 | DV | 1 |
|  | DX | 1 |
|  | NL | 1 |
|  | NM | 1 |
| CYP1094 | E | 1 |
| CYP109 | AL | 1 |
| CYP110 | A | 15 |
|  | AE | 4 |
|  | AF | 3 |
|  | AG | 1 |
|  | AH | 1 |
|  | AJ | 1 |
|  | AK | 2 |
|  | AL | 1 |
|  | AM | 4 |
|  | AN | 1 |
|  | AP | 1 |
|  | AQ | 2 |
|  | AR | 1 |
|  | AS | 2 |
|  | AT | 2 |
|  | B | 17 |
|  | C | 34 |
|  | D | 26 |
|  | E | 29 |
|  | F | 5 |
|  | G | 2 |
|  | K | 6 |
|  | L | 3 |
|  | M | 8 |
|  | P | 1 |
|  | Q | 4 |
| CYP1136 | A | 6 |
| CYP1146 | A | 2 |
| CYP1156 | A | 2 |
| CYP1158 | A | 1 |
| CYP1159 | A | 3 |
| CYP1177 | A | 1 |
| CYP1184 | A | 3 |
| CYP1185 | A | 1 |
| CYP120 | A | 35 |
|  | B | 16 |
|  | C | 5 |
|  | K | 1 |
|  | L | 1 |
|  | M | 1 |
| CYP1317 | A | 1 |
| CYP1320 | A | 1 |
|  | B | 1 |
|  | C | 1 |
| CYP1336 | A | 1 |
| CYP152 | E | 1 |
| CYP1536 | A | 1 |
| CYP1719 | A | 1 |
| CYP1762 | B | 1 |
| CYP1851 | A | 2 |
| CYP1912 | A | 2 |
| CYP1976 | A | 1 |
| CYP1676 | A | 1 |
| CYP197 | A | 1 |
|  | B | 4 |
|  | E | 4 |
|  | X | 2 |
| CYP2029 | A | 2 |
| CYP213 | A | 16 |
| CYP214 | A | 1 |
| CYP227 | A | 7 |
| CYP2287 | A | 4 |
| CYP2337 | A | 1 |
| CYP233 | A | 1 |
| CYP2725 | A | 1 |
| CYP284 | A | 9 |
|  | B | 1 |
|  | C | 1 |

Table S4. Secondary metabolite biosynthetic gene cluster analysis in cyanobacterial species. Standard abbreviations representing type of clusters as indicated in anti-SMASH (antibiotics & Secondary Metabolite Analysis Shell) [66] were used in the Table.

| **Type of Cluster** | **Count** | **Most similar known cluster** | **Count** |
| --- | --- | --- | --- |
| terpene | 235 | Heterocyst glycolipids | 25 |
| bacteriocin | 186 | 1-heptadecene | 17 |
| NRPS | 64 | Nostopeptolide | 12 |
| NRPS,T1PKS | 36 | Nostophycin | 12 |
| NRPS-like | 32 | Puwainaphycins | 9 |
| lassopeptide | 16 | 6,6'-oxybis(2,4-dibromophenol) | 7 |
| lanthipeptide | 13 | Anabaenopeptin | 6 |
| T3PKS | 13 | Anabaenopeptin NZ 857 / nostamide A | 6 |
| T1PKS | 12 | Hexose-palythine-serine / hexose-shinorine | 6 |
| terpene,bacteriocin | 12 | Hapalosin | 5 |
| T1PKS,NRPS-like | 10 | Calicheamicin | 4 |
| cyanobactin | 9 | Carbamidocyclophane | 4 |
| hglE-KS,T1PKS | 9 | Colicin V | 4 |
| ladderane | 9 | Microviridin K | 4 |
| microviridin | 9 | Shinorine | 4 |
| T1PKS,hglE-KS | 8 | Trichamide | 4 |
| hglE-KS | 7 | Yersiniabactin | 4 |
| lanthipeptide,bacteriocin | 7 | Aeruginosin | 3 |
| siderophore | 5 | Anacyclamide | 3 |
| T1PKS,NRPS | 5 | Anatoxin | 3 |
| T1PKS,terpene | 4 | Cryptophycin | 3 |
| arylpolyene | 3 | Cyanopeptin | 3 |
| indole | 3 | Nocuolin A | 3 |
| LAP | 3 | Nodularin | 3 |
| NRPS-like,T1PKS | 3 | Piricyclamide | 3 |
| T1PKS,NRPS,hglE-KS | 3 | Aeruginoside | 2 |
| thiopeptide | 3 | Anacyclamide D8P | 2 |
| betalactone | 2 | Asukamycin | 2 |
| cyanobactin,LAP | 2 | Curacin | 2 |
| oligosaccharide | 2 | Cylindrocyclophane | 2 |
| resorcinol | 2 | Cystothiazole A | 2 |
| T1PKS,NRPS-like,terpene | 1 | Divamide A | 2 |
| T3PKS,bacteriocin | 2 | Jamaicamide | 2 |
| T3PKS,T1PKS | 1 | Kijanimicin | 2 |
| amglyccycl | 1 | Merocyclophane C / merocyclophane D | 2 |
| bacteriocin,lanthipeptide | 1 | Microcystin | 2 |
| bacteriocin,NRPS | 1 | Micropeptin | 2 |
| bacteriocin,proteusin | 1 | Microviridin B | 2 |
| bacteriocin,terpene | 1 | Nonadecene | 2 |
| betalactone,NRPS | 1 | Saxitoxin | 2 |
| betalactone,NRPS,microviridin | 1 | Tenuecyclamide A / tenuecyclamide C | 2 |
| betalactone,NRPS-like,T1PKS | 1 | TP-1161 | 2 |
| betalactone,T1PKS,NRPS-like | 1 | Ajudazol | 1 |
| cyanobactin,thiopeptide,LAP,bacteriocin | 1 | Ambiguine | 1 |
| ectoine | 1 | Bartoloside 2 / bartoloside 3 / bartoloside 4 | 1 |
| hglE-KS,resorcinol | 1 | Bartolosides E / bartolosides F / bartolosides G / bartolosides H / bartolosides I / bartolosides J / bartolosides K | 1 |
| hglE-KS,T1PKS,NRPS | 1 | Bicornutin | 1 |
| hserlactone | 1 | Cahuitamycins | 1 |
| LAP,bacteriocin | 1 | Chondrochloren | 1 |
| LAP,bacteriocin,NRPS-like,NRPS,T1PKS | 1 | Crocacin | 1 |
| LAP,cyanobactin | 1 | Cyanopeptolin | 1 |
| lassopeptide,bacteriocin | 1 | Cylindrospermopsin | 1 |
| lassopeptide,ladderane | 1 | Desferrioxamine | 1 |
| microviridin,NRPS | 1 | Epothilone | 1 |
| NRPS,betalactone | 1 | GE2270 | 1 |
| NRPS,hglE-KS,resorcinol | 1 | Geosmin | 1 |
| NRPS,LAP | 1 | Hectochlorin | 1 |
| NRPS,microviridin | 1 | Heme D1 | 1 |
| NRPS,T1PKS,CDPS | 1 | Hydroxysporine | 1 |
| NRPS,T1PKS,NRPS-like | 1 | Luminaolide | 1 |
| NRPS-like,indole | 1 | Malleobactin | 1 |
| NRPS-like,indole,T1PKS | 1 | Melithiazol | 1 |
| NRPS-like,T1PKS,T3PKS | 1 | Microcystin-LR / microcystin-FR / microcystin-LA / microcystin-LAba / microcystin-LM / microcystin-LV / microcystin-LL | 1 |
| phosphonate,terpene | 1 | Microsclerodermins | 1 |
| proteusin | 1 | Myxalamid | 1 |
| T1PKS,NRPS,lanthipeptide | 1 | Myxochromide S | 1 |
| T2PKS | 1 | Neocarzinostatin | 1 |
| T3PKS,T1PKS,PKS-like | 1 | Nosiheptide | 1 |
| T3PKS,terpene | 1 | PcpA | 1 |
| terpene,thiopeptide | 1 | Prenylagaramide B / prenylagaramide C | 1 |
| terpene,thiopeptide,T1PKS,NRPS | 1 | Pseudospumigin A / pseudospumigin B / pseudospumigin C / pseudospumigin D / pseudospumigin E / pseudospumigin F | 1 |
| thiopeptide,LAP | 1 | Scytonemin | 1 |
| transAT-PKS-like | 1 | Spumigin | 1 |
| **73 types** | 770 | SRO15-3108 | 1 |
|  |  | Stigmatellin | 1 |
|  |  | Vioprolide | 1 |
|  |  | Viridisamide A | 1 |
|  |  | Xenocyloins | 1 |
|  |  | Xenotetrapeptide | 1 |
|  |  | **79 known clusters** | 228 |

Table S5. List of species that are not part of secondary metabolite biosynthetic cluster analysis as the genome IDs of species did not give any results at anti-SMASH (antibiotics & Secondary Metabolite Analysis Shell) [66].

*Crocosphaera watsonii*

*Cyanobacterium* sp. HL-69

*Cyanothece* sp. PCC 7425

*Gloeobacter violaceus*

*Moorea producens*

*Nostoc punctiforme*

*Nostoc* sp. PCC 7120

*Synechococcus* sp. UTEX 2973

*Synechocystis* sp. PCC 6803 GT-S

*Thermosynechococcus elongatus*

*Thermosynechococcus* sp. NK55
