# Supplementary figures and images for: Comprehensive Analyses of Cytochrome P450 Monooxygenases and Secondary Metabolite Biosynthetic Gene Clusters in Cyanobacteria
Source: Int J Mol Sci. 2020 Jan 19;21(2):656. doi: 10.3390/ijms21020656 (PMC7014017; doi:10.3390/ijms21020656)

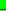 CYP110  
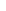 CYP120  
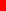 CYP213  
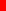 CYP197  
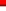 CYP1007  
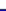 CYP284  
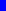 CYP227

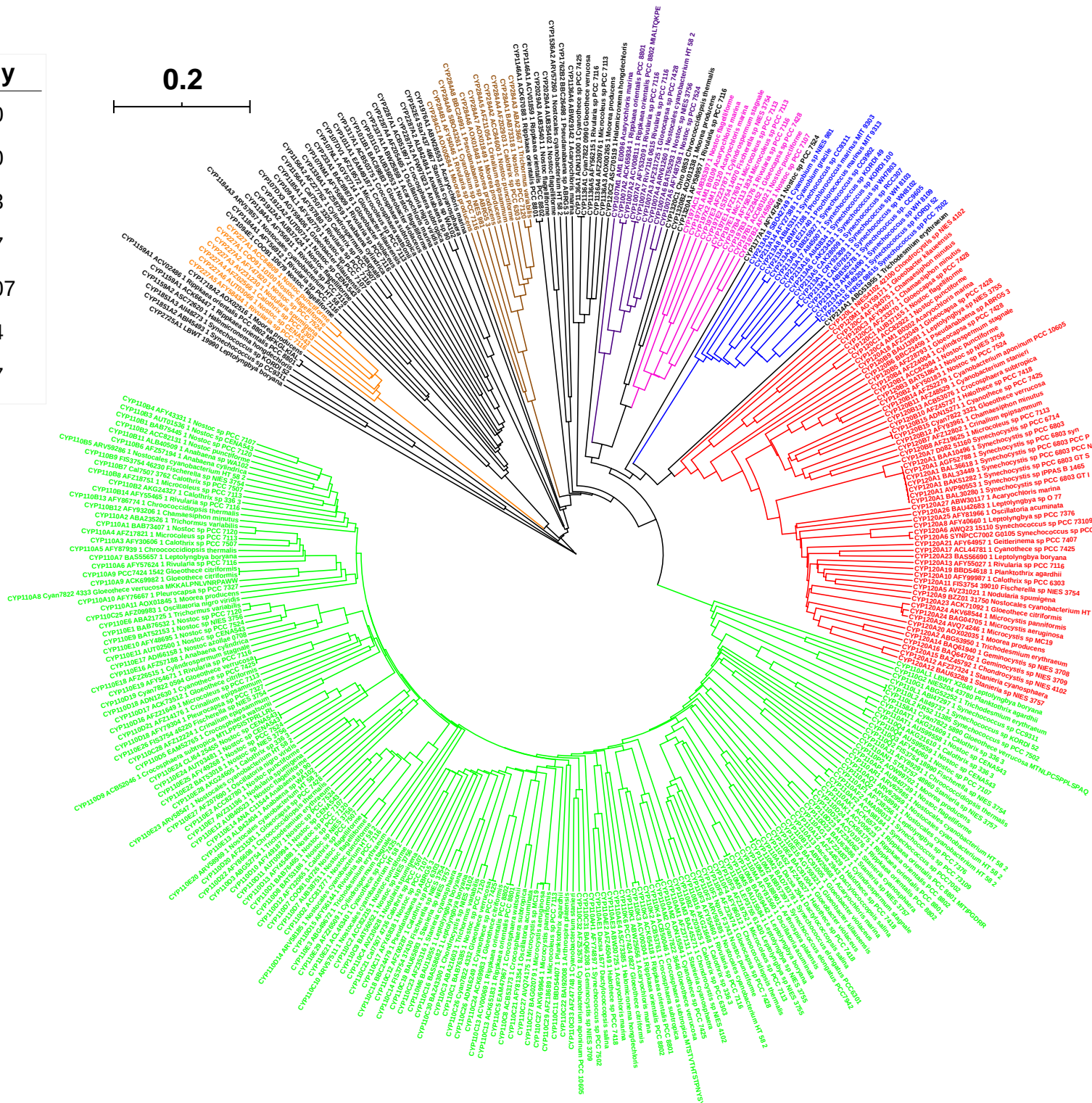

Supplement: Supplementary file 1 [file ijms-21-00656-s001.zip › Khumalo et al. 2019 supplementary files/Supplementary Dataset 2.pdf]
